# Transcriptomic profiling of the medicinal plant *Clitoria ternatea*: identification of potential genes in cyclotide biosynthesis

**DOI:** 10.1038/s41598-020-69452-7

**Published:** 2020-07-29

**Authors:** Neha V. Kalmankar, Radhika Venkatesan, Padmanabhan Balaram, Ramanathan Sowdhamini

**Affiliations:** 1 National Centre for Biological Sciences (TIFR), GKVK Campus, Bangalore, Karnataka 560065 India; 2 The University of Trans-Disciplinary Health Sciences and Technology (TDU), #74/2, Jarakabande Kaval, Post Attur, Via Yelahanka, Bangalore, Karnataka 560064 India; 3 Present Address: Department of Biological Sciences, Indian Institute of Science, Education and Research, Kolkata, Mohanpur, West Bengal 741246 India; 4 Molecular Biophysics Unit, Indian Institute of Science, Bangalore, Karnataka 560012 India

**Keywords:** Bioinformatics, Genomics, Plant biotechnology, Biotechnology, Computational biology and bioinformatics, Structural biology

## Abstract

*Clitoria ternatea* a perennial climber of the Fabaceae family, is well known for its agricultural and medical applications. It is also currently the only known member of the Fabaceae family that produces abundant amounts of the ultra-stable macrocyclic peptides, cyclotides, across all tissues. Cyclotides are a class of gene-encoded, disulphide-rich, macrocyclic peptides (26–37 residues) acting as defensive metabolites in several plant species. Previous transcriptomic studies have demonstrated the genetic origin of cyclotides from the Fabaceae plant family to be embedded in the albumin-1 genes, unlike its counterparts in other plant families. However, the complete mechanism of its biosynthesis and the repertoire of enzymes involved in cyclotide folding and processing remains to be understood. In this study, using RNA-Seq data and de novo transcriptome assembly of *Clitoria ternatea*, we have identified 71 precursor genes of cyclotides. Out of 71 unique cyclotide precursor genes obtained, 51 sequences display unique cyclotide domains, of which 26 are novel cyclotide sequences, arising from four individual tissues. MALDI-TOF mass spectrometry analysis of fractions from different tissue extracts, coupled with precursor protein sequences obtained from transcriptomic data, established the cyclotide diversity in this plant species. Special focus in this study has also been on identifying possible enzymes responsible for proper folding and processing of cyclotides in the cell. Transcriptomic mining for oxidative folding enzymes such as protein-disulphide isomerases (PDI), ER oxidoreductin-1 (ERO1) and peptidylprolyl *cis-trans* isomerases (PPIases)/cyclophilins, and their levels of expression are also reported. In particular, it was observed that the CtPDI genes formed plant-specific clusters among PDI genes as compared to those from other plant species. Collectively, this work provides insights into the biogenesis of the medicinally important cyclotides and establishes the expression of certain key enzymes participating in peptide biosynthesis. Also, several novel cyclotide sequences are reported and precursor sequences are analysed in detail. In the absence of a published reference genome, a comprehensive transcriptomics approach was adopted to provide an overview of diverse properties and constituents of *C. ternatea*.

## Introduction

Macrocyclics such as the cyclotides are a class of cyclic peptides (26–37 residues) containing three disulphide bonds. They are formed by cyclization of a gene encoded, linear precursor in specific plant species. In addition to the circular backbone, they form a cyclic cystine knot (CCK) arrangement formed by a conserved six cysteine framework (Cys I–Cys IV, Cys II–Cys V, Cys III–Cys VI)^1–3^. This knotted arrangement along with the circular backbone, renders these cyclotides exceptionally stable against enzymatic, chemical and thermal degradation, with their principal function thought to be in plant defense^4^. Thus, cyclotides are potential candidates for peptide-based drugs; either as scaffolds to stabilize susceptible peptide sequences or as drugs themselves^5^. To date, ~ 400 cyclotides have been sequenced and are largely from Rubiaceae, Violaceae, Fabaceae and others. A major challenge, however, is deciphering how their unique structural topology arises from linear precursors.

*Clitoria ternatea*, a perennial climber of Fabaceae family, commonly known as ‘Butterfly pea’ or Shankhapushpi (in India), has been used in traditional Ayurvedic medicine as a memory enhancer, nootropic, antidepressant, anticonvulsant, blood platelet aggregation-inhibitor, tranquilizing and sedative agent. Its extracts possess a wide range of pharmacological activities including antimicrobial, antipyretic, anti-inflammatory, analgesic, antidiabetic, etc^6–10^. Initial phytochemical screenings of *C. ternatea* have shown that the biologically active ingredients are rich in peptides and depleted in alkaloids, flavonoids, saponins, lignans, etc^7,11^. As the primary mode of extraction was boiling, it is plausible to infer that the active agents are in fact heat-stable proteins^12^. Poth et al*.,* had previously characterized 12 cyclotides (Cter A–L) from the seed extracts^13^. They also showed that Asn/Asp residues could occur at the cyclization site more commonly than previously understood. Later, they isolated 6 novel cyclotide sequences (Cter M–R) from leaf and flower tissues, and also determined the NMR structure of the chemically synthesized cyclotide Cter M^14^. Independently, Nguyen et al., screened for cyclotides in *C. ternatea* whole plant and reported 21 novel sequences and designated as cT1–21, of both cyclic and acyclic forms, and named them as ‘cliotides’^12,15^. Presently, there are 85 cyclotide sequences reported from *C. ternatea*, out of which 74 have come from transcriptomic approaches^16^, but given the abundance and potential variation of these macrocyclics, a larger number and diversity can be reasonably expected.

All the transcriptomic evidences from *C. ternatea* by various scientific groups have pointed out the occurrence of a single cyclotide domain in Fabaceae family. Unlike its counterparts Violaceae, Rubiaceae, Cucurbitaceae and Solanaceae, Faboideae possess pea-like albumin-1 genes^13,17–20^. These albumin-1 gene architecture contain a signal peptide, followed by a shorter albumin-1b chain, a short ~ 10 aa intervening linker region and ~ 50 aa long albumin-1a chain. Gilding et al., have demonstrated the evolution and co-option of the albumin-1b chain by the cyclotide domain. More importantly, they showed that the distribution of cyclotides in the plant tissues depends on the kind of herbivore attack, and suggested a combinatorial cyclotide defense^21^.

Asparaginyl endopeptidase (AEP, EC 3.4.22.34) or vacuolar-processing enzyme (VPE) are a class of cysteine proteases that recognize and target Asn/Asp residues for peptide bond hydrolysis. It was established a while ago that AEPs not only perform cleavage, but also have the enzymatic capability of ligating linear peptides^3^. Plants that lacked AEP produced acyclic versions of cyclotides. However, Nguyen et al., in 2014 reported the first experimental evidence on the ligation property of AEP in *C. ternatea*. It was termed butelase-1 and shown to be highly efficient in intermolecular peptide bond formation, even on non‐native-targets^22,23^. Prior and subsequent studies showed that mutations of Asn/Asp residue at the C-terminus of target proteins resulted in reduced cyclizing ability of the enzyme, implying the functional importance of an Asn/Asp residue in loop 6 of the cyclotide domain^24–27^. Since then several AEPs have been discovered in plants that make cyclotides, such as OaAEPs from *Oldenlandia affinis*, PxAEPs from *Petunia x hybrida* and HeAEP from *Hybanthus enneaspermus*^28,29^. Bioinformatics analysis on sequence and structural differences between the conventional AEPs (proteases) and dual functioning AEPs (proteases and ligases) needs to be explored.

The disulphide-bond formation is primarily catalysed by an ER-resident protein disulphide isomerase (PDI, EC 5.3.4.1), thereby acting as ER-chaperones^30–32^. PDIs (~ 50 kDa size) are oxidoreductase enzymes belonging to the thioredoxin superfamily. The classical PDI (hPDI) is composed of four thioredoxin domains (**a**, **b**, **b′**, **a′**) and an additional short α-helical **c** domain at the C-terminus. **a** and **a′** are the catalytically active domains characterized by the presence of a ‘CX_1_X_2_C’ motif, where X_1_ and X_2_ are mostly glycine and histidine residues^33^. The **b** and **b′** domains also bear the thioredoxin-like fold but are redox-inactive domains and do not bear the catalytic ‘CX_1_X_2_C’ motif. Also, C-terminal end of PDI sequences, bear an ER-retention signal composed of a highly conserved ‘[K/H/N][D/E]EL’ motif^30,34,35^. While, PDIs have been identified in several plant systems, there is no conclusive evidence for the involvement of PDIs in cyclotide biogenesis, except for one study that shows increased yields of folded kalata B1 in the presence of a disulphide isomerase isolated from *O. affinis* (OaPDI)^36^. To the best of our knowledge, despite all the transcriptomic evidence reported on cyclotide-producing plants, no reports on the sequences of PDIs have been reported in such *planta*. Here, we obtained several *C. ternatea* PDI sequences by transcriptomic mining and the functional annotation has been established by mapping all the functionally important residues of plant PDIs.

PDIs oxidize cysteine residues in the substrate polypeptides and become reduced. One of the enzymes involved in PDI reoxidation and activation is the endoplasmic reticulum oxidoreductin-1 (ERO1). This in conjunction with flavin adenine dinucleotide (FAD) cofactor generates and transfers disulphide bonds to PDI and its family members^37–40^. A recent study reports the identification of a *Conus* ERO1 and its role in the oxidative folding of conotoxins, a class of disulphide-rich peptides from venom glands, similar to the cyclotides^41^. The oxidative folding capacity of both ERO1 and its homolog ERO2 was investigated in plants using transgenic *A. thaliana* only recently^42^*.* Exactly how the PDIs are oxidized by an oxidoreductase upon cyclotide folding remains to be investigated. To address this, we have identified an ERO1 for the first time from *C. ternatea* transcriptome and compared it with yeast, human and plant ERO1 sequences.

Cyclotides are structurally grouped into three subfamilies, Möbius, Bracelet and trypsin inhibitor^2^. The core CCK topology is conserved in all subfamilies, but the amino acid composition in the six loops varies substantially. Möbius subfamily differs from the Bracelet subfamily by the presence of a *cis*-Pro peptide bond in loop 5 (intervening residues between Cys V and Cys VI). The majority of the cyclotide sequences identified to date belong to the Bracelet subfamily^43^, but the reason for this preference is unclear. Given the presence of a key *cis*-Pro peptide bond in loop 5, an enzyme mediated *cis-trans* isomerization reaction is likely to facilitate the protein folding. Peptidylprolyl *cis-trans* isomerases (PPIases, EC 5.2.1.8), also known as cyclophilins (CYP) and immunophilins, catalyze the *cis-trans* isomerization of proline residues and are ubiquitously distributed^44^. The role of PPIases in folding small disulphide-rich peptides is not well understood except on maurotoxin, a four disulphide-bridged scorpion toxin^45^ and on conotoxins in venom glands of cone snails^46,47^. In the present study, we have performed a comprehensive mining of PPIases genes and identified CYP gene members and their expression patterns in the *C. ternatea* transcriptome.

**Figure 1:**
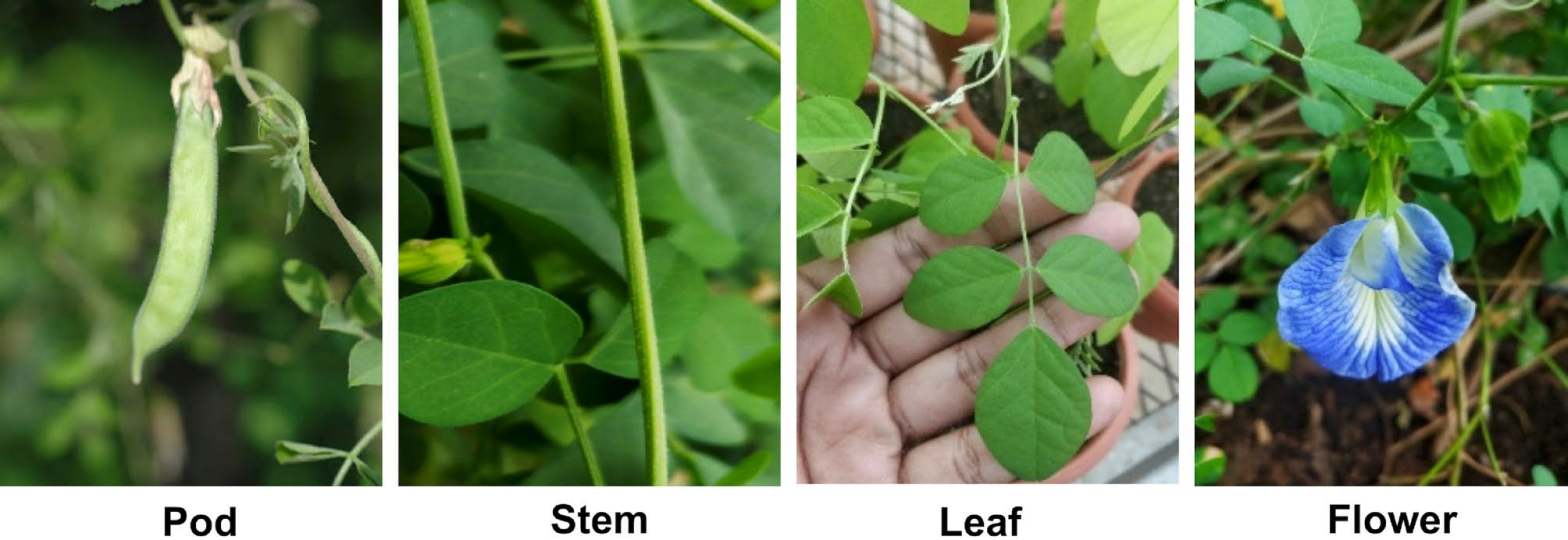
Morphology of *Clitoria ternatea* pods, stems, leaves and flowers.

Despite all the RNA-seq experiments on *C. ternatea*, a systematic functional annotation of a transcriptome assembly is yet to be performed. Here we present the de novo transcriptome assembly and annotation of *C. ternatea* plant (pod, stem, leaf and flower organs; Fig. 1), and report the presence of 26 new cyclotides that do not show high sequence similarity to those observed in previous studies. We have specifically investigated the expression of enzyme-coding genes such as asparaginyl endopeptidase (AEP), protein disulphide isomerase (PDI), ER oxidoreductin-1 (ERO1) and peptidylprolyl *cis-trans* isomerase (PPIase), which are key enzymes directly or indirectly involved, providing a concerted effect in the proper oxidative folding of disulphide-rich polypeptides, such as the cyclotides, in the cell.

## Materials and methods

### RNA preparation and sequencing

For transcriptome sequencing, RNA was extracted from four tissues of *C. ternatea* grown the on campus of National Centre for Biological Sciences, Bangalore (13°04′13.8″N 77°34′49.9″E). Samples were obtained from leaf, stem, pod and flower tissues (Fig. 1). Root tissue could be included in the current study, as quality-RNA from the root could not be purified. RNA was extracted using an RNeasy Plant Mini Kit (Qiagen, Inc.) as per manufacturer’s guidelines. A total of 16 libraries were obtained from two biological replicates per tissue with two technical replicates per biological sample. RNA integrity and concentration were evaluated on a Bionalyzer (Agilent, Inc.). Samples with RNA integrity number (RIN) ≥ 6.5 were submitted to AgriGenome Labs Pvt. Ltd. (Kochi, India) for library preparation and sequencing using Illumina HiSeq 2500 platform V4 chemistry, to yield 100-bp paired-end reads (Raw data summary is detailed in Supplementary Table S1).

### Transcriptome assembly and validation

Before transcriptome assembly, the quality of reads was assessed using FASTQC (v0.11.8) (www.bioinformatics.babraham.ac.uk/projects/fastqc/). A total of 293 million reads were obtained from 16 libraries. The raw reads were trimmed, to remove any low quality sequences or adapter contamination, using Trimmomatic v0.39^48^ (parameters: *LEADING:3 TRAILING:3 SLIDINGWINDOW:4:15 MINLEN:36)* and the trimmed reads was further used for assembly. The trimmed reads were de novo assembled using TRINITY assembler v2.6.6^49^ (parameters: –seqType fq—normalize_by_read_set—left reads_1.fastq—right reads_2.fastq). A total of 278,991 transcripts were obtained with a mean contig of 1,441.89 bp and an N50 of 2,437 bp. We identified the candidate coding regions within the de novo assembly using TransDecoder v5.5.0 (https://transdecoder.github.io). First step in TransDecoder is TransDecoder.LongOrfs, which selects long open reading frame (ORF) per transcript (parameters: ORF in all 6 reading frames, minimum ORF amino acid length-m 50). The second step, TransDecoder.Predict predicts the likely coding regions. A total of 387,057 candidate genes were obtained. The transcriptome assembly was assessed for its completeness by using BUSCO v3 (Benchmarking Universal Single-Copy Orthologs)^50^ on three datasets: Embryophyta_odb10, Eudicotyledons_odb10 and Viridiplantae_odb10.

### Functional annotation and orthology analysis

Gene Ontology (GO) annotation was performed using OrthoVenn with an E-value cut-off of 10^−10^.^51^ Orthologous groups of *C. ternatea* encoded proteins with four other Fabaceae plant species *i.e. Glycine max*, *Medicago truncatula*, *Phaseolus vulgaris*, *Vigna unguiculata* and model plant *Arabidopsis thaliana* were obtained using OrthoVenn with an E-value cut-off of 10^−10^.

### Cyclotide precursor search

Protein and nucleotide sequences of published cyclotides were extracted from Cybase and UniProt^52,53^. These were used as query sequences to search our *C. ternatea* transcriptome through various BLAST methods (v.2.7.1)^54,55^. In parallel, functional motifs of cyclotide, corresponding to two familes (PS51052, PS60008 (Bracelet) and PS60009 (Möbius)) were queried using PROSITE^56,57^ against our transcriptome database for pattern matching. The combined BLAST and PROSITE hits were manually inspected for authenticity based on sequence conservation with known cyclotide sequences and redundancy was removed. The protein sequences were aligned through Clustal Omega (Supplementary Data 1)^58^ and visualized using Unipro UGENE software^59,60^. The motif discovery algorithm (MEME Suite v5.1.0) was utilized for analysis of conserved motifs in cyclotide precursor sequences^61^.

### Identification of biosynthetic enzymes

The *C. ternatea* transcriptome was screened for several processing proteins AEP, PDI, ERO1 and PPIases that are known or likely to participate in the biosynthesis of cyclotides. Sequences of interest for each classical member of these enzyme/protein families, extracted from Uniprot, were used as queries to mine homologous sequences in the transcriptome using BLASTp (seq. cov. cut-off =  ≥70%; seq. ID cut-off =  ≥30%)^62^. The resulting hits were manually inspected for redundancy removal and identification. Identification involved deletion of signal peptides predicted by SignalP-5.0^63^ and mapping of functionally important residues (FIR) onto a multiple sequence alignment of the enzymes. The protein sequences were aligned through Clustal Omega (Supplementary Data 1)^58^ and visualized using BoxShade (https://sourceforge.net/projects/boxshade/).

### Phylogenetic analysis

Neighbor-joining method was used to construct and understand phylogenetic relationships between the cyclotide transcript obtained from our assembly and all the known peptides reported from *C. ternatea*. The Maximum Likelihood method was used to construct phylogeny trees for the implicated biosynthetic enzyme transcripts from *C. ternatea* with orthologs from various species under Viridiplantae. Sequences of transcripts of interest were aligned with all the previously canonical examples using Clustal Omega and manually curated^58^. Phylogenetic construction of the final alignments for various queries discussed in this study were performed using MEGA version X^64^ with the Neighbor-Joining/Maximum Likelihood method and nodal supports were calculated with 10,000 bootstrap iterations. The phylogenetic trees (bootstrap consensus) were visualized and imaged using FigTree v1.4.4^65^ or TreeDyn 198.3^66^. All the protein sequences obtained in the current transcriptome and from other species are recorded in Supplementary Data 1.

### Transcript quantification & differential expression analysis

RSEM was used to analyse and quantify the variation in gene expression across the four tissues^67^. The transcript abundance and Transcripts Per Million (TPM) was calculated by mapping raw reads to the assembled transcriptome. Clustering of TPMs of cyclotide genes was performed using Pearson correlation coefficient method with complete linkage and visualized with ClustVis webtool (https://biit.cs.ut.ee/clustvis)[68]. The TPM values for all the genes of interest and across the four tissues are provided in Supplementary Table S2.

### Peptide extraction

Fresh plant parts (leaves, stems, flowers & pods) from *C. ternatea* were collected and oven dried at 70 °C. The peptide extraction was performed from literature protocols^12–15,69^. Briefly, plant material was extracted with 1:1 DCM: MeOH (v/v) with overnight stirring. The extract was partitioned with 1:1 mixture of DCM: (MeOH: ddH_2_O, 2:3) using liquid–liquid phase extraction. MeOH/ddH_2_O phase was further fractionated on RP-C18 column (0%, 30%, 50%, 70% and 100% gradient elution using ACN/ddH_2_O). Initial screening of cyclotide-like masses in each elute was performed using MALDI-TOF mass-spectrometry following the protocol mentioned in Section 2.10 of Materials and Methods. Elutes 50%, 70% and 100% showed cyclotide-like masses (~ 3,000 Da). These elutes were pooled and freeze-dried for further analysis and will be referred to as “crude extract”.

### HPLC fractionation

The crude extract was analysed via LC–ESI–MS on a Maxis Impact Q-TOF mass spectrometer (Bruker Daltonics, Germany) interfaced to an Agilent 1,260 HPLC system. Samples were run on a reverse phase Agilent Poroshell C18 column (2.7 mm, 4.6 × 150 mm) using a linear gradient of 5%–95% ACN/ddH_2_O, with 0.1% formic acid, and flow-rate of 0.2 mL/min. Data acquisition and analysis were performed using Bruker Data Analysis v4.1 software. The ESI–MS data obtained for *C. ternatea* crude extracts is not included in this study. HPLC fractionation of the crude extracts was performed using a Shimadzu Prominence series equipped with a binary pump, autosampler, PDA detector, and fraction collector. Chromatography was performed using a semi-preparative Phenomenex Proteo C18 column (250 × 10 mm, 10 μm, 110 Å) at flow-rate of 3 mL/min. A linear gradient of 1% min^−1^ of 0–95% buffer B (100% ACN, 0.1% trifluoroacetic acid) was applied, and the eluents were monitored at 254, and 280 nm. Late-eluting peaks were separated into five fractions (A–E) for each plant tissue, lyophilized for further analysis and will be referred to as “purified fraction”.

### Cyclotide analysis by MALDI-TOF mass spectrometry

Each of the five purified fractions (A–E), per tissue, was subjected to MADLI-TOF mass-spectrometry (UltraFlex Bruker Daltonics) analysis with 50 Hz pulsed nitrogen laser (λ = 337 nm), in positive ion reflectron mode. The samples were prepared by mixing an equal amount of purified fractions (0.5 µL) with matrices α-cyano-4-hydroxycinnamic acid (CCA) or 2,5-dihydroxy benzoic acid (DHB) prepared in 1:1 mixture of H_2_O-ACN with 0.1% triflouroacetic acid, spotted on a stainless-steel target plate and air dried. The data were analysed using Flex Analysis v3.3.74 software. Identification of cyclotides was performed by matching the observed monoisotopic masses and calculated molecular weights of cyclotide transcript sequences.

## Results and discussion

### *Clitoria ternatea* transcriptome assembly, validation and annotation

A total of 281.8 million quality reads were assembled into a complete de novo assembly (details of assembly is provided in Supplementary Table S3). Quality of the assembly was assessed by examining the RNA-Seq read representation in the transcriptome by mapping reads back to the assembly using Bowtie2^70^. ~ 95% of the read fragments mapped as proper pairs and concordantly aligned 1 or more times to the transcriptome. Benchmarking Universal Single-Copy Orthologs (BUSCO) revealed the presence of 95.5% of complete BUSCOs out of the 1,375 orthologues searched from Embryophyta dataset, 94.5% of the 2,121 orthologues searched from Eudicotyledons dataset and 97.7% of the 430 orthologues searched from Viridiplantae dataset.

### Gene orthology and functional annotation of *Clitoria ternatea* transcriptome

Gene orthology analysis was performed using the predicted proteome of *C. ternatea* against proteomes of closely related species such as *Glycine max*, *Medicago truncatula*, *Phaseolus vulgaris*, *Vigna unguiculata* and *A. thaliana* using OrthoVenn tool*.* 10,778 orthogroups are common to all these species (Supplementary Fig. S1A). The plants form 57,821 orthologous clusters (at least contain two species) and 572 single-copy gene clusters. *C. ternatea* shares 753 and 494 orthogroups with closely related species *G. max* and *M. truncatula*, respectively. Gene Ontology (GO) annotation categories were assigned for *C. ternatea* and shown in Supplementary Fig. S1B. 24,950 orthogroups are specific to *C. ternatea* as seen in Supplementary Fig. S1A, and GO term enrichment analysis of these species-specific orthogroups show top GO-term hits with respect to stress-related genes, defense-response genes and serine-type endopeptidase activity, apart from the usual cell-processes genes (Supplementary Table S4).

### Cyclotide identification in the transcriptome assembly

**Figure 2:**
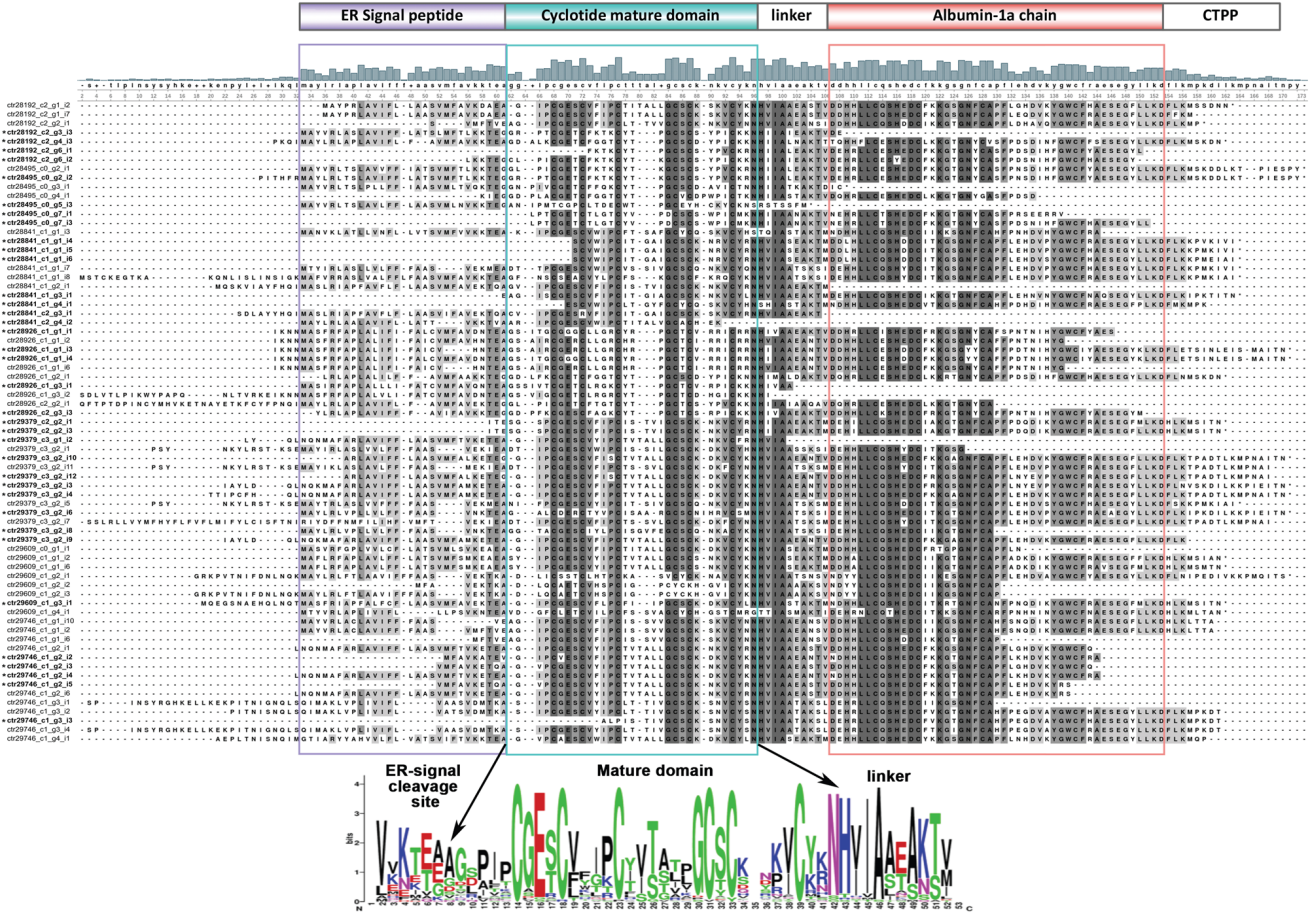
Multiple sequence alignment of 71 cyclotide precursor proteins obtained from the de novo transcriptome assembly of *C. ternatea*. Novel cyclotide sequences are highlighted in bold and an asterisk ‘*’, alongside the transcript ID. ER-signal peptide highlighted in purple, cyclotide mature domain highlighted in cyan and albumin-1a chain highlighted in red. Sequence logo of the cyclotide mature domain, ER-signal cleavage site and linker region, generated using MEME Suite^61^, is presented at the bottom of the figure. Key residues such as the cyclization site containing conserved Asn/Asp and membrane-binding conserved Glu residue is highlighted in red with arrows.

To mine cyclotide-coding genes in the transcriptome, mature cyclotides reported from *C. ternatea,* deposited in Cybase, were used as queries and a total of 3,753 redundant hits was obtained. The hits were filtered to remove redundancy and resulted in a final set of 71 unique cyclotide precursor genes. Out of 71 unique cyclotide precursor genes obtained, 51 displays unique cyclotide domain (redundant hits encode the same cyclotide but different albumin and/or ER-signal region), of which 26 are novel cyclotide domains that have not been reported earlier (Supplementary Table S5). These data expands our understanding of the cyclotide repertoire found in *C. ternatea* plant. RNA-seq and proteomic experiments from various groups have facilitated cataloguing of at least 85 cyclotide sequences produced in *C. ternatea* and independently we have identified additional new sequences. A comparison of the numbers reported by other groups and our group is depicted in Supplementary Fig. S2^15,16,21^. Figure 2 shows the multiple sequence alignment of all the 71 cyclotide precursor genes mined from the *C. ternatea* transcriptome. The figure highlights the ER signal peptide, immediately followed by the cyclotide mature domain, linked by a 10-residue linker and co-occurring with the albumin-1a chain, and a short ~ 10 residue C-terminal propeptide tail. Characterization of ‘cliotide’ genes from various transcriptomic approaches suggest a unique architecture in Fabaceae, that differs greatly from Violaceae and Rubiaceae cyclotide genes^15,20,21,69,71,72^. The cyclotide gene arrangement in *C. ternatea* is very similar to that of albumin gene of many legume species, wherein a signal peptide is followed by an albumin-1b domain, a linker region, and an albumin-1a domain. Similarities in arrangement and conservation of residues between albumin-1b and cliotide sequences suggests the albumin-1b domain could have been adapted into a cyclotide domain^21^. Out of 71 cyclotide precursor transcripts, 42 of them displayed nearly complete ER signal peptide region. 53 out of 71 hits displayed nearly complete albumin-1a domain. Figure 2 shows that the cyclotide mature peptide is cleaved off from the ER signal peptide at the highly conserved Thr-Glu-Ala (TEA) motif, most probably by a signal peptidase-1 enzyme^12^. Nine of the 71 precursor transcripts were partial sequences, lacking either 5′ end or 3′ end, or both.

**Figure 3:**
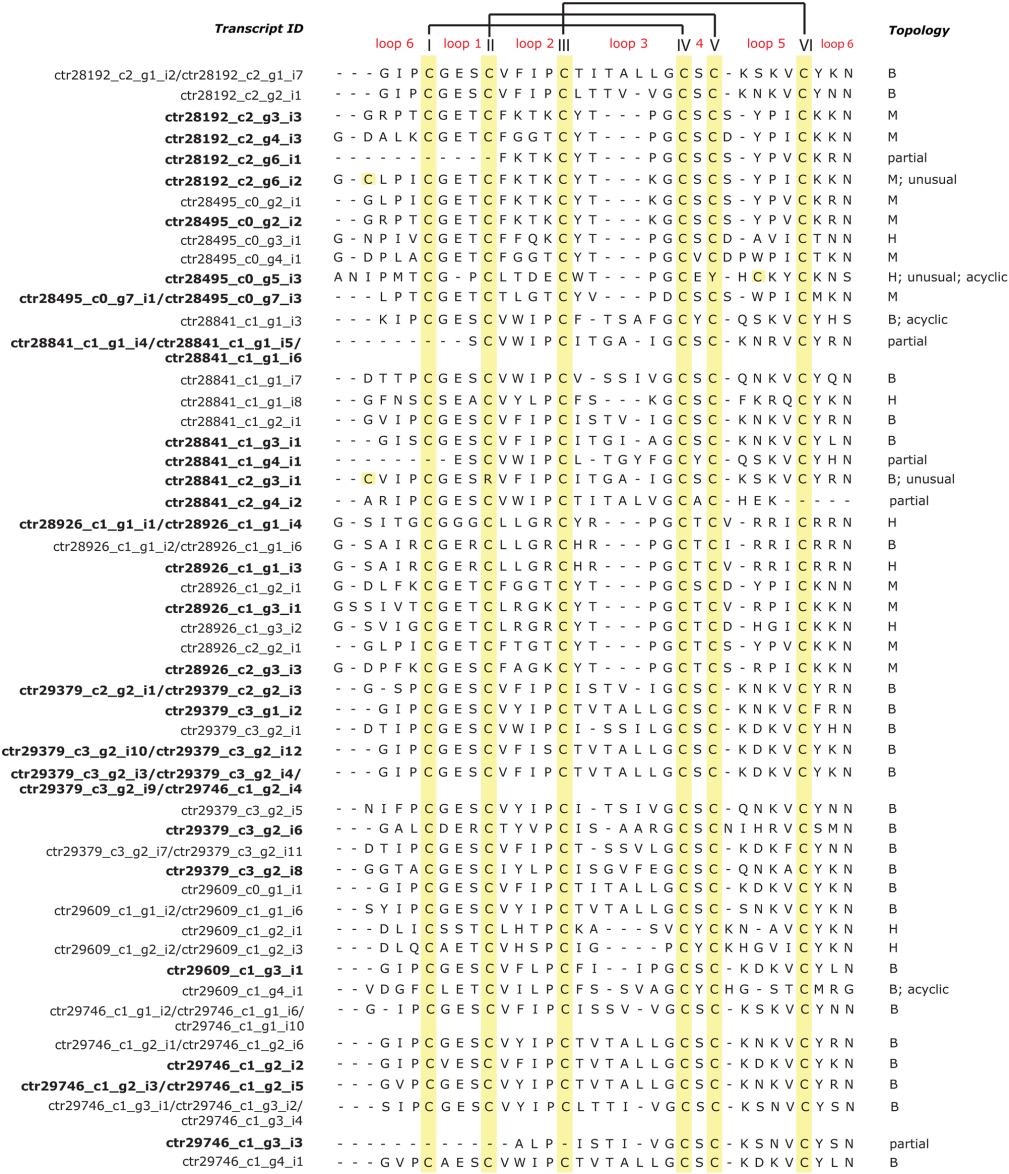
Sequence alignment of 51 unique cyclotide domains observed in *C. ternatea* transcriptome assembly. The transcript IDs of precursor genes corresponding to a unique cyclotide domain, are mentioned. Novel cyclotide domains are highlighted in bold. Cyclotide loop numbers are indicated above the alignment and the conserved cysteine residues are highlighted in yellow boxes. Based on sequence signatures of known cyclotides, the domains are classified as M-Möbius, B-Bracelet or H-Hybrid topology.

Interestingly, several sequences display novel residue stretches at the N-terminal side of the cyclotide domain (Fig. 3). Furthermore, the starting site was not necessarily a glycine residue and included residues such as valine, serine, alanine and aspartic acid, contrary to some of the early reports on cyclotides. However, an asparagine residue at the C-terminal processing site was highly conserved (Fig. 3), suggesting that a non-Gly residue can possibly undergo a transpeptidation reaction with the conserved Asn residue. Three sequences lacked the C-terminal asparagine, implying that these could form acyclic products. Cyclotides with Möbius topology were seen in 11 cases, whereas Bracelet topology was seen in 27 cases, and Hybrid topology^73,74^, i.e. bearing loop 2 and 3 similar to the Möbius and but lacking the *cis*-Pro in loop 5, were seen in 8 cases. One sequence contained an additional cysteine residue in the intervening loop 1 of the cyclotide domain (ctr28192_c2_g6_i2) and two sequences displayed atypical cysteine frameworks (ctr28495_c0_g5_i3 and ctr28841_c2_g3_i1) and these were termed as “unusual” cyclotide transcripts (Fig. 3).Interestingly, one the most abundant cyclotides known to be produced in *C. ternatea*, Cter M, and the only representative with a known structure^13^, was obtained as a truncated sequence in our transcriptome assembly. The transcripts, ctr28495_c0_g7_i1 and ctr28495_c0_g7_i3, lacks the ER-signal peptide region and the starting glycine residue of the cyclotide domain.

Out of the 51 unique cyclotide domain containing transcripts, five were truncated sequences. Some of the challenges of a de novo assembly are misassembly, sequencing errors and redundancy problems that couldlead to truncation of transcripts. We have explored whether the partial cyclotide transcripts could be further extended by mapping them to the raw reads. We were able to extend the incomplete transcripts to at least contain the complete cyclotide domain by manually searching through the raw reads. The results for these partial cyclotide transcripts (ctr28192_c2_g6_i1, ctr28841_c1_g1_i4, ctr28841_c1_g4_i1, ctr28841_c2_g4_i2 and ctr29746_g1_g3_i3) are provided in the Supplementary Data 2. However, as these extension of boundaries at the 5′ or 3′ ends are merely predicted information, the sequences have been considered originally as partial sequences in this study.

**Figure 4:**
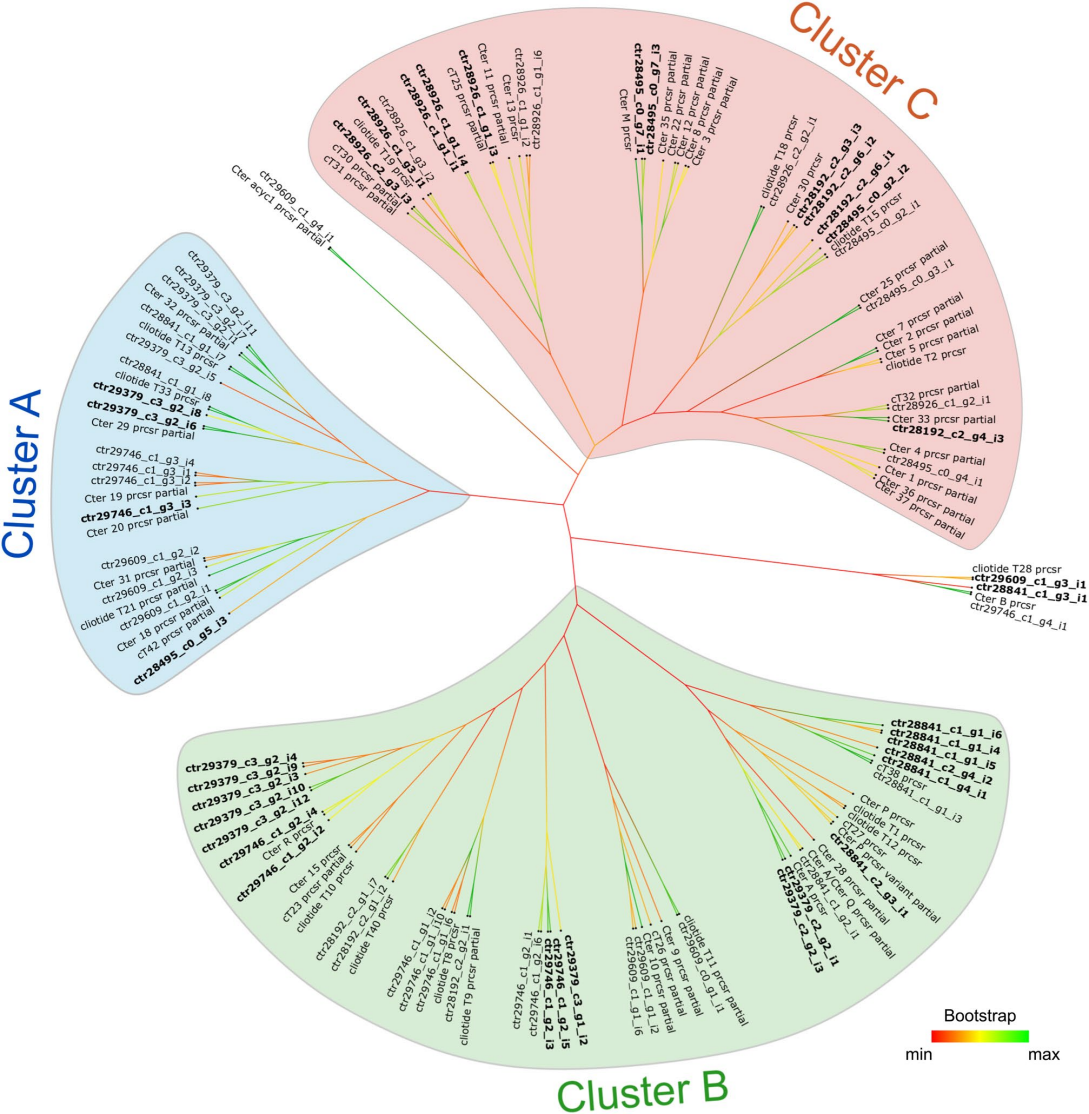
Phylogenetic tree of cyclotide precursor proteins from *Clitoria ternatea* (our transcriptome hits with all previously reported sequences from *Clitoria ternatea*). The tree contains 130 precursor sequences in total and was constructed using Neighbor-joining (NJ) method using MEGA version X software^64^. Three distinct cluster are highlighted as cluster A (blue), B (green) and C (red). Novel cyclotides obtained in the current study is highlighted in bold font. Bootstrap values for the unrooted tree is presented as a colour code ranging from minimum (red), mid-point (yellow) and maximum (green). “prcsr” = precursor.

Figure 4 represents the phylogenetic relationship between cyclotide precursor proteins from our transcriptome and those reported earlier. Cluster A represents 16 transcripts, 13 of which are classical *i.e.* bearing the typical ER-signal peptide, immediately followed by the mature cyclotide domain, a linker region and albumin-1a chain. The remaining three precursors are truncated and possess atypical N-terminal motif in the cyclotide mature domain (but end with an asparagine at the C-terminus). Transcript ctr28495_c0_g5_i3 from cluster A bears least similarity with the conventional ‘cliotide’ sequences, displaying unusual cysteine framework and lacks the required asparagine at the C-terminus. Notably, cluster A represents primarily a Bracelet family of ‘cliotides’, having longer loop 3 and lacking the proline residue in loop 5. Transcripts ctr29609_c1_g2_i1, ctr29609_c1_g2_i2, ctr29609_c1_g2_i3 and ctr28841_c1_g1_i8 are an exception to this rule, displaying shorter loop 3. Cluster B highlighted in Fig. 4 represents 31 members, of which 18 carry the ER-signal peptide and the remaining 13 transcripts are partial. Cluster B also displays high sequence similarity with known Bracelet family members of cyclotides, having a glycine at the N-terminus and a conserved asparagine at the C-terminus and classical sequence architecture. Cluster C contains 20 members, 13 of which have the ER-signal peptide followed by cyclotide domain. The remaining 7 transcripts are partial sequences. Most sequences in this cluster contain the conserved glycine at the N-terminus and all members bear the crucial asparagine at the C-terminus. However, transcript ctr28192_c2_g6_i2 contains an additional cysteine residue in the cyclotide domain at the N-terminus apart from the conventional six conserved cysteines. Interestingly, cluster C represents the Möbius family of cyclotides, displaying shorter sequence lengths, with 65% of the hits bearing the *cis*-Pro residue in loop 5 of the cyclotide mature domain. Out of the 71 precursor protein sequences, 4 transcripts do not cluster with any of the above and remain as outliers. Transcript ctr29609_c1_g4_i1 sequence shows high dissimilarity with most cyclotide precursor sequences, showing unusual stretches in the ER-signal region, cyclotide domain and linker region. Despite the presence of six conserved cysteine framework, it lacks the C-terminal asparagine residue required for cyclization step. The sequence is reported previously and named as ‘Cter acyc1’ precursor^21^. Occurrence of uncommon precursor sequences hint at the divergent evolution of cyclotides from the ancestral albumin gene. Transcript ctr29609_c1_g3_i1 clusters and shares 67% sequence identity with cliotide T28 (also known as Cter 16) precursor sequences^15,21^. Transcript ctr29746_c1_g4_i1 clusters with Cter B precursor sequence^14^, sharing 98% sequence identity. The MEME logos of motifs predicted for the above three phylogenetic clusters are presented in Supplementary Fig. S3.

### Differential expression of cyclotides

**Figure 5:**
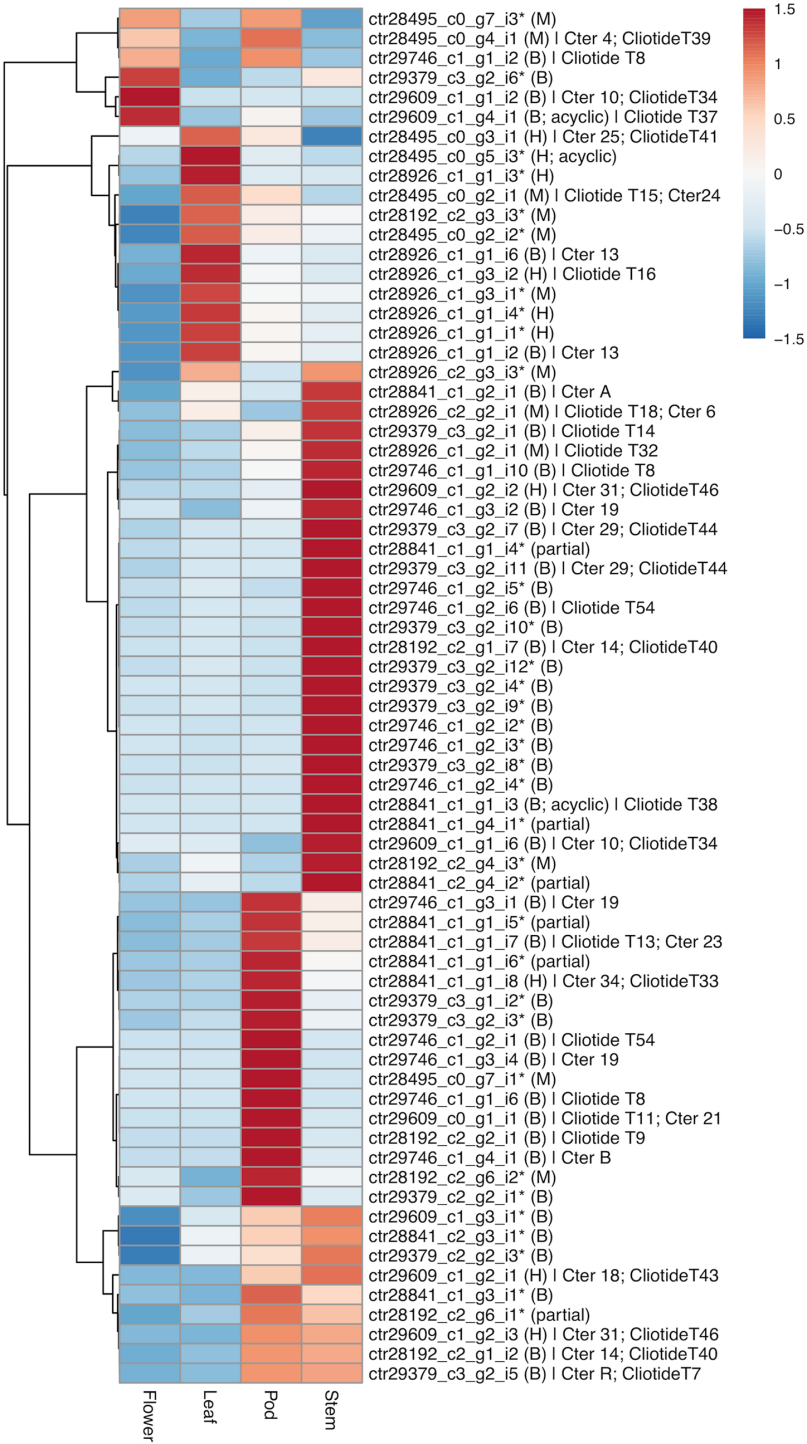
Heatmap of differential expression of cyclotide genes across four tissues of *C. ternatea *de novo transcriptome. The heatmap was generated using average TPM values and Pearson correlation coefficient clustering method with complete linkage on genes, using ClustVis tool^68^. Möbius (M) and Bracelet (B) topologies are highlighted in brackets and novel cyclotide sequences are highlighted with an asterisk ‘*’, alongside the transcript ID. Positive (red) and negative (blue) values correspond to up-and down-regulated clusters, respectively.

Transcriptome mining undertaken in this study resulted in a diverse set of cyclotide genes with reasonably high sequence diversity, all ranging from typical topologies of Möbius and Bracelet, to Hybrids and acyclic gene products. To find relationships between expression of different cyclotide sequences and tissue localization, we performed a hierarchical clustering of TPM (Transcript per million) values of cyclotide genes using Pearson correlation coefficient method with complete linkage. Figure 5 illustrates three major transcript clusters across the tissues, in terms of expression profiles. Pods and stem displayed high expression of most cyclotide genes, while leaves and flowers showed relatively lesser expression values. Most of the members of the Bracelet topology of cyclotides show significantly high expression in pods and stems, whereas members of the Möbius and Hybrid topology cluster mostly in leaf tissue. Furthermore, genes classified as ‘unusual’ were mostly found in the leaf cluster, as seen in Fig. 5.

Novel transcripts ctr28926_c2_g3_i3 and ctr28495_c0_g7_i3, with Möbius topology, showed highest expression in the leaf, stem and pod, flower tissues respectively. However, novel transcripts ctr29379_c2_g2_i3 and ctr29609_c1_g3_i1, with Bracelet topology, displayed highest expression in pod and stem. Flower exhibited low cyclotide expression profile, with Cter 10 (ctr29609_c1_g1_i2) being the highest expressing gene in flower, clustering with the novel cyclotide ctr29379_c3_g2_i6. Unusual cyclotide gene ctr28495_c0_g5_i3 showed highest expression in leaf and acyclic ‘Cter acyc1′ precursor (ctr29609_c1_g4_i1) showed high expression in flower. The predicted cyclotide gene expression was indeed tissue-specific, and they showed correspondence with untransformed TPM values (Supplementary Table S2). Additionally, Supplementary Table S2 shows that there is little variation between the biological replicates.

Such differential expression of cyclotide genes across different tissues, with varying levels of expression values indicates that cyclotides could be participating in varied plant responses towards biotic and/or abiotic stresses. Further, given the metabolic cost involved in production and maintenance of these peptides, it could be hypothesized that their distribution with the plant is controlled optimally. Whether differential distribution of cyclotides is useful to combat herbivore attack or environmental stresses remains to be studied.

### Proteomic identification of cyclotides

**Figure 6:**
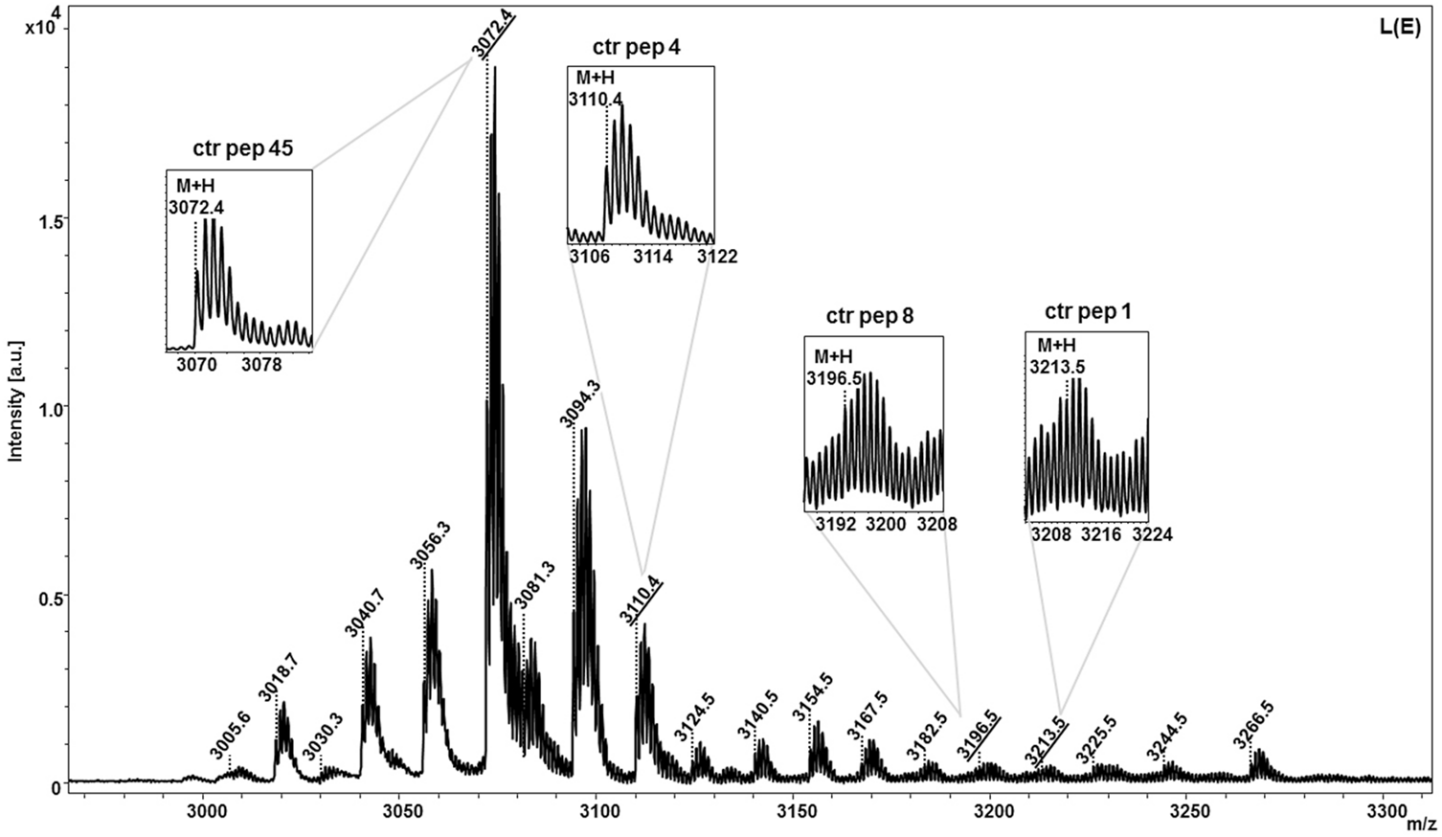
Representative MALDI-TOF mass-spectra of HPLC-purified fraction E from crude extract of leaves of *C. ternatea* (Refer to Supplementary Fig. S5). (Insets) The expanded isotopic multiplets for M + H masses that correspond to predicted cyclotide transcripts (peptide identifiers highlighted) are shown.

We aimed to discover the presence of cyclotides and identify their molecular diversity in *C. ternatea* by using a combined approach of proteomics and transcriptomics analysis. We were interested to determine the presence of cyclotides at the protein level by using primarily MALDI-TOF mass spectrometry method. Mass detected analytical LC ESI–MS profile (ion chromatogram) of the crude extracts are shown in Supplementary Fig. S4. Masses corresponding to cyclotides (> 3,000 Da) were detected in the retention time range 25–55 min. Presence of cyclotides in all the four tissue crude extracts of *C. ternatea* were confirmed by ESI–MS (data not included in this study). Further fractionation of these crude extracts using semi-preparative HPLC was performed and illustrated in Supplementary Fig. S5. The purified fractions were subjected MALDI-TOF mass-spectrometry analysis for detection of > 3,000 Da masses. Cyclotides were distributed in fractions B-E (Supplementary Fig. S5). A representative MALDI-TOF spectra of fraction E from crude extract of leaves of *C. ternatea* is shown in Fig. 6. The MS analysis showed two significant peaks of about 3,071.4 m/z (M + H = 3,072.4) and 3,109.4 m/z (M + H = 3,110.4) and two low intensity peaks of about 3,195.5 m/z (M + H = 3,196.5 m/z) and 3,212.5 m/z (M + H = 3,213.5) corresponding to predicted peptides ‘ctr pep 45’, ‘ctr pep 4’, ‘ctr pep 8’ and ‘ctr pep 1’, respectively. Based on sequence analysis, ctr pep 45 and ctr pep corresponds to cliotide T8 and cliotideT40 respectively, and ctr pep 4 and ctr pep 8 are novel sequences. The expanded isotopic multiplets for M + H masses in all fractions from four tissues that correspond to predicted cyclotide transcripts, are shown in Supplementary Fig. S6. Identification of cyclotides at the proteome level was done by matching the observed monoisotopic masses and calculated molecular weights (Supplementary Table S6). Out of the 51 unique cyclotide sequences obtained at the transcriptomic level, 30 of them were detected at the protein level. Proteomic detection combined with transcriptomic analysis provided us useful information on the diversity of cyclotides produced in *C. ternatea.* The combined -omics approach resulted in more than half of the transcript sequences being predicted at protein level. Nevertheless, to uncover complete cyclotide diversity,novel structural templates and possible PTMs in a single plant specimen, a combinatorial procedure of transcriptome analysis, LC–MS deconvolution and tandem MS sequencing and transcriptome analysis is essential^75–77^.

### Asparaginyl endopeptidase (AEP)

**Figure 7:**
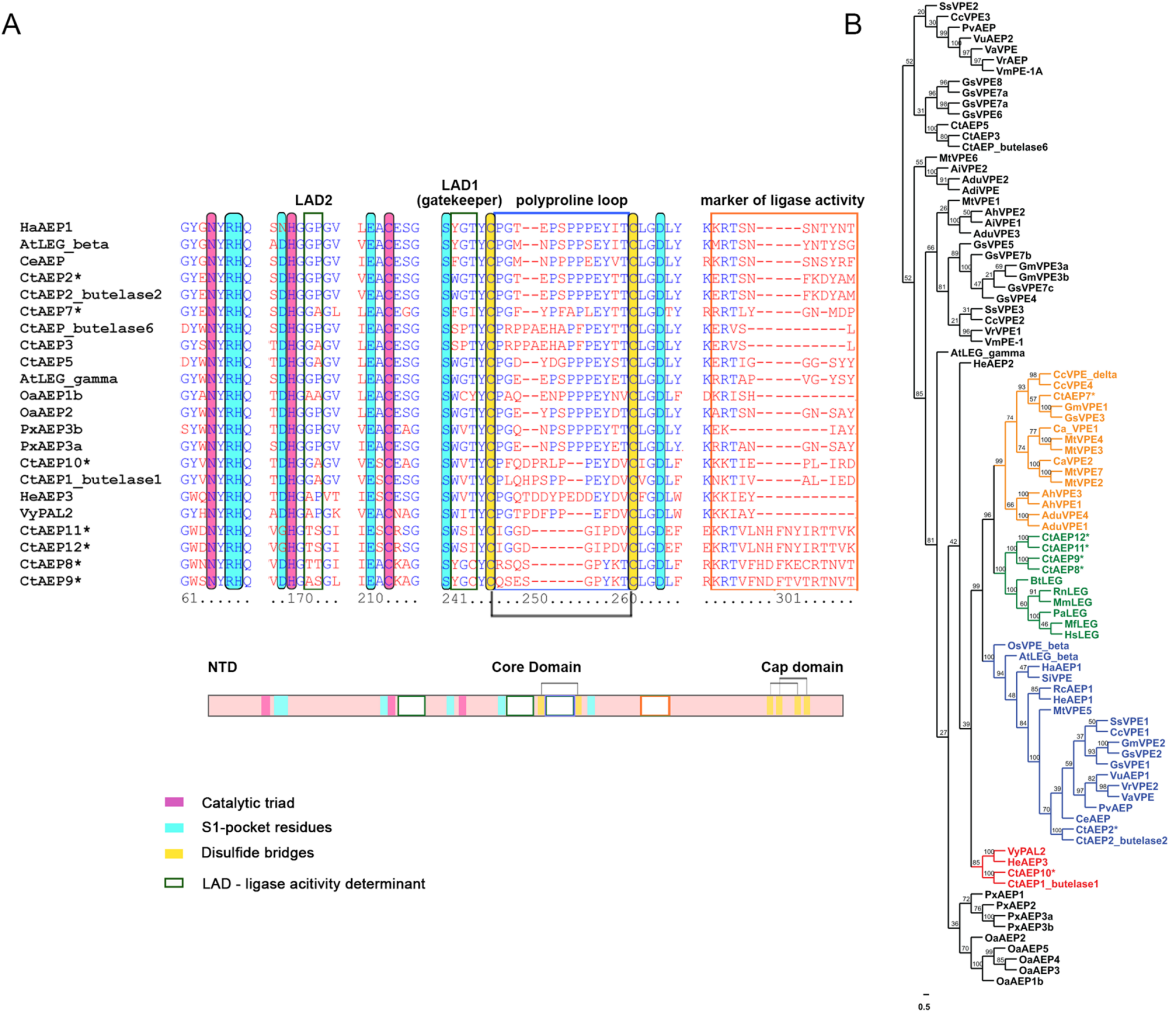
Asparaginyl endopeptidase sequences (AEPs) from *C. ternatea* transcriptome (**A**) Comparative sequence alignment of 7 nearly full-length AEPs from the current de novo transcriptome assembly and AEPs/legumains from *Oldenlandia affinis*, *Canavalia ensiformis*, *Petunia x hybrida*, *Arabidopsis thaliana*, *Helianthus annuus*, *Viola yedoensis* and *Clitoria ternatea* (butelases). The catalytic triad “Asn-His-Cys”is highlighted in pink. The conserved cysteines forming disulphide bridges are highlighted in yellow. S1-pocket residues is highlighted in blue. Ligase-activity determinant residues (LAD1 and LAD2), poly-proline loop (PPL) and marker of ligase activity (MAL) are boxed in green. (**B**) Phylogenetic tree of *Clitoria ternatea* AEP proteins with known mammalian legumains and plant AEP/VPEs. The tree contains 91 sequences in total and was constructed using Maximum likelihood (ML) method using MEGA version X software^64^. Bootstrap values at roots are indicated as percentages. Distinct clades are highlighted in different colour fonts, and outliers are coloured in black font. Novel cyclotide sequences are highlighted with an asterisk ‘*’.

We identified 14 AEP sequences in the transcriptome assembly. Seven out of the 14 hits were complete transcripts composed of the ER-signal and the mature active domain (Supplementary Table S7). Till date, five AEP sequences from *C. ternatea* (CtAEP1/butelase-1, CtAEP2/butelase-2, CtAEP3, CtAEP5 and butelase-6) have been well characterized^21,22^. AEP multiple sequence alignment and phylogeny was performed using AEP transcripts obtained in our transcriptome, Fabaceae and other plant AEP/VPE sequences, and mammalian legumains (Fig. 7). All the 7 hits contain the catalytic His and Cys residues, as highlighted in Fig. 7A. CtAEP10 is a partial sequence, lacking the N-terminal ER signal peptide and an incomplete 3′ end. Only one transcript (ctr29014_c2_g1_i2), bares 100% sequence identity with the known CtAEP2 sequence.

Recent studies indicate that two sites play an important role in differentiating a protease-type AEP and a ligase-type AEP. One is a “gatekeeper” position (GK) and the other is a “marker for ligase activity” (MLA) motif^29^. GK residue position (Cys247 in OaAEP1b), was found to be responsible for the ligation efficiency of OaAEP1b. This cysteine residue in some instances is replaced by a valine residue in the case of ligase-type AEPs (PxAEP3b) and glycine residue in case of protease-type AEP (PxAEP3a). The ligase-type PxAEP3b was also shown to present a truncated MLA region and the protease version, PxAEP3a, had a longer and hydrophilic MLA region^29^. On the contrary, a recent study determined that GK and MLA sites alone are not responsible for ascribing ligase activity to AEPs. Using X-ray crystallography and site-directed mutagenesis, it was suggested that as the MLA region is distantly located from the catalytic core and it might not play a crucial role in activity^78^. They identified putative ligase-activity determinants, LAD1 (contains GK site) and LAD2 as the key sites for activity determination, apart from the remotely located MLA region. Specifically, they propose that the first position in LAD1 should be a bulky aromatic amino acid and the second position should be hydrophobic such as Val/Ile/Cys/Ala but not Gly. Figure 7A shows that all the seven transcripts have aromatic residues such as phenylalanine, tyrosine and tryptophan at the first position in LAD1. However, the second position is a glycine or a serine residue at the GK site (position 243 in the alignment) in CtAEP2, CtAEP7, CtAEP8, CtAEP9, CtAEP11 and CtAEP12. This suggests that these could be the protease-type AEPs. A single sequence, CtAEP10, displays a valine residue at the GK site, implying this sequence to be a ligase-type AEP. The investigators also proposed that a small residue such as Gly or Ala be present at the first position of LAD2, which is the case in our CtAEP10 sequence (Fig. 7A).

Figure 7B reveals the phylogenetic clustering of CtAEP8 and CtAEP9, CtAEP11 and CtAEP12 transcripts with mammalian AEP sequences (green clade). Interestingly, CtAEP7 clusters with pigeon pea’s VPE4 and VPEδ (yellow clade). As these sequences also lack the GK Val/Ile/Cys/Ala at position 243 in the alignment (Fig. 7A), it is likely that these represent the protease-type AEP (Fig. 7B). Only two transcripts in the transcriptome are nested with known ligase-type AEP sequences clade. –CtAEP10 sequence co-clusters with CtAEP1/butelase1 and OaAEP1b (red clade), and CtAEP2 sequence co-clusters with CtAEP2/butelase2 (blue clade), as seen in Fig. 7B. TPM values of the AEP transcripts expressed across different tissues were calculated and compared with cyclotide transcripts (Supplementary Fig. S7). Most of the transcripts showed high expression in the flower based on the mean TPM value (Supplementary Table S2). However, CtAEP2 (butelase 2), CtAEP7 and CtAEP10, showed high expression in terms of TPM values in leaf, pod and stem tissues, respectively. Clustering of CtAEP10 (predicted ligase-type AEP) with several cyclotide transcripts, which get expressed highly in stem, provides evidence on the involvement of AEP in cyclotide cyclization.

### Protein disulphide isomerase (PDI)

PDI is a soluble protein in the ER and is involved in catalysing disulphide bond formation of substrate proteins. Furthermore, it is known to be involved in disulphide-bond formation of several seed storage proteins^79–81^. In butterfly pea, cyclotide gene is embedded within the albumin 1b gene, hence it is reasonable to assume the involvement of PDI for cyclotide folding. A variety of plant PDIs and PDI-like proteins have been identified through independent genomic approaches, resulting in a total of about 22 members in *A. thaliana*, 12 members from *Oryza sativa*, 32 from *Brassica rapa ssp. Pekinensis*, and 22 in *Zea mays* and 9 from *Triticum aestivum L*^82–85^*.* A novel PDI from the Rubiaceae plant *O. affinis* (OaPDI) was reported to be involved in the in vitro folding of knotted circular proteins^36^. In-depth analysis of PDI sequences in terrestrial plants have led to several distinct types of nomenclature^82,83,86,87^. The former study named *Arabidopsis* PDI isoforms as PDI1.1 to 1.6, PDI2.1 to 2.3 and PDI5.1 to 5.4^82^. More recent studies, specifically on soybean PDIs, a letter for various PDI classes and consecutive numbers for each isoform was used^88,89^. For example, classical PDIs were named PDI-L, and each subclass was distinctly numbered. Due to the ambiguity in nomenclature, we have not followed any of the above methods and instead named our *C. ternatea* PDI sequences numerically. We have found 34 PDI and PDI-like hits, of which 15 are nearly full-length sequences (exhibiting two TRX motifs) and 19 are partial/incomplete sequences. The list of full-length PDI sequences from *C. ternatea* are presented in Supplementary Table S8. Structural characteristics such as the active site residues, C-terminal signal sequence and the domain composition is also presented in Supplementary Table S8. The domain architecture adopted was found to be predominantly the classical type i.e. **a**–**b**–**b′**–**a′** arrangement of successive thioredoxin domains.

**Figure 8:**
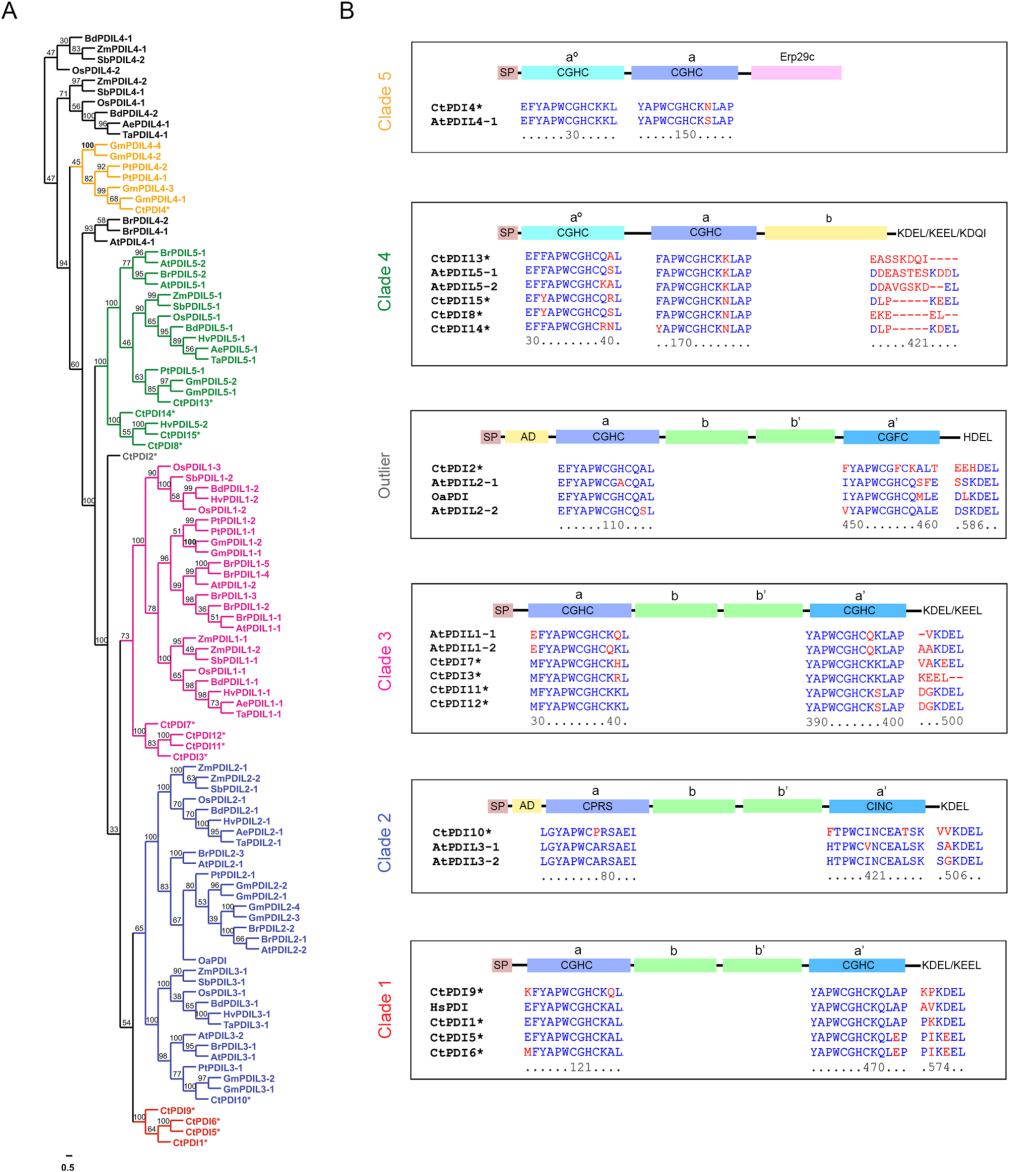
Protein disulphide isomerase sequences (PDIs) from *C. ternatea* transcriptome (**A**) Phylogenetic tree of *Clitoria ternatea* PDI hits with other plant PDIs. Distinct clades are highlighted in different colour fonts, and outliers are coloured in black font. The tree contains 104 sequences in total and was constructed using Neighbor-joining (NJ) method using MEGA version X software^64^. Bootstrap values at roots are indicated as percentages. (**B**) Domain architecture of CtPDI sequences aligned with canonical PDI sequences and categorized into different clades are displayed. The catalytic thioredoxin-like tetrapeptide “CXXC” and C-terminal ER-retention signal tetrapeptide of the 15 PDI sequences obtained from the de novo transcriptome assembly of *C. ternatea* along are reported. The putative signal peptides (SP), the a and b type domains, the N-terminal acidic domain (AD) and the Erp29c domain are highlighted.

Figure 8A highlights the phylogenetic construction of known plant PDIs with CtPDI sequences from the current transcriptome. Four sequences, CtPDI3, CtPDI7, CtPDI11 and CtPDI12, are nested within PDIL1 subfamily, highlighted as clade 3 (pink). Sequences CtPDI14, CtPDI15, CtPDI13 and CtPDI8 are nested within PDIL5 subfamily, highlighted as clade 4 (green). CtPDI4 sequence is nested within PDIL4 subfamily, highlighted as clade 5 (yellow). CtPDI10 is a single sequence that is nested within PDIL3 and PDIL2 subfamilies, (clade 2, blue). Four sequences, CtPDI1, CtPDI5, CtPDI6 and CtPDI9, form a separate cluster (clade 1, red). CtPDI2 sequence is an outlier and does not cluster with any of the above PDIL subfamilies, suggesting it could be a species-specific foldase. Figure 8B shows the domain structure of 15 PDI sequences extracted from the current *C. ternatea* transcriptome. Domain boundaries were identified by sequence alignment to *Arabidopsis* PDI sequences and human PDI sequence. It also highlights the sequence conservation of the redox active sites (**a** or **a′** or **a°**) and the ER-retention signal. The two thioredoxin active site motifs (CXXC) in **a** and **a′** (or **a°**) domains are fully conserved. **a** domain in fourteen of the CtPDI sequences has the motif CGHC, while CtPDI10 sequence has motif CPRS. **a′** (or **a°**) domain in 13 CtPDI sequences has the motif CGHC, while two CtPDI sequences have CHFC and CINC motif (CtPDI2 and CtPDI10 respectively). Thirteen out of the 15 full length sequences show the C-terminal ER-retention signal ‘K[D/E]EL’ motif. CtPDI13 has a unique ‘KDQI’ motif at the C-terminal end, suggesting that it may get secreted from the endoplasmic reticulum, instead of being retained in the ER (Fig. 8B). CtPDI2 bears reasonable sequence similarity with AtPDIL2-1, AtPDIL2-2 and OaPDI sequence^36,82^; however, it lacks the N-terminal acidic domain that is characteristic of PDIL2 subfamily (Fig. 8B) and possesses ‘HDEL’ motif at the C-terminus. A distinguishing characteristic of OaPDI is that the two active sites contain Gln residue (instead of a Lys residue) as commonly seen in many PDI sequences in humans and in Arabidopsis^36^. Similarly, CtPDI2 sequence also seems to be different from several known PDIs. Firstly, it has a CHFCXA motif in the active site of a’ domain and secondly, it bears a Gln residue in the first active site (**a** domain) and a Lys residue in the second active site (**a′** domain) at the X-position, which is an uncommon combination in canonical PDI sequences. A recent study on cone snail PDI family suggest that two distinct isoforms exist, canonical PDIs and conotoxin-specific PDIs (CsPDI)^90^. Supplementary Fig. S8 shows a multiple sequence alignment of PDIs from *C. ternatea* transcriptome, *Conus geographus* cone-specific PDI and *Conus geographus* canonical PDI. There is a high degree of sequence conservation between the butterfly pea PDI sequences and the cone snail PDIs, especially in the two thioredoxin domains (**a** and **a′**). However, two CtPDIs show significant differences in the + 2 position of the C-terminus and in both the CGHC active sites. CtPDI9 has CGHCKQ in both the catalytic domains. Such apparent differences in the active site motifs were observed in conotoxin-specific PDI (*C. geographus* csPDI) sequences^90^. This suggests that protein disulphide isomerases could have diversified in *C. ternatea* and in a correlated manner with the diversification of cyclotide sequences. Overall, Fig. 8 highlights the phylogenetic and domain analyses of CtPDI gene family. The amino acid conservation and co-clustering of PDI genes demonstrates that the PDI gene family is divergent in plants. Specifically, the phylogenetic analyses highlight that the PDIs from *C. ternatea* cluster independently to form phylogenetically distinct groups.

Hierarchical clustering analysis of the PDI expression levels, shows that out of the 15 sequences, 11 are highly expressed in the flower tissue (Supplementary Table S8). The expression level (TPM) of the fifteen genes of PDI family from *C. ternatea* are plotted along with the cyclotide transcripts across the four tissues (Supplementary Fig. S9). Consistent with the hypothesis of the role of PDI in disulphide isomerase activity of cyclotides, we observed high expression levels of few PDI genes correlated with cyclotide expression in each tissue. Especially CtPDI2, a predicted species-specific outlier in phylogeny, is up-regulated in the stem and clusters well with several cyclotide sequences. CtPDI4 and CtPDI10 also clusters with five cyclotide sequences (Cter 19, Cter 31, cliotide T8, cliotide T32 and cliotide T13) in the stem tissues. CtPDI13 shows high expression levels in pod and stem tissues, along with novel cyclotide transcripts ctr28841_c1_g3_i1 and ctr28192_c2_g6_i1.

### Endoplasmic reticulum oxidoreductin-1 (ERO1)

**Figure 9:**
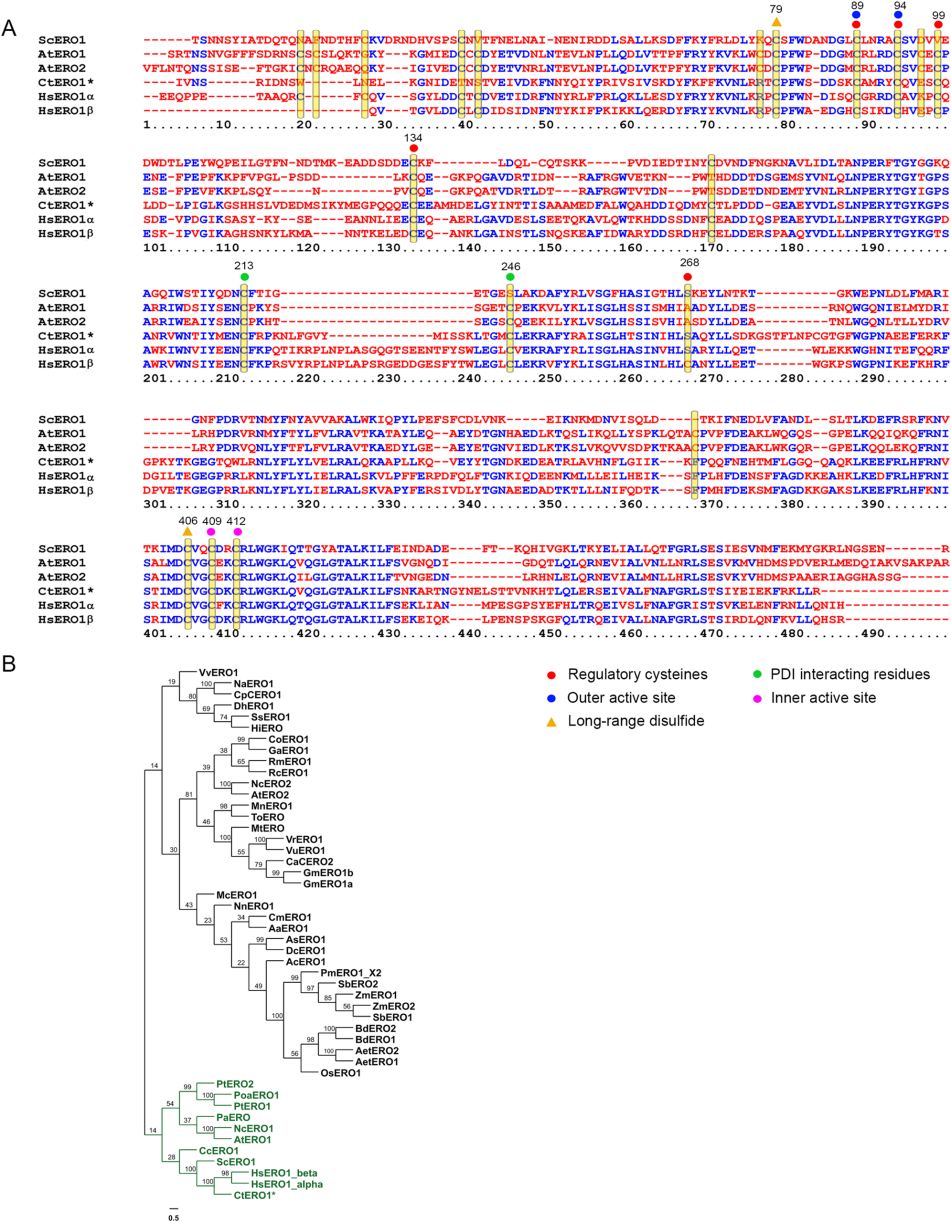
ER oxidoreductin-1 sequence from *C. ternatea* transcriptome (**A**) Comparative sequence alignment of the sole ERO1 protein sequence (highlighted with an asterisk “*”) obtained from the *C. ternatea* transcriptome with yeast (ScERO1), human (HsERO1α and HsERO1β) and *Arabidopsis* (AtERO1 and AtERO2) ER oxidoreductin-1 sequences. Cysteines are highlighted in yellow bars, regulatory cysteines are highlighted with a red circle, long-range disulphide is highlighted with an orange triangle, PDI-interacting partners are highlighted with a green circle, outer active site is highlighted with a blue circle, inner active site is highlighted with a pink circle and non-catalytic cysteines are not marked with a circle or triangle. Numbering of residues is based on the alignment. (**B**) Phylogenetic tree of *Clitoria ternatea* ERO1 hits with yeast (ScERO1), human (HsERO1α and HsERO1β) and plant ERO1 homologs. Distinct clades are highlighted in different colour fonts, and outliers are coloured in black font. The tree contains 48 sequences in total and was constructed using Neighbor-joining (NJ) method using MEGA version X software^64^. Bootstrap values at roots are indicated as percentages.

ERO1 catalyzes the formation of disulphide bonds in its substrate PDI in conjunction with FAD cofactor^91^. ERO1 proteins are highly conserved across kingdoms of life. Disulphide bond formation and proper folding of cyclotides in plants takes place in the ER, where several cyclotides are expressed and properly folded. Proper folding of disulphide-rich peptides requires the concerted efforts of multiple enzymes, as this is critical to their biological activity in the cellular environment^46,47,92^. Here, we have identified a single ERO1 protein sequence (474 amino acid) in the *C. ternatea* transcriptome, which has not been attempted before. Sequence of CtERO1 transcript (ctr203_c0_g1_i1) is described in Fig. 9A, with all the active site residues highlighted on the multiple sequence alignment. Figure 9A highlights the high amino acid sequence homology of CtERO1 to human ERO1α 48% sequence identity) and ERO1β (~ 50% sequence identity), as opposed to *A. thaliana* ERO sequences (< 39% sequence identity). This is substantiated again in the phylogeny of homologs from plants, yeast and human, where CtERO1 clusters with human and yeast ERO1 homologs with high nodal support (green clade) (Fig. 9B). The putative outer active sites (Cys89-Cys94) and inner active sites (Cys409–Cys412) are fully conserved in ERO1s across yeast, plant and human ERO sequence. The putative regulatory cysteines Cys89, Cys94, Cys99 and Cys134 are fully conserved. However, position 268 in CtERO1 sequence is occupied by serine instead of a cysteine residue, characteristic of HsERO1β. The putative long-range disulphide bridges (Cys79–Cys406) and PDI interacting partners (Cys213 and Cys246) are also conserved across the yeast, plant and human ERO sequences (Fig. 9A). Similar to the expression levels of several CtPDI genes, CtERO1 is also upregulated in the flower tissue and substantiates the fact that ERO1 might be participating in PDI re-oxidation and activation (Supplementary Fig. S9).

### Peptidylprolyl *cis-trans* isomerase (PPIase)/cyclophilin

**Figure 10:**
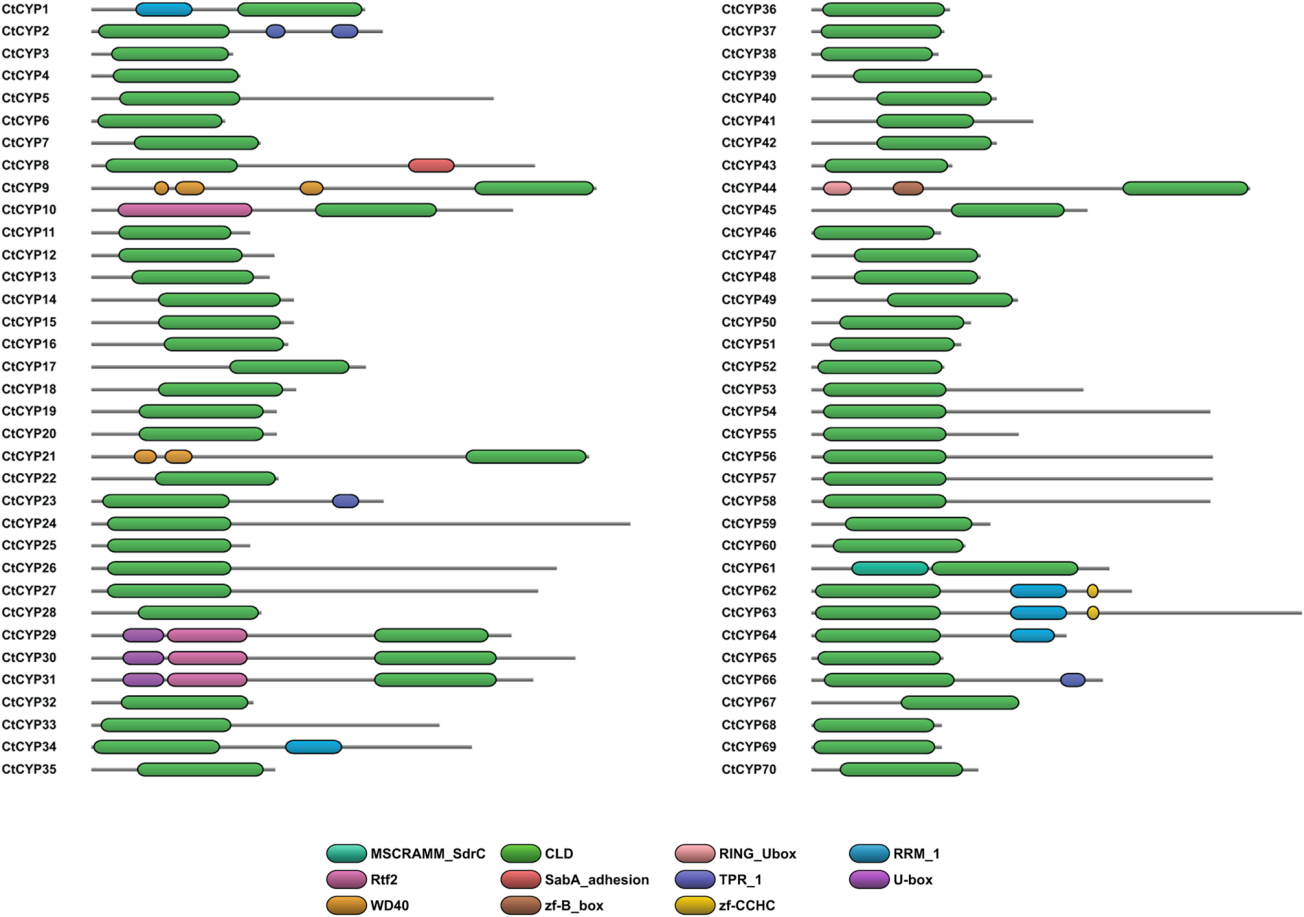
Schematic representation of single-domain and multi-domain peptidylprolyl *cis-trans* isomerase/cyclophilins in *C. ternatea* (70 sequences). The domain arrangement was created using DoMosaics tool^95^. The cyclophilin-like domain (CLD) is represented in green. *MSCRAMM_SdrC* microbial surface components recognizing adhesive matrix molecules-SdrC adhesion domain, *RING_Ubox* RING U-box domain, *RRM_1* RNA recognition motif 1, *Rtf2* Replication termination factor 2 RING-finger domain, *SabA_adhesion* Sialic acid binding adhesion domain, *TPR_1* Tetratricopeptide-like repeats, *U-box* U-box domain, *WD40* tryptophan-aspartate (WD) repeats, *zf-B_box* B-box zinc finger domain, *zf-CCHC* Zinc-finger motif.

To identify all members of the CYP gene family in *C. ternatea*, a BLASTp sequence search was performed against the transcriptome using *G. max* CYP1 sequence (UniProt entry Q8W171). This search identified a total of 70 unique CYP genes and named CtCYP1 to CtCYP70 (Supplementary Table S9). All the identified proteins were analysed for presence of CLD domain and additional domains using CDART, SMART and DoMosaics tool^93–95^. Of the 70 CYP genes, 53 encoded a protein with a single cyclophilin-like domain (CLD). 17 of the 70 genes contain cyclophilin-like domain (CLD) and additional domains, hence classified as multi-domain CYP genes (Supplementary Table S9). Figure 10 highlights a schematic representation of domain architectures of all CtCYPs sequences. CtCYP1, CtCYP34, CtCYP62, CtCYP63 and CtCYP64 comprises of an RNA recognition motif (RRM). CtCYP62 and CtCYP63 also contain an additional Zinc-finger (zf-CCHC) motif at the C-terminus end. CtCYP2, CtCYP23, CtCYP66 includes Tetratricopeptide-like repeats (TPR) at the C-terminus. CtCYP8 contains a Sialic acid binding adhesion domain (SabA adhesion) at the C-terminus end, which is not commonly known to exist in cyclophilin sequences. CtCYP9 and CtCYP21 comprise two/three tryptophan-aspartate (WD) repeats at the N-terminus. CtCYP10 includes a large Replication termination factor 2 (Rtf2) RING-finger domain at the N-terminus. CtCYP29, CtCYP30 and CtCYP31 contain Rtf2 and U-box domains at the N-terminus end of the protein. CtCYP44 includes a B-box zinc finger and RING U-box domains at the N-terminus. Lastly, CtCYP61 comprises of MSCRAMM (microbial surface components recognizing adhesive matrix molecules)-SdrC adhesion domains at the N-terminus, which is a novel arrangement seen in cyclophilin proteins. Subcellular localization was predicted using five different algorithms—DeepLoc1.0, YLoc, SherLoc2, CELLO v2.5 and MultiLoc2^96–100^. The results show that several CYPs are predicted to localise in the ER and there is a consensus among the different algorithms (Supplementary Table S9). Of the 70 CtCYPs, 19 have been predicted by at least one predictor to be targeted to the ER, therefore, likely to be involved in cyclotide biosynthesis. Hierarchical clustering analysis of the CYP expression levels show that these 19 CtCYPs are distributed among all the four tissues, clustering with differentially expressed cyclotide genes (Supplementary Fig. S10). TPM values for majority of PPIases in *C. ternatea* indicate high expression in the stem and flower tissue, and these predominantly are single domain CYP genes (Supplementary Table S9).

## Conclusions

**Figure 11:**
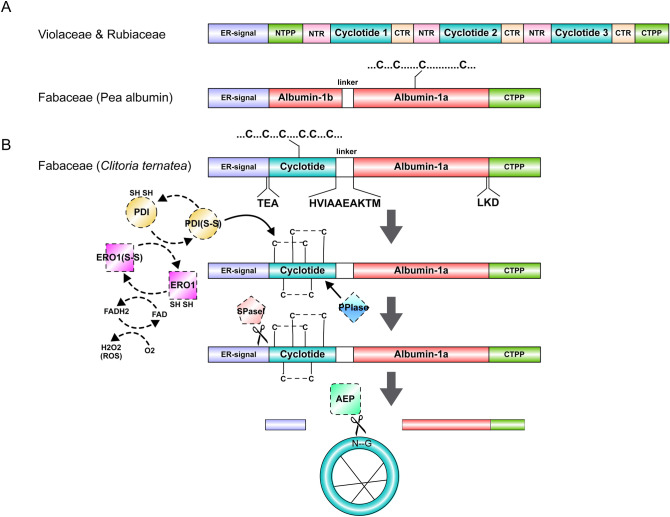
(**A**) Schematic view of the genetic arrangements of Violaceae and Rubiaceae cyclotide precursors and Fabaceae albumin precursor. (**B**) Overview of proposed biosynthetic pathway of cyclotides in *Clitoria ternatea* (Fabaceae). The concept figure was generated using Illustrator for Biological Sequences (IBS)^101^. *NTPP* N-terminal propeptide, *NTR* N-terminal repeat, *CTPP* C-terminal propeptide, *CTR* C-terminal repeat, *PDI* protein disulphide isomerase, *ERO1* ER oxidoreductin-1, *SPase1* signal peptidase-1, *PPIase* peptidylprolyl *cis-trans* isomerase, *AEP* asparaginyl endopeptidase.

In the present study, we describe the de novo assembly and annotation of the *C. ternatea* transcriptome. We identified a total of 71 cyclotide precursor sequences and 26 of them describe new cyclotide sequences. In-depth analysis of the ER-signal regions and cyclotide mature domains, highlight the sequence diversity at the transcript and proteome level. We have also highlighted tissue-specific gene expression of cyclotides through hierarchical clustering method. The results suggest that cyclotide expression occurs in a tissue specific manner and the levels of expression vary greatly. Figure 11 highlights the cyclotide gene architecture and the possible biosynthetic pathway, displaying the concerted effect of various enzymes in the proper folding and processing of cyclotides. Specifically, the cyclization enzyme and the oxidative folding enzymes have been explored in this study. Macrocyclizing AEPs are essential for producing cyclic peptides that impart ultra-stability to already stable molecules. Such AEPs have a wide potential in peptide engineering and synthetic biology. However, recombinant expression of macrocyclizing AEPs is challenging. The current model of cyclotide production suggests formation of disulphide bonds before cyclization step, within the ER. For this PDIs are essential and this study is the first to provide transcriptomic evidence for diverse PDI isoforms in *C. ternatea*. Furthermore, re-oxidization of reduced PDIs has not been addressed in plants producing cyclotides. However, the current study permits identification of candidate enzymes such as the ER oxidoreductin. A single isoform of ERO1 could be retrieved from *C. ternatea* transcriptome. The main difference between the two structural families of cyclotides is a *cis*-proline in loop 5, observed only in the Möbius family. To catalyze this crucial reaction, PPIases are essential and no sequence search efforts in a cyclotide producing plants have been attempted earlier. This study is the first to report 70 isoforms of PPIases in *C. ternatea*. In the absence of a complete genome sequence of *C. ternatea*, comprehensive transcriptomics analyses reported in this study might be useful for further developing applications of this medicinally relevant plant.

## Supplementary information

Supplementary Data 1

Supplementary Information

Supplementary Data 2

## Data Availability

The raw reads generated for this study have been deposited in BioProject Accession: PRJNA573557 (https://www.ncbi.nlm.nih.gov). The nucleotide and protein precursors generated and/or analysed during the current study are available in GenBank under the accession numbers for *C. ternatea* (a) cyclotide precursor sequences: MT468661-MT468731, (b) asparaginyl endopeptidase sequences: MT468732- MT468738, (c) protein disulphide isomerase sequences: MT468739- MT468753, (d) endoplasmic reticulum oxidoreductin-1 sequence: MT468754 and (e) peptidyl prolyl *cis-trans* isomerase sequences: MT468755- MT468824, respectively.
