# Supplementary material for: Transcriptomic profiling of the medicinal plant Clitoria ternatea: identification of potential genes in cyclotide biosynthesis
Source: Sci Rep. 2020 Jul 29;10:12658. doi: 10.1038/s41598-020-69452-7 (PMC7391643; doi:10.1038/s41598-020-69452-7)
Supplement: Supplementary file 1 — Supplementary Data 1 [file 41598_2020_69452_MOESM1_ESM.docx]

**Supplementary Data 1:**

**A. Cyclotide precursor sequences from *Clitoria ternatea***

>ctr29609_c1_g2_i1

GRKPVTNIFDNLNQKMAYLRLFTLAAVIFFFAASVEKTKADLICSSTCLHTPCKASVCYCKNAVCYKNHVIAATSNSVNDYYLLCQSHEDCIIKESGNFCAPFLEHDVYGWCFYAESEGYLLKDFLNIPEDIVKKPMQITS

>ctr29609_c1_g2_i2

MFAVEKTKADLQCAETC

VHSPCIGPCYCKHGVICYKNHVIAAAAKSVNDYYLLCQSHEDCIIKGSGNFCAP

>ctr29609_c1_g2_i3

GRKPVTNIFDNLNQKMAYLRLFTLAAVIFFFAASVEKTKADLQCAETC

VHSPCIGPCYCKHGVICYKNHVIAAAAKSVNDYYLLCQSHEDCIIKGSGNFCAP

>ctr29379_c3_g2_i6

MAYLRLVPLLVLFFFAASVNKTEAGALCDERC

TYVPCISAARGCSCNIHRVCSMNHVIAATSKSIDEHHLLCQSHEDCITKGTGNFCAPFLEHDVPYGWCFRA

EAEGYLLKDFLKIPKDILKKPIEITN

>ctr29379_c3_g2_i8

MAYLRLVPLLVLFFFAASVNKTEAGGTACGESC

IYLPCISGVFEGCSCQNKACYKNHVIAATSKSIDEHHLLCQSHEDCIIKGTGNFCA

>ctr29609_c1_g4_i1

MTYVRLAPLIVIFLLLPSVKNTEAVDGFCLETC

VILPCFSSVAGCYCHGSTCMRGTTIASMAKTIDEHRNLCQTHEDCITKKSGNFCARFPNHNINYGWCFNA

ESEGFLLKDHLKMLTAN

>ctr28841_c1_g2_i1

MQSKVIAYFHQIMASLRIAPFAVFLFLAASVMFAVEKTQAGVIPCGESC

VFIPCISTVIGCSCKNKVCYRNHVIAAEAKTM

>ctr28841_c2_g3_i1

SDLAYYHQIMASLRIAPFAVFLFLAASVIFAVEKTQACVIPCGESR

VFIPCITGAIGCSCKSKVCYRNHVIAAEAKT

>ctr28841_c1_g1_i3

MANVKLATLLVNFLLVTSVMFVVKKTEAKIPCGESC

VWIPCFTSAFGCYCQSKVCYHSTQIASTAKTMNDHHLLCQSHEDCIIKKSGNFCAHFPNHDVHYGWCFRA

>ctr28841_c1_g4_i1

ESCVWIPCLTGYFGCYCQSKVCYHNSHIASTAKTMNDHHLLCQSHEDCVTKGSGNFCAHFPDHDIHYGWCFRA

ESEGFPLKDFMKMPK

>ctr29746_c1_g3_i3

ALPISTIVGCSCKSNVCYSNHVIAATAKSLDEHRLLCQSHEDCFVKGTGNFCAHFPEGDVAYGWCFHA

ESEGYLLKDFLKMPKDT

>ctr29746_c1_g3_i2

PITNISNQLSQIMAKLVPLIVIFLVATSVDMTKASIPCGESC

VYIPCLTTIVGCSCKSNVCYSNHVIAATAKSLDEHRLLCQSHEDCFVKGTGNFCAHFPEGDVAYGWCFHA

ESEGYLLKDFLKMPKDT

>ctr29746_c1_g3_i1

SPINSYRGHKELLKEKPITNIGNQLSQIMAKLVPLIVIFLVAASVDMTKASIPCGESC

VYIPCLTTIVGCSCKSNVCYSNHVIAATAKSLDEHRLLCQSHEDCIIKGSGNFCA

>ctr29746_c1_g3_i4

SPINSYRGHKELLKEKPITNIGNQLSQIMAKLVPLIVIFLVAASVDMTKASIPCGESC

VYIPCLTTIVGCSCKSNVCYSNHVIAATAKSLDEHRLLCQSHEDCFTKGIGNFCAHFPEGDVAYGWCFRA

ESEGYLLKDFLKMPKDT

>ctr28841_c2_g4_i2

MAYLRLAALAVIFLLATTVKKTVAARIPCGESC

VWIPCTITALVGCACHEK

>ctr29609_c1_g3_i1

MQEGSNAEHQLNQTMASFRIAPFALFCFLAASVMFAVEKTEAGIPCGESC

VFLPCFIIPGCSCKDKVCYLNHVIASTAKTMNDHHLLCQSHEDCITKRTGNFCANFPNQDIKYGWCFRA

ESEGFMLKDHLKMSITN

>ctr29379_c2_g2_i1

ITESGSPCGESC

VFIPCISTVIGCSCKNKVCYRNHIIAAEAKTMDEHILLCQSHEDCIAKGTGNFCAPFPDQDIKYGWCFRA

ESEGFMLKDHLKMSITN

>ctr29379_c2_g2_i3

ITESGSPCGESC

VFIPCISTVIGCSCKNKVCYRNHIIAAEAKTMDEHILLCQSHEDCIAKGTGNFCAPFPDQDIKYGWCFRA

ESEGFLLKDHLKMSITN

>ctr29609_c1_g1_i2

MAFLRFAPLAVLFFLATSVMFSMKEAEASYIPCGESC

VYIPCTVTALLGCSCSNKVCYKNHVIASEAKTMDDHALLCQSHEDCIIKGTGNFCAPFADKDIKYGWCFRA

ESEGFFLKDHLKMSIAN

>ctr29609_c1_g1_i6

MAFLRFAPLAVLFFLATSVMFSMKEAEASYIPCGESC

VYIPCTVTALLGCSCSNKVCYKNHVIASEAKTMDDHALLCQSHEDCIIKGTGNFCAPFADKDIKYGWCFRA

VSEGFMLKDHLKMSMTN

>ctr29609_c0_g1_i1

MASVRFGPLVVLCFLATSVMLSVKEAEAGIPCGESC

VFIPCTITALLGCSCKDKVCYKNHVIASEAKTMDDHHLLCQSHEDCFRTGPGNFCAPFLN

>ctr29746_c1_g4_i1

AEPLTNISNQIMGTIARYYAHVVLFLVATSVIFTVKKTEAGVPCAESC

VWIPCTVTALLGCSCKDKVCYLNHVIASEAKTMDEHHLLCQSHEDCYKKGSGNFCAPFLNHDVKYGWCFRA

ESEGYLLKDFLKMQP

>ctr29746_c1_g2_i5

VMFAVKETQAGVPCGESC

VYIPCTVTALLGCSCKNKVCYRNHVIAAEASTVDDHHLLCQSHEDCFKKGTGNFCAPFLEHDVKYRS

>ctr29746_c1_g2_i6

LNQNMAFARLAVIFFLAASVMFTVKETEAGIPCGESC

VYIPCTVTALLGCSCKNKVCYRNHVIAAEASTVDDHHLLCQSHEDCFKKGTGNFCAPFLEHDVKYRS

>ctr29379_c3_g1_i2

LYQLNQNMAFARLAVIFFLAASVMFTVKETEAGIPCGESC

VYIPCTVTALLGCSCKNKVCFRNHVIA

>ctr29746_c1_g2_i1

LNQNMAFARLAVIFFLAASVMFTVKETEAGIPCGESC

VYIPCTVTALLGCSCKNKVCYRNHVIAAEASTVDDHHLLCQSHEDCFKKGSGNFCAPFLGHDVKYGWCFQ

>ctr29746_c1_g2_i3

VMFAVKETQAGVPCGESC

VYIPCTVTALLGCSCKNKVCYRNHVIAAEASTVDDHHLLCQSHEDCFKKGSGNFCAPFLGHDVKYGWCFQ

>ctr28192_c2_g1_i2

MAYPRLAVIFLLAASVMFAVKDAEAGIPCGESC

VFIPCTITALLGCSCKSKVCYKNHVIAAEASTVDDHHLLCQSHEDCFKKGSGNFCAPFLGHDVKYGWCFHA

ESEGFLLKDFLKMSSDNN

>ctr28192_c2_g1_i7

MAYPRLAVIFLLAASVMFAVKDAEAGIPCGESC

VFIPCTITALLGCSCKSKVCYKNHVIAAEASTVDDHHLLCQSHEDCFKKGSGNFCAPFLEQDVKYGWCFRA

ESEGFLLKDFFKM

>ctr29746_c1_g2_i2

MFAVKATEVGIPCVESC

VFIPCTVTALLGCSCKDKVCYKNHVIAAEANTVNDHHLLCQSHEDCFKKGTGNFCAPFLKHDVKYGWCFRA

>ctr29746_c1_g2_i4

LNQNMAFARLAVIFFLAASVMFTVKETEAGIPCGESC

VFIPCTVTALLGCSCKDKVCYKNHVIAAEANTVNDHHLLCQSHEDCFKKGTGNFCAPFLKHDVKYGWCFRA

>ctr29379_c3_g2_i3

IAYLDQLNQKMAFARLAVIFFLAASVMFAMKETEAGIPCGESC

VFIPCTVTALLGCSCKDKVCYKNHVIAAEANTVDDHHLLCQSHEDCFRKGSGNFCAPFLNYEVPYGWCFRA

ESEGYLLKDFLNVSKDILKKPIEITN

>ctr29379_c3_g2_i12

ARLAVIFFLAASVMFALKETEGGIPCGESC

VFISCTVTALLGCSCKDKVCYKNHVIAAEANTVDDHHLLCQSHEDCFRKGSGNFCAPFLNYEVPYGWCFRA

ESEGFLLKDFLKTPADTLKMPNAI

>ctr29379_c3_g2_i9

IAYLDQLNQKMAFARLAVIFFLAASVMFAMKETEAGIPCGESC

VFIPCTVTALLGCSCKDKVCYKNHVIAAEANTVDDHHLLCQSHEDCFKKGAGNFCAPFLEHDVKYGWCFRA

ESEGFLLKDFLK

>ctr29379_c3_g2_i10

ARLAVIFFLAASVMFALKETEGGIPCGESC

VFISCTVTALLGCSCKDKVCYKNHVIAAEANTVDDHHLLCQSHEDCFKKGAGNFCAPFLEHDVKYGWCFRA

ESEGFLLKDFLKTPADTLKMPNAITN

>ctr29379_c3_g2_i4

TTIPCFHQLNQNMAFARLAVIFFLAASVMFAMKETEAGIPCGESC

VFIPCTVTALLGCSCKDKVCYKNHVIAAEANTVDDHHLLCQSHEDCFKKGAGNFCAPFLEHDVKYGWCFRA

ESEGFLLKDFLKTPADTLKMPNAITN

>ctr28841_c1_g1_i8

MSTCKEGTKAKQNLISLINSIGKMAFVRRASLVALFFLFAASVMFAVKKTEAGFNSCSEAC

VYLPCFSKGCSCFKRQCYKNHVIAATSKSIDEHHLLCQSHYDCITKGSGNFCAPFLEHDVPYGWCFRA

ESEGYLLKDFLKKPMKIAI

>ctr28192_c2_g2_i1

SVMFTVEAGIPCGESC

VFIPCLTTVVGCSCKNKVCYNNHVIAAEANSIDDHHLLCQSHDDCIKKGTGNFCAPFLDHAVQYGWCFRA

ESEGYLLKDFLKMP

>ctr29746_c1_g1_i6

MFTVEAGIPCGESC

VFIPCISSVVGCSCKSKVCYNNHVIAAEANSVDDHHLLCQSHDDCIKKGTGNFCAP

>ctr29746_c1_g1_i10

MAYVRLACLAVIFFFAASVEAGIPCGESC

VFIPCISSVVGCSCKSKVCYNNHVIAAEANSVDDHHLLCQSHEDCIIKGSGNFCAHFSNQDIKYGWCFRA

ESEGFLLKDHLKLTTA

>ctr29746_c1_g1_i2

MAYVRLACLAVIFFFAASVMFTVEAGIPCGESC

VFIPCISSVVGCSCKSKVCYNNHVIAAEANSVDDHHLLCQSHEDCIIKGSGNFCAHFSNQDIKYGWCFRA

ESEGFLLKDHLKLTTA

>ctr28841_c1_g3_i1

EAGISCGESC

VFIPCITGIAGCSCKNKVCYLNHVIAAEAKTMDEHHLLCQSHEDCIKKGTGNFCAPFLEHNVNYGWCFNA

QSEGYLLKDFLKIPKTITN

>ctr29379_c3_g2_i7

SSLRLLVYMFHYFLFVFLMIFYLCISFTNIRIYDFFNMFILIHFVMFTVEKIEADTIPCGESC

VFIPCTSSVLGCSCKDKFCYNNHVIAATSKSMDEHHLLCQSHYDCITKGSGNFCAPFLEHDVPYGWCFRA

ESEGFLLKDFLKTPADTLKMPNAI

>ctr29379_c3_g2_i1

PSYNKYLRSTKSEMAYIRLASLVVLFFFAASVEKIEADTIPCGESC

VWIPCISSILGCSCKDKVCYHNHVIAASSKSIDEHHLLCQSHYDCITKGSG

>ctr29379_c3_g2_i11

PSYNKYLRSTKSEMAYIKLASLAVLFFFAASMEKIEADTIPCGESC

VFIPCTSSVLGCSCKDKFCYNNHVIAATSKSMDEHHLLCQSHYDCITKGSGNFCAPFLEHDVPYGWCFRA

ESEGFLLKDFLKTPADTLKMPNAI

>ctr28841_c1_g1_i6

SC

VWIPCITGAIGCSCKNRVCYRNHVIASEAKTMDDLHLLCQSHDDCITKGSGNFCAPFLEHDVSYGWCFHA

ESEGYLLKDFLKKPMEIAI

>ctr28841_c1_g1_i4

SC

VWIPCITGAIGCSCKNRVCYRNHVIASEAKTMDDLHLLCQSHDDCITKGSGNFCAPFLEHDVPYGWCFRA

ESEGYLLKDFLKKPVKIVI

>ctr28841_c1_g1_i5

SC

VWIPCITGAIGCSCKNRVCYRNHVIASEAKTMDDLHLLCQSHDDCITKGSGNFCAPFLEHDVPYGWCFHA

ESEGYLLKDFLKKPMKIVI

>ctr28841_c1_g1_i7

MTYIRLASLLVLFFFAASVEKMEADTTPCGESC

VWIPCVSSIVGCSCQNKVCYQNHVIAATSKSIDEHHLLCQSHYDCITKGSGNFCAPFLEHDVPYGWCFHA

ESEGYLLKDFLKKPMKIVI

>ctr29379_c3_g2_i5

PSYNKYLRSTKSEMAYTRLASLVVFFFFAASVEKMEANIFPCGESC

VYIPCITSIVGCSCQNKVCYNNHVIAATSKSMDEHHLLCQSHDDCITKGSGNFCAPFLEHDVSYGWCFHA

ESEGYLLKDFSKKPMKIAI

>ctr28926_c1_g1_i1

IKNNMASFRFAPLALIFIFALCVMFAVDNTEAGSITGCGGGC

LLGRCYRPGCTCVRRICRRNHIVAAEAKTVDDHRLLCISHEDCFRKGSGNYCAFSPNTNIHYGWCFYA

ES

>ctr28926_c1_g1_i4

IKNNMASFRFAPLALIFIFALCVMFAVDNTEAGSITGCGGGC

LLGRCYRPGCTCVRRICRRNHIIAAEANTVDDHHLLCESHDDCFKKGSGYYCAFFPDTNIHYGWCIYA

ESEGYKLKDFLETSINLEISMAITN

>ctr28926_c1_g1_i3

IKNNMASFRFAPLALIFIFAICVHNTEAGSAIRCGERC

LLGRCHRPGCTCVRRICRRNHIIAAEANTVDDHHLLCESHDDCFKKGSGYYCAFFPDTNIHYGWCIYA

ESEGYKLKDFLETSINLEISMAITN

>ctr28926_c1_g1_i2

IKNNMASFRFAPLALIFIFAICVMFAVHNTEAGSAIRCGERC

LLGRCHRPGCTCIRRICRRNHVIAAEANTVDDHHLLCESHEDCFKKGSGNYCAFFPNTNIHYG

>ctr28926_c1_g1_i6

IKNNMASFRFAPLALIFIFAICVHNTEAGSAIRCGERC

LLGRCHRPGCTCIRRICRRNHVIAAEANTVDDHHLLCESHEDCFKKGSGNYCAFFPNTNIHYG

>ctr28926_c1_g3_i1

MASIRFAPLALLLIFATCVMFAVQNTEAGSSIVTCGETC

LRGKCYTPGCTCVRPICKKNHIVAA

>ctr28926_c1_g3_i2

SDLVTLPIKWYPAPQNLTVRKEIKNNMASFRFAPLALVLIFATCVMFAVDNTEAGSVIGCGETC

LRGRCYTPGCTCDHGICKKNHI

>ctr28495_c0_g5_i3

MAYVRLTSLAVLFFLAASVMLNVKKTEGANIPMTCGPC

LTDECWTPGCEYHCKYCKNSRSTSSFM

>ctr28495_c0_g3_i1

MAYVRLTSLPLLFFIAASVMLTVQKTEGGNPIVCGETC

FFQKCYTPGCSCDAVICTNNHIIATKAKTDIC

>ctr28926_c2_g2_i1

QQFTPTDPINCYMHVKETNAYETKFCYFPNQIMAYLRLAPLAVIFLFAVMFAVEKTEGGLPICGETC

FTGTCYTPGCTCSYPVCKKNHIIAIAAQAVDQHRLLCESHEDCLKKGTGNYCA

>ctr28495_c0_g7_i1

LPTCGETC

TLGTCYVPDCSCSWPICMKNHIIAANAKTVNEHRLLCTSHEDCFKKGTGNYCASFPRSEERRV

>ctr28495_c0_g7_i3

LPTCGETC

TLGTCYVPDCSCSWPICMKNHIIAANAKTVNEHRLLCTSHEDCFKKGTGNYCASFPDSNIHFGWCFHA

ESEGYLL

>ctr28926_c2_g3_i3

YLRLAPLAVIFFFAVIFAVKKTEGGDPFKCGESC

FAGKCYTPGCTCSRPICKKNHIVAAEAKTVDDHHLLCKSHEDCFRKGTGNYCAFFPNTNIHYGWCFYA

ESEGYM

>ctr28495_c0_g4_i1

KIEGGDPLACGETC

FGGTCYTPGCVCDPWPICTKNHIIASTAKTVDQHRLLCESHEDCLKKGTGNYGASFPDSD

>ctr28192_c2_g4_i3

PKQIMAYLRLAPLAVIFLFAVMFAVKKTEAGDALKCGETC

FGGTCYTPGCSCDYPICKKNHIIALNAKTTTQHHFLCESHEDCLKKGTGNYCVSFPDSDINFGWCFFS

ESEGYLLKDFLKMSKDN

>ctr28926_c1_g2_i1

LRLAPLALILFFAVMFAAKKTEGGDLFKCGETC

FGGTCYTPGCSCDYPICKNNHIMALDAKTVDQHRLLCESHEDCLKKRTGNYCAPFPDSDIHFGWCFHA

ESEGYLLKDFLNMSKDN

>ctr28192_c2_g3_i3

MAYVRLASLAVIFFLATSLMFTLKKTEGGRPTCGETC

FKTKCYTPGCSCSYPICKKNHIIAIEAKTVDE

>ctr28192_c2_g6_i2

LKKTEGCLPICGETC

FKTKCYTKGCSCSYPICKKNHIIAIEAKTVDEHRLLCESYEDCFKKGTGNYCASFPDSNIHFGWCFHA

ESEGY

>ctr28495_c0_g2_i2

PITHFRMAYLRLTSLAVIFFLATSVMFTLKKTEGGRPTCGETC

FKTKCYTKGCSCSYPVCKRNHIIALEAKTVDEHRLLCESHEDCFKKGTGNYCASFPNSDIHFGWCFYA

ESEGYLLKDFLKMSKDDLKTPIESPY

>ctr28192_c2_g6_i1

FKTKCYTPGCSCSYPVCKRNHIIALEAKTVDEHRLLCESHEDCFKKGTGNYCASFPDSDIHFGWCFYA

ESEGYL

>ctr28495_c0_g2_i1

MAYVRLTSLAVVFFIATSVMFTLKKTEGGLPICGETC

FKTKCYTKGCSCSYPVCKRNHIIALEAKTVDEHRLLCESHEDCFKKGTGNYCASFPNSDIHFGWCFYA

ESEGYLLKDFLKMSKDDLKTPIESPY

>cT42_precursor_partial

MAYLRLFVIFFFATSVKKTKADIPKADIPCGSTCLHVKCI

PPCYCKNKVLCYRNVVIATTSKSVNDYY

>cT38_precursor

MANVKLATLLVNFLLVTSVMFVVKKTEAKIPCGESCVWIP

CFTSAFGCYCQSKVCYHSTQIASTAKTMNDHHLLCQSHED

CIIKKSGNFCAHFSNQD

>cT32_precursor_partial

LFFAVMFAAKKTEGGDLFKCGETCFGGTCYTPGCSCDYPI

CKNNHIMALDAKTVDQHRLLCAMIWLFM

>cT31_precursor_partial

MAYLRLAPLAVIFFFAVMFAVKKTEGGDPLKCGESCFAGK

CYTPGCTCDRPICKKNHIVAAEAKT

>cT30_precursor_partial

MAYLRLAPLAVIFFFAVMFAVKKTEGGDPLKCGESCFAGK

CYTPGCTCSRPICKKNHIVAAEAKTVDDHHLLCKSHEDCF

RKGTGNYCAFFPNTNIHYGW

>cT27_precursor

MASLRIAPFAVFLFLAASVMFAVEKTQAGVIPCGESCVFI

PCITGAIGCSCKSKVCYRNHVIAAEAKTMDDHHLLCQSHE

DCITKGTGNFCAPFPDQDIKYGWCFR

>cT23_precursor_partial

VKETQAGFPCGESCVFIPCTVTALLGCSCKDKVCYKNHVI

AAEANTVDDHHLLCQSHEDCF

>cT26_precursor_partial

MFSVKEAKAGFICGESCVYIPCITALLGCSCSNQICSKNH

VIAS

>cT25_precursor_partial

MASFRFAPLALIFIFAICVMFAVHNTEAGSIRCGERCLLG

RCHRPGCTCVRRICRRNHIIAAEANTVDDHHLLCESHEDC

FKKGSGNYCAFFPDTNIHYGWCFNAESEGYMLKDFLETSI

KDNLEIPVAI

>Cter_acyc_1_precursor_partial

MTYVRLAPLIVIFLLLPSVKNTEAVDGFCLETCVILPCFS

SVAGCYCHGSTCMRGTTIASMAKTIDEHRNLCQTHEDCIT

KKSGNFCARFPNHNINYGWCFNAES

>Cter_36_precursor_partial

AYLRLAPLAVIFLLAASVSKTEGGSPTCGETCFGGTCYTP

NCVCDPWPICTKNHIIASTAKTVDQYRLLCESHEDCLKKG

>Cter_33_precursor_partial

MAYLRLAPLAVIFFFAVMFAAKKTEGGDLFKCGETCFGGT

CYTPGCSCDYPICKKNHIIALNAKTTTQHHFLCESHED

>Cter_28_precursor_partial

MASVRLAPFAVIFLFATSVMLIVKDTEAGVIPCGESCVWI

PCISAAIGCSCKKNVCYRNHIIASEATTMDEHHLLCQSH

>Cter_25_precursor_partial

MAYVRLTSLPLLFFIAASVMLTVQKTEGGNPIVCGETCFF

QKCYTPGCSCDAVICTNNHIIATKAKTVDEHRLLCESHED

CFKKGTGNYCASFPDSDIHFG

>Cter_13_precursor

MASFRFAPLALIFIFAICVMFAVHNTEAGSAIRCGERCLL

GRCHRPGCTCIRRICRRNHVIAAEANTVDDHHLLCESHED

CFKKGSGNYCAFFPDTNIHYGWCFNAESEGYMLKDFLETS

IKDNLEIPMAITN

>Cter_9_precursor_partial

YFHQRNHTMAFLRLAPLAVICLIATSVIFTVKETEAGIPC

GESCVYIPCTVTALLGCSCRDKVCYKNHVIASEAKAIDDH

HLLCQ

>Cter_1_precursor_partial

GLPICGETCFGGTCNTPNCVCDPWPICTNNHIIAAAAKTV

DQYRLLCESHEDCLKKGTGNYCASFPNSDIHFGW

>cliotide_T40_precursor

MAYPRLAVIFLLAASVMFAVKDAEAGIPCGESCVFIPCTI

TALLGCSCKSKVCYKNHVIAAEANIVDDHHLLCQSHEDCF

KKGSGNFCAPFLEQDVKYGWCFRAESEGFLLKDFFKMSTD

NLKMLKSITSN

>cliotide_T33_precursor

MAFVRRASLVALFFLFAASVMFAVKKTEAGFNSCSEACVY

LPCFSKGCSCFKRQCYKNHVIAATSKSIDEHHLLCQFHED

CFRKGSGNFCAPFLNYEVLYGWCFRA

>cliotide_T11_precursor_partial

FLATSVMLSVKEAEAGIPCGESCVFIPCTITALLGCSCKD

KVCYKNHVIASEAKTMDDHHLLCQSHEDCIT

>cliotide_T28_precursor

MAYVKHAPLAVVFLLATSVMFIVKNAEAGGSIPCGESCVF

LPCFLPGCSCKSSVCYLNHIIASDAKTVDEHHLLCQSHED

CFKKGTGNFCAAFPDHDIAYGWCFHAQSEGYLLKDFLKMP

KGILKMPMEIAN

>cliotide_T10_precursor

MAFARLAVIFFLAASVMFAVKETQAGIPCGESCVYIPCTV

TALLGCSCKDKVCYKNHVIAAEANTVDEHHLLCQSHEDCF

KKGAGNFCAPFLGHDVKYGWCFRAESEGFLLKDFLKTSAD

TLKMPNAITN

>cliotide_T21_precursor_partial

MAYLRLFTLAAVIFFFAASVEKTKADLQCAETCVHSPCIG

PCYCKHGLICYRNHVIAAAAKSVNDYYLLCQSHE

>cliotide_T19_precursor

MASFRFAPLALVLMFATCVMFAVDNTEAGSVIKCGESCLL

GKCYTPGCTCSRPICKKNHIVAAEAKTVDDHHLLCKSHED

CFRKGTGNYCAFFPNTNIHYGWCFYAESEGYMLKDFLETS

IKDNLEIPMAITN

>cliotide_T18_precursor

MAYLRLAPLAVIFLFAVMFAVEKTEGGLPICGETCFTGTC

YTPGCTCSYPVCKKNHIIAIAAQAVDQHRLLCESHEDCFK

KGTGNYCASFPDSDIHFGWCFHAESEGYLLKDFLKMSKDD

LKMPTEITN

>cliotide_T13_precursor

MTYIRLASLLVLFFFAASVEKMEADTTPCGESCVWIPCVS

SIVGCSCQNKVCYQNHVIAATSKSIDEHHLLCQSHYDCIT

KGSGNFCAPFLEHDVPYGWCFHAESEGYLLKDFLKKPMKI

VI

>cliotide_T15_precursor

MAYVRLTSLAVVFFIATSVMFTLKKTEGGLPICGETCFKT

KCYTKGCSCSYPVCKRNHIIALEAKTVDEHRLLCESHEDC

FKKGTGNYCASFPDSDIHFG

>cliotide_T1_precursor

MASLRIAPLALFFFLAASVMFTVEKTEAGIPCGESCVFIP

CITGAIGCSCKSKVCYRNHVIAAEAKTMDDHHLLCQSHED

CITKGTGNFCASFPEQDIKYGWCFRAESEGFMLKDHLKMS

ITN

>Cter_37_precursor_partial

AYLRLAPLAVIFLLAASVSKTEGGSPTCGETCFGGTCYTP

GCVCDPWPICTKNHIIASTAKTVDQYR

>Cter_35_precursor_partial

FFLAASVMFTMEKTEGGAFCGETCVLGTCYTPGCSCAPVI

CLNNHVIAATAKTTDEHRLLCKSHEDCFRKGTGNYCA

>Cter_32_precursor_partial

MANVKLATLLVNFLLVTSVMFVVKKTEAKIPCGESCVWIP

CISSILGCSCKDKVCYHNHVIAASSKSIDEHHLLC

>Cter_31_precursor_partial

DNLNQKMAYLRLFTLAAVIFFFAASVEKTKADLQCAETCV

HSPCIGPCYCKHGVICYKNHVIAAAAKSVNDYYLLCQSHE

>Cter_30_precursor

MAYVRLASLAVIFFLATSLMFTLKKTEGGFPICGETCFKT

KCYTPGCSCSYPVCKKNHIIAIEAKTVDEHRLLCESHEDC

FKKGTGNYCASFPNSDIHFGWCFYAESEGYLLKDFLKMSK

DDLKTPIESPY

>Cter_29_precursor_partial

MAYLRLVPLLVLFFFAASVNKTEAGALCDERCTYVPCISA

ARGCSCNIHRVCSMNHVIAATSKSI

>Cter_22_precursor_partial

LTSLAVLFVLAASVMFTVEKTEGNTAFCGETCVLGTCYTP

DCSCTAIVCIKNHVIAATAKKTDDHRLLCESH

>Cter_20_precursor_partial

VMFAVEKTEAGVIPCGESCVYLPCLTTIVGCSCKNNVCYT

NHVIAATAKSLDEHRLLCQSHEDCFTKGSGNFCA

>Cter_19_precursor_partial

MAKLVPLIVIFLVAASVDMTKASIPCGESCVYIPCLTTIV

GCSCKSNVCYSNHVIAATAKSLDEHRLLCQSHEDCFTKGT

GNFCAHFPEGDVAYGWCFRA

>Cter_18_precursor_partial

MAYLRLFTLAAVIFFFAASVEKTKADLICSSTCLHTPCKA

SVCYCKNAVCYKNHVIAATSNSVNDYYLLCQSHE

>Cter_15_precursor

MAFARLAVIFFLAASVMFAVKETQAGIPCGESCVFIPCTV

TALLGCSCKSKVCYKNHVIAAEANTVDDHHLLCQSHEDCF

KKGAGNFCAPFLGHDVKYGWCFRAESEEFLLKDFLKTPAD

ILKMPNAISN

>Cter_12_precursor_partial

LTSLAVLFVLAASVMFTVEKTEGNTAFCGETCVLGTCYTP

DCSCKAVVCIKNHVIAATAKTTDEHRLLCKSHEDCFRKGT

G

>Cter_11_precursor_partial

RFAPLALIFIFAICVMFAVHNTEAGSIRCGERCLLGRCHR

PGCTCIRRICRRNHVIAAEANTVDDHHLLCESHED

>Cter_10_precursor_partial

FLATSVMFSMKEAEASYIPCGESCVYIPCTVTALLGCSCS

NKVCYKNHVIASEAKTMDDHALLCQSHEDCIIKGTGNFCA

>Cter_8_precursor_partial

MAYVRLTSLAVLFVLAASVMFTVEKTEGGSAFCGETCVLG

TCYTPDCSCKAVVCIKNHVIAATAKTTDEHRLLCKSHE

>Cter_7_precursor_partial

MAYLRLAPLAVIFFFAVMFAVKKTEGGDPFKCGESCFAGK

CYTPGCTCEYPICMNNHIIALDAKTMDQHRLLCESHEDCL

KKRTGNYCAPFPDSDIHFGWCFHAE

>Cter_5_precursor_partial

FLAASVMLNVKKTEGGEFLKCGESCVQGECYTPGCSCDYP

ICKNNHIMALDAKTVDQHRLLCESHEDCLKKRTGNYCAPF

PDSDIHFGWCFHAESEGYLLKDFLKMSKDDLKMPTEITN

>Cter_4_precursor_partial

AYLRLAPLAVIFLLAGSVYKIEGGDPLACGETCFGGTCYT

PGCVCDPWPICTKNHIIASTAKTVDQHRLLCESHED

>Cter_3_precursor_partial

FFLAASVMFTMEKTEGGAFCGETCVLGTCYTPDCSCKAVV

CIKNHVIAATAKTTDEHRLLCKSHEDCFRKGTGNYCA

>Cter_2_precursor_partial

VKKTEGGDPLKCGESCFAGKCYTPGCTCEYPICMNNHIIA

LDAKTMDQHRLLCESHEDCLKKRTGNYCAPFPDSDIHFGW

CFHAESEGYLLKDFLKMSKDDLKMPTEITN

>Cter_B_precursor

MGTIARYYAHVVLFLVATSVIFTVKKTEAGVPCAESCVWI

PCTVTALLGCSCKDKVCYLNHVIAFEAKTMDEHHLLCQSH

EDCYKKGSGNFCAPFFNHDVKYGWCFRAEFEGYLLKDFLK

MQPRDILKISKAIAK

>Cter_A_precursor

MASLRIAPFALFLFLAASVMFAVEKTEAGVIPCGESCVFI

PCISTVIGCSCKNKVCYRNHIIAAEAKTMDEHILLCQSHE

DCIAKGTGNFCAPFPDQDIKYGWCFRAESEGFMLKDHLKM

SITN

>cliotide_T12_precursor

MASLRIAPLALFFFLAASVMFTVEKTEAGIPCGESCVFIP

CITGAIGCSCKSKVCYRDHVIAAEAKTMDDHHLLCQSHED

CITKGTGNFCASFPEQDIKYGWCFRAESEGFMLKDHLKMS

VPN

>cliotide_T2_precursor

MAYVRLTSLAVLFFLAASVMLNVKKTEGGEFLKCGESCVQ

GECYTPGCSCDWPICKKNHIIATNAKTVNQHRLLCESHED

CFKKGTGNYCAFFPDSDVHFGWCFYAESDGYLLKDFFKMS

KDNLKMPMTIIN

>cliotide_T9_precursor_partial

MAYVRLACLAVIFFFAASVMFTVEAGIPCGESCVFIPCLT

TVVGCSCKNKVCYNNHVIAAEANSIDDHHLLCQSHDDCI

KKGTGNFCAPFLDHAVQYGWCFRAESEGYLLKDFLKMP

>Cter_P_precursor

MASLRIAPLALFFFLAASVMFTVEKTEAGIPCGESCVFIP

CITAAIGCSCKSKVCYRNHVIAAEAKTMDDHHLLCQSHED

CITKGTGNFCAPFPDQDIKYGWCFRAESEGFLLKDHLKMS

ITN

>Cter_R_precursor

MAFARLALIFFLAASVMFAVKETEAGIPCGESCVFIPCTV

TALLGCSCKDKVCYKNHVIAAEANTVNDHHLLCQSHEDC

FKKGTGNFCAPSLKHDVKYGWCFRAESEGFLLKDFLKTPV

DILKMSNVIGN

>cliotide_T8_precursor

MAYVRLACLAVIFFFAASVMFTVEAGIPCGESCVFIPCIS

SVVGCSCKSKVCYNNHVIAAEANSVDDHHLLCQSHDDCIK

KGTGNFCAPFLDHAVQYGWCFRAESEGYLLKDFLKMPKAL

TN

>Cter_A_or_Cter_Q_precursor_partial

IPCGESCVFIPCISTVIGCSCKNKVCYRNHVIAAEAKTMD

DHHLLCQSHEDCITKGTGNFCAPFPDQDIKYGWCFRAESE

GFMLKDHLKMSITN

>Cter_P_precursor_variant_partial

IPCGESCVFIPCITGAIGCSCKSKVCYRNHVIAAEAKTMD

DHHLLCQSHEDCITKGTGNFCAPFPDQDIKYGWCFRAESE

GFMLKDHLKMSITN

>Cter_M_precursor

MAYVRLTSLAVLFFLAASVMKTEGGLPTCGETCTLGTCYV

PDCSCSWPICMKNHIIAANAKTVNEHRLLCTSHEDCFKKG

TGNYCASFPDSNIHFGWCFHAESEGYLLKDFMNMSKDDLK

MPLESTN

**B. Asparaginyl endopeptidase protein sequences**

>CtAEP7 (ctr17958_c0_g1_i1)

NGNGHRHSADAKGKRWAILVAGSKGYENYRHQADVCHAYQLLRKGGLKEENIIVFMYDDIAFNPKNPRPNIIINKPHGPNVYKGVPKDYTGDASNSKNLFGVISGNRSAVSGGSGKVLKSGPNDIIFIYYSDHGGAGLIGMPVDGDHILANKLVDALKKKHAAKTYKKMVIYLEACEGGSMFEGLLPNDINIYASTASNASEDSFGIYCPGFYPFAPLEYTTCLGDTYSISWLEDSDKNNMREETLHQQYETVRRRTLYGNMDPGSHVMLYGDRKMNNDFLVTYIGASHAHINHQQNATNLRSSQPSSITQTRLVSQRDTHLLHLRNEMKKAPDGSEEKLKAQKELDVEIAQREYVDNVFHLIGDLLFGEGNRSSMMLHVRLVGQPLVDDWDCFKTLIKTYERHCGTLSSYGRKYLRALANMCNAGISKEQLVVALSQACPS

>CtAEP8 (ctr24604_c1_g1_i1)

IQVPYSYRKPSIDSESANNFFGGKKWVVLVAGSDGWNNYRHQ

ADICHAYQIVRENGIPKENIITMMVDDIANNPRNPVPGTIINQPNGTDVYKGVVIDYKGT

DVNSTNFLKIITGDKKAMQFIGTGKVIEGGPRDRIFINFVDHGTTGILGFPDDLLYADEL

NDALKTMYASASYRMVLMYIEACKAGSMFDGILRDNTDVLAVTASGPRENSYGCYCRSQS

GPYKTCLGDLFSVTWMENWDATVSQPSTRKRTVFHDFKECRTNVTESNVMVYGDFKTGHEPLSAFIGYKTHSKTHLSAEPIMDITNEPKNTVSSRNVRENTVQQQLASHELSFSERRRLS

TESRLNNEMRLIIDTALRTIYSKVVKARPEIKSKVGDFYEPNHLDLSLDVFPCYRSILNK

ITESCFSLPRNPYALDRLTIFANFCIVDKHIHQMVEKLVSASCSKVQKLNLRNVY

>CtAEP9 (ctr24604_c1_g2_i2)

AHVPKSTANEESFVGGKKWVVLVAGSEGWSNYRHQADICHAY

QIVKENGIPEENIITMMVDDIAYNSRNPTPGKIINKPKGNDVYQGVIIDYKGDDVNKSNF

LKIITGDQAGMRSIGTGKVVLGGQLDRIFINFVDHGASGLLGFPDDYLYADELNDAFTTM

NNNESYKKMLLYIEACKAGSMFDGILSEDTNIFAVTASGPRESSYGCYCQSESGPYKTCL

GDLFSVTWMEDLDKPTSKQSARKRTVFNDFTVTRTNVTKSNVMIYGDLDTGSEKLSSFIG

YRGSGGDDSSFVQQSDELNIKNTASSRDVHESSVKYELEHDMLSLPEALKLTAKLRKNNE

MRSVIDSVFRDIYSEVVKERPDVKSEIGEYDEPNNLKLNLAMFPCYRSVLNLITENCFSL

PKNPYVLDHLTVFANMCVADNQIHEMVGSIVTKSCSNIPKNIINVQ

**>CtAEP10 (ctr27109_c1_g4_i1)**

**LVAGSKGYVNYRHQADVCHAYQILKKGGLKDENIIVFMYDDIAYNESNPHPGVIINHPYGSDVYKGVPKDYVGEDINPPNFYAVLLANKSALTGTGSGKVLDSGPNDHVFIYYTDHGGAGVLGMPSKPYIFASDLNDALKKKHASGTYKSLVFYVESCEAGSMFDGLLPEDHNIYVMAASDTGESSWVTYCPFQDPRLPPEYDVCIGDLFSVSWLEDSDVHNLRTETIHQQCEVVKKKTIEPLIRDGTHIVQFGDTGLSNQSLFVYMGTDPANDNNTFVDKHSLVPPRKAVSQRDADLIHFWEKFRRAPEGSSRKAEAEKQLREVTSHRMHIDHSVKHIGKLLFGIEKGSKMLNSVRPAGLPVVDDWDCLKTLVRT**

>CtAEP11 (ctr28924_c0_g1_i1)

AIVKLIIVGPSNFIDECLELFGGAKKQTWVFLV

AGSKGWDNYRHQADVSHAYQTLLNNGIPVDRIIVMMTDDVAFDPKNPYSGELFNHPNGSD

VYQGVQVDYKGEEVKSEHFLKVLNGNKTAMMNIGSGRVIESNHRDNIFVYFVGHGTSGIL

AFPENYLYADELNNALQSMYSDHKFNGMLLYIESCRSGSLFDGILSESNNIFAVTAAGPR

ESSWSIYCIGGDGIPDVCLGDEFSCTWIEDQANLGILYPLQVNELVEKRTVLNHFNYIRT

TVKLSNVMPYGDFSVGQDKLSTYIGKSPEVFSKYSYHNSDIPSFNKQNDTNMSSSKFSKN

MYKPYQTKELHNEKNYSLKQSSIYNSIMRKVMDRTFSTILQKVIKQLPGFIGHIDELKEL

PSNLPLNVFPCYRQTLDHITANCFSLPKVKLQIINYLKFNHLYSVQNNAYDMAHGING

>CtAEP12 (ctr28924_c0_g1_i2)

AIVKLIIVGPSNFIDECLELFGGAKKQTWVFLV

AGSKGWDNYRHQADVSHAYQTLLNNGIPVDRIIVMMTDDVAFDPKNPYSGELFNHPNGSD

VYQGVQVDYKGEEVKSEHFLKVLNGNKTAMMNIGSGRVIESNHRDNIFVYFVGHGTSGIL

AFPENYLYADELNNALQSMYSDHKFNGMLLYIESCRSGSLFDGILSESNNIFAVTAAGPR

ESSWSIYCIGGDGIPDVCLGDEFSCTWIEDQANLGILYPLQVNELVEKRTVLNHFNYIRT

TVKLSNVMPYGDFSVGQDKLSTYIGKSPEVFSKYSYHNSDIPSFNKQNDTNMSSSKFSKN

MYKPYQTKELHNEKNYSLKQSSIYNSIMRKVMDRTFSTILQKVIKQLPGFIGHIDELKEL

PSNLPLNVFPCYRQTLDHITANCFSLPKNIYSLNEVHVFFNLCTRVINSEWKSEEFYNVF

EQVVHNVCSKNSSTFSNLNKITDIY

>CtAEP2 (ctr29014_c2_g1_i2)

ARLNPQKEWDSVIRLPTEPVDADTDEVG

TRWAVLVAGSNGYENYRHQADVCHAYQLLIKGGLKEENIVVFMYDDIAWHELNPRPGVII

NNPRGEDVYAGVPKDYTGEDVTAENLFAVILGDRSKVKGGSGKVINSKPEDRIFIFYSDH

GGPGVLGMPNEQILYAMDFIDVLKKKHASGGYREMVIYVEACESGSLFEGIMPKDLNVFV

TTASNAQENSWGTYCPGTEPSPPPEYTTCLGDLYSVAWMEDSESHNLRRETVNQQYRSVK

ERTSNFKDYAMGSHVMQYGDTNITAEKLYLFQGFDPATVNLPPHNGRIEAKMEVVHQRDA

ELLFMWQMYQRSNHLLGKKTHILKQIAETVKHRNHLDGSVELIGVLLYGPGKGSSVLQSV

RDPGLPLVDNWACLKSMVRVFESHCGSLTQYGMKHMRAFANICNSGVSESSMEEACMVAC

GGHDAGHLHPSKRGYIA

>ALG36103.1_OaAEP1b

ARDGDYLHLPSEVSRFFRPQETNDDHGEDSVGTRWAVLIAGSKGYANYRHQAGVCHA

YQILKRGGLKDENIVVFMYDDIAYNESNPRPGVIINSPHGSDVYAGVPKDYTGEEVNAKNFLAAILGNKSAITG

GSGKVVDSGPNDHIFIYYTDHGAAGVIGMPSKPYLYADELNDALKKKHASGTYKSLVFYLEACESGSMFEGILPEDLNIYALTSTN

TTESSWCYYCPAQENPPPPEYNVCLGDLFSVAWLEDSDVQNSWYETLNQQYHHVDKRISHASHATQYGNLKLGE

EGLFVYMGSNPANDNYTSLDGNALTPSSIVVNQRDADLLHLWEKFRKAPEGSARKEEAQTQIFKAMSHRVHIDSSIKLIG

KLLFGIEKCTEILNAVRPAGQPLVDDWACLRSLVGTFETHCGSLSEYGMRHTRTIANICNAGISEEQMAEAASQACASIP

>ALG36104.1_OaAEP2

ARDGYLKLPSEVSDFFRPRNTN

DGDDSVGTRWAVLLAGSNGYWNYRHQADLCHAYQILKRGGLKDENIVVFMYDDIAYNE

ENPRPGVIINSPHGSDVYAGVPKDYTGDQVNAKNFLAAILGNKSAITGGSGKVVNSGP

NDHIFIYYTDHGGPGVLGMPVGPYIYADDLIDTLKKKHASGTYKSLVFYLEACESGSM

FEGLLPEGLNIYATTASNAEESSWGTYCPGEYPSPPPEYDTCLGDLYSVAWMEDSEVH

NLRSETLKQQYHLVKARTSNGNSAYGSHVMQYGDLKLSVDNLFLYMGTNPANDNYTFV

DDNALRPSSKAVNQRDADLLHFWDKFRKAPEGSARKEEARKQVFEAMSHRMHIDNSIK

LVGKLLFGIERGAEILDAVRPAGQPLADDWTCLKSLVRTFETHCGSLSQYGMKHMRTI

ANICNAGITKEQMAEASAQACSSVPSNPWSSLHKGFSA

>ALG36105.1OaAEP3

EERTDGYLKLPTEVSRFFRT

PEQSSDGGDDSIGTRWAVLIAGSKGYDNYRHQADVCHAYQILKRGGLKDENIVVFMYD

DIAYNESNPRPGVIINSPHGSDVYAGVPKDYTGDEVNAKNFLAAILGNKSAITGGSGK

VVDSGPNDHIFIYYTDHGAPGVIGMPSKPYLYADELNDALRKKHASGTYKSMVFYLEA

CEAGSMFDGLLPDGLNIYALTASNTTEGSWCYYCPGQDAGPPPEYSVCLGDFFSIAWL

EDSDVHNLRSETLNQQYHNVKNRISYASHATQYGDLKRGVEGLFLYLGSNPENDNYTF

VDDNVVRPSSKAVNQRDADLVHFWEKFRKAPEGSSKKEEAQKQILEAMSHRVHIDSSI

NLIGKLLFGIEKGHKILTAVRSAGHPLVDDWACLRSLVRTFETHCGSLSQYGMKHTRT

LANICNAGITEEQMAEAASQACVSIPSNPWSSHDGGFSA

>OaAEP4

ARDGYLKLPSEVSDFFRPRNTNDGDDSVG

WAVLLAGSNGYWNYRHQADLCHAYQILKRGGLKDENIVVFMYDDIAYNEENPRPGVIINS

PHGSDVYAGVPKDYTGDEVNAKNFLAAILGNKSAITGGSGKVVDSGPNDHIFIYYTDHGA

PGVIGMPSKPYLYADELNDALRKKHASGTYKSMVFYLEACEAGSMFDGLLPDGLNIYALT

ASNTTEGSWCYYCPGQDAGPPPEYSVCLGDFFSIAWLEDSDVHNLRSETLNQQYHNVKN

RISYASHATQYGDLKRGVEGLFLYLGSNPENDNYTFVDDNVVRPSSKAVNQRDADLVHFW

EKFRKAPEGSSKKEEAQKQILEAMSHRVHIDSSINLIGKLLFGIEKGHKILTAVRSAGHP

LVDDWACLRSLVRTFETHCGSLSQYGMKHTRTLANICNAGITEEQMAEAASQACVSIPSN

PWSSHDGGFSA

>OaAEP5

EERTDGYLKLPTEVSRFFRTPEQSSDGGDDSI

WAVLIAGSKGYDNYRHQADVCHAYQILKRGGLKDENIVVFMYDDIAYNESNPRPGVIINS

PHGSDVYAGVPKDYTGEEVNAKNFLAAILGNKSAITGGSGKVVDSGPNDHIFIYYTDHGA

AGVIGMPSKPYLYADELNDALKKKHASGTYKSLVFYLEACEAGSMFEGLLTDDLNIYALT

ASNATEGSCPYYCPGDLNYSPPPEYDVCLGDFFSIAWLEDSDVHNLRSETLNQQYHNVKN

RISYASHATQYGDLKRGVEGLFLYLGSNPENDNYTFVDDNVVRPSSKAVNQRDADLVHFW

EKFRKAPEGSSKKEEAQKQILEAMSHRVHIDSSINLIGKLLFGIEKGHKILTAVRSAGHP

LVDDWACLRSLVGTFETHCGSLSEYGMRHTRTIANICNAGISEDQMKEAASQACASVPSN

SWSSLXKGFHARLAKIIA

>AWD84476.1_PxAEP3b

RNVLKLPSEASRFFKKGEDDDSVGTRWAVLLAGSNSYWNYRHQADVCHAY

QLLRKGGLKDENIVVLMYDDIAYNEENPRKGVIINNPAGEDVYKGVPKDYTGDDVNVDNFLAVLLGNKTAITGGSGKVVD

SGPNDHIFIFYTDHGGPGVLGMPTKPYLYASDLIGALKKKHASGTYKSLVLYVEACEAGSIFEGLLPEGLNVYATTASDA

VEGSWVTYCPGQNPSPPPEYTTCLGDLYSVSWMEDSEKHNLQTESLRQQYHLVKEKIAYASHVMQYGDLKLSMD

SLSMYMGTDPANDNYTFVDDNSLGTSSKAVNQRDADLLHFSDKFLKAPEGSARKVEAQKQFAEAMSHRLHLDNSMALVGK

LLFGIKKGPEVLKRVRSDGQLLVDDWACLKSFVRTFETHCGSLSQYGMKHMRSFANICNAGIKVEQMVEASSQACPSVPS

NTWSSLHRGFSA

>AIB06797.1_CtAEP1_butelase1

IRDDFLRLPSQASKFFQADDNVEGTRWAVLVAGSKGYVNY

RHQADVCHAYQILKKGGLKDENIIVFMYDDIAYNESNPHPGVIINHPYGSDVYKGVPKDY

VGEDINPPNFYAVLLANKSALTGTGSGKVLDSGPNDHVFIYYTDHGGAGVLGMPSKPYIA

ASDLNDVLKKKHASGTYKSIVFYVESCESGSMFDGLLPEDHNIYVMGASDTGESSWVTYC

PLQHPSPPPEYDVCVGDLFSVAWLEDCDVHNLQTETFQQQYEVVKNKTIVALIEDGTHVV

QYGDVGLSKQTLFVYMGTDPANDNNTFTDKNSLGTPRKAVSQRDADLIHYWEKYRRAPEGSSRKAEAKKQLREVMAHRMHIDNSVKHIGKLLFGIEKGHKMLNNVRPAGLPVVDDWDCFKTLIRTFETHCGSLSEYGMKHMRSFANLCNAGIRKEQMAEASAQACVSIPDNPWSSLHAGFSV

>ALL55651.1_CtAEP2_butelase2

ARLNPQKEWDSVIRLPTEPVDADTDEVGTRWAVLVAGSNGYENYR

HQADVCHAYQLLIKGGLKEENIVVFMYDDIAWHELNPRPGVIINNPRGEDVYAGVPKDYTGEDVTAENLFAVILGDRSKV

KGGSGKVINSKPEDRIFIFYSDHGGPGVLGMPNEQILYAMDFIDVLKKKHASGGYREMVIYVEACESGSLFEGIMPKDL

NVFVTTASNAQENSWGTYCPGTEPSPPPEYTTCLGDLYSVAWMEDSESHNLRRETVNQQYRSVKERTSNFKDYAMGSHVM

QYGDTNITAEKLYLFQGFDPATVNLPPHNGRIEAKMEVVHQRDAELLFMWQMYQRSNHLLGKKTHILKQIAETVKHRNH

LDGSVELIGVLLYGPGKGSPVLQSVRDPGLPLVDNWACLKSMVRVFESHCGSLTQYGMKHMRAFANICNSGVSESSMEEA

CMVACGGHDAGHLHPSKRGYIA

>ASR19270.1_CtAEP_butelase6

RHALPGDFLRFPSDQDNLPGTSWAVLLAGSKDYWNYRH

QADICHAYQILRKGGLKEENIIVFMYDDIAFNENNPRPGVIINKPDGDDVYEGVPKDYTG

EDVNVNNFFAVLLGNKSALTGGSGKVLNSGPNDHIFIFYSDHGGPGVLGMPTHPYLYADD

LNEVLKKKHASGTYKRLVFYIEACESGSIFEGLLPEDIDIYATTASNATESSSPTYCPRP

PAEHAPFPEYTTCLGDLYSITWMEDSEKHNLQTETLHQQYKLLKERVSLRSNVMQYGDID

ISSDVLFQYLGTNPTNENFTFMDENYLRSSSKSINQRDADLIHFWHKFHKALEGSTHKNT

AQKQVLEVMSHRMHIDNSVQLIRKLLFSIEKGPETLNKVRPAGSVLVDDWGCLKTMVRTF

ETHCGSLSQYGMKHMRSFANICNARIKNEQMAKASAQACVSIPTNPWSSLQRGFSA

>ALL55652.1_CtAEP3

RHALAGDFLRLPSESDTRGTRWAVLLAGSWGYSN

YRHQADVCHAYQILRKGGLKEENIVVFMYDDIAFNEENPRPGVIINKPDGGDVYEGVPKD

YTGEDVNVNNFLAVLLGNKSALTGGSGKVLNSGPDDHIFIYFTDHGGAGVLGMPAGPFIF

ADDLNEVLKKKHASGTYKRLVFYLEACESGSIFEGLLPEDIDIYATTASNATESSSPTYC

PRPPAEHAPFPEYTTCLGDLYSITWMEDSEKHNLQTETLHQQYKLLKERVSLRSNVMQYG

DIDLSSDVLFQYLGTNPANDNFTFVDENYLRSSSKPVNQHDADLIHFWDKFRKAPGDSAK

KNTAQKQLLEVMSHRMHIDNTVQLIGKLLFGVEKGPEILNNVRPVGSVLVDDWACMKAMVRTFETHCGSLSQYGMKHMRSFANICNAGIKNEQMAEASGQACVSIPANPWSSLQRGFSA

>ALL55653.1_CtAEP5

RHRLAGDFLRLPSDQDNLPGTSWAVLLAGSKDYWNYRH

QADICHAYQILRKGGLKEENIIVFMYDDIAFNENNPRPGVIINKPDGSDVYEGVPKDYTG

EDVNVNNFLAVLLGNKSALTGGSGKVLNSGPDDHIFIYFTDHGGAGVLGMPAGPFIFADD

LNEVLKKKHASGTYKRLVFYLEACESGSIFEGLLPEDINVYATTASNAEESSWGTYCPGD

YDGPPPEYSTCLGDLYSIAWMEDSDIHNLRTETLHQQYKLVKERTIGGGSYYGSHVMQYG

DIDLSSDVLFQYLGTNPANDNFTFVDENYLRSSSKPVNQHDADLIHFWDKFRKAPGDSAK

KNTAQKQLLEVMSHRMHIDNTVQLIGKLLFGVEKGPEILNNVRPVGSVLVDDWACMKAMVRTFETHCGSLSQYGMKHMRSFANICNARIKNEQMAKASAQACVSIPANPWSSLQRGFSA

>XP_011535272.1_HsLEG

MVWKVAVFLSVALGIGAVPIDDPEDGGKHWVVIVAGSNGWYNYRHQADACHAYQIIHRNG

IPDEQIVVMMYDDIAYSEDNPTPGIVINRPNGTDVYQGVPKDYTGEDVTPQNFLAVLRGD

AEAVKGIGSGKVLKSGPQDHVFIYFTDHGSTGILVFPNEDLHVKDLNETIHYMYKHKMYR

KMVFYIEACESGSMMNHLPDNINVYATTAANPRESSYACYYDEKRSTYLGDWYSVNWMEDSDVEDLTKETLHKQYHLVKSHTNTSHVMQYGNKTISTMKVMQFQGMKRKASSPVPLPPVTHLDLTPSPDVPLTIMKRKLMNTNDLEESRQLTEEIQRHLDARHLIEKSVRKIVSLLAASE

AEVEQLLSERAPLTGHSCYPEALLHFRTHCFNWHSPTYEYALRHLYVLVNLCEKPYPLHR

IKLSMDHVCLGHY

>XP_017170484.1_MmLEG

VPVGVDDPEDGGKHWVVIVAGSNGWYNYRHQADACHAYQIIHR

NGIPDEQIIVMMYDDIANSEENPTPGVVINRPNGTDVYKGVLKDYTGEDVTPENFLAVLR

GDAEAVKGKGSGKVLKSGPRDHVFIYFTDHGATGILVFPNDDLHVKDLNKTIRYMYEHKM

YQKMVFYIEACESGSMMNHLPDDINVYATTAANPKESSYACYYDEERGTYLGDWYSVNWMEDSDVEDLTKETLHKQYHLVKSHTNTSHVMQYGNKSISTMKVMQFQGMKHRASSPISLPPVTHLDLTPSPDVPLTILKRKLLRTNDVKESQNLIGQIQQFLDARHVIEKSVHKIVSLLAG

FGETAERHLSERTMLTAHDCYQEAVTHFRTHCFNWHSVTYEHALRYLYVLANLCEAPYPI

DRIEMAMDKVCLSHY

>NP_071562.2_RnLEG

VHIGVDDPEDGGKHWVVIVAGSNGWYNYRHQADACHAYQIIHR

NGIPDEQIIVMMYDDIANNEENPTPGVVINRPNGTDVYKGVPKDYTGEDVTPENFLAVLR

GDEEAVKGKGSGKVLKSGPRDHVFVYFTDHGATGILVFPNEDLHVKDLNKTIRYMYEHKM

YQKMVFYIEACESGSMMNHLPDDIDVYATTAANPNESSYACYYDEERSTYLGDWYSVNWMEDSDVEDLTKETLHKQYHLVKSHTNTSHVMQYGNKSISTMKVMQFQGMKHRASSPISLPPVTHLDLTPSPDVPLTILKRKLLRTNNMKESQVLVGQIQHLLDARHIIEKSVQKIVSLLAG

FGETAQKHLSERAMLTAHDCHQEAVTHFRTHCFNWHSVTYEHALRYLYVLANLCEKPYPI

DRIKMAMDKVCLSHY

>NP_776526.1_BtLEG

VPLEDPEDGGKHWVVIVAGSNGWYNYRHQADACHAYQIVHRNG

IPDEQIIVMMYDDIANSEDNPTPGIVINRPNGSDVYQGVLKDYTGEDVTPKNFLAVLRGD

AEAVKGVGSGKVLKSGPRDHVFVYFTDHGATGILVFPNEDLHVKDLNETIRYMYEHKMYQ

KMVFYIEACESGSMMNHLPPDINVYATTAANPRESSYACYYDEQRSTFLGDWYSVNWMED

SDVEDLTKETLHKQYQLVKSHTNTSHVMQYGNKSISAMKLMQFQGLKHQASSPISLPAVS

RLDLTPSPEVPLSIMKRKLMSTNDLQESRRLVQKIDRHLEARNIIEKSVRKIVTLVSGSA

AEVDRLLSQRAPLTEHACYQTAVSHFRSHCFNWHNPTYEYALRHLYVLVNLCENPYPIDR

IKLSMNKVCHGYY

>NP_001126789.1_PaLEG

VPIDDPEDGGKHWVVIVAGSNGWYNYRHQADACHAYQIIHRNG

IPDEQIVVMMYDDIAYSEDNPTPGIVINRPNGTDVYQGVPKDYTGEDVTPQNFLAVLRGD

AEAVKGIGSGKVLKSGPQDHVFVYSTDHGSTGILVFPNEDLHVEDLNETIHYMYKHKMYR

KMVFYIEACESGSMMNHLPDNINVYATTAANPRESSYACYYDEKRSTYLGDWYSVNWMEDSDVEDLTKETLHKQYHLVKSHTNTSHVMQYGNKTISTMKVMQFQGMKHKASSPISLPPVTHLDLTPSPDVPLTIMKRKLMNTNDLEESRQLTEEIQQHLDARHLIEKSVRKIVSLLAASE

AEVEQLLSERAPLTGHSCYPEALLHFRTHCFNWHSPTYEYALRHLYVLVNLCEKPYPLHR

IKLSMDHVCLGHY

>XP_005562111.1_MfLEG

VPIDDPEDGGKHWVVIVAGSNGWYNYRHQADACHAYQIIHRNG

IPDEQIVVMMYDDIAYSEDNPTPGIVINRPNGTDVYQGVPKDYTGEDVTPQNFLAVLRGD

AEAVKGIGSGKVLKSGPQDHVFVYFTDHGSTGILVFPNEDLHVKDLNETIYYMYKHKMYR

KMVFYIEACESGSMMNHLPDNINVYATTAANPRESSYACYYDEKRSTYLGDWYSVNWMEDSDVEDLTKETLHKQYHLVKSHTNTSHVMQYGNKTISTMKVMQFQGMKHKASSPLSLPPVTHLDLTPSPDVPLTIMKRKLMNTNDLEESRQLTEEIQRHLDARHLIEKSVRKIVSLLAASE

AEVEQLLSERAPLTGHSCYPEALLHFRTHCFNWHSPTYEYALRHLYVLVNLCEKPYPLHR

IKLSMDHVCLGHY

>XP_015625561.1_OsVPE_beta

KRTWEPVIRMPGEVVEEEVATVPRGSEGTEEEEKDGVG

TRWAVLVAGSSGYGNYRHQADVCHAYQILRKGGLKEENIVVFMYDDIANNILNPRPGVIV

NHPQGEDVYAGVPKDYTGDEVTAKNFYAVLLGNKTAVTGGSRKVIDSKPNDHIFIFYSDH

GGPGVLGMPNLPYLYAADFMKVLQEKHASNTYAKMVIYVEACESGSIFEGLMPEDLNIYV

TTASNAEESSWGTYCPGMEPSPPSEYITCLGDLYSVSWMEDSETHNLKEESIKKQYEVVK

KRTSDMNSYGAGSHVMEYGDRTFKDDKLYLYQGFDPANAEVKNKLSWEGPKAAVNQRDADLLFLWRRYELLHDKSEEKLKALREISDTVMHRKLLDSSVDLVGKLLFGFGNGPSVLQAVRPSGQPLVDDWDCLKRMVRIFESHCGPLTQYGMKHMRAFANICNNGISGASMKEASIATCS

SHNSGRWSSLVQGYSA

>NP_001291333.1_SiVPE

GGGRRSGPWDPIIRWPLDRRETEDNATRWAVLVA

GSNGFGNYRHQADVCHAYQILKKGGLRDENIIVFMYDDIAMNELNPRKGVIINHPTGGDV

YAGVPKDYTGEQVTAENLYAVILGDKSAIKGGSGKVVDSKPNDRIFIYYSDHGGPGVLGM

PNMPYLYANDFIEVLKKKHASGTYKEMVIYVEACESGSVFEGLMPDDLDIYVTTASNAEE

SSWGTYCPGMDPPPPPEYITCLGDLYSVAWMEDSESHNLKRETVEQQYQQVKERTSNFNT

YNAGSHVMEYGNKSIKSEKLYLYQGFDPATENMPPSENHLKPHMDVVNQRDADLLFLWER

YKRLDGGAKKKSELFKLITDTMLHRKHMDDSIDIIGAFLFGPENGPSILKSVRDRGLPLA

DDWDCLKSMVRLFEAHCGSLTQYGMKHTRAFANICNSRVSSADMEDACMAACRGHDFAGWSPLNRGYSA

>AWD84471.1_PxAEP1

RNVLRLPSEVSRFFGADESVRNKDDDSVGTRWAILLA

GSNGYWNYRHQADICHAYQLLKKGGLKDENIVVFMYDDIANNEENPRPGIIINSPHGEDV

YKGVPKDYTGDDVTVDNFLAVILGNKAALSGGSGKVVNSGPNDHIFIYYSDHGGPGVLGM

PTDPYLYANDLIDVLKKKHASGTYKSLVFYLEACESGSIFEGLLPEGLNIYATTASNAEE

SSWGTYCPGEYPSPPIEYETCLGDLYSIAWMEDSDIHNLRTESLKQQYHLVKDRTANGNP

FYGSHVMQYGDLNLSKNPLFVYMGTNPANDNYTFGADNSLRVSKVVNQRDADLLHFWYKFRKAPEGSARKFEAQKQLNEAISHRMHLDNSIALVGKLLFGIKNVPEVLSSVRPAGQPLVD

DWDCLKSYVRTFETHCGSLSQYGMKHMRSIANICNAGIKMEQMVEASAQACPRVPSNTWSSLHRGFSA

>NP_176458.1_AtLEG_beta

RGRFEPKILMPTEEANPADQDEDGVGTRWAVLVAGSSGYGNYRHQAD

VCHAYQILRKGGLKEENIVVLMYDDIANHPLNPRPGTLINHPDGDDVYAGVPKDYTGSSVTAANFYAVLLGDQKAVKGGSGKVIASKPNDHIFVYYADHGGPGVLGMPNTPHIYAADFIETLKKKHASGTYKEMVIYVEA

CESGSIFEGIMPKDLNIYVTTASNAQESSYGTYCPGMNPSPPSEYITCLGDLYSVAWMEDSETHNLKKET

IKQQYHTVKMRTSNYNTYSGGSHVMEYGNNSIKSEKLYLYQGFDPATVNLPLNELPVKSKIGVVNQRDAD

LLFLWHMYRTSEDGSRKKDDTLKELTETTRHRKHLDASVELIATILFGPTMNVLNLVREPGLPLVDDWEC

LKSMVRVFEEHCGSLTQYGMKHMRAFANVCNNGVSKELMEEASTAACGGYSEARYTVHPSILGYSA

>NP_195020.1_AtLEG_gamma

ARSGPDDVIKLPSQASRFFRPAENDDDSNSGTRW

AVLVAGSSGYWNYRHQADICHAYQLLRKGGLKEENIVVFMYDDIANNYENPRPGTIINSP

HGKDVYQGVPKDYTGDDVNVDNLFAVILGDKTAVKGGSGKVVDSGPNDHIFIFYSDHGGP

GVLGMPTSPYLYANDLNDVLKKKHALGTYKSLVFYLEACESGSIFEGLLPEGLNIYATTA

SNAEESSWGTYCPGEEPSPPPEYETCLGDLYSVAWMEDSGMHNLQTETLHQQYELVKRRT

APVGYSYGSHVMQYGDVGISKDNLDLYMGTNPANDNFTFADANSLKPPSRVTNQRDADLV

HFWEKYRKAPEGSARKTEAQKQVLEAMSHRLHIDNSVILVGKILFGISRGPEVLNKVRSA

GQPLVDDWNCLKNQVRAFERHCGSLSQYGIKHMRSFANICNAGIQMEQMEEAASQACTTL

PTGPWSSLNRGFSA

>AWD84473.1_HeAEP1

QATRSSRFDPGILMPTEKQPEAADDDEIGTRWAVLVAGSNGYGN

YRHQADVCHAYQLLRKGGLKEENIVVFMYDDIAKNELNPRPGVIINHPQGEDVYHGVPKDYTGQHVTAHN

LYAVLLGNKTAVKGGSGKVVDSKPNDRIFLYYSDHGGPGVLGMPNMPYLYAMDFLEVLKKKHASKSYREM

VIYVEACESGSIFEGIMPEDLSIYVTTASNAQENSWGTYCPGEDPGAPPEFTTCLGDLYSVAWMEDSETH

NLKKETIKDQYKTVKARALRANTYHEGSHVMEYGNRSIKGEKLYLYQGFDPATVNLPPNNGLIDKPMEVV

NQRDAELIFLWQMYKRSEDKSEKKTEILNQIKETMRHRNHLDGSMELIGTLLFGPRKGSSILHSVREPGL

PLVDDWKCLKSMVRLFETHCGSLTQYGMKHMRAFANICNYGISEASMEEASSAACSGHDVGQWHPSVQGY

SA

>AWD84470.1_HeAEP2

GGRDIVDDVLLLPSDVSNFFHNNNKQTNNDDNNKDDDSTGTRWAVLIAG

SNGYWNYRHQADVCHAYQLLKKGGLKDENIIVFMYDDIAHNFENPRPGIIINNPKGEDVYKGVPKDYTGE

DVNAGNFYAVILGNKTALTGGSGKVVNSGPNDHIFIYYTDHGGPGILGMPTSPYIYADKLVDVLKQKHAS

GTYKSLVFYLEACESGSIFEGLLPEGLNIYATTASNAIESSWGTYCPGDHISPPPEYETCLGDLYSVAWM

EDSDVHNLRTETLHQQYELVKQRTAHSNGYGSHVMQYGDVPLSKENLFLYMGTNPANENFTFVDDNSLSL

PSKAVNQHDADLLHFWHKYHRAREGSSRKLEAQKEFVEMMSHRMHLDHSVKFIGKLLFGMDEASEVLNAV

RPAGNPLTDDWDCLRTLVRTFETHCGSLSQYGMKHMRSFANLCNAGISKEQMAEASSQACASFPSNPWSS

LRKGFSA

>AWD84474.1_HeAEP3

RPDDFLRLPSEAAKSFLHNDDDSVGTRWAVLIAGSKGWQNYRHQADVC

HAYQILKKGGLKDENIIVFMYDDIAYNESNPRPGIVINKPKGEDVYKGVPKDYTGENVNAVNFLAVLLAN

RSALTGGSGKVLDSGPNDRIFIYYTDHGAPVTIGMPSKPYLVAKDLVDTLKKKHAAGTYKSMVFYIESCE

SGSMFDGLLPEDANIYGMTATNSTEGSWVTYCPGQTDDYPEDDEYDVCFGDLWSVAWLEDCDAHNLRTET

LDQQYEVVKKKIEYAHIPAQYGNVSLAKDSLFVYMGTDPANDNKTFVEENTLRRPLKAVHSRDADLLHFW

HKYHKAPEGTSRKIDAQKQLVEVLSHRTHVDNSIKLVGELLFGVGKASEVLNTIRPAGQPLVDDWDCLKT

MVRTFETHCGSLSEYGMKHMRSFANMCNAGVQKEQMAVAAGQACVTFPSNPWSSLDEGFSV

>AWD84472.1_PxAEP3a

EGRNVLKLPSEASRFFDKGDDDSVGTRWAVLLAGSNGYWNY

RHQADVCHAYQLLRKGGLKDENIIVFMYDDIAYNEENPRKGVIINSPAGEDVYKGVPKDY

TGDDVNVDNFLAVLLGNKTALTGGSGKVVDSGPNDHIFVFYSDHGGPGVLGMPTNPYLYA

SDLIGALKKKHASGTYKSLVLYIEACESGSIFEGLLPEGLNVYATTASNAVESSWGTYCP

GENPSPPPEYETCLGDLYAVSWMEDSEKHNLQTESLRQQYHLVKRRTANGNSAYGSHVMQ

FGDLKLSVDSLSMYMGTDPANDNSTFVDDNSLGASSKAVNQRDADLLHFWDKFLKAPEGS

ARKVEAQKQFTEAMSHRMHLDNSMALVGKLLFGIQKGPEVLKRVRSDGQPLVDDWACLKS

FVRTFETHCGSLSQYGMKHMRSIANICNAGIKMEQMVEASSQACPSVPSNTWSSLHRGFS

A

>AWD84475.1_PxAEP2

RDVLKLPSEASKFFSEKYGDGSVEGTRWGVLLAGSRGYWNY

RHQADVCHAYQLLKKGGLKDENIIVFMYDDIANNYENPRPGIIINSPDGEDVYKGVPKDY

TGHNVTVNNFLAVILGDKAALTGGSGKVVESGPNDHIFIFYSDHGGPGVLGMPTYPNLYA

DELIDALKRKHASGTYKSLVFYIEACESGSIFEGLLPEGLNIYATTASNAEEDSWATYCP

GDNQSPPPEYQTCLGDLYSVSWMEDSEKHDLQTETLGMQYELVRRRTANSFPFASSHVMQ

YGDLKLRDDPISLYMGTNPANYTYSFLDENSLLSSKPVNQRDADLLHFWEKFLKARQGSA

RKLEAQKQLTEAMTHRMHIDDSITLVGKLLFGIEKGTEELTRVRPSGEPLVDDWDCLKSF

VGTFETYCGSLSQYGLKYMRAIANICNASIKVEQMAKASAQACVDVPSNSWDSLDEGFSA

>AIZ09514.1_HaAEP1

AAGRESSGGQKWRWGWDPLIRSPVDAEQEVDEQMTNGTKWAVLVAGSKGYGNYRHQADV

CHAYQVLKKGGLKDENIVVFMYDDIAKSEMNPRPGIIINSPKGEDVYAGVPKDYTGKNVTVDNLSAVLLGDRSAVKGGSG

KVVDSKPEDRIFLFYSNHGGPGVLGMPNEPHLVAKDLVDVLKKKHAMGTYKEMVIYLEACESGSIFEGILPEDLNIYATT

ASGAQENSYGTYCPGTEPSPPPEYITCLGDLYSVAWMEDSETHNLKKESLEQQFNKVKKRTSNSNTYNTGSHVMEYGSKD

IKPEKVYLYLGFDPATVNLPANQIHFDKLDGVNQRDADLIFLWQRYKKSSESTRPEILREITETLTHRGHLDSSIDMIGV

LLFGPQNGRSTLHSARAPGLPLVDDWECFKSTARLFEKHCGLLTQYGMKHMRAFANICNSSVEKSKVEEVFIATCGGKNI

GPYGTFGAYSV

>VyPAL2

GGLDVDSLQLPSEAAKFFHNDNSTNDDDSIGTRWAVLIAGSKGYHNYRHQADVCHMYQI

LRKGGVKDENIIVFMYDDIAYNESNPFPGIIINKPGGENVYKGVPKDYTGEDINNVNFLAAILGNKSAIIGGSGKVLDTS

PNDHIFIYYADHGAPGKIGMPSKPYLYADDLVDTLKQKAATGTYKSMVFYVEACNAGSMFEGLLPEGTNIYAMAASNSTE

GSWITYCPGTPDFPPEFDVCLGDLWSITFLEDCDAHNLRTETVHQQFELVKKKIAYASTVSQYGDIPISKDSLSVYMGTD

PANDNRTFVDENSLRPPLKVIHQHDADLYHIWCKYNMAPEGSSKKIEAQKQLLELMSHRAHVDNSITLIGKLLFGVNKAS

KVLNTVRPVGQPLVDDWQCLKAMIRTFETHCGSLSEYGMKHTLSFANMCNAGIQKEQLAEAAAQACVTFPSNPYSSLAEG

FSA

>NP_001310660.1_RcAEP1

SRLNPFEPGILMPTEEAEPVQVDDDDQLGTRWAVLVAGS

MGFGNYRHQADVCHAYQLLRKGGLKEENIIVFMYDDIAKNELNPRPGVIINHPQGEDVYAGVPKDYTGEH

VTAKNLYAVLLGDKSAVQGGSGKVVDSKPNDRIFLYYSDHGGPGVLGMPNLPYLYAMDFIEVLKKKHAAG

GYKKMVIYVEACESGSIFEGIMPKDVDIYVTTASNAQESSWGTYCPGMEPSPPPEFTTCLGDLYSVAWME

DSESHNLKKETVKQQYSSVKARTSNYNTYAAGSHVMQYGNQSIKADKLYLFQGFDPASVNFPPNNAHLNA

PMEVVNQRDAELHFMWQLYKRSENGSEKKKEILQQIKDAIKHRSHLDSSMQLIGDLLFGPKKASAILKSV

REPGSPLVDDWGCLKSMVRVFETCCGSLTQYGMKHMRTFANICNAGVSHTSMEEACNAACSGHDAGQWHP

TNQGYSA

>BAA76744.1_VmPE-1

GRDEILRMPSEASRFFQAPATDENDEGTRWAVLIAGSNGYWNYRHQSDVCH

AYQLLTKGGLKEENIVVFMYDDIAFNEENPRPGVIINSPHGNDVYKGVPKDYVGEDVTVNNFFAAILGNK

SALTGGSGKVVNSGPNDHIFIYYSDHGGPGVLGMPTSPYMYASDLIEVLKKKHASGTYKSLAFYLEGCES

GSIFGGLLPEGLNIYATTAANAEESSWGTYCPGDNPSPPPEYETCLGDLYSVAWMEDSDIHNLRTETLHQ

QFELVKQRTMNGNSAYGSHVMQYGDVGLSKNNVSLYLGTNPANDNFPFREKNSLVPPSKAVNQRDADLVH

FWDKFPKAPLGSSRKSVAQKQILEAMSHRMHIDDSVTLIGKLLFGIEEGPELLSSVRPAGQPLVDDWDCL

KTLVRTFETHCGSLSQYGMKHMRSFANLCNAGIRKEQMAEASAQACVSIPATPWSSLSSGFSA

>BAA76745.1_VmPE-1A

RRDHVGDFLRLPSDSGNDDNVQGTRWAILFAGSNGYWNYRHQADICH

AYQILRKGGLKEENIIVFMYDDIAFNWDNPRPGVIINKPDGDDVYEGVPKDYTGEDATAHNFYSALLGDK

SALTGGSGKVVSSGPDDRIFIFYSDHGGPGVLGTPAGPYIYASDLVEVLKKKHASGTYKNLVFYLEACEA

GSIFEGLLPEDINIYATTASNAEESSWGTYCPGEYPSPPPEYSTCLGDLYSVAWMEDSDRHNLRTESLHQ

QYKVVKDRTLSGGWYGSHVMQYGDVEFSKDALFLYLGTDPANDNLTFVDE NSLWSSSTAVNQRDADLVHF

WHKFRKAPEGSPKKNEARKQVLEVMSHRMHIDDSVKLVGKLLFGFEKAPEVLNAVRPAGSALVDDWACLK

TMVRTFETHCGSLSQYGMKHMSPFANICNVGIKKEQMAEASAQACVTVPASSWSSLQRGFSA

>BAA06596.1_CeAEP

RLNRREWDSVIQLPTEPVDDEVGTRWAVLVAGSNGYGNYRHQADVCHAYQLLIKG

GVKEENIVVFMYDDIAYNAMNPRPGVIINHPQGPDVYAGVPKDYTGEDVTPENLYAVILGDKSKVKGGSG

KVINSNPEDRIFIFYSDHGGPGVLGMPNAPFVYAMDFIDVLKKKHASGGYKEMVIYIEACESGSIFEGIM

PKDLNIYVTTASNAQENSFGTYCPGMNPPPPEEYVTCLGDLYSVSWMEDSETHNLKRETVQQQYQSVRKR

TSNSNSYRFGSHVMQYGDTNITAEKLYLYHGFDPATVNFPPHNGNLEAKMEVVNQRDAELLFMWQMYQRS

NHQPEKKTHILEQITETVKHRNHLDGSVELIGVLLYGPGKSSSVLHSVRAPGLPLVDDWTCLKSMVRVFE

THCGSLTQYGMKHMRAFGNVCNSGVSKASMEEACKAACGGYDAGLLYPSNTGYSA

>CAB17078.1_PvAEP

GRDLVGDFLRLPSDSGNGDNVHGTRWAILFAGSSGYWNYRHQADIC

HAYQLLRKGGLKDENIIVFMYDDIAFNSENPRRGVIINSPNGDEVYKGVPKDYTGEDVTAHNFYAALLGD

KSKLTGGSGKVVNSGPNDHIFIFYSDHGGPGVLGSPAGPYIYASDLNEVLKKKHASGTYKNLVFYLEACE

SGSIFEGLLPEDINVYATTASNADESSWGTYCPGEDPSPPPEYSTCLGDLYSVAWMEDSDRHNLRTETLH

QQYKLVKERTISGGLYYGSHVMQYGDVGLSKDILFHYLGTDPANENLTFVDENSLWSSSKAVNQRDADLV

HFWDKFRKAPEGSPKKNEARKQVLEVMSHRMHIDDSVELVGKLLFGIEKAPELLNAVRPAGSALVDDWDC

LKTMVRTFETHCGSLSQYGMKHMRSFANMCNVGIKKEQMREASAQACVTIPANPWSSLQRGFSA

>CAB17079.1_PvAEP

KEQDSVIKLPTQEVDAESDEVGTRWAVLVAGSNGYG

NYRHQADVCHAYQLLIKGGVKEENIVVFMYDDIATHELNPRPGVIINNPQGPDVYAGVPKDYTGESVTSH

NFFAVLLGDKSKVKGGSGKVINSKPEDRIFVYYSDHGGPGVLGMPNMPYLYAMDFIDVLKKKHASGGYKE

MVIYVEACESGSIFEGIMPKDLNIYVTTASNAQENSWGTYCPGMYPPPPPEYITCLGDLYSVAWMEDSES

HNLKKESVEQQYQSVKQRTSNFEAYAMGSHVMQYGDANMTAEKLYLYHGFDPATVNFPPHNGRLKSKMEV

VNQRDAELLFMWQVYQRSNHLPEKKTDILKQIEEIVKHRKHLDGSVELIGVLLYGPEKASSVLRSVRTTG

LPLVDDWTCLKSMVRVYETHCGSLTQYGMKHMRAFANICNSGVSETSMEKACVAACGGYHAGLLHPSNTG

YSA

>AAK15049.1_VrAEP

GRDLVGDFLRLPSDSGNDDNVKGTRWAILFAGSNGYWNYRHQADIC

HAYQILRKGGLKEENIIVFMYDDIAFNWDNPRPGVIINKPDGDDVYEGVPKDYTGEDATAHNFYSALLGD

KSALTGGSGKVVNSGPDDRIFIFYSDHGGPGVLGTPAGPYIYASDLVEVLKKKHASGTYKNLVFYLEACE

AGSIFEGLLPEDINIYATTASNAEESSWGTYCPGEYPSPPPEYSTCLGDLYSVAWMEDSDRHNLRTESLH

QQYKVVKDRTLSGGWYGSHVMQYGDVEFSKDTLFLYLGTDPANDNLTFVDENSLWSSSTAVNQRDADLVH

FWHKFRKAPEGSPKKNEARKQVLEVMSHRMHIDDSVKLVGKLLFGFEKAPEVLNAVRPAGSALVDDWACL

KTMVRTFETHCGSLSQYGMKHMRSFANICNVGIKKEQMAEASAQACVTVPASSWSSLQRGFSA

>XP_017410149.1_VaVPE

AGARPNRRELDSFIKLPTEPEDAESDELGTRWAVLVAGS

NGFGNYRHQADVCHAYQLLIKGGVKKENIVVFMYDDIAHNKQNPRPGVIINHPRGPDVYAGVPKDYTGEN

VNSRNFFAVLLGDKKKVKGGSGKVINSKAEDRIFIYYSDHGGPGVLGMPNLPYLYAMDFVDVLKKKHASR

GYKKMVIYVEACESGSIFEGIMPKNLNIYVTTASNAQENSWGTYCPGMNPSPPPEYITCLGDLYSVAWME

DSESHNLKKETVAQQYQSVKHRTSNLQNYGMGSHVMEYGDANITAEKLYLYQGFNPATVNFPPFNARPEA

KMEVVNQRDADLFFMWQMYQRSNQQPEKKTDILKQITETVKHRKHLDGSVELIGVLLYGPGKASSVLQSV

RTPGLPLVDDWTCLKSMVRVFETHCGSLTQYGMKHMRAFANICNSGVSETSMENACVAACGGYHAGQLHP

SNTGYSA

>XP_017430387.1_VaVPE

RRDLVGDFLRLPSDSGNDDNVQGTRWAILFAGSNGYWNYRHQADICH

AYQILRKGGLKEENIIVFMYDDIAFNWDNPRPGVIINKPDGGDVYEGVPKDYTGEDATAHNFYAALLGDK

SALTGGSGKVVNSGPDDRIFIFYSDHGGPGVLGTPAGPYIYASDLVEVLKKKHASGTYKNLVFYLEACEA

GSIFEGLLPEDINIYATTASNAEESSWGTYCPGEYPSPPPEYSTCLGDLYSVAWMEDSDRHNLRTESLHQ

QYKVVKDRTLAGGYYGSHVMQYGDVGFNKDNLFLYLGTDPANDNLTFVDENSLWSSSTAVNQRDADLVHF

WHKFRKAPEGSPKKNEARKQVLEVMSHRMHIDDSVKLVGKLLFGFEKAPEVLNAVRPAGSALVDDWACLK

TMVRTFETHCGSLSQYGMKHMRSFANICNVGIKKEQMAEASAQACVTIPASSWSSLQRGFSA

>AEO79971.1_AdiVPE

SGRRDIVGGTLRLPSEAISRFFHEPENEGTKWAVLLAGSNGYWNYRHQA

DICHAYQLLRSGGVKEENIIVFMFDDIAYSEENPRPGVIINKPDGGDVYKGVPKDYTGKDVNVNNFFAAL

LGNKSALTGGSGKVVDSGPNDHIFVFYSDHGGPGILGMPVGPYLYANDLNEVLKKKHASGGYKSLVFYLE

ACESGSIFEGLLPEDINIYATTASNAVESSWGTYCPGEDPSPPPEYSTCLGDLYSISWMEDSDTHNLRTE

TLHQQYKLVKDRTLNGNAYYGSHAMQYGDVGISENLLFQYLGTNPANDNYTFVDENSLRTPSKAVNQRDA

DLIHFWEKFRKAPEGSSSKITAQKQVVEVMSHRMHIDNSVKLIGNLLFGTEKGPELLSAVRPAGKPLVDD

WDCLKNMVRTFETHCGSLSQYGMKHMRTFANICNAGIHKDQMDEATAQACVSIPSNPWSSLERGFSA

>XP_015971413.1_AduVPE1

LEGRHVSNNFHPLVSNDDSEGTRWAVLVAGSNGFGNYRHQADICHA

YQIMKNGGLKDENIIVFMSDDIANNEENPRPGVIINHPNGSDVYKGVPKDYTGNYTSAENLYAVISGNQS

AITGGSGKMLNSGPNDTIFIYYADHGGPGIIGMPVGDFVVANDFIDVLKSKHAANAYKKMVIYLEACESG

SMFEGILPENINIYVTTASNANESSWGCYCPGDPTYPPPPEFQTCLGDSYSVLWMEDSDKNDRTKETLQQ

QYETVREKTLDWEAKGSHVMQYGNKNFTDDFLVTYIGATGVSTSTNAYPSSATPTKFVDQRDALLHYLSH

KFKNAPEGSKERMEAQKGLIEEIAQREHVDNSVKAIADHLFGEEYRASVISSVRPSGQPLVDDWDCFKNY

IKIYESYCGALSTYGRKYTRAIANICNAGVSQEKMVVASSLACP

>XP_015972214.1_AduVPE2

SGRRDIVGGTLRLPSEAISRFFHEPENEGTKWAVLLAGSNGYWNYRHQA

DICHAYQLLRSGGVKEENIIVFMYDDIAYSEENPRPGVIINKPDGGDVYKGVPKDYTGKDVNVNNFFAAL

LGNKSALTGGSGKVVDSGPNDHIFVFYSDHGGPGILGMPVGPYLYANDLNEVLKKKHASGGYKSLVFYLE

ACESGSIFEGLLPEDINIYATTASNAVESSWGTYCPGEDPSPPPEYSTCLGDLYSISWMEDSDTHNLRTE

TLHQQYKLVKDRTLNGNAYYGSHVMQYGDVGISENLLFQYLGTNPANDNYTFVDENSLRTPSKAVNQRDA

DLIHFWEKFRKAPEGSSSKITAQKQVVEVMSHRMHIDNSVKLIGNLLFGTEKGPELLSAVRPAGKPLVDD

WDCLKNMVRTFETHCGSLSQYGMKHMRTFANICNAGIHKDQMDEATAQACVSIPSNPWSSLERGFSA

>XP_015935151.1_AduVPE3

VRDPIGEVIRLPSEASRFFKAPSDDDDNVEEGTRWAILIAGSNGYWNYRHQ

ADVCHAYQLLRKGGLKEENIIVFMYDDIAFNEENPRPGVIINSPHGDDVYKGVPKDYVGGDVTVNNFFAA

ILGNKSALTGGSGKVVDSGPNDHIFIYYSDHGGPGVLGMPTSPYLYASDLIEVLKKKHASGTYKSMVFYL

EACESGSIFEGLLPEGLNIYATTAANAEESSWGTYCPGENPSPPPEFGTCLGDLYSVAWMEDSAIHNLRT

ETLHQQYQLVKERTSNGNSMYGSHVMQYGDIGISSNNLFLYLGTNPANENFTVMDNNSLKLPSKTAVNQR

DADLVHFWDKYLKAPEGSPRKAAAEKQVMEAMSHRMHIDSSVKLIGKLLFGIEKGQEILSSVRPSGEPLV

DDWDCLKSLVTTFETHCGSLSQYGMKHMRSFANFCNAGIRREQLADASAHACTSVPSNPWSSLHRGFSA

>XP_015971429.2_AduVPE4

ITMLDCRVTNLFHPLSNEDGEEGKKWAVLVAGSKGYFNYRHQADI

CHAYQIMKNGGLKDENIIVFMYDDIANNELNPRPGVIINHPNGSDVYKGVPKDYTGNYTTAENLYAVISG

NRSAITGGSGKVVDSGPNDTIFIFYADHGGPGIVSMPVGDDVVAYDFIDVLKRKHAANAYKKMVIYLEAC

ESGSMFEGILPENINIYVTTASNANESSWGCYCPGDPTYPPPPQFLTCLGDSYSVLWMEDSDESDRTKET

LQQQYETVREKTLSWAGKGSHVMQYGNKNFTNDYLVTYIGATGVSETSNAYSSSIPTLLVNQRDALLLYL

THKLENAPDGSSEKVEAQKMLVNEIAEREHVDNVVKKIGDLLFGEDNSATLMASYRPSGQPLVDDWDCFK

NYIKIYERYCGTLSTYGKKYTRVFANMCNAGISEDKMVAASSQACP

>XP_016162949.1_AiVPE2

HRDIVGGTLRLPSEAISRFFHEPENEGTRWAVLLAGSNGYWNYRHQA

DICHAYQLLRSGGVKEENIIVFMYDDIAYSEENPRPGVIINKPDGGDVYKGVPKDYTGKDVNVNNFFAAL

LGNKSALTGGSGKVVDSGPNDHIFVFYSDHGGPGILGMPVGPYLYANDLNEVLKKKHASGGYQSLVFYLE

ACESGSIFEGLLPEDINIYATTASNAVESSWGTYCPGEDPSPPAEYSTCLGDLYSISWMEDSDIHNLRTE

TLHQQYKLVKDRTLNGNAYYGSHVMQYGDVGISENLLFQYLGTNPANDNYTFVDENSLRTPSKAVNQRDA

DLIHFWEKFRKAPEGSSSKITAQKQVVEVMSHRMHIDNSVKLIGNLLFGTEKGPELLSAVRPAGKPLVDD

WDCLKNMVRTFETHCGSLSQYGMKHMRTFANICNAGIHKDQMDEATAQACVSIPSNPWSSLERGFSA

>XP_016163867.1_AiVPE1

VRDPDREVIRLPSEASRFFKAPSDDGDNVEEGTRWAILIAGSNGYWNYRHQ

ADVCHAYQLLRKGGLKEENIIVFMYDDIAFNEENPRPGVIINSPHGDDVYKGVPKDYVGGDVTVNNFFAA

ILGNKSALTGGSGKVVDSGPNDHIFIYYSDHGGPGVLGMPTSPYLYASDLIEVLKKKHASGTYKSMVFYL

EACESGSIFEGLLPEGLNIYATTAANAEESSWGTYCPGENPSPPPEFGTCLGDLYSVAWMEDSAIHNLHT

ETLHQQYQLVKERTFNGNSMYGSHVMQYGDIGISSNNLFLYLGTNPANENFTFVDNNSLKLPSKTAVNQR

DADLVHFWDKYLKAPEGSPRKAAAEKQVMEVMSHRMHIDSSVKLIGKLLFGIEKGQEILSSVRPSGEPLV

DDWDCLKSLVTTFETHCGSLSQYGMKHMRSFANFCNAGIRREQLADASAHACTSVPSNPWSSLHGGFSA

>AES73372.1_MtVPE1

GDDFLRLPSQASRFFQSDDDNNEGTKWAILIAGSNGYWNYRHQSDVC

HAYQVLRKGGLKEENIIVFMYDDIADNQENPRPGVIINSPHGDDVYKGVPKDYTGDDVNVNNFFAALLGN

KSALTGGSGKVVDSGPNDHIFIYYSDHGGPGVLGMPTGPFMYATDLIEVLKKKHASETYKSLVFYLEACE

SGSIFEGLLPEGLNIYATTAANAEESSWGTYCPGENPSPPPEYETCLGDLYSVAWMEDSDIHNLQTETLH

QQYELVKERTSNGNSIYGSHVMQFGDIGLSRDSLFLYLGSNPANENFTFMGRNSLVPPSKTVNQRDADLI

HFWDKFRKAPQGSPRKVAAQKQVLEAMSHRMHIDESIKLVGKLLFGMKKGPEVLASVRPAGQPVVDDWDC

LKSLVRTFETYCGSLSQYGMKHMRSFANFCNAGIHSEQMAEASAQACINIPANPWSSLHGGFSA

>AES78483.1_MtVPE2

EGESTTGKKWAFLVAGSNGYVNYRHQADICHAYQILKKGGLKDEN

IVVFMYDDIAYNPQNPRRGVLINHPNGSDVYNGVPKDYIGDYGNLENFLAVLSGNKSATKGGSGKVLDTG

PDDTIFIFYTDHGSPGSIGIPDGGLLYANDFVDALKKKHDAKSYKKMVIYMEACEAGSMFEGLLPNDINI

YVTTASNKSENSYGFYCPNSYLPPPPEYDICLGDLYSISWMEDSEKNDMTKEILKEQYETVRQRTLLSHV

LQYGDLNISNDTLITYIGADPTNVNDNFNVTSTTNVFSFDDFKSPNPTRNFGQRDAHLIYLKTKLGRASS

GSEDKLKAQKELEVEIARRKHVDNNVHQISDLLFGEEKGSIVMVHVRASGQPLVDNWDCLKTLVKTYESH

CGTLSSYGRKYLRAFANMCNNGITVKQMVAASLQACLEKN

>AES79969.2_MtVPE3

KGIRPMVDEHNEQGNFEVVRKKWALLVAGSKDYPNYRHQANICHAY

HVLKNGGLQDENIIVFMYDDIAYHKENPRQGVIINRPDGPNVYPGVPKDYTGNNTNAENFFAVLNGNLSG

ITGGSGKVLNSGPIDTVFIYYSGHGYPGLIGMADQGIVYAKDFVDALKKKHASNSYKKMVIYVEACYSAS

LFEGLLPNNMSIYVATSTNARELGYGFYCPDSKNLSSTEYTICLGDTFGISWMEDSDKNDRTYETLQQQY

FTVRDRVISHRNFASHVTQLGDLNISNDFLVTYISAAPHNNVSDNYNLSNTTSFVSQDDAYLLHLRLKLK

KALNGSEDKLKVQNELDAEIAHRKHVDNNIDLIENILFGEKKKSSAMMFDFRSIDQPLVDDWNCLKILFK

TYESQCGILSTYGRKYSKAFAYMCNIGISEKQMIAVVSQVCPGI

>AES80307.2_MtVPE4

KGVVRPMVHKHDEQGNFEVVGKKWALLVAGSKGYSNYRHQSNICHA

YHILKSGGLQDENIIVFMYDDIAYHNENPRPGVIINRPDGPNVYPGVPKDYTGNNTNAENFFAVLNGNLS

GITGGSGKVLNSDPNDTIFIYYSGHGYPGLIGMADQSLVYAKDLVDALKKKHASNSYKKMVIYVEACYSA

SLFEGLLPNNISIYVTTSANARELGYGFYCPGSINLSSTEYTTCLGDTFGISWMEDSDKNESTNETLQQQ

YVTVRDRTITSHVTQLGDLNISNDFLDTYIGSAPLNNVSDNYNLTNTTSVYSFEPFNTSTSLVNQDDAYL

LHLKLKLEKAVDGSKDKLKAQNELDAEIAHRKHVDHNIHLIGNILFGEKKSSIMMSDLRSAGQPLIDDWN

CLKILFKTYESHCGILLSTYGRKYSRVFAYMCNIGIFEKQTISAVSQVCSRIHHSS

>AES90963.2_MtVPE5

RPNHLEWDPVIRLPGEVVDDAEVDEVGTRWAVLVAGSSGY

GNYRHQADVCHAYQLLIKGGVKEENIVVFMYDDIANNELNPRPGVIINHPQGPNVYVGVPKDYTGDNVTA

ENLYAVILGDKSKVKGGSGKVINSKSEDRIFIYYSDHGGPGVLGMPNMPYVYAMDFIDVLKKKHASGGYK

KMVVYIEACESGSMFEGVMPKDLNVYVTTASNAQESSWGTYCPGVEPAPPPEYITCLGDLYSVAWMEDSE

SHNLKRETVKQQYKSVKERTSNYNNYALGSHVMQYGDTNITDEKLYLYQGFDPATVNLPPHNDKLESKME

VVNQRDAEILFMWEMYKRLDHQTEKKREILEKIAETVKHRNHLDGSVELIGVLLFGPTKGSSVLQAVRAT

GLPLVDDWECLKSRVRLFETHCGSLTQYGMKHMRAFANICNSGISEDSMEKACMVACGGYKLELLHPSNR

VYSA

>KEH39941.1_MtVPE6

SRDLPGDYIRLPSQSQASRFFHEPENDDNDQGTRWAILLAGSNGYW

NYRHQADVCHAYQLLRKGGLKEENIIVFMYDDIASNVENPRPGVIINKPDGGDVYEGVPKDYTGAEVHAD

NFYAALLGNKSALTGGSGKVVDSGPNDHIFVYYTDHGGPGVLGMPVGPYLYASDLNEVLKKKHASGSYKS

LVFYLEACESGSIFEGLLPEDINIYATTASNAVESSWGTYCPGEYPPPPPEYSTCLGDLYSIAWMEDSDI

HNLRTESLHQQYKLVKDRTINGYYGSHVMEYGDVGLSNNHLFLYLGTNPANDNISFVDESSLKLRSPSTA

VNQRDADLIHFWDKFRKAPEGSLRKNEAQKEVLEAMSHRMHVDNSVKLIGKLLFGIEKGTELLDNVRPAG

SPLVDNWDCLKTMVKTFETHCGSLSQYGMKHMRSFANICNAGIQTEQMAEASAQACASIPANPWSSLQRG

FSA

>XP_003622265.2_MtVPE7

MNVGSSRKQFSSKIHLNSCGRYTRPAPNEDRSTTGKKWAFLVAGSNGYVNYRHQADICHAYQILKKGGLK

DENIVVFMYDDIAYNPQNPRRGVLINHPNGSDVYNGVPKDYIGDYGNLENFLAVLSGNKSATKGGSGKVL

DTGPDDTIFIFYTDHGSPGSIGIPDGGLLYANDFVDALKKKHDAKSYKKMVIYMEACEAGSMFEGLLPND

INIYVTTASNKSENSYGFYCPNSYLPPPPEYDICLGDLYSISWMEDSEKNDMTKEILKEQYETVRQRTLL

SHVLQYGDLNISNDTLITYIGADPTNVNDNFNVTSTTNVFSFDDFKSPNPTRNFGQRDAHLIYLKTKLGR

ASSGSEDKLKAQKELEVEIARRKHVDNNVHQISDLLFGEEKGSIVMVHVRASGQPLVDNWDCLKTLVKTY

ESHCGTLSSYGRKYLRAFANMCNNGITVKQMVAASLQACLEKN

>XP_004499587.1_Ca_VPE1

ARPLNNQLQEGTGVKWAFLVAGSRGYGNYRHQADVCHAYQVLK

SGGLKDENIIVMMYDDIADNVENPFRGTIINQPDGPDVYQGVPKDYTGTDANMFNFYAVLRGDKSATDGG

SGKVLSSKPEDTVFIFIVSHGNKGIMGLPDDNIIYADMLVDSLKINYKKMVIYIESCKAGSMLEGLLQDD

VNIYATTSSRADEDSAAFYCPRGIMPPSPKYTTCLGDLYSIAWLEYSDQYDRANKTLLDQYDVTWFRTLF

TGEEYSAHVMPYGNITMHDDLLETYFGRAKPIGANDNYHFNRTTTHEHSNKRFNTTTRLVSQQDAHLNYL

KLKLEKAPDGSLDKSKAQIELDDEISHRKHDDQSVYLIWKLLFGEDTTSTMMANLRSAGQPLVDDWDCLR

MLKNTYEHHCGVLSHYGWKYMQAFANMCNNGISKKQMIAAASQVCPKKNS

>XP_027190454.1_CaVPE2

LRPMSNQEESLTGKKWALLVAGSSGYENYRHQADICHAYQILKK

GGLKDENIIVFMYDDIAYNVQNPRPGVLINHPNGPDVYYGVPKDIYQATNHVTLQGMTLPKQIGPFATLT

FSLPNGGLLYGNDFVDALKKKHAAKSYQKMVIYMEACDAGSMFEGLLPNDINIYVTTASNTNENSYAFYC

PDLYPAPPPEYNICLGDTYSIAWMEDSSRNDMTKETLKQQYETVRRRTLINNADQYSHVVEYGDMKINND

SLVTYIGADPANVIDDYGSYLNVVPTNVNDNFNVTSTTMTPTKDVGQRDARLIHLMHKLQSASDGSEDKL

KAQKELEVEIAHREYVDKSVHMISSILFGEEKGSTIIVDVRASGQPLVDDWDCLKTFMKTYESHCGTLSS

YGRKYSRAFANMCNAGISEKQMIAASTQACRERI

>XP_027918860.1_VuAEP1

AGARPNRREQGSIIKLPSEAVDADSDEVGTRWAVLVAGS

NGYGNYRHQADVCHAYQLLIKGGVKEENIVVFMYDDIANHDLNPRPGIIINHPQGPDVYAGVPKDYTGEN

VTSRNFYAVLLGDKSKVKGGSGKVINSKPEDRIFIYYSDHGGPGVLGMPNMPYLYAMDFMDVLKKKHASG

GYKKMVIYVEACESGSIFEGIIPKDLNIFVTTASNAQENSWGTYCPGMIPSPPPEYITCLGDLYSVAWME

DSESHNLKRESVAQQYQSVKQRTSNLQDYGMGSHVMQYGDANITDEKLYLYQGFDPATVNFPPHNRLEAK

MEVVNQRDAELFFMWQMYQRSNQQPEKKTDILKQITETVKHRKHLDGSVELIGVLLYGPGKASSVLQSVR

TPGLPLVDDWTCLKSMVRVFETHCGSLTQYGMKHMRAFANICNSGVSETSMENACVAACGGYQAGQLHPS

NIGYSA

>XP_027940765.1_VuAEP2

RRDLVGDFLRLPSDSGSDDNFQGTRWAILFAGSNGYWNYRHQADICH

AYQILRKGGLKEENIIVFMYDDIAFNWDNPRPGVIINKPDGDDVYEGVPKDYTGEDATAHNFYAALLGDK

SALKGGSGKVVNSGPDDRIFIFYSDHGGPGVLGTPAGPYLYASDLVETLKKKHASGTYKNLVFYLEACEA

GSIFEGLLPEDINIYATTASNAEESSWGTYCPGEYPSPPPEYSTCLGDLYSIAWMEDSDRHNLRTESLHQ

QYKVVKDRTLSGGYYGSHVMQYGDVGLSKDSLFLYLGTDPANDNLTFVDENSLRASSTAVNQRDADLVHF

WHKFRKAPEGSPKKNEARKQVLEVMSHRMHIDDSVKLVGKLLFGIEKAPEVLNAVRPAGSALVDDWECLK

TMVRTFETHCGSLSQYGMKHMRSFANICNVGIKKEQMGEASAQACVSIPDSPWSSLQRGFSA

>KHN04332.1_GsVPE1

AAARANRKEWDSVIKLPAEPVDADSDHEVGTRWAVLVAGSNG

YGNYRHQADVCHAYQLLIKGGLKEENIVVFMYDDIATDELNPRPGVIINHPEGQDVYAGVPKDYTGENVT

AQNLFAVILGDKNKVKGGSGKVINSKPEDRIFIYYSDHGGPGVLGMPNMPYLYAMDFIEVLKKKHASGGY

KKMVIYVEACESGSMFEGIMPKDLQIYVTTASNAQENSWGTYCPGMDPSPPPEYITCLGDLYSVAWMEDS

ETHNLKRESVKQQYKSVKQRTSNFNNYAMGSHVMQYGDTNITAEKLYLYQGFDPAAVNFPPQNGRLETKM

EVVNQRDAELFFMWQMYQRSNHQPEKKTDILKQIAETVKHRKHIDGSVELIGVLLYGPGKGSSVLQSMRA

PGLALVDDWTCLKSMVRVFETHCGTLTQYGMKHMRAFANICNSGVSEASMEEVCVAACEGYDSGLLHPSN

KGYSA

>KHN09785.1_GsVPE2

AAARPNRKEWDSVIKLPTEPVDADSDEVGTRWAVLVAGSNG

YGNYRHQADVCHAYQLLIKGGLKEENIVVFMYDDIATNELNPRHGVIINHPEGEDLYAGVPKDYTGDNVT

TENLFAVILGDKSKLKGGSGKVINSKPEDRIFIYYSDHGGPGILGMPNMPYLYAMDFIDVLKKKHASGSY

KEMVIYVEACESGSVFEGIMPKDLNIYVTTASNAQENSWGTYCPGMDPSPPPEYITCLGDLYSVAWMEDS

EAHNLKRESVKQQYKSVKQRTSNFNNYAMGSHVMQYGDTNITAEKLYLYQGFDPATVNFPPQNGRLETKM

EVVNQRDAELLFMWQMYQRSNHQSENKTDILKQIAETVKHRKHIDGSVELIGVLLYGPGKGSSVLQSVRA

PGSSLVDDWTCLKSMVRVFETHCGTLTQYGMKHMRAFANICNSGVSEASMEEACLAACEGYNAGLFHPSN

RGYSA

>KHN27584.1_GsVPE3

VRPLPNPLIEAHGHVKHATAKRWAVLVAGSKGYDNYRHQADVCH

AYQVLKKGGLKDENIIVFMYDDIANHTLNPRLGTVINKPNGPDVYKGVPKDYTGNATTSENFYAVISGNR

SALSGGSGKVVDSGPNDTIFIYYADHGATGVIGMPVGDFVMANDFVDVLKKKHAAKSYKKMVIYMEACES

GSMFEGILPNNIDVYATTAANTDEDSYGFYCPDLYPTPPPEYTTCLGDEYSISWLEDSDKNDMVNETLQQ

QYETVRRRTLVSHINATSHVMQYGDKELNNDSLAIYIGALAPSLSLNENAHSFEQSTTQTKLISQRDTRL

LHLRLELQKAQDGSEKLKAQKELADEIAHREHVDNVVHLIGDLLFGEENSSAMMFHVRPAGKPLVDDWDC

FKTLVKTYESQCGTLSSYGRKYTRAFANMCNAGIYEEQLKTTTSQACPQKNHAS

>KHN29737.1_GsVPE4

ARHDILRLPSEASTFFKAPGGDQNDEGTRWAVLIAGSNGYWNYRHQSDVCH

AYQLLRKGGLKEENIVVFMYDDIAFNEENPRPGVIINSPHGNDVYKGVPKDYIGEDVTVGNFFAAILGNK

SALTGGSGKVVDSGPNDHIFIYYSDHGGPGVLGMPTNPYMYASDLIEVLKKKHASGSYKSLVFYLEACES

GSIFEGLLPEGLNIYATTASNAEESSWGTYCPGEYPSPPSEYETCLGDLYSVAWMEDSDIHNLQTETLHQ

QYELVKQRTMNGNSIYGSHVMQYGDIGLSENNLVLYLGTNPANDNFTFVHKNSLVPPSKAVNQRDADLIH

FWDKFRKAPVGSSRKAAAEKQILEAMSHRMHIDDSMKRIGKLLFGIEKGPELLSSVRPAGQPLVDDWDCL

KTLVRTFETHCGSLSQYGMKHMRSFANFCNAGIRKEQMAEASAQACVNIPASSWSSMHRGFSA

>KHN32223.1_GsVPE5

ARHDILRLPSEASRFFKAPANADQNDEGTRWAVLVAGSNGYWNYRHQSDVC

HAYQLLRKGGVKEENIVVFMYDDIAFNEENPRPGVIINSPHGNDVYKGVPKDYVGEDVTVDNFFAAILGN

KSALTGGSGKVVDSGPNDHIFIYYSDHGGPGVLGMPTNPYMYASDLIEVLKKKHASGTYKSLVFYLEACE

SGSIFEGLLPEGLNIYATTASNAEESSWGTYCPGEYPSPPPEYETCLGDLYSVAWMEDSDIHNLRTETLH

QQYDLVKERTMNGNSIYGSHVMQYGDIGLSKNNLVLYLGTNPANDNFTFVHKNSLVPPSKAVNQRDADLI

HFWDKFRKAPVGSSRKAAAEKEILEAMSHRMHIDDNMKLIGKLLFGIEKGPELLSSVRPAGQPLVDDWDC

LKTLVRTFETHCGSLSQYGMKHMRSFANFCNAGIRKEQMAEASAQACVSIPASSWSSLHRGFSA

>KHN37015.1_GsVPE6

RPHLASETDNDDNFKGTRWAVLLAGSNGYWNYRHQADVCHAYQILRKGG

LKEENIIVFMYDDIAFNGENPRPGVIINKPDGGDVYEGVPKDYTGEDVTVGNFFAALLGNKSALTGGSGK

VVDSGPDDHIFVYYTDHGGPGVLGMPAGPYLYADDLIEVLKKKHASGTYKNLVFYLEACESGSIFEGLLP

EDINIYATTASNAEESSWGTYCPGEYPSPPPEYSTCLGDLYSVAWMEDSDRHNLRTETLHQQYKLVKERT

ISGDSYYGSHVMQYGDVGLSSDVLFHYLGTDPANDNFTFVDENSLWSPSKPVNQRDADLIHFWDKFRKAP

EGSLRKNAAQKQVLEAMSHRMHVDNSVKLIGKLLFGIEKGPEVLNAVRPAGSALVDDWHCLKTMVRTFET

HCGSLSQYGMKHMRSFANICNVGIKNEQMAEASAQACVSIPSNPWSSLQRGFSA

>RZB58253.1_GsVPE7a

RPHLAGDFLRLPSETDNDDNVQGTRWAVLLAGSNGYWNYRHQADVCHAY

QILRKGGLKEENIIVFMYDDIAFNGENPRPGVIINKPDGGDVYEGVPKDYTGEDVTVGNFFAALLGNKSA

LTGGSGKVVDSGPDDHIFVYYTDHGGPGVLGMPAGPYLYADDLIEVLKKKHASGTYKNLVFYLEACESGS

IFEGLLPEDINIYATTASNAEESSWGTYCPGEYPSPPPEYSTCLGDLYSVAWMEDSDRHNLRTETLHQQY

KLVKERTISGDSYYGSHVMQYGDVRLSSDVLFHYLGTDPANDNFTFVDENSLWSPSKPVNQRDADLIHFW

DKFRKAPEGSLRKNAAQKQVLEAMSHRMHVDNSVKLIGKLLFGIEKGPEVLNAVRPAGSALVDDWHCLKT

MVRTFETHCGSLSQYGMKHMRSFANICNVGIKNEQMAEASAQACVSIPSNPWSSLQRGFSA

>RZB68282.1_GsVPE7a

RRDLV

GDFLRLPSETDNDDNFKGTRWAVLLAGSNGYWNYRHQADVCHAYQILRKGGLKEENIIVFMYDDIAFNGE

NPRPGVIINKPDGGDVYKGVPKDYTGEDVTVDNFFAALLGNKSALTGGSGKVVDSGPDDHIFVYYTDHGG

PGVLGMPAGPYLYADDLIEVLKKKHASGTYKNLVFYLEACESGSIFEGLLPEDINIYATTASNAEESSWG

TYCPGEYPSPPPEYTTCLGDLYSVAWMEDSDRHNLRTETLHQQYKLVKERTISGDSYYGSHVMQYGDVGL

SRDVLFHYLGTDPANDNFTFVDENSLWSPSKPVNQRDADLIHFWDKFRKAPEGSLRKNTAQKQVLEAMSH

RMHVDNSVKLIGKLLFGIEKGPEVLNAVRPAGSALVDDWHCLKTMVSFLF

>RZC15086.1_GsVPE7b

ARHDILRLPSEASTFFKAPGGDQNDEGTRWAVLIAGSNGYWNYRHQSDVCH

AYQLLRKGGLKEENIVVFMYDDIAFNEENPRPGVIINSPHGNDVYKGVPKDYIGEDVTVGNFFAAILGNK

SALTGGSGKVVDSGPNDHIFIYYSDHGGPGVLVLKKKHASGSYKSLVFYLEACESGSIFEGLLPEGLNIY

ATTASNAEESSWGTYCPGEYPSPPSEYETCLGDLYSVAWMEDSDIHNLQTETLHQQYELVKQRTMNGNSI

YGSHVMQYGDIGLSENNLVLYLGTNPANDNFTFVHKNSLVPPSKAVNQRDADLIHFWDKFRKAPVGSSRK

AAAEKQILEAMSHRMHIDDSMKRIGKLLFGIEKGPELLSSVRPAGQPLVDDWDCLKTLVRTFETHCGSLS

QYGMKHMRSFANFCNAGIRKEQMAEASAQACVNIPASSWSSMHRGFSA

>RZC15087.1_GsVPE7c

ARHDILRLPSEASTFFKAPGGDQNDEGTRWAVLIAGSNGYWNYRHQMTDEP

LSFHQSDVCHAYQLLRKGGLKEENIVVFMYDDIAFNEENPRPGVIINSPHGNDVYKGVPKDYIGEDVTVG

NFFAAILGNKSALTGGSGKVVDSGPNDHIFIYYSDHGGPGVLGMPTNPYMYASDLIEVLKKKHASGSYKS

LVFYLEACESGSIFEGLLPEGLNIYATTASNAEESSWGTYCPGEYPSPPSEYETCLGDLYSVAWMEDSDI

HNLQTETLHQQYELVKQRTMNGNSIYGSHVMQYGDIGLSENNLVLYLGTNPANDNFTFVHKNSLVPPSKA

VNQRDADLIHFWDKFRKAPVGSSRKAAAEKQILEAMSHRMHIDDSMKRIGKLLFGIEKGPELLSSVRPAG

QPLVDDWDCLKTLVRTFETHCGSLSQYGMKHMRSFANFCNAGIRKEQMAEASAQACVNIPASSWSSMHRG

FSA

>XP_028199243.1_GsVPE8

RRDLVGDFLRLPSETDNDDNFKGTRWAVLLAGSNGYWNYRHQADVCHA

YQILRKGGLKEENIIVFMYDDIAFNGENPRPGVIINKPDGGDVYKGVPKDYTGEDVTVDNFFAALLGNKS

ALTGGSGKVVDSGPDDHIFVYYTDHGGPGVLGMPAGPYLYADDLIEVLKKKHASGTYKNLVFYLEACESG

SIFEGLLPEDINIYATTASNAEESSWGTYCPGEYPSPPPEYTTCLGDLYSVAWMEDSDRHNLRTETLHQQ

YKLVKERTISGDSYYGSHVMQYGDVGLSRDVLFHYLGTDPANDNFTFVDENSLWSPSKPVNQRDADLIHF

WDKFRKAPEGSLRKNTAQKQVLEAMSHRMHVDNSVKLIGKLLFGIEKGPEVLNAVRPAGSALVDDWHCLK

TMVRTFETHCGSLSQYGMKHMRSFANICNVGIKNEQMAEASAQACVSIPSNPWSSLQRGFSA

>XP_014501414.1_VrVPE1

GRDEILRMPSEASRFFQAPATDENDEGTRWAVLIAGSNGYWNYRHQSDVCH

AYQLLRKGGLKEENIVVFMYDDIAFNEENPRPGVIINSPHGNDVYKGVPKDYVGEDVTVNNFFAAILGNK

SALTGGSGKVVDSGPNDHIFIYYSDHGGPGVLGMPTNPYMYASDLIEVLKKKHASGTYKSLVFYLEACES

GSIFEGLLPEGLNIYATTAANAEESSWGTYCPGEYPSPPAEYETCLGDLYSVAWMEDSDIHNLRTETLHQ

QFELVKQRTINGNSAYGSHVMQYGDIGLSKNNLSLYLGTNPANDNFPFLEKNSLVPPSKAVNQRDADLVH

FWDKFRKAPLGSSRKSVAQKQILEAMSHRMHIDDSVTLIGKLLFGIEEGPELLSSVRPAGQPLVDDWDCL

KTLVRTFETHCGSLSQYGMKHMRSFANLCNAGIRKEQMAEASAQACVSIPASPWSSLSSGFSA

>XP_014510094.1_VrVPE2

AGARHNRRELKSFIKLPTEAVDADSDEVGTRWAVLVAGS

NGYGNYRHQADVCHAYQLLIKGGVKEENIVVFMYDDIANNKQNPRPGVIINHPQGPDVYAGVPKDYTGEN

VTSKNFFAVLLGDKSKVKGGSGKVINSKAEDRIFIYYSDHGGPGVLGMPNLPYLYAMDFIDVLKKKHASG

GYKKMVIYVEACESGSIFEGIMPKDLNIYVTTASNAQENSWGTYCPGFNPSPPPEYSTCLGDLYSVAWME

DSESHNLKKESVAQQYQSVKQRTSNLQDYGMGSHVMEYGDANITAEKLYLYQGFDPATVNFPPLNNARRE

AKMEVVNQRDAELFFMWQMYQRSNQQPEKKTDILKQITETVKHRKHLDGSVELIGVLLYGPGKASSVLQS

VRTPGLPLVDDWTCLKSMVRVFETHCGSLTQYGMKHMRAFANICNSGVSETSMENACVAACGGYHAGQLH

PSNTGYSA

>XP_025606149.1_AhVPE1

VRPIMRNIQQQKGANGNRWGVLVAGSNGYENYRHQADVCHAYQV

LKKGGLKDENIIVFMYDDIANNTQNPKPGTIINKPNGPDVYKGVPKDYSGEHTNAKKFYAVLSGNRSAIT

GGTGKVVDSGPNDTIFIYYADHGAPGFVTMPVGENVFANDFIDVLKKKHAAKGYKKIVIYLEACESGSMF

EGILTNNLNIYATTASNSTDPSFAAYCDNEYDTCLGDVFSVSWLEDSDKTDGRKETLKRQYERVRERTLH

WDDGSSSEVMQYGDKMISNDFLVNYIGANPAINKNSKTNNAYFFDAPTIFVSQRDATILHLRHKLSIAPE

GTSEKSEAEKRLLLEIAEREQVDNNIKRIVNLLFGEKSGSEVITNVRSAGQPLVDDWDCFKNYMKIYETH

CGTLSTYGKKYSRTFANICNAGISEEQMIVASAKAC

>XP_025613441.1_AhVPE2

VRDPIGEVIRLPSEASRFFKAPSDDGDNVEEGTRWAILIAGSNGYWNYRHQ

ADVCHAYQLLRKGGLKEENIIVFMYDDIAFNEENPRPGVIINSPHGDDVYKGVPKDYVGGDVTVNNFFAA

ILGNKSALTGGSGKVVDSGPNDHIFIYYSDHGGPGVLGMPTSPYLYASDLIEVLKKKHASGTYKSMVFYL

EACESGSIFEGLLPEGLNIYATTAANAEESSWGTYCPGENPSPPPEFGTCLGDLYSVAWMEDSAIHNLRT

ETLHQQYQLVKERTSNGNSMYGSHVMQYGDIGISSNNLFLYLGTNPANENFTVMDNNSLKLPSKTAVNQR

DADLVHFWDKYLKAPEGSPRKAAAEKQVMEAMSHRMHIDSSVKLIGKLLFGIEKGQEILSSVRPSGEPLV

DDWDCLKSLVTTFETHCGSLSQYGMKHMRSFANFCNAGIRREQLADASAHACTSVPSNPWSSLHRGFSA

>XP_025658873.1_AhVPE3

VRPMRNIQQQKGANGNRWGVLVAGSNGYENYRHQADVCHAYQVL

KKGGLKDENIIVFMYDDIANNTQNPKPGTIINKPNGPDVYKGVPKDYSGEHTNAKNFYAVLSGNRSAITG

GSGKVVDSGPNDTIFIYYADHGAPGFVTMPVGEDVFANDFIDVLKKKHAAKGYKKIVIYLEACESGSMFE

GILTNNLNIYATTASNSTDPSFAAYCDNEYDTCLGDVYSVSWLEDSDKTDRRKETLKRQYESVRERTLHW

DDGSSSEVMQYGDKMISNDFLDNYIGANPATNKNSKTNNAYFFDAPTIFVSQRDATILHLRHKLSIAPEG

SSEKSEAEKRLLLEIAEREQVDNNIKRIVNILFGEKSGSEEVITNVRSAGQPLVDDWDCFKNYMKIYENH

CGTLSTYGKKYSRTFANICNAGISEEQMIVASAKAC

>KYP55392.1_CcVPE1

RPNRREWDSVIKLPTEPVDADADEVGTRWAVLVAGSNGYG

NYRHQADVCHAYQLLIKGGLKEENIVVFMYDDIATNELNPRPGIIINHPQGQDVYAGVPKDYTGDEVTSQ

NLFAVILGDKSKVKGGSGKVIDSKPEDRIFIYYSDHGGPGVLGMPNMPYLYAMDFIDVLKKKHASGGYKE

MVIYVEACESGSMFEGIMPNDLNIYITTASNAQENSWGTYCPGMEPSPPPEYITCLGDLYSVAWMEDSET

HNLKRESVKQQYYSVKKRTSNFNNYAMGSHVMQYGDTNITGEKLYLYQGFDPDTVNFPPHNGELETKMEV

VNQRDAELIFMWQMYQRSNHHPEKKTDILKQIAETVKHRKHLDGSMDLIGLLLYGPGKGSSILQSVRAPG

LPLVDDWICLKSMVRVFETHCGSLTQYGMKHMRAFANICNSGVSEASMEEASVAACGGYDAGLLHPSNRG

YSA

>KYP58763.1_CcVPE2

GRDNFLQLPSDASTAHENHEGTRWAVLIAGSNGYWNYRHQSDVCHAYQLLR

KGGLKEENIVVFMYDDIAFNEENPRPGVIINSPDGNNVYQGVPKDYVGEDVNVNNFFAAILGNKSALTGG

SGKVVDSGPNDHIFIYYSDHGGPGVLGMPTSPYLYASDLIEVLKKKHASGTYESLVFYLEACESGSIFEG

LLPEGLNIYATTAANAAESSWGTYCPGDYPSPPPEYETCLADLYSVAWMEDSDMHNLRTETLHQQYELVK

QRTINGNAMYGSHVMQFGDIELSKNNLFLYLGTNPANDNFTFVDKNSLVPPSKAVNQRDADLVHFWDKFR

KAPVGSSRKTAAEKQLLEAMSHRMHIDDNMKLIGKLLFGIEKGPELLTSVRPAGQPLVDDWDCLKTLVRT

FETHCGSLSQYGMKHMRSFANFCNAGIQKEQMSEASAQACISIPATPWSSLHKGFSA

>KYP60634.1_CcVPE3

EDFLRLPSQSDNDDNDQGTRWAILLAGSNGYWNYRHQADVCHAYQI

LRKGGLKEENIIVFMYDDIAYNEENPRQGVIINKPDGDDVYEGVPKDYTGEDVTANNFYAALLGNKSALT

GGSGKVVDSGPDDHIFVYYTDHGGPGVLGMPAGPYIYASDLIEVLKKKFDAGTYKNLVFYLEACESGSIF

EGLLPEDINIYATTASNAEESSWGTYCPGEYPSPPPEYTTCLGDLYSVAWMEDSDRHNLRTETLHQQYKL

VKDRTLYGDAYYGSHVMQYGDVGFSNDILFLYMGTNPANDNLTFVDENTLRSRSPPSKAVNQRDADLVHF

WEKFRKSPEGSPRKNAAQKQVLEAMSHRMHVDKSVKLIGKLLFGIEKGPELLNAVRPAGLALVDDWDCLK

TMVRTFETHCGSLSQYGMKHMRSFANICNAGVKNEQMAEASAQACDRFPANPWSSLQRGFSA

>XP_020232504.1_CcVPE4

VPVLHDHGENATEKKWALLVAGSYGYQNYRHQADVCHAYQLLKN

GGLKDENIIVFMYDDIAFNPQNPRPGTIINKPNGPNVYKGIPKDYTGNDTTTENFYAVISGNKSAISGGS

GKVVNSGPNDTIFIYFVDHGSNGILSMPVGEYITANDFVNVLKKKHDAKSYKKMVIYLEACESGSMFDGI

LPNDINIYATTASNASEDSYAYYCPHHYPFPPPEYTTCLGDVYSNAWLEDSDDNDMTKETLQQQYETVRR

RTLVGNINASSHVTQYGDTKFSNDFLATFIGAHPASPNGNSTSSGNASSFEPSTTQTKLVKQRDAPLLHL

WLELQKAPNGSKEKLEAQKELDDEIAHRKHVDSVFHLIGDLLFGEENNSSTMLLHVRPPGQPLVDDWDCF

KTLIKTYESHCGKLSIYGRKYTRAFANMCNAGISEEQMVVASSQACPKENHAS

>XP_020203756.1_CcVPE_delta

ALLASLWMSFSVSVLEGVGPMADPLKLHRHGKN

IGGKKWAILVAGSNGYGNYRHQADICHAYQILKKGGLKDENIVVFMYDDIAFDPQNPRAGVIINKPNGPN

VYEGVPKDYVGDAANAKNFYAVLSGNRSALSGGSGKVVDSGPNDTIFIYYSDHGAPGLVTMPVGEYVMAN

DFVNVLKKKHDAKSYKKMVIYLEACESGSMFEGILPNNISIYATTASNADEDSFAYYCPHSYPSPPTEYT

TCLGDVYSISWLEDSDKNDMTIETLQQQYETVRRRTLIGNVDTSSHVKQYGDRKFENDTLATYIGAPVKT

NPTNSANAYSFEPYSPQTRHVSQRDAHLLYLKLELQKAPDGSMEKLKAQIELDDEIAHRKHLDSVFHLIG

DLLFGEENNISTMLLHVRPPGQPLVDDWDCFKTLIKTYESNCGKLSIYGRKYTRAFANMCNAGISEEQMV

VASSQACPKENPS

>XP_006578073.1_GmVPE3b

ARHDILRLPSEASTFFKAPGGDQNDEGTRWAVLIAGSNGYWNYRHQSDVCH

AYQLLRKGGLKEENIVVFMYDDIAFNEENPRPGVIINSPHGNDVYKGVPKDYIGEDVTVGNFFAAILGNK

SALTGGSGKVVDSGPNDHIFIYYSDHGGPGVLGMPTNPYMYASDLIEVLKKKHASGSYKSLVFYLEACES

GSIFEGLLPEGLNIYATTASNAEESSWGTYCPGEYPSPPSEYETCLGDLYSVAWMEDSDIHNLQTETLHQ

QYELVKQRTMNGNSIYGSHVMQYGDIGLSENNLVLYLGTNPANDNFTFVLKNSLVPPSKAVNQRDADLIH

FWDKFRKAPVGSSRKAAAEKQILEAMSHRMHIDDSMKRIGKLFFGIEKGPELLSSVRPAGQPLVDDWDCL

KTLVRTFETHCGSLSQYGMKHMRSFANFCNAGIRKEQMAEASAQACVNIPASSWSSMHRGFSA

>XP_006599066.1_GmVPE1

VRPLPNPLIEAHGHVKHATAKRWAVLVAGSKGYDNYRHQADVCH

AYQVLKKGGLKDENIIVFMYDDIANHTLNPRLGTVINKPNGPDVYKGVPKDYTGNATTSENFYAVISGNR

SALSGGSGKVVDSGPNDTIFIYYADHGATGVIGMPVGDFVMANDFVDVLKKKHAAKSYKKMVIYMEACES

GSMFEGILPNNIDVYATTAANTDEDSYGFYCPDLYPTPPPEYTTCLGDEYSISWLEDSDKNDMVNETLQQ

QYETVRRRTLVSHINATSHVMQYGDKELNNDSLAIYIGALAPSLSLNENAHSFEQSTTQTKLISQRDTRL

LHLRLELQKAQDGSEKLKAQKELADEIAHRKHVDNVVHLIGDLLFGEENSSAMMFHVRPAGKPLVDDWDC

FKTLVKTYESQCGTLSSYGRKYTRAFANMCNAGIYEEQLKTTTSQACPQKNHAS

>XP_014629960.1_GmVPE3a

ARHDILRLPSEASTFFKAPGGDQNDEGTRWAVLIAGSNGYWNYRHQMTDEP

LSFHQSDVCHAYQLLRKGGLKEENIVVFMYDDIAFNEENPRPGVIINSPHGNDVYKGVPKDYIGEDVTVG

NFFAAILGNKSALTGGSGKVVDSGPNDHIFIYYSDHGGPGVLGMPTNPYMYASDLIEVLKKKHASGSYKS

LVFYLEACESGSIFEGLLPEGLNIYATTASNAEESSWGTYCPGEYPSPPSEYETCLGDLYSVAWMEDSDI

HNLQTETLHQQYELVKQRTMNGNSIYGSHVMQYGDIGLSENNLVLYLGTNPANDNFTFVLKNSLVPPSKA

VNQRDADLIHFWDKFRKAPVGSSRKAAAEKQILEAMSHRMHIDDSMKRIGKLFFGIEKGPELLSSVRPAG

QPLVDDWDCLKTLVRTFETHCGSLSQYGMKHMRSFANFCNAGIRKEQMAEASAQACVNIPASSWSSMHRG

FSA

>NP_001236678.2_GmVPE2

AAARPNRKEWDSVIKLPTEPVDADSDEVGTRWAVLVAGSNG

YGNYRHQADVCHAYQLLIKGGLKEENIVVFMYDDIATNELNPRHGVIINHPEGEDLYAGVPKDYTGDNVT

TENLFAVILGDKSKLKGGSGKVINSKPEDRIFIYYSDHGGPGILGMPNMPYLYAMDFIDVLKKKHASGSY

KEMVIYVEACESGSVFEGIMPKDLNIYVTTASNAQENSWGTYCPGMDPSPPPEYITCLGDLYSVAWMEDS

EAHNLKRESVKQQYKSVKQRTSNFNNYAMGSHVMQYGDTNITAEKLYLYQGFDPATVNFPPQNGRLETKM

EVVNQRDAELLFMWQMYQRSNHQSENKTDILKQIAETVKHRKHIDGSVELIGVLLYGPGKGSSVLQSVRA

PGSSLVDDWTCLKSMVRVFETHCGTLTQYGMKHMRAFANICNSGVSEASMEEACLAACEGYNAGLLHPSN

RGYSA

>TKY55414.1_SsVPE1

SAAARPNRKEWDSVIKLPTEPVDADSDEVGTRWAVLVAGS

SGYGNYRHQADVCHAYQLLIKGGLKEENIVVFMYDDIATNDLNPRPGVIINHPQGQDVYAGVPKDYTGES

VTSENLFAVILGDKSKVKGGSGKVINSKPEDRIFMYYSDHGGPGVLGMPNMPYLYAMDFIDVLKKKHASG

GYKEMVIYVEACESGSMFEGIMPKDLNIYVTTASNAQENSWGTYCPGMNPSPPPEYITCLGDLYSVAWME

DSETHNLKRESVKQQYQSVKKRTSNFNNYAMGSHVMQYGDTNITTEKLYLYQGFDPATVNFPPHNGRLRA

KMEVVNQRDAELFFMWQMYQRSNHHPEKKTDILKQIGETVKHRKHLDGSVELLGVLLYGPGKGSSVLQSV

RAPGLPLVDDWACLKSMVRVFETHCGSLTQYGMKHMRAFANICNSGISEASMEEACVATCGGYDAGQLYP

SNRGYSA

>TKY57951.1_SsVPE2

RRDLAGDFLRLPSQPDNDDNVQGTRWAILLAGSNGYWNYRHQADICHAYQI

LRKGGLKEENIIVFMYDDIAFNEENPRPGVIINKPDGDDVYEGVPKDYTGEDVTANNFFAALLGNKSALT

GGSGKVVDSGPDDRIFIYYSDHGGPGVLGMPAGPYLYASDLIEVLKKKHASGTYKSLVFYLEACESGSIF

EGLLPEDINIYATTASNAEESSWGTYCPGEYPSPPPEYTTCLGDLYSVAWMEDSDRHNLRTETLHQQYKL

VKERTIYGGMYYGSHVMQYGDIERSSDVLFLYLGTNPANDNFTFADENSLGSPSKAVNQRDADLIHFWDK

FRKAPEGSPRKNAAQKQVLEAMSHRMHIDNSVKLVGKLLFGIEKGPEVLNAVRPSGLALVDDWDCLKTMV

RAFETHCGSLSQYGMKHMRSFANICNAGIKNEQMAEASAQACVSIPANTWSSLQRGFSA

>TKY74927.1_SsVPE3

GRDDILRLPSEASRFFQAPAADENDEGTRWAVLIAGSNGYWNYRHQSDVCH

AYQLLRKGGLKEENIVVFMYDDIAFNEENPRPGVIINSPHGNDVYKGVPKDYVGEEVTVNNFFASILGNK

SALTGGSGKVVDSGPNDHIFIYYSDHGGPGVLGMPTSPYMYASDLIEVLKKKHASGTYKSLVFYLEACES

GSIFEGLLPEGLNIYATTAANAEESSWGTYCPGEYPSPPPEYETCLGDLYSVAWMEDSDTHNLRTETLHQ

QYELVKQRTMNGNSIYGSHVMQYGDIRLSKNNLFLYLGTNPANDNFTFVDENSLVPPSKAVNQRDADLIH

FWDKFRKAPVGSSRKAAAEKQILEAMSHRMHIDDSIKLIGKLLFGIEKGPEVLSSVRPAGQPLVDDWDCL

KTLVRTFETHCGSLSQYGMKHMRSFANFCNAGIQKEQMAEASAQACDSIPATPWSTLHRVSVHNS

**C. ER oxidoreductin-1 protein sequences**

>CtERO1 (ctr203_c0_g1_i1)

MSSRSANCLFMLVVNVCLYMSVVCFFSSSSTIVNSRIDNSWLNELKGNIDETNSTVEIVD

KFNNYQIYPRIVSIVSKDYFKFFKVNLRRTCPFWSDDSKCAMRYCQVESCQLDDLPIGLK

GSHHSLVDEDMSIKYMEGPQQQECEEAMHDELGYINTTISAAAMEDFALWQAHDDIQDMY

CTLPDDDGEAEYVDLSLNPERYTGYKGPSANRVWNTIYMENCFRPKNLFGVYMISSKLTG

MCLEKRAFYRAISGLHTSINIHLSAQYLLSDKGSTFLNPCGTGFWGPNAEEFERKFGPKY

TKGEGTQWLRNLYFLYLVELRALQKAAPLLKQVEYYTGNDKEDEATRLAVHNFLGIIKKF

PQQFNEHTMFLGGQQAQKLKEEFRLHFRNVSTIMDCVGCDKCRLWGKLQVQGLGTALKIL

FSNKARTNGYNELSTTVNKHTLQLERSEIVALFNAFGRLSTSIYEIEKFRKLLR

>OsERO1

MPPQEPAPAANGAAPAPAPAAAAAAAGGKRRKRGRWAAAAGVGALLVALLAVAVSSRSFP

AASSSSRGGDCGCPGTRKYTGMVEDCCCDYETVDAINEEVLHPILQELVTLPFFRYFKVK

LWCDCPFWPDDGMCRLRDCSVCECPENEFPEPFKKPYSGLSPDSMICQEGKPQATVDRTL

DAKVFKGWIETDNPWTYDDETDNAEMTYVNLQLNPERYTGYTGDSARRIWDSVYKENCPK

YPSEEMCQEKKALYKLISGLHSSISVHIAYDYLLDESANLWGHNLPLLYDRVLKYPERVQ

NLYFTYLFVLRAVTKAADYLEQAEYNTGNPEEDLKTQSLVKQLLYNPKLRSACPLPFDEA

KLWQGENGPELKQEIQKQFRNISAIMDCVGCEKCRLWGKLQVLGLGTALKILFSVDGENH

LNQSLQLQRNEVIALVNLLNRLSESVNFVHEKGPSIEDVIKQQSSSTVKPVFPI*

>GmERO1a

MVKSEIEKKGCSTRQWLWLVMALVAVFVAMAMSSKTSPKALFGAIDRACPCARGTPKYSG

MVEDCCCDYETVDRLNEEVLHPSLQELVKTPFFRYFKVKLWCDCPFWPDDGMCRLRDCSV

CECPENEFPESFKKPDRRLSMTDLVCQEGKPQAAVDRTLDSKAFRGWTEIDNPWTNDDET

DNDEMTYVNLQLNPERYTGYTGPSARRIWDAVYSENCPKYPSQELCQEEKILYKLISGLH

SSISIHIASDYLLEEATNLWGQNLTLMYDRVLRYPDRVRNLYFTFLFVLRAVTKASDYLE

QAEYDTGNPNEDLTTQSLIKQLLYNPKLQAACPIPFDEANLWKGQSGPELKQKIQQQFRN

ISALMDCVGCEKCRLWGKLQVLGLGTALKILFSVDGQENSSHTLQLQRNEVIALTNLLNR

LSESVKFVHEVGPTAERIMEGGHFSAHTRTLISSWKKIWSYVSKT

>GmERO1b

MVKAEIEKKGCSTRRWLWLVMALVAVFVAMVMSSRTSPKALFGAIDRACPCARGTPKYSG

MVEDCCCDYETVDRLNEEVLHPSLQELVKTPFFRYFKVKLWCDCPFWPDDGMCRLRDCSV

CECPESEFPESFKKPDHRLSMTDLVCQEGKPQAAVDRTLDSKAFRGWTEIDNPWTNDDET

DNDEMTYVNLQLNPERYTGYTGPSARRIWDAVYSENCPKYPSQELCHEEKILYKLISGLH

SSISIHIASDYLLDEATNLWGQNLTLMYDRVLRYPDRVRNLYFTFLFVLRAVTKASDYLE

QAEYDTGNPNEDLTTQSLIKQLLYNPKLQAACPIPFDEANLWKGQSGPELKQKIQQQFRN

ISAMMDCVGCEKCRLWGKLQVLGLGTALKILFSVDGQENSSHTLQLQRNEVIALTNLLNR

LSESVKFIQEMGPTAERIMEGGHFSAHTRTLISSWKKIWSYVSKT

>AetERO1

MSEGPPPEADGAVRRRRRRWAAAAGALLVALLAVALSSRGFPSISSLSRGGGCGCPGARKYTGMVEDCCC

DYETVDAINEEVLNPILQDLVALPFFRYFKVKLWCDCPFWPDDGMCRLRDCSVCECPDNEFPEPFKKPYS

GLSPENMICQEGKPEATVDRTLDTKVFKGWVETDNPWTSDDETDNAEMTYVNLQLNPERYTGYTGDSARR

IWDAIYKENCPKYPSEDMCQEKKALYKLISGLHSSISVHIAYDYLLDESANLWGHNLSLLHDRVLKYPER

VENLYFTYLFVLRAVTKAADYLEQAEYNTGNPEEDLKTQSLVRQLLYNHKLRSACPLPFDEAKLWQGENG

PELKQEIQKQFRNISAIMDCVGCEKCRLWGKLQVLGLGTALKILFSVDGENNLNQQFQLQRNEVIALVNL

LNRLSESVKFVHETGSSSQEIIKQQSFSTLQKGAS

>AtERO1

MGKGAIKEEESEKKRKTWRWPLATLVVVFLAVAVSSRTNSNVGFFFSDRNSCSCSLQKTG

KYKGMIEDCCCDYETVDNLNTEVLNPLLQDLVTTPFFRYYKVKLWCDCPFWPDDGMCRLR

DCSVCECPENEFPEPFKKPFVPGLPSDDLKCQEGKPQGAVDRTIDNRAFRGWVETKNPWT

HDDDTDSGEMSYVNLQLNPERYTGYTGPSARRIWDSIYSENCPKYSSGETCPEKKVLYKL

ISGLHSSISMHIAADYLLDESRNQWGQNIELMYDRILRHPDRVRNMYFTYLFVLRAVTKA

TAYLEQAEYDTGNHAEDLKTQSLIKQLLYSPKLQTACPVPFDEAKLWQGQSGPELKQQIQ

KQFRNISALMDCVGCEKCRLWGKLQVQGLGTALKILFSVGNQDIGDQTLQLQRNEVIALV

NLLNRLSESVKMVHDMSPDVERLMEDQIAKVSAKPARLRRIWDLAVSFW

>AtERO2

MAETDVGSVKGKEKGSGKRWILLIGAIAAVLLAVVVAVFLNTQNSSISEFTGKICNCRQA

EQQKYIGIVEDCCCDYETVNRLNTEVLNPLLQDLVKTPFYRYFKVKLWCDCPFWPDDGMC

RLRDCSVCECPESEFPEVFKKPLSQYNPVCQEGKPQATVDRTLDTRAFRGWTVTDNPWTS

DDETDNDEMTYVNLRLNPERYTGYIGPSARRIWEAIYSENCPKHTSEGSCQEEKILYKLV

SGLHSSISVHIASDYLLDEATNLWGQNLTLLYDRVLRYPDRVQNLYFTFLFVLRAVTKAE

DYLGEAEYETGNVIEDLKTKSLVKQVVSDPKTKAACPVPFDEAKLWKGQRGPELKQQLEK

QFRNISAIMDCVGCEKCRLWGKLQILGLGTALKILFTVNGEDNLRHNLELQRNEVIALMN

LLHRLSESVKYVHDMSPAAERIAGGHASSGNSFWQRIVTSIAQSKVPSEVNIEWVEKRE

>BdERO1

MTSKPAPTANGAGPDAGAGGGVRRRRRFWVAAAVVLLLALLAAAVSSRSFPAISSYSRSGGCGCPGARKY

TGMVEDCCCDYETVDAINEEVLYPILQELVSLPFFRYFKVKLWCDCPFWPDDGMCRLRDCSVCECPDNEF

PEPFKKPYSGLSPENMICQEGKPEATVDRTLDTKVFKGWVETDNPWTSDDETDNAEMTYVNLQLNPERYT

GYTGDSARRIWDSIYKENCPKYPSEDMCQEKKALYKLISGLHSSISVHIAYGYLLDESANLWGHNLPLLY

DRVLKYPERVQNLYFTYLFVLRAVTKAADYLEQAEYNTGNPEEDLKTQSLVKQLLYNHKLRSACPLPFDE

AKLWQGENGPELKQEIQKQFRNISAIMDCVGCEKCRLWGKLQVLGLGTALKILFSVDGENNLNQQFHLQR

NEVIALVNLLNRLSESVKFVHEKGQSTEEGIKQLRSSTVQKGAS

>BdERO2

MTSKPSPTPNGAGAGAGGGVRRWKRLWVATAAGLLLVLLAAAVSSRSLPAIPSYSRSGGCGCPGARKYTG

LVEDCCCDYETVDAINEDVLYPILQQLVSLPFFRYFKVKLWCDCLFWPDDGVCALKDCSVCECPDNEFPE

PFKKPYSGLSPQNMICQGGKPEATVDRTLDSKVFKGWVETDSPWTSDDETDNAKMNYVNLRLNPERYTGY

TGDPARRIWDSIYKEKCIKYPSEDMCQEKKALYKLISGLHSSISVHIAYDYLLDKSTDLWGHNLPLLYDR

VLKYPERVQNLYFTYLFVLRAVTKATDYLEQAEYNTGNPEEDLKTQSLVKQLLYNHKLRSTCPLPFDEAK

LWQGENGPELKQEIQKQFRNISAIMNCVGCEKCLLWGKLQVLGLGTALKILFSVDRENNLNQQFHLQRND

AIALVNLLNKLSESVKFVHEKGPSAEEGIKQLRSSTVQKGAS

>HsERO1_alpha

MGRGWGFLFGLLGAVWLLSSGHGEEQPPETAAQRCFCQVSGYLDDCTCDVETIDRFNNYRLFPRLQKLLE

SDYFRYYKVNLKRPCPFWNDISQCGRRDCAVKPCQSDEVPDGIKSASYKYSEEANNLIEECEQAERLGAV

DESLSEETQKAVLQWTKHDDSSDNFCEADDIQSPEAEYVDLLLNPERYTGYKGPDAWKIWNVIYEENCFK

PQTIKRPLNPLASGQGTSEENTFYSWLEGLCVEKRAFYRLISGLHASINVHLSARYLLQETWLEKKWGHN

ITEFQQRFDGILTEGEGPRRLKNLYFLYLIELRALSKVLPFFERPDFQLFTGNKIQDEENKMLLLEILHE

IKSFPLHFDENSFFAGDKKEAHKLKEDFRLHFRNISRIMDCVGCFKCRLWGKLQTQGLGTALKILFSEKL

IANMPESGPSYEFHLTRQEIVSLFNAFGRISTSVKELENFRNLLQNIH

>HsERO1_beta

MSQGVRRAGAGQGVAAAVQLLVTLSFLRSVVEAQVTGVLDDCLCDIDSIDNFNTYKIFPKIKKLQERDYF

RYYKVNLKRPCPFWAEDGHCSIKDCHVEPCPESKIPVGIKAGHSNKYLKMANNTKELEDCEQANKLGAIN

STLSNQSKEAFIDWARYDDSRDHFCELDDERSPAAQYVDLLLNPERYTGYKGTSAWRVWNSIYEENCFKP

RSVYRPLNPLAPSRGEDDGESFYTWLEGLCLEKRVFYKLISGLHASINLHLCANYLLEETWGKPSWGPNI

KEFKHRFDPVETKGEGPRRLKNLYFLYLIELRALSKVAPYFERSIVDLYTGNAEEDADTKTLLLNIFQDT

KSFPMHFDEKSMFAGDKKGAKSLKEEFRLHFKNISRIMDCVGCDKCRLWGKLQTQGLGTALKILFSEKEI

QKLPENSPSKGFQLTRQEIVALLNAFGRLSTSIRDLQNFKVLLQHSR

>RcERO1

MVGSEPEKKKRNSEGKQWRWVVIGAIIALIFAFTTASITTPNINLFGQPNESCSCPQKYS

GMVEDCCCDYETVDRLNEEVLHPSLQDLVKTPFFRYFKVKLWCDCPFWPDDGMCRLRDCS

VCECPEGEFPESFRTPFRGGLPSDNLLCQEGKPQAAVDRTLDSKAFRGWTEIDNPWTNDD

ETDNAEMTYVNLQLNPERYTGYTGPSARRIWDAVYSENCPRYPSEELCQEERILYKLISG

LHSSISIHIAADYLLDESKNLWGQNLTLMYDRVLRYPDRVRNLYFSFLFVLRAVTKAAEY

LEQAEYDTGNPTEDLRTHSLMRQLLYNSKLQAACPLPFDEAKLWKGQRGPELKQKIQERF

RNISALMDCVGCEKCRLWGKLQVLGLGTALKILFSDNGREHLGQTLSSQWLTHEPFQLLV

IAQLVTYFYFGTETNCLGPQLQLQRNEVIALMNLLNRLSESVKLVPEMGPAVELSIGQIS

SPFSPSNLWQRVWGFKHELADGNTTSEQNTLER

>SbERO1

MTMDAAPVANGAAAGAGGGANRRGGRLWYAAAGALLVALLAVAVSYRSFPDIPSSSPGSCGCPAARKYTG

MVEDCCCDYETVDAINEEVLHPILQELVKLPFFRYFKVKLWCDCPFWPDDGMCKLRDCSVCECPENEFPE

PFRKPYSGLSPDSMMCQEGKPQAAVDKTLDSKVFKGWVETDNPWTSDDETDNNEMTYVNLQLNPERYTGY

TGDSARRIWDAIYKENCPKYPSEELCHEKKALYKLISGLHSSISVHIAYDYLLDESTNSWGQNLPLLYDR

VLKYPERVQNLYFTYLFVLRAVTKAADYLEQAEYNTDNPEDDLKTESLVKQLLYNSKLRSACPLPFDEAK

LWQGENGPELKQEIQKQFRNISAIMDCVGCEKCRLWGKLQVLGLGTALKILFSVDGDSHLNQPLQLQRNE

VIALFNLLNRLSESVKFVHEKGSSIEEVIKEQIPSTFQKGASKPNLKLDFL

>SbERO2

MTMDPAPAANGAAAVAGGGMKRQGGRWWYAAAGALIVALLAVAVSFYSFRGIPSSPPGGCGCPVRISPLA

ARKYTGMVEDCCCDYETVDAINEEVLHPILQELVKLPFFRYFKVKLWCDCPFWPDDGMCKLRDCSVCECP

ENEIPEPFKKPYSGLSPDSMICQEGKPQATIDKTLDSKVFKIWVETDNPWTSDDEADSKEMAYVNLQLNP

ERYTGYTGDSARRIWDAIYKENCPKYPSEELCHEKKALYKLISGLHSSISVHIAYDYLLDESTNSWGQNL

PLLYDRVLKYPERVQNLYFTYLFVLRAVTKAADYLEQADYNTGNPEDDWKTRSLVKQLLYNSKLRSACPL

PFDEAKLWQGENGPELKQEIQKQFRNISAIMDCIGCEKCRLWGKLQVLGLGTALKILFSVNGDSHLNQPL

QLQRNEVIALFNLLNRLSESVKFVHEKGSSIEEVIKEQSPSTLQKGGSKPNVKLDFL

>PtERO1

MVKKNESFKPKQESKKERKRWSSWGLIGALVAVALAVVVAVTVSLMTASKIGSLINSNNKSCQCPSSQDS

GKYKGVIEDCCCDYESVDSVNGEVLHPLLQELVTTPFFRYFKVKLWCDCPFWPDDGMCRLRDCSVCECPE

NEFPEPLKKPFLYGLPADDVTCQEGNPQAAVDRTLDSRAFKGWIETDNPWTNDDETDNDEMTYVNLLLNP

ERYTGYVGPSARRIWDAVYSENCPKYPSGEMCQEKKVLYKLISGLHSSISIHIAVDYLLDESTNKWGQNP

ELMFDRVLRYPDRVRNLYFTFLFVLRAVAKAADYLEQAEYDTGNHTEDLETQSLVRQLLHNPKLQAACPL

PFDEAKLWQGQSGPELKQQIQKQFRNISALMDCVGCEKCRLWGKLQVLGLGTALKILFSVDGQNQPSESL

QLQRNEVIALVNLLNRLSESIKYVCEQGPSIEKTMERQISDPSETKYAST

>PtERO2

MVKFTKSFKPKQENKKERRKWCSWWLIGASLAVVLAVIAAGTVSPMNASKIGSLISSNYKSCQCSSAQDS

GKYKGMIEDCCCDYESVDSVNGEVLHPLLQELVTTPFFRYFKVKLWCDCPFWPDDGMCRLRDCSVCECPE

NEFPEPFKKPFRRGLSADDLMCQEGKPQAAVDRTLDSRAFRGWIVTDNPWTNDDETDNGELTYVNLLLNP

ERYTGYAGSSARRIWDAVYSENCPKYASGEICQEKKVLYKLISGLHSSISIHIAADYLLDESTNKWGQNL

ELMYDRVLRYPDRVRNLYFTFLFVLRAMTKAADYLEQAEYDTGNNTEDLKTQSLVRQLLYNPKLQAACPL

PFDEAKLWQGQSGPELKQQIQKQFRNISALMDCVGCEKCRLWGKLQVLGLGTALKILFSVDGQNQPSESP

QLQRNEVIALVNLLNRLSESVKFVREQGPSIEKIMERQISDSSEPKHGSKWQRAGESLFQLW

>ScERO1

MRLRTAIATLCLTAFTSATSNNSYIATDQTQNAFNDTHFCKVDRNDHVSPSCNVTFNELNAINENIRDDL

SALLKSDFFKYFRLDLYKQCSFWDANDGLCLNRACSVDVVEDWDTLPEYWQPEILGTFNNDTMKEADDSD

DECKFLDQLCQTSKKPVDIEDTINYCDVNDFNGKNAVLIDLTANPERFTGYGGKQAGQIWSTIYQDNCFT

IGETGESLAKDAFYRLVSGFHASIGTHLSKEYLNTKTGKWEPNLDLFMARIGNFPDRVTNMYFNYAVVAK

ALWKIQPYLPEFSFCDLVNKEIKNKMDNVISQLDTKIFNEDLVFANDLSLTLKDEFRSRFKNVTKIMDCV

QCDRCRLWGKIQTTGYATALKILFEINDADEFTKQHIVGKLTKYELIALLQTFGRLSESIESVNMFEKMY

GKRLNGSENRLSSFFQNNFFNILKEAGKSIRYTIENINSTKEGKKKTNNSQSHVFDDLKMPKAEIVPRPS

NGTVNKWKKAWNTEVNNVLEAFRFIYRSYLDLPRNIWELSLMKVYKFWNKFIGVADYVSEETREPISYKL

DIQ

>ZmERO1

MTMTPAPVANGAAAGAGGGMKRRGGRLWYAAAGALLVALLAVAVSYRSFPGVPSSPSSPGSCGCPAARKY

TGMVEDCCCDYETVDAINEEVLHPILQELVKLPFFRYFKVKLWCDCPFWPDDGMCKLRDCSVCECPENEF

PEPFRKPYNGLSPDSMMCQEGKPQAAVDKTLDSKVFKGWVETDNPWTSDDETDNNEMTYVNLQLNPERYT

GYTGDSARRIWDAIYKENCPKYPSEELCHEKKALYKLISGLHSSISVHIAYDYLLDESTNSWGQNLPLLY

DRVLKYPERVQNLYFTYLFVLRAVTKAANYLEQAEYNTGNPEDDLKTESLVKQLLYNSKLRSACPLPFDE

AKLWQGENGPELKQEIQKQFRNISAIMDCVGCEKCRLWGKLQVHGLGTALKILFSVDGDSHMNQPLQLQR

NEVIALFNLLNRLSESVKFVHEKGSSIEEVIEEQIPSTVQKSVSMPNLKLDFL

>ZmERO2

MNPAPVANGAAAGAGVGMNRLGGRLWYAAAGALIVALLAVAVSYRSFPDIPSSSPGSCGCPAARKYTGMV

EDCCCDYETVDAINEEVLHPILQELVKLPFFRYFKVKLWCDCPFWPDDGMCKLRDCSVCECSENEFPEPF

RKPYSGLSPDSMMCQEGKPQAAVDKTLDSKVFKRWVETDNPWTSDDETDNNEMTYVNLQLNPERYTGYTG

DSARRIWDAIYKENCPKYPSEELCHEKKVLYKLISGLHSSISVHIAYDYLLDESTNSWGQNLSLLYDRVL

KYPERVQNLYFTYLFVLRAVTKAADYLEQAEYNTGNPEDDLKTESLVKQLLYNSQLRSACPLPFDEAKLW

QGENGPELKQEIQKQFRNISAIMDCVGCEKCRLWGKLQVLGLGTALKILFSVDGDSHLNQPLQLQRNEVI

ALFNLLNRLSESVKFVHEKGSSVEEVINEQSPSTVQKGASKTNLKGLFGYSQYTWIGWD

>A0A2G9HAS9_HiERO

MVEAKAFADEKKIRKKLKEGKRGKMKWAAIWAVVVVAIAITSKLAQNHKSCLCSKDSQKY

TGIVEDCCCDYETVDSLNGAVLHPLLQELVTTPFFRYFKVKLWCDCPFWPDDGMCKLRDC

SVCECPENEFPETFKKPMQRILSSDDLKCQEGKPKAAVDRTLDSKAFRGWIEVDNPWTND

DETDNSEMTYVNLQLNPERYTGYTGPSARRIWDAVYSENCPKYTSGEICQEKRVLYKLIS

GLHSSISIHIASEYLLDEAKNLWGRNLELMHDRVLRYPDRVQNLYFTFMFVLRAVTKAAD

YLEQAEYDTGNVEEDLKAQSLMRQLLYNPKLRAACPLPFDEAKLWQGQSGPELKQEIQKN

FRNISALMDCVGCEKCRLWGKLQVLGLGTALKILFSVDSKNHPYQPLQLQRNEVIALVNL

LNRLSESVKFVHEIASVEKTMERFTSEHPMQEIGLWQRENISALMDCVGCEKCRLWGKLQ

VLGLGTALKILFSVDSKNHPYQPLQLQRNEVIALVNLLNRLSESVKFVHEIASVEKTMER

FTSEHPMQEIGLWQRVWEARKWLRSIIPL

>A0A124SHU0_CcERO1

MVEMDGKENAGGNGGYKKRNNNNRWRRWGVVGAVIAVLVAVYLNPLWHSHTISFLHKPYL

CSETSLIGLFYFSFVEQDTCKYTGIVEDCCCDYESIDDVNGAVLHILLQELVATPFFRYF

KVKLWCDCPFWPDDGMCRLRDCSVCECPDHEFPESFKKPSLHILPKDDPVCQEGKPEATV

DRTLDAKSFRGWVEIDNPWTHDDETDNGEMTYVNLQLNPERHTGYTGPSARRIWDAIYSE

NCPRYAFGEVCPEKKVLYKLISGMHSSISVHIAADYLLDETANQWGPNLELMHDRVVKHP

DRVQNLYFTFLFILRAVTKAATYLDQAEYDSGNHAEDLKAQSLIRKLVHNPKLQAACALP

FDEAEIALMDCVGCEKCRLWGKLQVLGLGTALKILFSVNDRGNPDPHLQLQRNEVIALIN

LLNRLSESLIYVNKMGSSVPSAKEMSSIHRQWGSLIAHWYTLSCSNHATLDLWLKTKSCE

LLFSVGDGEGDGERRPPTEAATETKGDGDEGPTATTTGEGEGCKLWEKMERCLALDMDME

MEMETM

>A0A200QG63_McERO1

MVKAEIGEKSEKKRWKWGWVVGAILVVLLASAVTSRSPSKISLLPHKIKEACKCAESRQY

TGIVEDCCCDYETVDSLNGEVLNPLLQELVTTPFFRYFKVKLWCDCPFWPDDGMCRLRDC

SVCECPENEFPETFKKSIHHGLSSQDLVCQEEKPQATVDRTLDSKAFRGWVEIDNPWTND

DETDNAKMTYVNLQLNPERYTGYTGPSARRIWDAIYTENCPKYPFGEFCQEKKVLYKLIS

GLHSSISIHIAADYLLDEAVNLWGQNLELMYDRVLQYPDRVRNLYFTFLFVLRAVTKAAD

YFEQAEYDTGNPTEDLKTKSLMKQLLYSPKLQAACPIPFDEAKLWQGQSGPELKQQIQKQ

FKNISALMDCVGCEKCRLWGKLQVLGLGTSLKILFSVDGQDHLDHHLQLQRNEVIALVNL

LNRLSESIKWVREMGPSSEKIMEGRVFAHTDQSSTWQRIWTSLARPRARQNTADHNEKDV

RKTDRWSRFWGMVWFLAREAKLHVGLIQGLKE

>A0A438CH07_VvERO1

MAESREKKESRRQVWRWSLGAPLLAVLVATAITSIAFPEISLFNGNTNDNRKFCHCAQDS

LKYTGIVEDCCCHYETVSLNEEVLHPWLQELVTMPFFRYFKNALKVELEIGVFLEIFCDW

LSCGVTALFWPDDGMCKLRDCSVCECPESEFPEPFKSPFKRTLQSDNLACQEGKPQATVD

RTLDSKAFRGWIETDNPWTNDDETDNAEMTYVNLQLNPERYTGYAGPSARRIWDAIYSEN

CPNHSHGETLQEKNLLYKLISGLHSSISIHIAADYLLDETTNLWGQNLGLMHDRVLRYPD

RVRNLYFTFLFVLRAMRKVSIYRISLLEQAEYNTGNHAEDLKTQSLMRQLLCNPKLQAAC

PLSFDEAKLWQGQSGPELKQQFQKQFKHISALMDCVGCEKCRLWGKLQILGLGTALKILF

SVDDQNHTPLQLQRNEVIALVNLLSRLSVSFKLVNEIRPSAEKLMGGQISAAPVLENRPW

QRIWEFMDRLRYFTSLPLHAINSFT

>A0A2P5E9Y6_ToERO

MVGMKGQRKRWGWVLGALIAIFVAVAMTTRTAPHISLFGRTNKPCHCDQDKHKYSGIVED

CCCDYETVDHLNEEVLHPSLQELVKTPFFRYFKVKLWCDCPFWPDDGMCRLRDCSVCECP

ENEFPESFKKPHRALSSDDLVCQEGKPQAAVDRTLDSQAFRGWTETDNPWTNDDETDNTE

MTYVNLQLNPERYTGYTGLSARRIWDAIYAENCPKYPSEELCQEERILYKLISGLHSSIS

VHIAADYLLDETRNMWGQNISLLYDRVLRYPDRVRNLYFTYLFVLRAVTKAADYLENADY

DTGNPIEDLKTKSLMKQLLYNPKLQAACPLPFDEAKLWKGQNGPELKQKIQKQFRNISAL

MDCIGCEKCRLWGKLQVLGLGTALKILFSVDGQENLGQRLQLHRNELIALMNLLNRLSES

VKFVHQMGTSAEITMKGQISAPISPNCPLQRIWTSLFEGPAPSCPLQRLWSSVSNGPTSQ

NCPLKRIWASLAHR

>A0A0B0MYJ7_GaERO1

MVESEVKKIKKGDKMKWRWVIGAFTTILLAIALASRSTPKIPFKSGQFSKSCHCPQDKHR

YSGIVEDCCCDYETVDHLNEEVLHPLLQDLVKTPFFRYFKVKLWCNCPFWPDDGMCRLRD

CSVCECPENEFPELFKKPYHHGLPSDDLKCQEGKPQAAVDRTLDSKAFRGWTETDNPWTY

DDETDNSEMTYVNLQLNPERYTGYTGPSARRIWDAVYSENCPKYPAEELCQEEKILYKLI

SGLHSSISIHIASDYLLDEATNLWGHNLDLMYNRVLRYPNRVQNLYFTFLFVLRAVTKAA

DYLEQAEYDTGNPTEDLKTHSLMRQLLYNPKLQAACPLPFDEAKLWKGQRGPELKQKIQA

QFKNISALMDCVGCEKCRLWGKLQVLGLGTALKILFSVNGEDHSHQTLELQRNEVIALIN

LLNRLSESIKFVHEMGAAAEKLNEGTVTSTRLNSLVQQAWASIVMIQLQRTASFFQKLD

>A0A1D1YM89_AaERO1

MKKGMEVNGSDRAGVAGGGARWRWRWWWWWAAAMGAVFAVLLATAVASGRIYPVMPLFGS

ASSFCPCRGSQKYTGMVEDCCCDYETVDSLNKEVLHPILQELVASPFFRYFKVKLWCDCP

FWPDDGMCRLRDCSVCECPESEFPEPFKKPFHGLSADNMMCQEGKPQAAVDRTIDTKVFR

GWVEVDNPWTYDDETDNAEMTYVNLQLNPERYTGYSGPSARRIWEAIYTENCPKYPSGET

CPEKKVLYKLISGLHSSISVHIASDFLLDEASNVWGHNLELLYDRVLRHPDRVRNLYFTF

LFVLRAVTKAKDYLEEAEYNTGNSAEDLKTQSLVKQLLYNPKLQAACPLPFDEAKLWQGQ

SGPELMQQIQKQFRNISAIMDCVGCEKCRLWGKLQVLGLGTALKILFSVDGQNHLKHHLQ

LQRNEVIALVNVLNRLSESVKFVREMGPAAEKMMGVRMPSAFHESITWLKGLLKL

>A0A2G3C2W4_CpcERO1

MVEESEVKKKENERSSNGRKLGGSRIGAILVLIIAVGVTFLYTHKGKSCPCIQDSIKYTG

IVEDCCCDYETVDTINGAVLHPLLQELVTTPFFRYFKVKLWCDCPFWPDDGMCKLRDCSV

CECSENEFPESFRRLPLPADDLKCQEGKPEAAVDRTLDSKVFRGWIEVDNPWTNDDETDN

GEMTYVNLLLNPERYTGYTGPSARRIWDAVYSENCPKYASGEICQEKKVLYKLISGLHSS

ISIHIAADYLLDETKNLWGTNPDLMYDRVLQYPERVRNLYFTYLFVLRAVTKAKDYLEQA

EYDTGNPEEDLKAQSLMRQLLYNQKLQAACPVPFDEAKLWKGQSGPELKQQIQKQFRNIS

AIMDCVGCEKCRLWGKLQVLGLGTALKILFSVDGESRHDQHLQLQRNEVIALVNLLNQLS

ESIELVQEMSPTFEKSTKGLSLQPAAKLISSWKRLWETVVGDRQTSLSLVCGSLF

>A0A1J6IN36_NaERO1

MVEETEVNELKKKGNETRRSQRKLGRSAIWAILVLIIALCAAFFHTQKGKPCPCFQDSRK

YTGIVEDCCCDYETVDTINGAVLHPLLQGLVTTPFFRYFKVKLWCDCPFWPDDGMCKLRD

CSVCECPENEFPESFRRPPLGLPADDLKCQEGKPEAAVDRTLDSKVFRGWIEVDNPWTND

DETDNDEMTYVNLLLNPERYTGYTGPSARRIWDAVYSENCPKYASGEICQEKKVLYKLIS

GLHSSISIHIAADYLLDETKNLWGTNPDLMYDRVLQYPDRVRNLYFTFLFVLRAVTKAKD

YLEQAEYDTGNPEEDLKAQSLMRQLLYSPKLQAACPVPFDEAKLWKGQSGPELKQQIQKQ

FRNISAIMDCVGCEKCRLWGKLQVLGLGTALKILFSVDGEYRHDKHLQLQRNEVIALVNL

LNRLSESIKLVQEMSPSFEKTIGGLSLQPAAKLISSWKRLWETVVGDRLRKLPF

>A0A3S3MIJ9_CmERO1

MGMKAEGGEEERPMKRRRTWRWAFAALIVVFFAAAFASRNAPKISLFGFTNKPCQCSDSR

KYTGIVEDCCCDYETVDTINEEVLHPILQEAVTTPFFRYFKVKLWCDCPFWPDDGMCRLR

DCSVCECPETEFPEPFKKPFHGLSSDNLICQEGKPEAAVDRTLDNKVFRGWVEIDNPWTY

DDETDNAEMTYVNLQLNPERYTGYAGPSARRIWEAIYRENCPKYPSGEFCPEKRVLYKLI

SGLHTSISVHIAADYLFDETANLWGQNLELLYDRVLRYPDRVKNLYFTFLFVLRAVTKAA

DYLEQAEYDTGNSAEDLKAQSLMKQLLYNQKLRAACPLPFDEAKLWQGQSGPELMQQTQK

QFRNISALMDCVSCEKCRLWGKLQVLGLGTALKILFSVNGHEHLNQSLQLQRNEVIALVN

LLNRLSESVKLVHEWALQLKRSWNNRLPHLLLRRVACSTIHFKVLGPLDFAIK

>A0A1J3K8D9_NcERO2

MAETDAGNVKGKEKGSGKRWVLLIGAIAAVLLAVVAAVFLNTQNSSLCGFTGKICNCRQA

EKQKYIGMIEDCCCDYETVNKLNTEVLHPLLQDLVKTPFYRYFKVKLWCDCPFWPDDGMC

RLRDCSVCECPESEFPEPFKKPLSKDNPVCQEGKPQAAVDRTLDTRAFNGWTVTDNPWTS

EDETNNDEMTYVNLRLNPERYTGYIGPSARRIWDAIYSENCPKYTSEESCQEEKILYKLV

SGLHSSISVHIASDYLLDEATNLWGQNLTLLYDRVLRYPDRIRNLYFTFLFVLRAVTKAE

NYLGEAEYETGNVIEDLKTKSLVKQLVSDPKTKAACPLPFDEAKLWKGQRGPELKQQIQK

QFRNISAIMDCVGCEKCRLWGKLQILGLGTALKILFTVNGEDNLRHSLELQRNEVIALMN

LLNRLSESVKLVHDMSPVAERIAGSQASAENSLWKRAVTFLLAQIKGLSGKSS

>A0A3L6S3R5_PmERO1_X2

MTTKPAPMENGAAAGAGRGNRRRGGRWRYAAAGALLVAVLAAAVSSRSFPGAPSSSPGGC

GCPAARKYTGMVEDCCCDYETVDSINEEVLHPTLQELVKLPFFRYFKVKLWCDCPFWPDD

GMCRLRDCSVCECPDNEFPEPFKKPYNGLSPDSMICQEGKPQAAVDKTLDTKVFKGWVET

DNPWTSDDETDNNEMTYVNLQLNPERYTGYTGDSARRIWDSIYKENCPKYPSEELCHEKK

ALYELISGLHSSISVHIAYDYLLDESTNLWGQNLPLLYDRVLKYPERVQNLYFTYLFVLR

AVTKAADYLEQAEYNTGNPEDDLKTQSLVKQLLYHPKLRSACPKPFDEAKLWQGENGPEL

KQEIQKQFRNISAIMDCVGCEKCRLWGKLQVLGLGTALKILFSVDGDNNLNQPLQLQRNE

VIALFNLLNRLSESVKFVHEKGSSIEEVIKEQSPPTVQKGASKPNLKPGFL

>A0A4U5P6W6_PoaERO1

MVKKNESFKPKQESKKERKRWSSWGLIGALVAVSLAVVVAVTVSPMTASKIGSLINSNNK

SCQCPSSQDSGKYKGVIEGCCCDYESVDSVNGEVLHPLLQELVTTPFFRYFKVKLWCDCP

FWPDDGMCRLRDCSVCECPENEFPEPLKKPFLHGLPADDVACQEGKPQAAVDRTLDSRAF

KGWIETDNPWTNDDETDNDEMTYVNLLLNPERYTGYVGPSARRIWDAVYSENCLKSPLGE

MCQEKMVLYKLISGLHSSISTHIAADYLLDESTNKWGQNPELMYDRVLRYPDRVRNLYFT

FLFVLRAVTKAADYLEQAEYDTGNHTEDLKTESLVRQLLHNPKLQAACPLPFDEANLWQG

QSGPELKQQIQKQFRNISALMDCVGCEKCRLWGKLQVLGLGTALKILFSVDGQNQPSESL

QLQRNEVIALVNLLCRLSESIKYVCEQGPSIEKTMERQISDPSETKYAST

>A0A2P5D1W6_PaERO

MVENGRMTMMMRRTLVGAVAIVFIAAALSSITAPSYLNFSLLFKDPNSCHCPPRDQNKYT

GLIEDCYCDYETVDSINGEFLYPLLQELVTTSFFRYFKVKLWCDCPFWPDDGMCRLRDCS

VCECPENEFPETFKKPWTHGLPSDDLVCQEGKPQSTVDRTLDSRAFRGWIVTDNPWTNDD

ETDNGEMTYVNLQLNPERYTGYTGPSARRIWDAVYSENCPRYSSGEICQEKKVLYKLISG

LHSSISIHIAADYLLDETTNQWGENVELLYDRVLKYPHRVRNLYFTFLFVLRAVTKAADY

LEQAEYDTGNRSEDLKTQSLMKQLLYSHKLQAACPLPFDEAKLWQGQSGPQLRQQIQKQF

RNISALMDCVGCEKCRLWGKLQVLGLGTALKILFSVDADNTPVQPLQLQRNEVIALLNLL

NRLSESVKYVHEKGPSVEKILGEIDHVTYEDSTRKRMWNLIDGLKFRKRL

>A0A199VV84_AcERO1

MVLIAMAKEEHGGDVGGGTRRRRRRWAFTAAAAVGAVVALFLATAASSRSFPKLSISGLS

KRPCECPDRDSRKYAGIVEDCCCDYETVNSINEEVLHPILQELVTTPFFRYFKVKLWCDC

PFWPDDGMCRFRDCTVCECPESEVPEPFRRKSSSGLSADDMICQEGKPQAAVDRTIDSKA

FRGWIEVDNPWTYDDETDDAEMTYVNLQLNPERYTGYSGPSAWRIWDAIYKENCPKYPSE

EFCQEKKLLYKLISGLHSSISVHIASDYLLDESSNLWGHNLELLYDRVLKYPDRVRNLYF

TFLFVLRAVTKAADYLEQAEYDTGNPQEDLKTQSLVRQLVYNPKLQAACPLPFDEAKLWQ

GENGPELKQQIQKQFRNISALMDCVGCEKCRLWGKLQVLGLGTALKIVFSLDDQNQLNQP

LQLQRNEAIALINLLNRLSESVKFVHEMAPTAEKVVEGKISSAEGKSIS

>A0A1J3HHI8_NcERO1

MIMGNGAIKEEESERKSKTWRWPFAALVVVLLAVAVSSRTASNVGFFFTDRNSCSCSLEG

SGKYKGMIEDCCCDYETVDNLNSEVLNPLLQDLVTTPFFRYFKVKLWCDCPFWPDDGMCR

LRDCSVCECPENEFPEPFKRPYNIAGLPSDDLICQEGKPQAAVDRTIDNRAFRGWVETNN

PWTHEDDTDNSEMTYVNLQLNPERYTGYTGPSARRIWESIYSENCPKYSSGEKCPEKKVL

YKLISGLHSSISMHIAGDYLLDESSNQWGQNMELMYDRILRHPDRVRNMYFTYLFVLRAV

TKATAYLEQAEYDTGNNAEDLKTQSLIKQLLYSPKLQTACPVPFDEAKLWQGQSGPELKQ

QVQKQFRNISALMDCVGCEKCRLWGKLQVQGLGTALKILFSVGNQDQTLQLQRNEVIALV

NLLNRLSESVKMVHDMGPDVERLMEDQIAKVSAKPGRLRRIWDLAASFW

>A0A1R3H621_CoERO1

MVESEVKKSEKSEKMRWKWVIGAFIAIIIAIALASRSSPKISLFGQTDKSCSCSQDKHKY

SGIVADCCCDYETVDHINKEVLHPLLQELVKTPFFRYFKVKLWCDCPFWPDDGMCRLRDC

SVCECPESEFPESFKKPYHRGLPSDDLKCQEGKPEAAVDRTLDSKAFRGWTETDNPWTND

DETDNSEMTYVNLQLNPERYTGYTGPSARRIWDAVYSENCPKYPSEELCQEEKLLYKLIS

GLHSSISIHIASDYLLDEATNLWGHNLKLMYDRVLKYPNRVENLYFTFLFVLRAVTKATD

YLEQAEYDTGNPTEDLKTQSLMRQLLYNPKLQAACPLPFDEAKLWKGQRGPELKQQIQAQ

FKNISALMDCVGCEKCRLWGKLQVLGLGTALKILFSVNGQEHLGQELQRNEVIALINLLN

RLSESVKFVHEMGPAAERIGEGKISSPAGLDNPVQKIWESIVKK

>G7IGL6_MtERO

MVNSELKKKEKGFGGKWVWVVIPLIAAIVAISISSRTSSKISLFGVIGKACQCAMGTPKY

SGMVEDCCCDYETVDNLNEEVLYPSLQELVKTPFFRYFKAKLWCDCPFWPDDGMCRLRDC

SVCECPENEFPESFKKPKRLSLNDLVCQEGKPEAAVDRTLDSKAFTGWTEIDNPWTNDDE

TDNDELTYVNLQLNPERYTGYTGTSARRIWDAVYSENCPKYLSQESCQEEKILYKLISGL

HSSISVHIASDYLLDEATNTWGQNLTLMYDRVLQYPDRVRNLYFTFLFVLRAVTKAADYL

EQAEYNTGNPNEDLKTESLIKQLLYKPKLQAACPVPFDEAKLWKGQSGPELKQKIQHQFR

NISALMDCVGCEKCRLWGKLQVLGLGTALKILFSDDGPENMVQTLQLQRNEVIALMNLLN

RLSESVKFVHEMGPTAERITEGHLFGHTKLISSLRKIWSRILQT

>A0A2P2J689_RmERO1

MASEKEKESGKERKDWRWVGIGAIVALVIAVALTSTAAPKISLFGSSNKSCSCSQKYSGM

VEDCCCDYETVNHLNEEVLHPNLQELVKTPFFRYFKVKLWCDCPFWPDDGMCRLRDCSVC

ECPENEFPELFKRPSHHALPADDPVCQEGKPQATVDRTLDGVTFREWTVLDNPWTNDDET

DNAEMTYVNLQLNPERYTGYTGPSARRIWDAVYSENCPKYPSEELCQEERVLYKLISGLH

SSISTHIAIDYLLDESKNLWGENLTLMYDRVLRYPDRVRNMYFTFLFVLRAVMKATNYLE

HANYDTGNAAEDPKTQSMIRELLSNSKLQTACPIPFDEAKLWKGQQGPELKQKIQGQFRN

ISALMDCVGCEKCRLWGKLQILGLGTALKILFSDNDHDNLSQNLQLQRNEVIALVNLLNR

LSESVRFVQEKGHDVDMIVGEQISSPTAPRSLWQSVAGPFFKS

>A0A4D6NM82_VuERO1

MVKAEPKKKGRGVPWLWVLISLVAVFVAMAFSSKTSPRISLSGAIDRACQCARGAPKFSG

MVEDCCCDYETVDHLNEEVLNPSLQELVKTPFFRYFKVKLWCDCPFWPDDGMCRLRDCSV

CECPESEFPESFKKPHGLSMNDLVCQESQPQAAVDRTLDSKVFRWTEIDNPWTNDDETDN

DEMTYVNLQLNPERYTGYTGDSARRIWDAVYSENCPKYPSQELCQEEKILYKLISGLHSS

ISIHIAAEYLLDEASNLWGQNLTLMYDRVLKYPDRVSNLYFTFLFVLRAVTKAAEYLEQA

QYDTGNHNEDLKTQSLIKQLLHDPKLQSACPIPFDEANLWRGQSGPELKQKIQQQFRNIS

ALMDCIGCEKCRLWGKLQVLGLGTALKILFSVDGQENSSQTLQLQRNEVIALMNLLNRLS

ESVKFVHEMGPAAADSVMEGHVSAHKTLINAWKNIWSFVSKT

>A0A1U7ZF53_NnERO1

MANSEIGENGRRRRWGWVVGALIAVLLATAATSRSAPKISLFAQNNKDCKCVDSRKYTGI

VEDCCCDYETVDTVNKEVLHPLLKEIVATPFFRYFKVKLWCDCPFWPDDGMCRLRDCSVC

ECPANEFPEPFKKPSRHSLSSDDLICQEGKPQAIVDRTLDTKAFRGWVDIDNPWTKDDET

DNDEMTYVNLQLNPERYTGYSGPSARRIWDAIYMENCPKYPSEEFCAERVLYKLISGLHS

SISIHIAADYLLDEVTNLWGQNLELMYDRVLRYPERVRNLYFTFLFVLRAVTKAEDYLEQ

AEYDTGNPEEDLKTCSLMRQMLYNPKLQAACPLPFDEAKLWQGQSGPELKQQIQKQFRNI

SALMDCIGCEKCRLWGKLQVFGLGTALKILFSVDGHDKLDQPLQLQRNEVIALINLLNRL

SESVQFVHEMGPVAENIIEAQISAPSAVSRTWQRILAFVRKQ

>A0A1S3TYB1_VrERO1

MVKTEPKKKGWGVPWLWVLMPLVAVFVAMAVSSKTSPRISLSGAIDRACQCARGAPKFSG

MVEDCCCDYETVDHLNEEVLNPSLQELVKTPFFRYFKVKLWCDCPFWPDDGMCRLRDCSV

CECPENEFPESFKKPHAFSMNDLVCQEGKPQAAVDRTLDSKVFRGWTEIDNPWTNDDETD

NDEMTYVNLQLNPERYTGYSGASARRIWDAVYSENCPKYPSQELCQEEKILYKLISGLHS

SISVHIAADYLLDEASNLWGQNLTLVYDRVLKYPDRVSNLYFTFLFVLRAVTKAADYLEQ

AEYDTGNHNEDLKTQSLIKQLLHDPKLQAACPIPFDEANLWRGQSGPELKLKIQQQFRNI

SALMDCIGCEKCRLWGKLQVLGLGTALKILFSVDSQENSSQTLQLQRNEVIALMNLLNRL

SESVKFVREVGPAADIVMEGHVSAHQTLISAWKKIWSFVSKT

>A0A151S2W1_CacERO2

MVKKEVEKKGCGTRWLWVVVALVAVFVAMAMSSKTSPRIALFGAIDRACQCARGTPKYSG

MVEDCCCDYETVDRLNEEVLHPSLQDLVKTPFFRYFKVKLWCDCPFWPDDGMCRLRDCSV

CECPENEFPESFKKPHRLSMNDLICQEGKPEAAVDRTLDSKAFRGWTEIDNPWTNDDETD

NDEMTYVNLQLNPERYTGYTGPSARRIWDAVYSENCPKYPSQELCQEEKILYKLISGLHS

SISIHIASDYLLDEATNLWGQNLTLMYDRVLKYPDRVRNLYFTFLFVLRAVTKAADYLDQ

AEYDTGNPTEDLKTQSLIKQLLYNPKLQAACPIPFDEANLWKGQSGPELKQKIQQQFRNI

SALMDCVGCEKCRLWGKLQVLGLGTALKILFTVDGQENSSQTLQLQRNEVIALTNLLNRL

SESVKFVHEVGPTAEGIMEGHLSAHTTLINSWKKIWSYVSKT

>A0A2Z7CTG0_DhERO1

MAEAKAVVDNNEKVKKLMEEKVGKRKWALIGALVVLMVAVSLTLKHSRDNKSCHCSQDSR

KYTGIVEDCCCDYETIDSVNEAVLHPLLQELVKTPFFRYFKVKLWCDCPFWPDDGMCKLR

DCSVCECPENEFPEPFKKAMLNGLPSDDLKCQEGNQQDVVDRTLDTKAFMGWIEVDNPWT

HDDETDNREMTYVNLQLNPERYTGYTGASARRIWDAIYSENCPKYSSGEICPEKRVLYKL

ISGLHSSISIHIASDYLIDEIKNLWGRNLELMYDRVFRFPDRVRNLYFTFMFVLRAVAKA

APYLEHAEYNTGNLEEDLKAQSLMRQLVYNPKLQAACPLPFDEAKLWQGQSGPELKLEIQ

KSFKNIRQVNSKFCALMDCVGCEKCRLWGKLQVLGLGTALKILFSVDGENQQNFPVQLQR

NEVIALVNLLHRLSESVKLVQELGPSVEQTVDGLMSLSTSN

>A0A2I0VLL7_DcERO1

MGKMEAAGNGIGGGRCRRWGWAAAALFSVLIATAVIPRRAFFFRISKQACDLADSRKYTG

IVEDCACGYETVDSLNKEVLHPILQELVATPFFRYFKVKLWCDCPFWPDDGMCRLRDCSV

CECPDNEFPEPFKKPFAGLSADDLICQEGKPEAVVDRTLDRKAFRGWVVIDNPWTNDDET

DNAEMTYVNLQLNPERYTGYVGPSARRIWDAIYTENCPRYSSGEICPEKRVLYKLISGLH

ASISVHIASDYLLDEATNMWGQNLELLYERVLRYPDRVRNLYFTFLFVLRAATKAADYLE

QAEYSTGNPDEDLKTQSLVRQLVYNPELQAACPVPYDEAKLWQGESSPELKQQIQKQFRN

ISALMDCVGCEKCRLWGKLQVLGLGTALKILFSVNGQNHLNQPLQLQRNEVIALVNLLNR

LSESVNFVREMGPSAENIMERRPSSPARKTPL

>W9QC89_MnERO1

MAGIQTKRWAWVVGALVAIFVAVAMTSRTAPQISLFGQSNKPCRCDRDMRKYSGIVEDCC

CDYETVDRLNEEVLHPSLQELVKTPFFRYFKVKLWCDCPFWPDDGMCRLRDCSVCECPDH

EFPESFKNSYQGLSSEDLVCQEGKPQAAVDRTLDSKAFRGWTVTDNPWTNDDETDNDEMT

YVNLQLNPERYTGYTGPSARRIWDAIYSENCPKYPSEELCQEERILYKLISGLHSSISIH

IAADYLLDEATNSWGRNISLLHDRVLRYPDRIRNLYFTYLFVLRAVTKAADYLEYAEYDT

GNPVEDLKTQSLMKQLLYNPNLQAACPLPFDEAKLWKGQSGPELKQKIQKQFRNISALMD

CVGCEKCRLWGKLQVLGLGTALKILFSVDGHEKLGQNLQLQRNEVIALVNLLNRLSESVK

LVHDMGQSAEMTMEGQIGTCKFKETHCLTE

>M8D1V8_AetERO2

MSEGPPPEADGAVRRRRRRWAAAAGALLVALLAVALSSRGFPSISSLSRGGGCGCPGARK

YTGMVEDCCCDYETVDAINEEVLNPILQDLVALPFFRYFKVKLWCDCPFWPDDGMCRLRD

CSVCECPDNEFPEPFKKPYSGLSPENMICQEGKPEATVDRTLDTKVFKGWVETDNPWTSD

DETDNAEMTYVNLQLNPERYTGYTGDSARRIWDAIYKENCPKYPSEDMCQEKKALYKLIS

GLHSSISVHIAYDYLLDESANLWGHNLSLLHDRVLKYPERVENLYFTYLFVLRAVTKAAD

YLEQAEYNTGNPEEDLKTQSLVRQLLYNHKLRSACPLPFDEAKLWQGENGPELKQEIQKQ

FRNISAIMDCVGCEKCRLWGKLQVLGLGTALKILFSVDGENNLNQQFQLQRNEVIALVFC

KDARGQVVSITPLLHTGRVMYMQGAAS

>A0A2I0BCL3_AsERO1

MGNMDVVGGAEAGGRCWRWGWAAVALIAVLLATGALPRKNFIRNPRRACELTESRVYTGI

VEDCACDYETVDSLNEEVLHLILQEIVATPFFRYFKVKLWCDCPFWPEDGMCRLRDCSVC

ECPDNEFPEPFKKPFTGLPADDLICQEGKPEAVVDRTLDSKAFRGWVEIDNPWTNDDETD

NAEMTYVNLQLNPERYTGYVGRSAQRIWEAIYIENCPKYPSEEFCQEKKILYKLISGLHA

SISVHIASDYLLDEATNLWGQNLELLYERVFRHPDRVKNLYFTFLFVLRAVTKAADYLEQ

AEYNTGNPEEDLKTQSLVRQLVYNPKLQAACPVPFDEAKLWQGEIGPELKQQIQKQFKNI

SALMDCVGCEKCRLWGKLQVLGLGTALKILFSVDGQSHVNQSLQLQRNEVIALVNLLNRL

SESVKFVREVGPSSDKIMERMPSSS

>A0A4D8ZG61_SsERO1

MAEAKAVGVGKKKHLKKSKEGKGGMRKLAAIGAALVVVVAIAMTYNHSQIHKSCHCSLVL

FVLLFSPFLFISEMVEWGIVQRTFMYTIAGSRRTKSSFFYLLIMQDARKYTGIVEDCCCD

YETVDSLNEGVLHPLLQDLVRTPFFRYFKVKLWCDCPFWLDDGMCKLRDCSVCECPENEF

PELFKRPIQYGLSSDDLKCQEGKPQAAVDRTLDSKAFRGWIEVDNPWTLDDETDNSGMTY

VNLQLNPERYTGYTGPSARRIWDAIYSENCPKYQSGEICQEKKVLYKLISGLHSSISIHI

AAEYLLDEARNLWGRNLELMYDRVLRYPDRVRNLYFTFMFVLRAVTKAANYLEQAEYSTG

NLEEDLKAQSLMRQLLYNPKLQAACPLPFDEAKLWQGQSGPELKLEIQKNFRNISGLMDC

VGCEKCRLWGKLQILGLGTALKILFSVDSKNHPNKPLQLQRNEVIALVNLLHRLAESVKL

VNEIGPSVEKTVEEFTSEPAVQEISLLERARLAVGRIR

**D. Protein disulfide isomerase protein sequences**

>CtPDI1 (ctr16675_c0_g1_i1)

EEVKEDEGVLVLTKGNFKEVTSS

TEFILVEFYAPWCGHCKALAPEYAKAAKKLAESGSAIKLGKVDATEEQELAESHGVRGYP

TLKFFRNGNPVEYSGGRTAEEIVNWLNKKTGPPAKTLATVEEAKAFVEKAASVAVIGFFK

DQASDAAKVFLSVASIVDDYPFAITSSDEVFAEYKVEDGKVVLFKQFDEGRTDFEGELKE

DVLKKFISSNALPLVVEFNHETAQKIFGGEIKSHLLLFFSKEAGHFDEHLENARTVAKEF

RDQLLFVTINADEEDHDRILDFFGMKKAEVPAMRLIRLEEDMAKYKPAEPKLDADSIRAF

VQSFLDGKLKQHLLSQDLPEDWDKNPVKVLVSSNFDEVAFDKEKDVLVEFYAPWCGHCKQ

LAPIFDQLGEKFKDNPNIVVAKMDATINELEHTKIPSFPTLKLYAKGDNKVIEYNGERTL

EGLSKFLETGGVYGQAAPDETEDVDEDDDQPKKDEL

>CtPDI2 (ctr21421_c0_g1_i1)

ASDVVPLTKDTFNDFIKQNDLVLAEFYAPWCGHCQALAPEY

EEAASKLAEKKIPLVKVDCTEEAQLCKKHDVDGYPTLKIFRGSHNFSAYTGARKAPAIVS

YMVKMSLPAVSILNKDTIEDFKKAEQVVLVAYLDMEDKTSNSTYTALAEKLRENYLFGAI

HDSELAKSEGVTFPTIVLYKSFDEGKVIHSGSFDGEEIEKFAKIASTPLIGEIGPETFQS

YMASGIPIAYIFAQTEDLRKSLSDALRPVAEKYKGIVNFATIDAATYGAHAANINLEVDK

FPAFGIHQTSGNKKFPFDQEKEITAETIGNFVSQYVDGKIEPNIKSEPIPESQDGPVKVV

VAKNFDEIVMDDSKDVVLEFYAPWCGFCKALTPKYNILGQLYKDANLSDKVTIAKIDATA

NDFSQNIDGFPTIMLFKAGDKQNPITYDGDREVEELVAFVKKGTHEASVEYTTEMKEKEE

SSISKEQGDENSEKNNDEVEEEHDEL

>CtPDI3 (ctr23276_c0_g1_i1)

DSVLEWGDNDFESGVASHET

ALVMFYAPWCGHCKRLKPEYEKAASLLVGNDPAISLVKVDCTEAGKETCSKFGVTGYPTL

KIFRNGEVSQDYNGPREAAGIVKYMRAQVGPSSVEITAVKAFTDFISKSDVGVIGFFSPD

SALQSTFLKVADKLREKVRFGHTSDKDVFAKQSIKSDSIVLYRPPQMQNKFEPNFVTYDG

DANKDSIEKFINKNYHGLVGHRQRDNMNDFKSPIVVAYYNVDYVKNVKGTNYWRNRILKV

AQNYADSFTFAVSSKDDFQHELNEFGIDYVPGDKPKVSARNAKDQKFIMKDEFSLESFEQ

FLKDLKAGALEPFLKSEPIPEDNSGPVKVAVGKNFDEVVVNNDKDVFIEFYAPWCGHCKK

LAPVWDELGEKLKDEDVEIVKMDASNNDVPSPFEVRGFPTLYWVPKNSKSSPVRYEGGRD

IDDFVSYVAKQATNELKGYNRKGKPTKEEL

>CtPDI4 (ctr23512_c0_g1_i5)

DDVVVLSEDNFEKEVGQDKGALVEFYAPWCGHC

KKLAPEYEQLGSSFKKAKSVLIGKVDCDEHKSLCSKYGVSGYPTIQWFPKGSLEPKKYEG

PRTAESLAEFVNTEGGTNVKIATAPSNVVVLTPENFNEVVLDETKDVLVEFYAPWCGHCK

NLAPTYEKVATAFKSEEDVVIANLDADKYRDLAEKYDVSGFPTLKFFPKGNKAGEEYGGG

RDLDDFVAFINEKAGTSRDVKGHLTSKAGIVESLDVLVKEFVAAGDEEKKAVFARIEEEV

GKLKGSASRHGKVYLKAAKNSIEKGSDYAKNEIQRLQRILDKSVSPAKADELTLKKNILS

TYV

>CtPDI5 (ctr24519_c0_g1_i1)

EEIEKEEEVLVLTTKNFDDAVKQFDYILLEFYAPWCGHCKAL

APEYAKAAQALVKDGSEVKLAKIDATKEPSLAEKHGVRGYPTLKFMKKGNLIDYNGGRQA

DDIINWVTKKTGPPAKELTSVDDAKSLIDAHNVVIVGFFKDAASDAAKVFIDVANSIDDH

VFAITSDDKIFEEYKAEDGKIVLFKKFDEGKVVFDGEFVADKVKEFITVESLPLIVDFNQ

ETAQKIFGGEIKSHLLLFLSKKEGHYESYVEGVKETAQKYKGQILFVTIDGDETDHERIL

EFFGMKKDNVPAMRIIKLEQDMSKYKPETDKLDAESIMKFVTDFAEGNLKRHLLTQDLPE

DWDKESVKILVGTNFADIALDKTKNVLVEFYAPWCGHCKQLEPIYKELGDKFASMDDVVI

AKMDSTANELEEVKVNSFPTIYLYKKETNEAIEYSGERTLEGLSKFVESGGEVGKAPEEA

QDEDEDDDVPIKEEL

>CtPDI6 (ctr24519_c0_g1_i2)

CKKMKPEYEKAAKMMVDKKIPGKLAAVDTTVEQSLGKRYKVQGYPTIKLFLDGEFKFDAN

VRDSEKIVALMTDPTKAPPVPEEEKPWSAVPSEVVHLTTDDFKQVLKKKRHALVMFYAPW

CGHCKALAPEYAKAAQALVKDGSEVKLAKIDATKEPSLAEKHGVRGYPTLKFMKKGNLID

YNGGRQADDIINWVTKKTGPPAKELTSVDDAKSLIDAHNVVIVGFFKDAASDAAKVFIDV

ANSIDDHVFAITSDDKIFEEYKAEDGKIVLFKKFDEGKVVFDGEFVADKVKEFITVESLP

LIVDFNQETAQKIFGGEIKSHLLLFLSKKEGHYESYVEGVKETAQKYKGQILFVTIDGDE

TDHERILEFFGMKKDNVPAMRIIKLEQDMSKYKPETDKLDAESIMKFVTDFAEGNLKRHL

LTQDLPEDWDKESVKILVGTNFADIALDKTKNVLVEFYAPWCGHCKQLEPIYKELGDKFA

SMDDVVIAKMDSTANELEEVKVNSFPTIYLYKKETNEAIEYSGERTLEGLSKFVESGGEV

GKAPEEAQDEDEDDDVPIKEEL

>CtPDI7 (ctr24519_c0_g2_i1)

AEEDVLELTDSDFTATLDTYDNTLVMFYAPWCGHCKHLKPEFA

KAAGELKRNDPPITLAKVDCTEAGKDTCGKYSVSGYPTLKIFSKSEMISDYNGPREADGI

VKYMKSQVGPASKELKGEDCHKNFLETNEVSYILYTEKEDSPLAKAFHSVAKKLREKARF

AHTIAKSLVEKDGFKNKIVLYRPKILQNKFEESILHYDGEANVADISDFVTKNYFGLAGI

RTRDNAPSFVNPLVVAYYSVDYVKNPKGTNYWRNRILKASKNYKSFNYAISNKDDFQHEL

NDFGVTFAKDDKPVILARDEKNLKYVMKDEFNVENFEKFLKDFDNNVLEPFIKSEPIPED

NSAPVKVVVGKNYDDIITNSDKDVLIEFYAPWCGHCKKLAPIFDELGEKLVDENVVIAKF

DATANDVPVQFEVRGFPTLYWIPKDAKSSPVKYEGGRELNDFVQYIAKHSTDGLKNFDRK

GKAKVAKEEL

>CtPDI8 (ctr24519_c0_g3_i1)

MYSSTSDVVELTPSNFDKLVINSDQIWVVEFYAPWCGHCQSLTP

EYNKAATALKGVVKVGAVNADDHKSLGGQYGVRGFPTIKIFGANKKKPEDFNGARTAAGI

VDGALSAAKDKVKAVLGGKKSDSKKSKGSSDDVIELTDDNFDKLVLNSDDIWLVEFFAPW

CGHCKNLAPEWASAASELKGKVKLGALDATVHTSKASEYNVRGYPTIKYFAGGKKDSDSV

ADYDGGRTSQAIVSWALDKFNDNLPAPDVIQLTGKQSMKDACDDKPLCVVSVLPHILDCQ

SKCRNDYLNTLKTLAEKYKQKMWGWVWAEAGTQPEVEEALEIGGFGYPALAAVNARKMKF

SLLKGSYSFDGINSFLRDLSYGRGGTAPLRGAELPKIHETEAWDGKDGVPPQEEEIDLSD

VDLDEKEEL

>CtPDI9 (ctr26632_c5_g4_i3)

DAGVQEQVAVESDEGVLVLTKDNFQSIVSSSEYLLVKFYAPW

CGHCKQLAPEYANAAQHLTQNELSVKLGKVDATIESDLAEQFGIRGYPTLKFFKNGKPID

YSGGRTKDEIVQWVLKKSGPAAKALQSEEELQSFIEGKNVAIVGYFENLESEAAKLFSEL

ADSVDDHPFGLVSDYSKFSSLEHKDTFVLYKDFDEKKVPFDKDIANVEDIKKFIFVHSLP

PIIEFNQDTAQKIFGGQIKSHLLLFLSKKEGHFEKFIDDIKPVALDFRGKIVIVTIDADE

EEHQRILEFFGMKKNEVPSMRAIKLEDDMTKFKPESPELTGENVRKFVSDFVEGKVKQHL

LSEELPEDWNKTPVWTLTATNFDSVALDSTKNVLVEFYAPWCGHCKQLAPIFDKVGEYFA

DKDDIVIAKMDATVNELEHTKISSFPTLTFYPKGDSPKAIEYNGDRTLEAIIKFIEADGK

QESSSSTTSEEEEEEEEREKPKDEL

>CtPDI10 (ctr28710_c1_g1_i4)

DDKDEDGLEELLAVDDEVEQEAEVGGVKLSE

AEVLSKAQRIVLELNNENTERVVTGNEFVLVLGYAPWCPRSAELMPRFAEAATSLKELGT

PLLMAKLDADRYPKSASFLGVKGFPTLLLFVNGTSQPYSGGFTADDIVIWARKKTGIPVI

RISSVAEAEEFLKKYQTFLIGRFDKFEGPDYEEFVSAAKSDNETQFVEVSQVELAQFLYP

DIKPTDHFLGIVKSEPERYTAYDGAFTLNKILQFIDYNKFPLVTKLTEMNSIRVYSSPIK

LQVLVFANVDDFKNLLDPLQDIARNFKSKIMFLYVDINDENLAKPFLTLYGLEESKNTVV

AAFDNGMSSKYLLESEPTRSNIEEFCNKLVQGSLSPYFKSQPIPDNTEASVHVIVGKTFD

DEILSSKKDVVLEVFTPWCINCEATSKQVEKLAKHYKGSTNLIFARIDASANEHPKLQVD

DYPTLLLYTANDKMDPIKLSTKSSLKELAASISKHLKVKNQVVKDEL

>CtPDI11 (ctr29427_c2_g3_i1)

DSVVDLSDSDFDSSVAEYDTSLVMFYAPWCGHCKKLKPEF

EKAAKTLLKEDPPVTLAKVDCTEAGKEVCNKFGVSGYPTIKIFRNGEVSKEYNGPRDAAG

IVKYMKSQVGPSSKDLSSEDIIKNFLSKDDVVVVGFFETETDLKGKFVQLANKLREKVNF

GHTTSQSVIDKYNYKNNVVLYRPKHLSNKFEPDFVVYDGEETTSALEAWITSNYHGLVGY

RQKENMEAFKPPYVGVYYAVDYVKNPKGTNYWRNRVLKVAKSVKDVTLAINNKDDFQHEI

NEYGLEFVSDDKPIVLARSLDNKKYIMKDEFSVDNLEKFVKNFQNGNLEPYIKSEAVPED

NTTPVKVAVAKNFDDLVINNGVDTLVEFYAPWCGHCKSLAPVYEQVAEKLKDEAVSLVKM

DATANDVPSTFDVRGFPTLYWLPKDSKNKPIRYEGGRDVNDFIKYIASKATDELHGFDRS

GNPKDGKDEL

>CtPDI12 (ctr29427_c2_g3_i2)

DSVVDLSDSDFDSSVAEYDTSLVMFYAPWCGHCKKLKPEF

EKAAKTLLKEDPPVTLAKVDCTEAGKEVCNKFGVSGYPTIKIFRNGEVSKEYNGPRDAAG

IVKYMKSQVGPSSKDLSSEDIIKNFLSKDDVVVVGFFETETDLKGKFVQLANKLREKVNF

GHTTSQSVIEKYNYKNNVVLYRPKHLSNKFEPDFVVYDGEESTSALESWITSNYHGLVGY

RQKENMEAFKPPYVGVYYAVDYVKNPKGTNYWRNRVLKVAKSVKDVTLAINNKDDFQHEI

NEYGLEFVSDDKPIVLARSLDNKKYIMKDEFSVENLEKFVNDFQDGNLEPYIKSESIPED

NTTPVKVAVAKNFDDLVINNGVDTLVEFYAPWCGHCKSLAPVYEQVAEKLKDEAVSLVKM

DATANDVPSTFDVRGFPTLYWLPKDSKNKPIRYEGGRDVNDFIKYIASKATDELHGFDRS

GNPKDGKDEL

>CtPDI13 (ctr30222_c3_g6_i1)

LYGPSSPVLQLNPSNFKSKVLNS

NGVVLVEFFAPWCGHCQALTPIWEKAATVLKGVATVAALDADAHQSLAQEYGIKGFPTIK

VFVPGKPPVDYQGARDVKPIADFAFQQVKALLKDRLNGKTTGGSNEKTETSASVELNSRN

FDELVLKSKELWVVEFFAPWCGHCKKLAPEWKKAANNLKGKVKLGHVDCDAEKSLMSRFNVQGFPTILVFGADKDTPIPYEGARTALAIESFALEQLETNVAPPEVTELHSPDVLEEKCG

SASICFVSFLPDILDSKAEGRNKYLQQLLSVAEKFKRSPYSYVWVAAGKQPDLEKHVGVG

GYGYPALVALNLKKGVYAPLKSAFELDHIMEFVKEAGRGGKGNLPLQGTPTIVKTEPWDG

KDGEIIEEDEFSLEELMGEEASSKDQI

>CtPDI14 (ctr39989_c0_g1_i1)

LYPSSSDVVELTPDNFDRKVIQSDEVWIVEFFAPWCGHCRNL

VPEYTKAASQLKGVVKVGAVDADQHKSLGGQYGVRGFPTIKIFGANKRKPEDYNGPRTAK

GLVDGALDAIRKKIESQGGSGGSSSGGGSKDNKDVVELTDSNFEETVLKSEDMWLVEFYA

PWCGHCKNLAPHWAAAATELKGKVKLGALDATVHQLTASKYRVEGYPTIKYFAGGKKDAD

SVQDYNGGRVTKDIVEWAMEKVLENIKPPEIKQLVSEAVLKEACEEHPLCVVSVLPHILD

CQSKCRNDYLSILKTMGEKYKNKGWGWVWSEAGAQTDLEGALDIGGFGYPAMAVVNTKKM

KYSILKGSFSSDGIKEFLRDLSYGRGSTAPVKGASLPKTNTVDPWDGKDGELPPEEDIDL

SDVELDDLPKDEL

>CtPDI15 (ctr6173_c0_g1_i1)

IYPSNSDVIELTDDNFNQVLQSVEIWVVEFYAPWCGHCQRLV

PEYSKAAKALKGIVKVAAIDADKYPSFAGRYGVQGFPTVKIFVDKNKPQDFTGDRTAVGI

TDEVTKAIKNAISANLQGVPYGSSKSSKKSSSGDDVVELTDSNFDKLVLNSDDIWLVEFF

APWCGHCKNLAPHWAAAASELKGKVKLGALDATVHSSKAQEFNIRGYPTIKFFPSGTSSS

SGAEEYTGGRTSSDIVSWAMQKHQENVPPPDIIEIVNEDTFKAGCSEHALCVVSVLPHIL

DCQASCRNEYLNTLRSLGDKFKQKLWGWLWAEAGKQPELESTLEIGGFGYPALAVLNVKK

MKYSILRGSFSEDGIKEFLRDLSYGRGTTAPVKGAALPEIQATEPWDGKDGELPTADDID

LSDVDLDDLPKEEL

>Cg_csPDI_ AMM62654.1

MKFATVFSLTLLAFVACEEVEQEEKVYVLKKKNFDNFIKENEFVLVEFYAPWCGHCKKLAPMYSEAAGKLMDEGSNIKLAKVDATVETDLAGKFEVKGFPTIKFFIDGESVDYTGGRQTSDIINWLKKKTGPPAKDVKTS

EEAKTFIDSDEVIVMGFFKDQEGKDAAAFKKTASKIEDVAFGITSEDSVFKEHKMKKDGVVLFKKFDEGRNDFSGDFEEAAMSKFVKDNRLPLINEFTQETAQKIFAGDIQSHLMLFVKKEEAKDTLDTFKAAAGEFKGKVLFIYLDTTKEENEHIMGFFGLKAADAPAMRLIQLGEDLAKYKPESDSLDKSTVTKFVQDFLDGKLKPHL

KSEEVPEDWDAQPVKVLVSKNFKEVAMDKSKAVFVEFYAPWCGHCKKLAPIWDQLGEKFKDSKDIIIAKMDSTTNELEEVQIKSFPTLKYFSKGSNEIIEYDGERTLEELTKFVESGGKQEPPKKEEEEKEEDDDKKKDEL

>Cg_PDI_ AMM62646.1

MKFSSCLVLTLLVFVSAEDVKQEEGVYVLTAKNFDSFIADNEFVLVEFYAPWCGHCKALAPEYAKAATTLEEEKLNIKLGKVDATVEESLAAKFEVRGYPTIKFFRKEKPDGPADYSGGRQAADIVSWLKKKTGPPAKELKEKDEVKSFVEKDEVVVIGFFKDQESTGALAFKKAAAGIDDIPFAITSEDHVFKEYKMDRDGIVLLKKFD

EGRNDFDGEFEEEAIVKHVRENQLPLVVEFTQESAQKIFGGEVKNHILLFLKKEGGEDTIEKFRGAAEDFKGKVLFIYLDTDNEENGRITEFFGLKDDEIPAVRLIQLAEDMSKYKPESSDLETATIKKFVQDFLDGKLK

PHLMSEDVPDDWDAKPVKVLVGKNFKEVAMDKSKAVFVEFYAPWCGHCKQLAPIWDELGEKYKDSKDIVVAKMDATANEIEEVKVQSFPTLKYFPRDSEEAVDYNGERTLDAFVKFLESGGTEGAGVPEDEEEEEEDEEG

DDEDLPRDEL

>TaPDIL1-1_CAI30635.1

EEAAAAEEAAAAPEAVLTLHADNFDDAIAKHPFILVEFYAPWCGHCKSLAPEYEKAAQLLSKHDPAIVLAKVDANDEKNKPLAGKYEVQGFPTLKIFRNGGKNIQEYKGPREAEGIVEYLKKQVGPASKEIKAPEDATYLEDGKIHIVGVFTEFSGTEFTNFLELAEKLRCDYDFGHTVHANHLPRGDAAVERPLVRLFKPFDELVVDSKDFDVSALEKFIDASSTPKVVTFDKNPDNHPYLLKYFQSNAPKAMLFLNFSTGPFESFKSAYYGAVEEFSGKDVKFLIGDIEASQGAFQYFGLKEDQAPLILIQDSDSKKFLKEQVEAGQIVAWLKDYFDGKLTPFRKSEPIPEANNEPVKVVVADNIHDVVFKSGKNVLIEFYAPWCGHCKKLAPILDEAAATLQSEEDVVIAKIDATANDVPGEFDVQGYPTLYFVTPSGKKVSYEGGRTADEIVDYIKKNKETAGQAAAAATEKAAEPAATEPLKDEL

>TaPDIL2-1_CBG76696.1

AVPTSNPDIDLEYLIKNAGLDDPTPATTATDPEDDGAPDFPGLDADYDDEVLFGDDDGPEEDSSHPSAADEAHVLLLTAANFTPVLAARRHVMVEFYAPWCGHCRALAPHYAAAASALAEQGVDVALAKVDATEDHDLAQAHGVQGYPTLLFFIDGVPRDYAGERTKDAIVAWTSKKLGPAVQNLTTADEAEKIVTGDDVAVLAYLDHLSGAHSDELAAASRLEDTISFYQTTSPDVAKLFHIDPEAKRPSVVLLKKEEEKLTVFDGEFRASAIAEFVSANKIPLITTLTQETAPAIFDNPIKKQILLFAVAKESPQFLPIIKETAKSFKGKLLFVFVERDNEEVGEPVANYFGIAGQETTVLAYTGNEDAKKFFFSGEISLDTIKEFAQGFLEDKLTPSYKSDPVPESNDEDVKVVVGKSLDQIVLDESKDVLLEVYAPWCGHCQSLEPIYNKLAKYLRGIDSLVIAKMDGTNNEHPRAKPDGFPTILFYPAGKKSFEPITFEGDRTVVEMYKFLKKHAAIPFKLKRPDSSAARTDGADGSGSTTEGEKSSGSNPKDEL

>TaPDIL3-1_CBG91897.1

AKLDLDEVDDSEVLEALLAVDEEEEDAAPPGGGGGAEAVRRTQSMVLVLDNDNAARAVRDHPELLLLGYAPWCERSAKLMPRFAEAAAALRAMGSAVAFAKLDGERFPKAASTVGVNGFPSVLLFVNGTEHAYTGLHTKDAIVTWVRKKTGTPVIRIESKDSAEELLKKGQTFALGLFKNYEGTDHEEFMKAATAENEVQFVETNDRNVAKILFPGIASEEQFLGLVKSEPEKFEKFDGAFEEKEILQFVELNKFPLITVFTDLNSAKVYSSPIKLQVFTFAEAYDFEDLESIVQEVARGFKTKIMFIYVDTAEENLAKPFLTLYGLEGDKPTVTAFDTSKGAKYVLEADINAKNLKEFSLSLLDGTLPPYFRSEPVPQEEGLVEKVVGRTFDSSVLQSPHNILLEAHAPWCVDCEAISKNIEKLAKHFSGLDNLKFARIDASVNEHPKLQVNNYPTLLLYPAEDKTNPIKLSKKLSLKDMARFLKEKLQISDVEIKEKLQTPNIETVAAADNVKDEL

>TaPDIL4-1_CBG91898.1

DGDEVLALTESTFEKEVGQDRGALVEFYAPWCGHCKKLAPEYEKLAASFKKAKSVLIAKVDCDEHKSVCSKYGVSGYPTIQWFPKGSLEPKKYEGQRTAEALTEYVNSEAATNVKIAAVPSSVVVLTEETFDSVVLDETKDVLVEFYAPWCGHCKSLAPIYEKVASVFKQDEGVVIANLDADKYTSLAEEYGVSGFPTLKFFPKGNKAGEEYESGRELDDFVKFINEKSGTSRDSKGQLTSEAGLVASLDALVKEFHSAADDKRREILSKIEEEAAKLSGPAVKHGKIYVNVAKKILQKGSDYTKKETERLHRLLEKPISPSKADEFAIKKNILSAFSS

>TaPDIL5-1_CBG91899.1

LYSAGSPVLQLNPNNFKKVLNANGVVLVEFFAPWCGLCKQLTPIWEKAAGVLKGVATVAALDADAHKELAQQYGIRGFPTIKVFLPGKPPVDYEGARDVKPIVNFALSQVQGLLRDRLDGKTSGGSSGKTSGGSSEKKNEPNESVELNSSNFDELVVRSKDLWIVEFFAPWCGHCKKLAPEWKRAAKNLKGQVKLGHVDCDSDKSLMSKYKVEGFPTILVFGADKESPFPYQGARAASAIEPFALEQLEANAAPPEVSELTSADVMEEKCASAAICFVSFLPDILDSKAEGRNKYLELLLSVAEKFKKSPYSFVWAGAGKQADLEKQVGVGGYGYPAMVALNVKKGAYAPLRSAFELAEITEFVKEAGRGGKGNLPLEGAPTVVESEPWDGKDGEVIE

EDEFSLEELMADSSAPNDEL

>OsPDIL1-1_Os11g09280.1

EEAAAAEEGGDAAAEAVLTLDADGFDEAVAKHPFMVVEFYAPWCGHCKKLAPEYEKAAQELSKHDPPIVLAKVDANDEKNKPLATKYEIQGFPTLKIFRNQGKNIQEYKGPREAEGIVEYLKKQVGPASKEIKSPEDATNLIDDKKIYIVGIFSELSGTEYTNFIEVAEKLRSDYDFGHTLHANHLPRGDAAVERPLVRLFKPFDELVVDSKDFDVTALEKFIDASSTPKVVTFDKNPDNHPYLLKFFQSSAAKAMLFLNFSTGPFESFKSVYYGAAEEFKDKEIKFLIGDIEASQGAFQYFGLREDQVPLIIIQDGESKKFLKAHVEPDQIVSWLKEYFDGKLSPFRKSEPIPEVNDEPVKVVVADNVHDFVFKSGKNVLVEFYAPWCGHCKKLAPILDEAATTLKSDKDVVIAKMDATANDVPSEFDVQGYPTLYFVTPSGKMVPYESGRTADEIVDFIKKNKETAGQAKEKAESAPAEPLKDEL

>OsPDIL1-2_Os04g35600.1

VDATEELKEAVLTLDAGNFSEVVAKHPFIVVKFYAPWCGHCKQLAPEYEKAASILRKNELPVVLAKVDAYNERNKELKDKYGVYSYPTIKIMKNGGSDVRGYGGPREADGIVEYLKRQVGPASLKLESAEEAAHSVVDKGVILVGVFPEFAGMEYENFMVVAEKMRADYDFFHTSDASILPRGDQSVKGPIVRLFKPFDELFVDSEDFGKDALEKFIEVSGFPMVVTYDADPTNHKFLERYYSTPSSKAMLFVSFGDDRIESFKSQIHEAARKFSGNNISFLIGDVADADRVFQYFGLRESDVPLLFVIASTGKYLNPTMDPDQIIPWLKQYIVEYGNLTPYVKSEPIPKVNDQPVKVVVADNIDDIVFNSGKNVLLEFYAPWCGHCRKFALILEEIAVSLQDDQDIVIAKMDGTVNDIPTDFTVEGYPTIYFYSSSGNLLSYDGARTAEEIISFINENRGPKAGAAAAVDEKTQIDAVEEEVTSSSEPVKDEL

>OsPDIL1-3_Os02g34940.1

ASSTAFAAAFALLLLASSAAAEGEAVLTLDAGNFTEVVGAHDFIVVEFYAPWCGHCNQLAPEYEAAAAALRSHDPPVVLAKVDASADLNRGLAGEHGVQGYPTIRILRDRGARSHNYAGPRDAAGIVAYLKRQAGPASVEIAASASPPAADSIANDGVVVVGVFPELSGSEFESFMAVAEKMRADYDFRHTTDAGVLPRGDRTVRGPLVRLFKPFDELFVDSQDFDRDALEKFIESSGFPTVVTFDTSPANQKYLLKYFDNAGTKAMLFLSFSDDRAEEFRTQFHEAANQYSANNISFLIGDVTASQGAFQYFGLKESEVPLVFILASKSKYIKPTVEPDQILPYLKEFTEGTLAPHVKSEPIPEVNDQPVKTVVADNLREVVFNSGKNVLLEFYAPWCGHCQKLAPILEEVAVSLKDDEDVVIAKMDGTANDVPSDFAVEGYPSMYFYSSGGNLLPYDGRTAEEIIDFITKNKGSRPGEATTTESVKDEL

>OsPDIL2-1_Os02g01010.1

SDDDLDYLIDNADDIPANDPDGWLQEGSPDDDDDDDLFHHGQAQDHPIDETHVFLLSAANFSDFLASHRHVMVEFYAPWCAHCQALAPDYAAAAADLSPLAHQVALAKVDATEDTDLAQKYDVQGFPTILFFIDGVPKDYNGARTKEAIVSWVNKKLAPGVQNITTVDEAEKILTGEDKAILAVLDSLSGAHSDEIAAASRLEDAINFYQTSNPDVAKLFHLDPAAKRPSLVLLKKQEEEKLTFYDGPFKASAIADFVSANKLPLVNTLTQETAPSIFDNPIKKQILLFVVANESSKFLPIFKEASKSFKGKLLFVFVERDNEEVGEPVANYFGITGQETTVLAYTGNEDARNFFLDGEISVENIKRFAEDFLEEKLTPFYKSEPVPESNEGDVKIVVGKNLDQIVLDESKDALLEIYAPWCGHCQELEPTYNKLGKHLRGIDSLVIAKMDGTANEHPRAKPDGFPTILFYPAGKKSFEPITFEGDRTVVEMYKFIKKHASIPFKLKRPDSSATKTEKDQSTASTNLRGERSSGTNFKDEL

>OsPDIL3-1_Os06g06790.1

RLDLDDDGDDSEVLDELLAVDEEEERGELGGGGEAAAAEAVRRAQSMVLVLDNDNARRAVEENAEVLLLGYAPWCERSAQLMPRFAEAAAALRAMGSAVAFAKLDGERYPKAASAVGVKGFPTVLLFVNGTEHQFTGLHTKDAIVTWVRKKTGAPASRIQSKDSAEEFLKKDQTFAVGLFKNFEGAEYEEFVKAATSENEVQFVETNDRNVAKILFPGIASEEQFLGLVKSEPEKFEKFNGAFEEKEIIQFVELNKFPLITVFTDLNSGKVYGSPIKL

QVFTFAEAYDFEDLESMIQEVARGFKTKIMLIYVDTAEEKLAKPFLTLYGLEPEKPTVTAFDTSKGTKYLMEAEINAKNLQDFCLSLLEGTLPPYFRSEPVPEEKGPIEKVVGRTFDSSVLESPQNVFLEVHAPWCVDCEAISKNVEKLAKHFNDLGQTNLKFARIDASVNEHPKLQINNYPTLLLYPAQDKSNPIKLSKKSNLKDMAKFVKEKLQIADVETVAAGDIVKDEL

>OsPDIL4-1_Os05g06430.1

DDVLALTESTFEKEVGQDRAALVEFYAPWCGHCKKLAPEYEKLGASFKKAKSVLIAKVDCDEHKSVCSKYGVSGYPTIQWFPKGSLEPKKYEGQRTAEALAEYVNSEAATNVKIAAVPSSVVVLTPETFDSVVLDETKDVLVEFYAPWCGHCKHLAPIYEKLASVYKQDEGVVIANLDADKHTALAEKYGVSGFPTLKFFPKGNKAGEDYDGGRELDDFVKFINEKCGTSRDSKGQLTSEAGIVESLAPLVKEFLGAANDKRKEALSKMEEDVAKLTGPAANRYGKIYVNSAKKIMEKGSEYTKKESERLQRMLEKSISPSKADEFVIKKNILSTFSS

>OsPDIL4-2_Os01g23740.1

DGDDVVALTESTFEKEVGQDRGALVEFYAPWCGHCKKLAPEYEKLGASFKKAKSVFIAKVDCDEHKSVCSKYGVSGYPTIQWFPKGSLEPKKYEGQRSAEALAEFVNTEGGTNVKLATIPSSVVVLGPDNFDSIVLDENKDILVEFYAPWCGHCKHLAPIYEKLASVYKLDDGVVIANLDADKHKDLAEKYGVSGYPTLKFFPKGNKAGEDYDGGRELDDFVKFINEKCGTSRDTKGQLTSEAGRIASLDALAKEFLGAANDKRKEILSNMEEEVVKLSGSAAKHGKVYIAIAKKILDKGHDYTKKETERLERMLEKSISPSKADEFIIKKNVLSTFSS

>OsPDIL5-1_Os09g27830.1

LYSAGSPVLQFNPNNFKSKVLNSNGVVLVEFFAPWCGHCQQLTPIWEKAAGVLKGVATVAALDADAHKELAQEYGIRGFPTIKVFVPGKPPVDYQGARDVKPIVEFALSQVKALLRDRLNGKTSAGSGGKKSGGSSEKTEPSASIELNSQNFDKLVTKSKDLWIVEFFAPWCGHCKKLAPEWKKAAKNLKGQVKLGHVDCDAEKSLMSKYKVEGFPTILVFGADKESPFPYQGARVASAIESFALEQLEANAAPPEVSELTGPDAMEEKCASAAICF

VSFLPDILDSKAEGRNKYLELLLSVAEKFKKSPYSFVWTAAGKQADLEKQVGVGGYGYPAMVALNVKKGAYAPLRSAFQLDEITEFVKEAGRGGKGNLPLDGTPTIVQSEPWDGKDGEVIEEDEFSLEELMADNSPVNDEL

>HvPDIL1-1_BAJ89205

EEAAAAEEAAAPEAVLTLHADNFDDAIAQHPFILVEFYAPWCGHCKSLAPEYEKAAQLLSKHDPAIVLAKVDANDEKNKPLAGKYEVQGFPTLKIFRNGGKSIQEYKGPREAEGIVEYLKKQVGPASKEIKAPEDATYLEDGKIHIVGVFTEFSGPEFTNFLEVAEKLRSDYDFGHTVHANHLPRGDAAVERPVVRLFKPFDELVVDSKDFDVSALEKFIDASSTPKVVIFDKNPDNHPYLLKFFQSNAPKAMLFLNFSTGPFESFKSAYYGAVEEFSGKDVKFLIGDIESSQGAFQYFGLKVDQAPLILIQDGDSKKFLKEHVEAGQIVAWLKDYFDGKLTPFRKSEPIPEANNEPVKVVVADNVHDVVFKSGKNVLIEFYAPWCGHCKKLAPILDEAAATLQSEEDVVIAKMDATENDVPGEFDVQGYPTLYFVTPSGKKVSYEGGRTADEIVDYIRKNKETAGQAAAATEKAAEPAATEPLKDEL

>HvPDIL1-2_BAJ99269

AEVDATAMPGEAVLTLDAGNFSEVVTKHEFIVVEFYAPWCGHCKELAPEYEKAASVLRKRDPPVVLAKVDAYDESNKELKDKYKVHGYPAIKIIRKGGSDVSAYGGPRDAEGIVEYLMRQVGPASLEIKSAVDASRSIGDKGVVLVGVFPEFAGIEYENFMAVANKMRTDYDFFHTLDASILPRGDLTVKGPLIRLFKPFDELFVDSQDFDSDAIKKFIEVSGFPTVVTFNADPTNHKFIERYYSTPSAKAMLFLRFNDDRVETFKSQMHEAARQLSGNNISFLIGDVSTADRAFEYFGLKESDVPLLLVLASTGKYLNPTMEPDQLIPWMKQYIYGNLTPYVKSESIPKVNDQPVKVVVADNIDEIVFNSGKNVLLEFYAPWCGHCRKLAPILEEVAVLLQDDKDVVIAKMDGTANDIPTDFSVEGYPALYFYSSSGGNLLLYDGPRKADEIISFIKKNRGAKAAAAEVTQMDDVEEEVTSSTPSESVRDEL

>HvPDIL2-1_BAK01309

SSTPTSNPDIDLDYLIKNAGLDDTTTEDAAPDFPGLDADYDDDEEDLFDDDDGPEAESSSAASQDQEAVDEAHVLLLTAANFTSVLAARRHVMVEFYAPWCGHCRALAPHYAAAAAHLALDQPGLDVALAKVDATEDHDLAQAHDVQGYPTLLFFIDGVPRDYAGERTKDAIVAWITKKLGPAVQNLTAVDEAEKIVTGDDVAVLAYLHHLSGAHSDELAAASRLEDTVSFYQTTSPDVAKLFHIDPEAKRPSVVLLKKEEEKLTVFDGEFRASAIAEFVSANKIPLITTLTQETAPAIFDNPIKKQILLFAVAKESSKFLPILKETAKSFKGKLLFVFVERDNEEVGEPVADYFGITGQETTVLAYTGNEDAKKFFFSGEISLDSIKAFAQDFLEDKLTPFYKSDPVPESNDEDVKVVVGKSLDQIVLDESKDVLLEIYAPWCGHCQSLEPIYNKLAKFLHGIDSLVIAKMDGTNNEHPRAKPDGFPTILFYPAGKKSFEPITFEGDRTVVEMYKFLKKHAAIPFKLKRPGSYSSATQTDSTDGPGSSTEAEKSSGSNPKDEL

>HvPDIL3-1_BAJ98166

KLDLDDVDDSEVLEALLAVDDEEEAAPPGSGGGGGGGAEAVRRTQSMVLVLDNDNAARAVQDHPELLLLGYAPWCERSAQLMPRFAEAAAALRAMGSAVSFAKLDGERFPKAAAAVGVNGFPSVLLFVNGTEHPYTGLHTKDAIVTWVRKKTGTPVIRLESRDSAEEFLKKGQTFALGVFKDYEGADHEEFVKAATAENEVQFVETNDRNVAKILFPGIASEEQFLGLVKNEPEMFEKFDGSFEEKEIIQFVELNKFPLITVFTDLNSAKVYSSPIKLQVFTFAEAYDFEDLESIVQEVARGFKTKIMFIYVDTAEENLAKPFLTLYGLEGDKPTVTAFDTSKGTKYLLEADINTKNLKEFCLSLLDGTLPPYFRSEPVPQEKGLVEKVVGRTLDSSVLQSPHNVLLEAYAPWCVDCEAISKNIEKLAKHFSGLDNLKFARIDASVNEHPKLQVNNYPTLLLYPAEDKTNPIKLSKKLSLKDMARFIKEKLQISDVEIKEKLQTPDVETVAAADNVKDEL

>HvPDIL5-1_BAJ84858

LYSAGSPVLQLNPNNFKKVLNANGVVLVEFFAPWCGHCKQLTPIWEKAAGVLKGVATVAALDADAHKELAQQYGIRGFPTIKVFLPGKPPVDYEGARDVKPIVNFALSQVKGLLRDRLDGKASGGSSSKTSGGSSEKKNEPNESVELNSSNFDELVIKSKDLWIVEFFAPWCGHCKKLAPEWKRAAKNLKGQVKLGHVDCDSDKSLMSKYKVEGFPTILVFGADKDSPFPYQGARAASAIESFALEQLEANAAPPEVSELTSADVMEEKCASAAICFVSFLPDILDSKAEGRNKYLELLLSVAEKFKKSPYSFVWAGAGKQADLEKQVGVGGYGYPAMVALNVKKGAYAPLRSAFELAEITEFVKEAGRGGKGNLPLEGAPTVVQSEPWDGKDGEVIEEDEFSLEELMADSSAPNDEL

>HvPDIL5-2_BAJ90795

IYPSNSDVIELTDDNFNQVLQSVEIWVVEFYAPWCGHCQRLVPEYTKAAKALKGIVKVAAIDADKYPSFAGRYGVQGFPTVKIFVDKNKPQDFTGDRTAVGITDEVIKAIKNAISANLQGVPYGSSKSSKKSSSGDDVVELTDSNFDKLVLNSDDIWLVEFFAPWCGHCKNLAPHWAAAASELKGKVKLGALDATVHSSKAQEFNIRGYPTIKFFPSGTSSSSGAEEYTGGRTSSDIVSWAMQKHQENVPPPDIIEIVNEDTFKAGCSEHALCVVSVLPHILDCQASCRNEYLNTLRSLGDKFKQKLWGWLWAEAGKQPELESTLEIGGFGYPALAVLNVKKMKYSILRGSFSEDGIKEFLRDLSYGRGTTAPVKGAALPEIQATEPWDGKDGELPTADDIDLSDVDLDELPKEEL

>SbPDIL1-1_Sb05g006150.1

EEPAAEGAAEAVLTLDVDSFDEAVAKHPFMVVEFYAPWCGHCKKLAPEYETAAKELSKHDPPIVLAKVDANEEKNRPLATKYEIQGFPTLKIFRNQGKNIQEYKGPREADGIVDYLKKQVGPASKELKSQEDVATHYDDKNIYIVGVFTEFSGTEFTNFMEVAEKLRSDYDFGHTLHANHLPRGDAAVERPLVRVLKPFDELVVDTKDFDVAALLKFIDATTVPRVVTFDKNPDNHPYLMKFFQSSAPKAMLFLNFSTGPFDSFKSVYYAAAEEFQNKEIKFLIGDLESSQGALQYFGLKEDQAPLILIQDGDSKKFLKDQIEADQIVSWLKEYFDGKLTPFKKSEPIPEVNNEPVKVVVADNIHDFVFKSGKNVLIEFYAPWCGHCKKLAPILEEAATTLQSDEEVVIAKMDATANDVPSEFEVQGYPTMYFVTPSGKVTAYDSGRTADDIVDFIKKSKETAGATQATTTTSEKAADAAEKAEPVKDEL

>SbPDIL1-2_Sb06g017160.1

EVETAVELGEVVLTLDASNFSEVVAKHQFIVVEFYAPWCGHCKQLAPEYEKAAAVLRNHDPPLVLAKVDAYDERNKEIKDKYQVHAYPTIKIIENGGKDVRGYGGPRDADGIVGYLKKQVGPASIELSSAEAAQSSIGDKGVVLVGVFPEFAGVEYENFMAVAEKKRSDYDFFHTSDASILPRGDQTIKGPVVRLFKPFDELFADSRDFDTDALEKFIDVSGFPAVVTFDADPTNHKFLERYYSTPSAKAMLFLNFSDDRVEAFKNQIQEAAKKFSANNISFLIGDVEAADRAFQYGNLTPYVKSEPIPKVNDQPVKVVVADSIDDVVFNSGKNVLLEFYAPWCGHCRKLAPILEEVAVSLQDDEDVVIAKMDGTANDIPTDLAVEGYPTIYFYSTTGDLYSYNGGRTAEDIISFIKKNKGPRAGAVDEVTQTDAGAVEEGTAPSSTSELPKDEL

>SbPDIL2-1_Sb04g000230.1

AGSNKAEEELDDLQYLIDNSHDIPANDPDGWPEGGGGGDDDDDDDLLFQDQDEDLLGHQPQIDETHVVVLTAANFSSFLSATRHVMVEFYAPWCGHCQELAPEYAAAAAHLAAHPHQADLALAKVDATEETDLAQRYDVQGFPTILFFIDGVPKDYNGARTKDAIVDWINKKLGPAVQNVTSVDEAERILTGDDKAVLAFLDTLSGAHSDELAAASRLEDSINFYQTLTPDVAKLFHIDAATKRPSIVLLKKEEEKLTFYDGEFKASAIADFVSANKLPLVTTLTQETSPSIFGNPIKKQILLFAIASESSKFLPIFKEAAKPFKGKLLFVFVERDNEEVGEPVADYFGITGQETTVLAYTGNEDAKKFFLDGEVSLEAIKDFAEGFLEDKLTPFYKSEPVPESNDGDVKMVVGKNLDLIVLDESKDVLLEIYAPWCGHCQSLEPTYNKLARHLRGVDSLVIAKMDGTANEHPRAKSDGYPTILFYPAGKKSFEPITFEGERTVVDMYKFIKKHASIPFKLKRQESSTQMEEGVKSSDTNLKDEL

>SbPDIL3-1_Sb10g004440.1

RLDLDDDDDSGVLDDLLAIDEEPDRAGLDAAAGGAAEAVRRAQSMVLVLDNDNARRAVEDHAELLLLGYAPWCERSAQLMPRFAEAAAALRAMGSAVAFAKLDGERYPKAAAAVGVRGFPTVLLFVNGTEHAYQGLHTKDAIVTWVRKKTGVPVIRLQSKESAEEFLKKDQTFVIGLFKNFEGAEHEEFVKAATTDNEVQFVETSDTSVAKVLFPGITSVEKFVGLVKSEPEKFEKFDGEFEEKAILRFVELNKFPLITVFTELNSGKVYSSPIKLQVFTFSEAYDFEDLESMVEEIARAFKTKIMFIYVDTAEENLAKPFLTLYGLESEKRPTVTAFDTSNGAKYLMEADINAKNLREFCLSLLDGTLPPYHKSEPVPQEKGLVEKVVGRTFDSSVLESHQNVFLEVHTPWCVDCEAISKNVEKLAKHFNGLDNLKFARIDASVNEHPKLKVNNYPGLFLFLAEDKSKPIKLSKKSSVKDMAKLIKEKLQISDVETVAAPDNVKDDVETVAAPDSVKDEL

>SbPDIL4-1_Sb09g004370.1

DDVVALTEADFEKEVGQDRGALVEFYAPWCGHCKKLAPEYEKLGASFKKAKSVLIAKVDCDEHKGLCSKYGVSGYPTIQWFPKGSLEPKKYEGQRSVEALAEYVNSEAGTNVKIVAIPSSVVVLTPETFDSIVLDETKDVLVEFYAPWCGHCKHLAPVYEKLASVFKQDDGVVIANLDADKHTDLAEKYGVSGFPTLKFFPKGNKAGEDYDGGRDLDDFVKFINEKCGTSRDSKGQLNSEAGLVASLNPLVKEFLNAAADKRKEVISKIEEDVAKLSGSAAKHGKIYVTAAKKIMDKGSDYTKKETERLHRLLEKSISPSKADEFIIKKNILSTFSS

>SbPDIL4-2_Sb03g013630.1

DGDDVVALTESTFEKEVGQDRGALVEFYAPWCGHCKKLAPEYERLGASFKKAKSVLIAKIDCDEHKSLCSKYGVSGYPTIQWFPKGSLEPKKYEGQRTAEALAEFVNTEGGTNVKLATIPSSVVVLTPETFDSIVLDEAKDVLVEFYAPWCGHCKSLAPTYEKVASVFKLDEGVVIANLDADKYRDLAEKYGVTGFPTLKFFPKGNKAGEDYDGGRDLGDFVKFINEKSGTSRDTKGQLTSEAGRIASLDVLAKEFLGASSDKRKEVLSSM

EEEAAKLSGPSARHGKVYVNIAKKILEKGNEYTKKETERLDRMLEKSINPSKADEFIIKKNVLSTFSS

>SbPDIL5-1_Sb02g026300.1

LYSAGSPVLQLNPNNFKSKVLNSNGVVLVEFFAPWCGHCKQLAPAWEKAAGVLKGVATVAALDADAHQALAQEYGIRGFPTIKVFSPGKPPVDYQGARDVKPIVEFALSQVKSLLRERLSGKASAGSNGKTSGGSSEKSEPSASVELNSRNFDELVVKSKDLWIVEFFAPWCGHCKKLAPEWKKAAKNLKGQVKLGHVDCDAEKSLMSKYKVEGFPTILVFGADKESPFLYQGARVSSAIESFALEQLEANSGPAEVSELTGPDVMEEKCASAAICFVSFLPDILDSKAEGRNKYLELLLSVAEKFKKSPYSFVWTAAGKQANLENQVGVGGYGYPAMVALNVKKGAYTPLRSAFQRDEIIEFVKEAGRGGKGNLPLNGAPTVVTSEPWDGKDGEVIEEDEFSLDELMGDSSSVNDEL

>AtPDIL1-1_AT1G21750.1

EETETKEFVLTLDHTNFTDTINKHDFIVVEFYAPWCGHCKQLAPEYEKAASALSSNVPPVVLAKIDASEETNREFATQYEVQGFPTIKIFRNGGKAVQEYNGPREAEGIVTYLKKQSGPASAEIKSADDASEVVSDKKVVVVGIFPKLSGSEFDSFMAIAEKLRSELDFAHTSDAKLLPRGESSVTGPVVRLFKPFDEQFVDSKDFDGEALEKFVKESSIPLITVFDKDPNNHPYVIKFFESTNTKAMLFINFTGEGAESLKSKYREVATSNKGQGLSFLLGDAENSQGAFQYFGLEESQVPLIIIQTADDKKYLKTNVEVDQIESWVKDFKDGKIAPHKKSQPIPAENNEPVKVVVSDSLDDIVLNSGKNVLLEFYAPWCGHCQKLAPILDEVAVSYQSDSSVVIAKLDATANDFPKDTFDVKGFPTIYFKSASGNVVVYEGDRTKEDFISFVDKNKDTVGEPKKEEETTEEVKDEL

>AtPDIL1-2_AT1G77510.1

EETKEFVLTLDHSNFTETISKHDFIVVEFYAPWCGHCQKLAPEYEKAASELSSHNPPLALAKIDASEEANKEFANEYKIQGFPTLKILRNGGKSVQDYNGPREAEGIVTYLKKQSGPASVEIKSADSATEVVGEKNVVAVGVFPKLSGDEFDSFMALAEKLRADYDFAHTLDAKFLPRGESVEGPAVRLFKPFDELFVDSKDFNGEALEKFVKESSIPLVTVFDSDPNNHPYVAKFFESPATKAMMFVNFTGATAEALKSKYREVATSNKDQSLAFLVGDAESSQGAFQYFGLEESQVPLIIIQTPDNKKYLKVNVEVDQIESWFKDFQDGKVAVHKKSQPIPAENNEPVKVVVAESLDDIVFKSGKNVLIEFYAPWCGHCQKLAPILDEVALSFQNDPSVIIAKLDATANDIPSDTFDVKGFPTIYFRSASGNVVVYEGDRTKEDFINFVEKNSEKKPTSHGEESTKSEEPKKTEETAAKDEL

>AtPDIL2-1_AT3G54960.1

ENASSGSDLDEELAFLAAEESKEQSHGGGSYHEEEHDHQHRDFENYDDLEQGGGEFHHGDHGYEEEPLPPVDEKDVAVLTKDNFTEFVGNNSFAMVEFYAPWCGACQALTPEYAAAATELKGLAALAKIDATEEGDLAQKYEIQGFPTVFLFVDGEMRKTYEGERTKDGIVTWLKKKASPSIHNITTKEEAERVLSAEPKLVFGFLNSLVGSESEELAAASRLEDDLSFYQTASPDIAKLFEIETQVKRPALVLLKKEEEKLARFDGNFTKTAIAEFVSANKVPLVINFTREGASLIFESSVKNQLILFAKANESEKHLPTLREVAKSFKGKFVFVYVQMDNEDYGEAVSGFFGVTGAAPKVLVYTGNEDMRKFILDGELTVNNIKTLAEDFLADKLKPFYKSDPLPENNDGDVKVIVGNNFDEIVLDESKDVLLEIYAPWCGHCQSFEPIYNKLGKYLKGIDSLVVAKMDGTSNEHPRAKADGFPTILFFPGGNKSFDPIAVDVDRTVVELYKFLKKHASIPFKLEKPATPEPVISTMKSDEKIEGDSSKDEL

>AtPDIL2-2_AT5G60640.1

ASSSDDVDDEDLSFLEDLKEDDVPGADSLSSSTGFDEFEGGEEEDPDMYNDDDDEEGDFSDLGNPDSDPLPTPEIDEKDVVVIKERNFTDVIENNQYVLVEFYAPWCGHCQSLAPEYAAAATELKEDGVVLAKIDATEENELAQEYRVQGFPTLLFFVDGEHKPYTGGRTKETIVTWVKKKIGPGVYNLTTLDDAEKVLTSGNKVVLGYLNSLVGVEHDQLNAASKAEDDVNFYQTVNPDVAKMFHLDPESKRPALVLVKKEEEKISHFDGEFVKSALVSFVSANKLALVSVFTRETAPEIFESAIKKQLLLFVTKNESEKVLTEFQEAAKSFKGKLIFVSVDLDNEDYGKPVAEYFGVSGNGPKLIGYTGNEDPKKYFFDGEIQSDKIKIFGEDFLNDKLKPFYKSDPIPEKNDEDVKIVVGDNFDEIVLDDSKDVLLEVYAPWCGHCQALEPMYNKLAKHLRSIDSLVITKMDGTTNEHPKAKAEGFPTILFFPAGNKTSEPITVDTDRTVVAFYKFLRKHATIPFKLEKPASTESPKTAESTPKVETTETKESPDSTTKSSQSDSKDEL

>AtPDIL3-1_AT1G52260.1

SPDSNVESNEPGFDSDLDQLLAVDEQLQEDRPEQQSEAETVSKAQRIVLELNGDYTKRVIDGNEFVMVLGYAPWCARSAELMPRFAEAATALKEIGSSVLMAKIDGDRYSKIASELEIKGFPTLLLFVNGTSLTYNGGSSAEDIVIWVQKKTGAPIITLNTVDEAPRFLDKYHTFVLGLFEKFEGSEHNEFVKAAKSDDEIQFIETRDSDVAKLLFPDLKSNNVFIGLVKPEAERYTVYDGSYKMEKILEFLGSNKFPLFTKLTETNTVWVYSSPVKLQVMLFSKADDFQKLAQPLEDIARKFKSKLMFIYVDITNENLAMPFLILFGIEAGNKTVVAAFDNNLNSKYLLESDPSPNSIEEFCSGLAHGTVSRYYRSEPVPDNENASIVTVVGKTFDGLVLNSRENVLLEVHTPWCVNCEALSKQIEKLAKHFKGFENLVFARIDASANEHTKLQVDDKYPIILLYKSGEKEKPLKLSTKLSAKDIAVFINEELLKPKNGSAKDEL

>AtPDIL3-2_AT3G16110.1

SDVAVEAGSEEELDDLEQLLAVDEQLQEERPEQQSEAETVSKAQRIVVELNGDNTKRLIDGNEYVMVLGYAPWCARSAELMPRFAEAATDLKEIGSSVLMAKIDGERYSKVASQLEIKGFPTLLLFVNGTSQSYTGGFSSEEIVIWVQKKTGASTIKLDTVDEASGFLKKHHTFILGLFEKSEDSSGHDEFVKAASLDNEIQFVETSSIDVAKLLFPNLKTNNVFVGLVKTEAEKYTSYDGPCQAEKIVEFLNSNKFPLVTKLTESNTVRVYSSPVKLQVMVFSKTDDFESLAQPLEDIARKFKSKLMLIYIDISNENLAMPFLTLFGIEDAKKTVVAAFDNNLNSKYLLESDPSPSNIEEFCFGLAHGTVSAYYKSQPIPDNQNASVVAVVGRTFDEVVLRSSENVLLEVHTPWCINCEALSKQVEKLSQHFKGFENLVFARIDASANEHPKLTVDDYPTILLYKTGEKENPLKLSTKSSAKDMAVLINKELKWKDQSGKDEL

>AtPDIL4-1_AT2G47470.1

DDVVVLTDDSFEKEVGKDKGALVEFYAPWCGHCKKLAPEYEKLGASFKKAKSVLIAKVDCDEQKSVCTKYGVSGYPTIQWFPKGSLEPQKYEGPRNAEALAEYVNKEGGTNVKLAAVPQNVVVLTPDNFDEIVLDQNKDVLVEFYAPWCGHCKSLAPTYEKVATVFKQEEGVVIANLDADAHKALGEKYGVSGFPTLKFFPKDNKAGHDYDGGRDLDDFVSFINEKSGTSRDSKGQLTSKAGIVESLDALVKELVAASEDEKKAVLSRIEEEASTLKGSTTRYGKLYLKLAKSYIEKGSDYASKETERLGRVLGKSISPVKADELTLKRNILTTFVASS

>AtPDIL5-1_AT1G04980.1

LYGSSSPVLQLTPSNFKSKVLNSNGVVLVEFFAPWCGHCQSLTPTWEKVASTLKGIATVAAIDADAHKSVSQDYGVRGFPTIKVFVPGKPPIDYQGARDAKSISQFAIKQIKALLKDRLDGKTSGTKNGGGSSEKKKSEPSASVELNSSNFDELVTESKELWIVEFFAPWCGHCKKLAPEWKKAANNLKGKVKLGHVNCDAEQSIKSRFKVQGFPTILVFGSDKSSPVPYEGARSASAIESFALEQLESNAGPAEVTELTGPDVMEDKCGSAAICFVSFLPDILDSKAEGRNKYLEMLLSVADKFKKDPYGFVWVAAGKQPDLEKRVGVGGYGYPAMVALNAKKGAYAPLKSGFEVKHLKDFVKEAAKGGKGNLPIDGTMEIVKTEAWDGKDGEVVDADEFSLEDLMGNDDEASTESKDDL

>AtPDIL5-2_AT2G32920.1

LYGSSSPVVQLTASNFKSKVLNSNGVVLVEFFAPWCGHCKALTPTWEKVANILKGVATVAAIDADAHQSAAQDYGIKGFPTIKVFVPGKAPIDYQGARDAKSIANFAYKQIKGLLSDRLEGKSKPTGGGSKEKKSEPSASVELNASNFDDLVIESNELWIVEFFAPWCGHCKKLAPEWKRAAKNLQGKVKLGHVNCDVEQSIMSRFKVQGFPTILVFGPDKSSPYPYEGARSASAIESFASELVESSAGPVEVTELTGPDVMEKKCGSAAICFISFLPDILDSKAEGRNKYLEMLLSVAEKFKKQPYSFMWVAAVTQMDLEKRVNVGGYGYPAMVAMNVKKGVYAPLKSAFELQHLLEFVKDAGTGGKGNVPMNGTPEIVKTKEWDGKDGELIEEDEFSLDELMGGDDAVGSKDEL

>GmPDIL1-1_Glyma04g42690.1

EESSEKEFVLTLDHSNFHDTVSKHDFIVVEFYAPWCGHCKKLAPEYEKAASILSSHDPPVVLAKIDANEEKNKDLASQYDVRGYPTIKILRNGGKNVQEYKGPREADGIVDYLKKQSGPASTEIKSADEATAFIGENKVAIVGVFPKFSGEEFDNFSALAEKLRSDYDFGHTLNAKHLPRGESSVSGPVVRLFKPFDELFVDFQDFNVEALEKFVEESSTPVVTVFNNDPSNHPFVAKFFNSPNAKAMLFINFTAEGAESFKSKYREAAEQHKQQGVSFLVGDVESSQGAFQYFGLKEEQVPLIIIQHNDGKKFFKPNLEADHIPTWLKAYKDGNVAPFVKSEPIPEANDEPVKVVVGNSLEDIVFKSGKNVLLEFYAPWCGHCKQLAPILDEVAISYQSDADVVIAKLDATANDIPSETFDVQGYPTVYFRSASGKLSQYEGGRTKEDIIEFIEKNRDKPAQQEQGQDKPAQQEQGQDEQEKGKDEL

>GmPDIL1-2_Glyma06g12090.1

EESSEKEFVLTLDHSNFHDTVSKHDFIVVEFYAPWCGHCKKLAPEYEKAASILSSHDPPIVLAKVDANEEKNKDLASQYDVKGFPTINILRNGGKNVQEYKGPREADGIVDYLKKQSGPASTEIKSADEATAFIGENKVAIVGVFPKFSGEEFDNFSALAEKLRSDYDFGHTLNAKLLPRGESSVSGPVVRLFKPFDELFVDFQDFNVEALEKFVEESSTPVVTVFNNEPSNHPFVVKFFNSPNAKAMLFINFTAEGAEAIKSKYREAAEQYKQQGVSFLVGDVESSQGAFQYFGLKEEQVPLIIIQHNDGKKFFKPNLEADHIPTWLKAYKDGHVAPFVKSEPIPETNDEPVKVVVGASLEDIVFKSGKNVLLEFYAPWCGHCKQLAPILDEVAISYQNEADVVIAKLDATANDIPSETFDVQGYPTVYFRSASGKLSQYDGGRTKEDIIEFIEKNRDKPAQQEQGKDEQEQGKDEL

>GmPDIL2-1_Glyma12g29550.1

DDLTDDEDLGFLDEPSAAPEHGHYHDDDANFGDFEEDPEAYKQPEVDEKDVVILKEKNFTDTVKSNRFVMVEFYAPWCGHCQALAPEYAAAATELKGEDVILAKVDATEENELAQQYDVQGFPTVYFFVDGIHKPYNGQRTKDAIMTWIKKKIGPGIYNLTTVEDAQRILTNETKVVLGFLNSLVGPESEELAAASRLEDDVNFYQTVDPDVAKLFHIDPDVKRPALILVKKEEEKLNHFDGKFEKSEIADFVFSNKLPLVTIFTRESAPSVFENPIKKQLLLFATSNDSEKLIPAFKEAAKSFKGKLIFVYVEMDNEDVGKPVSEYFGISGNAPKVLGYTGNDDGKKFVLDGEVTADKIKAFGDDFLEDKLKPFYKSDPVPESNDGDVKIVVGNNFDEIVLDESKDVLLEIYAPWCGHCQALEPIYDKLAKHLRNIESLVIAKMDGTTNEHPRAKPDGFPTLLFFPAGNKSFDPITVDTDRTVVAFYKFLKKHASIPFKLQKPTSTSDAKGSSDAKESQSSDVKDEL

>GmPDIL2-2_Glyma13g40130.1

DHLADDEDLSFLDEPSAAPEHDHHYGADDSNFGDFEDFEEDDAEAYKQPEVDEKDVVVLKEKNFTDAVKNNRFVMVEFYAPWCGHCQALAPEYAAAATELKGEDVILAKVDATEENELAQQYDVQGFPTVHFFVDGIHKPYNGQRTKDAIVTWIRKKIGPGIYNLTTVEEAQRILTNETKVVLGFLNSLVGPESEELAAASRLEDDVNFYQTVNPDVAKLFHIDQDVKRPALILIKKEEEKLNHFDGKFEKSAIADFVFSNKLPLVTIFTRESAPSVFENPIKKQLLLFATSNDSETLVPAFKEAAKSFKGKLIFVYVEMDNEDVGKPVSEYFGISGNAPKVLGYTGNDDGKKFVLDGEVTTDKIKAFGEDFVEDKLKPFYKSDPVPESNDGDVKIVVGNNFDEIVLDESKDVLLEIYAPWCGHCQSLEPIYNKLAKHLRNIDSLVIAKMDGTTNEHPRAKPDGFPTLLFFPAGNKSFDPITVDTDRTVVAFYKFLKKHASIPFKLQKPTSTSESDSKGSSDAKESQSSDVKDEL

>GmPDIL2-3_Glyma12g07260.1

DKIPPQNDNNNDDDEDLSFLEEPDDAAATSHHGHFPDPDRFDEDGDDDGDFGDFSGFDHSTEEAFEVDDKDVVVLKERNFTTVVENNRFIMVEFYAPWCGHCQALAPEYAAAATELKPDGVVLAKVDATVENELANEYDVQGFPTVFFFVDGVHKPYTGQRTKDAIVTWIKKKIGPGVSNITTVDDAERILTAESKVVLGLLNSLVGTESDELAAASKLEDDVNFYQTVVADVAKLFHIDPSVKRPALILLKKEEEKLNHFDGQFVKAEIADF

VTSNKLPLVTIFTRESAPVIFESQIKKQLLLFVTSNDTEKFVPVFKEAAKKFKGKLIFVHVELDNEDVGKPVADYFGITGNGPKVLAYTGNDDGRKFLLDEELTVDTITAFGNDFLEEKLKPFLKSDPVPESNDGDVKIVVGNNFDEIVLDESKDVLLEIYAPWCGHCQALEPTYNKLAKHLRNIESIVIAKMDGTTNEHPRAKSDGFPTLLFFPAGNKSSDPIPVDVDHTVKAFYKFLRKHASIPFQLQKPTSTAKTGSESSYVKESQSSSTDVKDEL

>GmPDIL2-4_Glyma11g20630.1

DKTPPQNDNKKNNNDDDEDLSFLEESDDAATTSHQGHFPDPDEFDEDDGDDEDDFGDFAGFDHSSEEAFKEPEVDDKDVVVLKERNFTTVVENNRFVMVEFYAPWCGHCQALAPEYAAAATELKPDGVVLAKVDATVENELANEYDVQGFPTVFFFVDGVHKPYTGQRTKDAIVTWIKKKIGPGVSNITTVEEAERVLTAGSKVVLGFLNSLVGAESDELAAASKLEDDVNFYQTVVADVAKLFHIDASVKRPALILLKKEEEKLNHFDGQFVKAEIADFVTSNKLPLVTTFTRESAPVIFESQIKKQLLLFVTSNDTEKFVPVFKEAAKIFKGKLIFVHVESDNEDVGKPVADYFGIAGNGPKVLAFTGNDDGRKFLLDGEVTIDTITAFGNDFLEDKLKPFLKSDPVPESNDGDVKIVVGNNFDEIVLDESKDVLLEIYAPWCGHCQALEPTYNKLAKHLRSIESIVIAKMDGTTNEHPRAKSDGFPTLLFFPAGNKSSDPIPVDVDRTVKDFYKFLRKHASIPFQLQKLASTTKTASESSDVKESQSSTTEVKDEL

>GmPDIL3-1_Glyma15g01880.1

EVEDELEELLAVDEEVEQEAEKGGEKLSEAEVLSKAQRIVIELNNDNTERVVNGNEFVLVLGYAPWCPRSAELMPHFAEAATSLKELGSPLVLAKLDADRYSKPASFLGVKGFPTLLLFVNGTSQPYSGGFAADDIVIWAQKKTSTPVIRIGSVTEAEKFLRKYQTFLIGRFDKFEGPDYEEFVSAAQSDNEIQFVETNQVELAQVLYPDIKPTDQFLGIVKSEPERYTAYDGAFTMNKILEFVDYNKFPLVTKLTEMNSIRVYSSPIKLQVLVFANIDDFKNLLETLQDVAKTFKSKIMFIYVDINDENLAKPFLTLFGLEESKNTVVAAFDNAMSSKYLLETKPTQSNIEEFCNNLVQGSLSPYFKSQPIPDNTESSVHVIVGKTFDDEILSSEKDVLLEVFTPWCINCEATSKQVEKLAKHYKGSSNLIFARIDASANEHPKLQVNDYPTLLLYRADDKANPIKLSTKSSLKELAASINKYVKVKNQVVKDEL

>GmPDIL3-2_Glyma13g43430.1

EVKDELEELLAVDEEVEREAEKGGEKLSEAEVLSKAQRIVIELKNENTERVVNGNEFVLVLGYAPWCPRSAELMPHFAEAATSLKELGSPLIMAKLDADRYPKPASFLGVKGFPTLLLFVNGTSQPYSGGFTADDIVIWAQKKTSTPVIRISSVAEAEKFLTKYQTFLIGRFENFEGPDYEEFVSAAKSDNEIQFVETSQVELAQVLYPDIKPTDRFLGIVKSEPERYSAYDGAFILNKILEFVDYNKFPLVTKLTEMNSVRVYSSPIKLQVLVFANIDDFKNLLDTLQDVAKTFKSKIMFIYVDINDENLAKPFLTLFGLEESKNTVVSAFDNSMSSKYLLESKPTQINIEEFCNNLMQGSLSPYFKSQPIPDNTEASVRAIVGKTFDDEILSSKKDVLLEVFTPWCMNCEATSKQVEKLAKHYKGSSNLIFARTDASANEHPKLQVNDYPTLLFYRADDKANPIKLSTKSSLKELAASINKYLKVKNQVLKDEL

>GmPDIL4-1_Glyma02g01750.1

DDVVVLSEDNFEKEVGQDRGALVEFYAPWCGHCKKLAPEYEKLGSSFKKAKSVLIGKVDCDEHKSLCSKYGVSGYPTIQWFPKGSLEPKKYEGPRTADSLAEFVNTEGDLLAGTNVKIATAPSNVVVLTSENFNEVVLDETKDVLVEFYAPWCGHCKSLAPTYEKVATAFKLEEDVVIANLDADKYKDLAEKYDVSGFPTLKFFPKGNKAGEEYGGGRDLDDFVAFINEKSGTSRDVKGQLTSQAGIVESLDVLVKEFVAASDEEKKFVFTRMEEEVEKLKGSASRHGKIYLKAAKNYLEKGSDYAKNEIQRLQRILDKSISPAKADELTLKKNILSTYAA

>GmPDIL4-2_Glyma19g41690.1

DDVVALTEETFENEVGKDRAALVEFYAPWCGHCKRLAPEYEQLGASFKKTKSVLIAKVDCDEHKSVCGKYGVSGYPTIQWFPKGSLEPKKYEGARTAEALAAFVNIEAGTNVKIASVASSVVVLSPNNFDEVVFDETKDVLVEFYAPWCGHCKALAPIYEKVAAAFNLDKDVVIANVDADKYKDLAEKYGVSGYPTLKFFPKSNKAGENYDGGRDLDDFVAFINEKCGTYRDGKGQLTSKAGIIASLDDLVKEFVSADSNEKKAVYSRLEEEVKKLKGSSARHGDLYLKLAKKGMEKGADYAKNEIQRLERMLEKSVSPAKADEFTLKKNILSIFA

>GmPDIL4-3_Glyma10g01820.1

DDVVVLSEDNFEKEVGQDRGALVEFYAPWCGHCKKLAPEYEKLGSSFKKAKSVLIGKVDCDEHKSLCSKYGVSGYPTIQWFPKGSLEAKKYEGPRTAESLVEFVNTEGGTNVKIATVPSNVVVLTPENFNEVVLDEAKDVLVEFYAPWCGHCKSLAPTYEKVATAFKLEEDVVIANLDADKYRDLAEKYDVSGFPTLKFFPKGNKAGEDYGGGRDLDDFVAFINEKSGASRDGKGQLTSQAGIVESLDVLVKEFVAASDEEKKSVFTRLEEEVVKLKGSASRYGKIYLKAAKNYREKGSDYAKNEIQRLQRILDKSISPAKADELTLKKNILSTYAA

>GmPDIL4-4_Glyma03g39130.1

DDVVALTEETFENEVGKDRAALVEFYAPWCGHCKRLAPEYEQLGTTFKKTKSVLIAKVDCDEQKSVCSKYGVSGYPTIQWFPKGSLEPKKYEGARTAEALAAFVNIEAGTNVKIASVPSSVVVLSPDNFDEVVLDETKDVLVEFYAPWCGHCKALAPIYEKVAAAFNLDKDVVMANVDADKYKDLAEKYGVSGYPTLKFFPKSNKAGEDYNGGRDLDDFVAFINEKCGTYRDGKGQLTSKAGIIASLDDLVKEFVSADSNEKKAVYSRLEEEVKKLKGSSARHGDLYLKLAKKGIEKGADYAKNEIQRLERMLEKSISPAKADEFTLKKNILSTFA

>GmPDIL5-1_Glyma14g05520.1

LYGASSPVLQLTPSNFKSKVLNSNGVVLVEFFAPWCGHCQALTPIWEKAATVLKGVVTVAAIDADAHPSLAQEYGIRGFPTIKVFAPGKPPVDYQGARDVKPIAEFALQQVKALLKDRLSGKATGGSSDKTETSSSVELNSGNFDELVIKSKELWIVEFFAPWCGHCKKLAPEWKKASNSLKGKVKLGHVDCDAEKSLMSRFKVQGFPTILVFGADKDSPIPYEGARTALAIESFALEQLETNVAPPEVTELHSPDVLEEKCGSAAICFVAFLPDILDSKAEGRNIYLQQLLSVAEKFKRSPYSYVWVAAGNQPDLEKNVGVGGYGYPALVALNLKKAVYAPLKSAFELDQIIEFVKEAGRGGKGNLPLQGTPTIVKTEPWDGKDGEIIEEDEFSLEELMGEDASSKDEL

>GmPDIL5-2_Glyma02g43460.1

LYGASTPVLQLTPSNFKSKVLNSNGVVLVEFFAPWCGHCQALTPIWEKAATVLKGVVTVAAIDADAHPSLAQEYGIRGFPTIKVFAPGKPPVDYQGARDVKPIAEFALQQVKALLKDRLSGKATGGSSEKTETSSSVELNSGNFDELVIKSKELWIVEFFAPWCGHCKKLAPEWKKASNNLKGKVKLGHVDCDAEKSLMSRFKVQGFPTILVFGADKDSPIPYEGARTASAIESFALEQLETNIAPPEVTELYSPDVLEEKCGSAAICFVAFLPDILDSKAEGRNRYLQQLLSVAEKFKRSPYSYVWVAAGKQPDLEKNVGVGGYGYPALVALNLKKAVYAPLKSAFELDQIIEFVKEAGRGGKGNLPIEGTPTIVKTEPWDGKDGEIIEEDEFSLEELMGEDASSKDEL

>PtPDIL1-1_Potri.002G082100.1

EDESKEYVLTLDHSNFNETVSKHDFIVVEFYAPWCGHCKKLAPEYEKAASILSSNDPQVVLAKVDANEDANKEIASQ

YDVKGFPTIVILRKGGKSVQEYKGPREADGIVEYLKKQSGPASAELKSDDDATGFIGDKKVVIVGVFPKFSGEEFENFLAVAEKLRSDYEFGHTLDAKYL

PRGESSVSGPLVRLFKPFDELFVDSKDFNVDALEKFVEESSIPIVTLFNKDPSNHPFVVKYFDSPLAKAMLFMNFSSENGDSIRTKYQEVAGLHKGDGLV

FLLGDVEASQGALQYFGLKEDQVPLIVIQTTDGQKYLKPNLVSDQIAPWLKEYKEGKVPPFKKSEPIPEVNDEPVKVVVADSLDELVTKSGKNVFLEFYA

PWCGHCQKLAPILEEVAISFQSDADVVIAKLDATANDIPSDTYDVKGFPTIFFRSATGKLVQYEGDRTKQDIIDFIEKNRDKIGQQEPAKEEEPAKEQET

AKDEL

>PtPDIL1-2_Potri.005G179000.1

EDESKEYVLTLDHSNFTETVTKHDFVVVEFYAPWCGHCQNLAPEYEKAASILSSNDPQIVLAKVNADEKVNQEISE

KYEVQGFPTIKILRKGGTSVNEYKGPRDADGIAEYLKKQTGPASAELKSADDATSFIGDNKVVIVGVFPKFSGEEFESFLAVADKLRSDYEFAHTLDAKH

LPRGESSVSGPLVRLFKPFDELFVDSKDFNVDALEKFIEESSAPIVTVYDDEPSNHPYIVKYFDSPLDKAMLFLNFSGDSADSIKTNYQEVAEQHKGDGL

IFLLGDLEASQSALQYFGLKEDQAPLLVIQTTDGKKYLKSNLESDHIAPWVKEYKEGKVPPFIKSEPIPEANEEPVKVVVADSLDDLVTKSGKNVLLEFY

APWCGHCQKLAPILEEIAVSYQSDADVLLAKLDATANDIPGDTYDVKGFPTVYFRSASGKLVQYEGDKTKQDIIDFIEKNRDKVAQQEPAKDEL

>PtPDIL2-1_Potri.009G013600.1

KLQNAAAEDDDEDLSFLEEETDAVPHGQGHGHDHDHDHYPDPDQFDEEFDNEDDLDNYSDLDDSELDSYKEPEID

DKDVVVLKEGNFSDFVTKNKFVMVEFYAPWCGHCQSLAPEYAAAATELKAEEVMLAKVDATEENELAQEYDIQGFPTVYFFVDGVHRPYPGPRNKDGIVT

WIKKKIGPGIYNITTVDDAERLLTSETKLVLGFLNSLVGPESEELAAASRLEDEVSFYQTVNPDVAKLFHLDPQAKRPALVMLKKEAEKLSVFDGNFSKS

EIAEFVFANKLPLVTIFTRESAPLIFESTIKKQLLLFAISNDSEKVVPIFQEAARLFKGKLIFVYVEMDNEDVGKPVSEYFGISGTAPKVLAYTGNDDAK

KFVFDGDVTLDKIKAFGEDFIEDKLKPFFKSDPVPESNDGDVKIVVGNNFDEIVLDESKDVLLEIYAPWCGHCQSLEPTYNKLATHLRGIESIVIAKMDG

TTNEHPRAKSDGFPTLLFFPAGNKSFDPITVDTDRTVVAFYKFIKKHASIPFKLQKPASASKAESSDAKDGIESSTRDVKDEL

>PtPDIL3-1_Potri.001G183500.1

NEDPTVETDNDGADSDLQELIAIDEQEGGGGEEQQQGDQQKEAEVLSKAQRIVLELNSDNARRVIDQNEFVLILGYAPWCARSAELMPQFAEAANKLKELGSPVLMAKLDAERYPKVASTLGIKGFPTLLLFVNGTSQV

YTGGFSGEDIVIWARKKTGVPVIRISSSVEAEDFQKKYHLFVLGLFDKFEGHDYEEFIKAATIDNEIQFVEVSSSAVAKILFPNINAKDNFIGIVKSEPE

KYTAYGGIFEKDTILQFLEYNKFPLVTILTELNSARVYSSPVKLQVIVFADADDFKNLIRPLQEVARKFISKIMFIYIDIADENQAKPFLTLFGIEDSEN

TVVTAFDNRMSSKYLLESNPTSSNIEEFCSRLLHGSLSPYFKSQPIPDNKEKILQVVVGKTLDDLVLSSPKNVLLEVYTPWCISCETTTKQIEKLAKHFK

GVDNLVFARIDASANEHPKLLVDDYPTLLFYPVGDKENPVKLSTKSSSKDLATVIKSLLRAKEDVPKDEL

>PtPDIL4-1_Potri.002G198300.1

DDVVVLTEDNFEKEVGQDKGALVEFYAPWCGHCKKLAPEYEKLGSSFKKAKAVLIGKVDCDEHKGVCSKYGVSGYPT

LQWFPKGSLEPKKYEGPRTAEALAEFVNNEGGSNVKIAAVTSSVVVLTADNFNDIVLDENKDVLVEFYAPWCGHCKNLAPIYEKVATAFKSEEDVVVANL

EADKYRDLAEKYGVSGFPTLKFFPKGNKAGEEYEGGRDLDDFVAFINEKAGTSRDGKGQLTSKAGIVESLDALVKEFVAAGDDEKKAVFSRIEEEVEKLK

GSTARHGKIYLKAAKTCMVKGAGYAKNEIERLQRMLEKSISPAKADEFTLKKNILSTFA

>PtPDIL4-2_Potri.014G122800.1

DDVVVLTEDNFEKEVGQDRGALVEFYAPWCGHCKKLAPEYEKLGSSFRKAKTVLIGKVDCDEHKGVCSKYGVSGYPT

LQWFPKGSLEPKKYEGPRTAEALTEYVNTEGGTNVKIAAVPSNVAVLTADNFNNIVLDETKDVLVEFYAPWCGHCKNLAPTYEKVATAFKSEEDVVVANL

DADKHKDLAEKYGVSGFPTLKFFPKGNKAGEDYEGGRDLDDFVAFINEKSGSSRDGKGQLTSKAGIVESLDALVKEFVAAGDDEKKAVFSQIEEEVEKLK

GSAARYGKIYSKAAKNCMAKGDYAKNEIERLQRMLQKTISPAKADEFTLKKNILSTFA

>PtPDIL5-1_Potri.014G160000.1

LYGPSSPVLQLNPSNFKSKVLNSNGVVLVEFFAPWCGHCKALTPTWEKAAAVLKGVATVAALDADAHQSLAQEYGI

RGFPTIKVFVPGNPPVDYQGARDVKPIAEYALKQIKALLKDRLNGKSTGGSSEKSETSLSVELNSRNFDELVLKSKELWIVEFFAPWCGHCKKLAPEWTK

AANNLQGKVKLGHVDCDSEKSLMSRFNVQGFPTILVFGADKDTPIPYEGARTASAIESFALEQLESNVAPPEVTELTGPDVMEEKCGSAAICFVAFLPDI

LDSKAEGRNKYLEQLLSVAEKFKRSPYSYVWAAAGKQPDLENRVGVGGYGYPALVALNAKKGAYAPLKSAFELEHIVEFVKEAGRGGKGNLPLNGNPEIV

KTEPWDGKDGEIIEEDEFSLEELMGEDAGSKDEL

>BrPDIL1-1_Bra016405

EETATETTKEFVLTLDHTNFTDTVNKHDFIVVEFYAPWCGHCKQLAPEYEKAASELSSHVPPVVLAKIDASEETNRE

FATQYEVQGFPTIKIFRNGGKAVQEYNGPREADGIVTYLKKQSGPASFEIKAAEDASEFDKKVIVVGVFPKLSGSEFDSFLATAEKLRSDYDFAHTSDAK

LLPRGESVTGPVVRLFKPFDELFVDSKDFDGEALEKFVKESSIPLITVFDKDPNNHPYVIKFFDSSNTKAMLFINFTGEGAESLKSKYREVATSYKGQGL

SFLLGDAENSQGAFQYFGLEESQVPLIIIQTVDDKKYLKTNIEIDQIESWVKDFKDGKVAPHKKSQPIPTENNEPVKVVVAESLDEMVFNSGKNVLLEFY

APWCGHCQKLVPILDEVAVSYQSDPSVVIAKLDATANDFPNDTFDVKGFPTIYLRSASGNIVLYDGDRTKEDIISFIDKNKDTAGEPKKEETTTEAVKDEL

>BrPDIL1-2_Bra012293

ETKEFVLTLDHSNFTDTINKHDFIVVEFYAPWCGHCKQLAPEYEKAASELSSNVPAVVLAKIDASEETNKEFATK

YEVQGFPTIKIFRNGGKAVQEYKGPREADGIVSYLKKQSGPASFEIKSGDDVVGDKKVVVVGVFPKLAGSEFDSFLATAEKLRSDYDFAHTSDAKLLPRG

ESVTGPVVRLFKPFDELFVDSKDFDGEALEKFVKESSIPLITVFDKDPNNHPYVIKFFDSPNTKAMFFINFTGESAETLKSKYREVATSNKGQGLSFLLG

DAENSQGAFQYFGLEESQVPLIIIQTADDKKYLKTNVEVDQIGSWIKDFKDGKVSPHKKSQPIPTENNEPVKVVVGESLDDMVFNSGKNVLLEFYAPWCG

HCQKLVPILVEVAVSYQSDPSVVIAKLDATANDFPRDTFDVKGFPTIYFRSASGNVVLYEGDRTKEDFISFIDKNKDTAGEPKTEDKTAEATKDEL

>BrPDIL1-3_Bra017948

KETKEFVLTLDHTNFTETINKHDFIVVEFYAPWCGHCKQLAPEYEKAASELSSHVPPVVLAKIDASEETNKEFATKY

SVQGFPTIKILRNGGKAVQEYNGPREADGIVTYLKKQSGPASLEIKSADAASEVVGDKNVVAVGVFPKLSGAEFDSFMATAEKLRSDYDFAHTTDAKLLP

RGESVTGPVVRLFKPFDELFVDFRDFVGEALEKFVKESSIPLITVFDSDPNNHPYVLKFFEIPNTKALFFLNFNGEGAETLKSKYREVAASNKGHGLSFL

LGDAKNSEEALQHYGVEQRQLPLIILQTVDDKKYLKTNVEVDQIESWINDFKDGKASPYKKSQPIPGENNEPVKVVVAENLDEMVFSSGKNVLLEFYAPW

CGHCQNLVPILDEVAVSYQSDPSVVIAKFDATANDFPHDTFDVKGFPTIYLRSANGNIVLYKGDRTKEDIISFIDKNKDTAGETKTEEKKTKEVKDEL

>BrPDIL1-4_Bra015665

EETETKEFVLTLDHSNFTDTINKHDFIVVEFYAPWCGHCKSLAPEYEKAAAELSSQSPPIFLAKIDASEESNKGIAN

DYKIQGFPTIKILRKGGKSIQDYNGPREAAGIVTYVKKQSGPASAEIKSADGAGEVIGEKSVVAVGVFPKLSGEEFDSFMALAEKLRADYDFAHTLDAKL

LPRGDSSVAGPVVRLFKPFDELFVDSKDFNGEALEKFVKESSIPLVTVFDKDPSNHPYVSKFFDNPATKVMMFVNFTGETAESLKSKFREVATSSKGQDL

AFLVGDAESSQGALQYFGLEESQVPLIIIQTPDSKKYLKANVVVDQIESWMKDFKDGKVAAHKKSQPIPAENNEPVKVVVAESLDEMVFNSGKNVLIEFY

APWCGHCQKLAPILDEVALAFQNDPSVIVAKLDATANDIPSDTFDVKGFPTIYFRSADGKVVVYEGSRTKEDFISFIEKNKPASHSEESSTTVRSGEHKT

EESAAKDEL

>BrPDIL1-5_Bra008311

EETETKEFVLTLDHSNFTETINKHDFIVVEFYAPWCGHCKSLAPEYEKAASELITHNPPLVLAKIDASEESNKGIANE

YKIQGFPTIKILRNGGKSIQDYNGPREAPGIVSYVKKQSGPASSEIKTAADAAEVVGEKNVVAVGVFPKLSGEEFDSFIALAEKLRGDYDFAHTLDAKLL

PRGDSSVAGPVVRLFKPFDELFVDSKDFNGEALEKFLKESSIPLVTVFDSDPSNRPYVASFFDSSATKVMMFVNFTGESAESLKSKFRKVATSYKGQDLS

FLVGDAEGGKGALEYFGVEESQVPLVIIQTPDSKKYLKANVVVEEIESWMKDFKDGKVDVFKKSQPIPAENNEPVKVVVAETLDDIVLKSGKNVLIEFYA

PWCGHCQKIAPILDEVALAFKNDPSVIIAKLDATANDIPSEPFDVKGFPTIYFRSVSGTVVAYEGNRTKEDFISFIEKNKPTTSHVEDTTSSTKTEEPKK

IDDASDTKDEL

>BrPDIL2-1_Bra020239

ADVDDEEDLSFLEDLTEEVKAPAKPLTDDFEGGEDDDDEEEDGEHFSDVSNQDSDPFPLSDVDEKDVVVVKERNF

TDVIENNEYVMVEFYAPWRGHCQSLAPEYAAAATELKGDGVVLAKIDATVENELAHQYSVQGFPTILFFVDGEHKLYTGGRTKETIVTWVKKKIGPSVYN

LTTLDDAEKVLTSGNKVVLGYLNSLVGVEHDQLAAASKAEDDVNFYQTVNPDVAKMFHIDPESKRPALVLVKREEEKISHFDGEFVKSGLVSFVSANKLP

LVTVFTPESSQEIFESAIKKQLLLFATENGSEKVLQEFEEAATLFKGKLIFVSVDVDNEDYGKPVAEYFGVSSSNAPKLVAFTGNEDPQKHYFEGEIKSD

KIKIFGEEFLSDKLKPFYKSDPIPEKNDGDVKIVVGDNFDEIVLDESKDVLLEVYAPWCGHCQALEPMYNKLAKHLRSIDSVVIAKMDGTTNEHPKAKAE

GFPTVLFFPAGNKTSSEPITVDADRTVVAFYKFLRKHATIPFKLEKPAASTESPTAAESTPKVETTETKGKLESTTTKSTESDSKDEL

>BrPDIL2-2_Bra002464

TSDVDDEDLSFLEDPKEEHDPTKPLTSTESELDEFNEGEEEDPEMYEGDDEEEGEDLSDLGNPDSDPFPTPDVDE

KDVVVVKERNFTDVIENNQYVMVEFYAPWCGHCQSLAPEYAAAATELKGDGVVLAKIDATEENELAHQYSVQGFPTILFFVDGEHKPYTGGRTKDTIVTW

VKKKIGPSVYNLTTLDDAEKVLTSGNKVVLGYLNSLVGVEHDQLAAASKAEDDVNFYQTVNPDVAKLFHIDPEAKRPAVVLVKREAEKISHFDGEFVKSD

LASFVSANKLPLVSVFTRESAPEIFESAIKKQILLFVTQNGSEKVLPEFEEAAKSFKGKLIFVSVDLDNEDYGKPVAEYFGVSGNGPKLIAYTGNEDPKK

HFFDGEIKSDKIKTFAEEFLSDKLKPFYKSDPIPEKNDGDVKIVVGDNFDDIVLDESKDVLLEVYAPWCGHCQALEPMYNKLAKHLREIDSLVIAKMDGT

TNEHPKAKAEGFPTILFFPAGNKTAEPITVDTDRTVVAFYKFLRKHATIPFKLEKPAASTESPKTAKSTPKVETTETKGNPQSTTKSTESDLKDEL

>BrPDIL2-3_Bra007120

ENAANGSDLDEELAFLAAEESKEEQQHHANSHHDQYRDFENYEDLEQGGEFHHGEHEGGGEYHEEEPQLPIVDEKDV

AVLTKDNFTEFVGNNSFAMVEFYAPWCGACQALAPEYAAAATELKGVAALAKIDATEEGDLAQKYEIQGFPTVFLFVDGEMRKTYEGERTKDGIVTWMKK

KASPSIHNITTVEEAERVLSAEPKVVLAFLDSLVGSESAELAAASRLEDDLSFYQTTSPDIAKLFEIETEVKRPALVLLKKEEEKLARFDGNFTKAAISE

FVSANKSPLVINFTREGASLIFENSVKNQLILFATTNESEKHLPTLREVAKSFKGKFVFVYVQMDNEDYGEAVSGFFGVTGTAPKVLVYTGNEDMRKFIL

DGELTVNNIKTLAEDFLADKLKPFYKSDPVPETNDGDVKIIVGNNFDEIVLDESKDVLLEIYAPWCGYCQSFEPIYNKLGKYLKGIDSLVVAKMDGTTNE

HPRAKADGFPTILFFPGGNKSFDPITVDVDRTVVELYKFLKKHASVPFKLAKPSATPEQVITTKKADEKTESDGAKDEL

>BrPDIL3-1_Bra018958

SSNHPGSDEESDDLEQLLAVDEQLQQDLPLHHQQSEAETVSRAQRIVLELSGDNARRVVGGNEFVMVLGYAPWC

ARSADLMPKFSEAATALKEIGSPVVMAKIDGDRYGKVASEMEIKGFPTLLLFVNGTSKAYTGGFSAEEIVIWVQKKTGAPIVTVNTVDEAQRFLKKYHTF

VVGLFNKFEGSEYNEFVKAAKSDDEIQFVETSDSEVAKLLFPEIKTSDVFIGMVKTEAERYTSYAGSYKMENILEFLSKNKFPLITKLSESNTAWVYSSP

VKLQVMIFAKADDFQNMAQPLENFARRFKSKLMFIYIDITNENLAMPFLTLFGIEHANKTVVAAFDNKLNSKYLLESDPSPTNIEDFCSGLADGTIPQYY

RSEPVPDNENASIVTVVGKTFDELVLNSQENVLLEVHTPWCVNCEAMSKQVVKLAKHFKGFENLVFARIDASTNEHAKLQVNDYPTILLYKSGEKEKPLK

ISTKLSAKDMAVFINEELKPRGGSAKDEL

>BrPDIL4-1_Bra004455

DDVVVLTEDSFEKEVGKDKGALVEFYAPWCGHCKKLAPEYEKLAASFKKAKSVLIAKVDCDEHKGVCTKYDVSGYPTI

KWFPKGSLEPQKYEGPRNAEALAEFVNKEGGTNVKLAAVPQNVVVLTPDNFDEIVLDQNKDVLVEFYAPWCGHCKSLAPVYEKVATVFKQEDGVVIANLD

ADAHKSLGEKYGVSGFPTLKFFPKDNKAGQDYDGGRDLDDFVTFINEKVGTSRDSKGQLTSKAGVVESLDALVKELVAASEDEKKAILSRIEEEASNLKG

STARYGKLYSSLAKKYIEKGSGYATKEAERLGRVLSKSMSPVKADELTLKRNILNTFVASS

>BrPDIL4-2_Bra000454

DDVVVLTDDSFEKEVGKDRGALVEFYAPWCGHCKKLAPEYEKLGASFKKAKSILIAKVDCDEHKSVCTKYGVSGYPT

IQWFPKGSLEPQKYEGARNAEALAEYVNKEGGTNVKLAAAPQNVVVLTPDNFDEIVLDQNKDVLVEFYAPWCGHCKSLAPVYEKVATVFKQEEGVVIANL

DADAHKSLGEKYGVSGFPTLKFFPKDNKAGQDYEGGRDLDDFVGFINEKVGTSRDSQGQLTSKAGIVESLDALVKELVAASEDEKKTILSRIEEEASNLK

GSTTRYGKLYSKLAKSYIEKGSAYATKEVERLGRVLGKSISPVKADELTLKKNILSTFVASS

>BrPDIL5-1_Bra005546

LYGSSSPVVQLTASNFKSKVLNSNGVVLVEFFAPWCGHCKALTPTWEKVASVLKGVATVAAIDADAHQSAAQDYG

IQGFPTIKVFVPGKPPVDYQGARDAKSIANFAYKQIKALLSDRLEGKSKPSGGGSSEKKSEPSASVELNSSNFDELVIKSNDLWIVEFFAPWCGHCKKLA

PEWKRAAKNLKGKVKLGHVNCDVEQSIMSRFKVQGFPTIMVFGVDKSSPYAYDGARSASAIESFATELVEASAGPVEVTELTGPDVMEKKCGSAAICFVS

FLPDILDSKAEGRNKYLEMLLSVAEKFKRHPYSFVWVAAVTQPDLEKRVNVGGYGYPAMVAMNVKKGVYAPLKSAFELQHLLEFVKDAGAGGKGNVPMNG

TPEIVETKAWDGKDGEVMEEDEFSLEELMGGDDDANVGTKDEL

>BrPDIL5-2_Bra015375

LYGSSSPVLQLTPSNFKSKVINSNGVVLVEFFAPWCGHCKSLTPTWEKVATTLKGIATVAAIDADAHKSVSQDYGVR

GFPTIKVFVPGKPPIDYQGARDAKAISQFAIKQIKALLKDRLDGKTTGTTTGGGSSEKKSEPSASVELNSSNFDELVTESKDLWIVEFFAPWCGHCKKLA

PEWKKAAKNLKGKVKLGHVDCDADKAIQSRFKVKGFPTILVFGADKSSPLPYEGARSASAIESFALEQLEANAGPAEVTELTGPDAMEEKCGPAAICFVS

FLPDILDSKAEGRNKYLEMLLSVAEKFKKDPISFVWVAAGKQPDLEKRVGVGGYGYPAMVALNAKKGAYAPLKSGFEVKHLIEFVKEAQKGGKGNLPIDG

TLEIVKTEAWDGKDGEVVDADEFSLEELMADD

>BdPDIL1-1_Bradi4g23180.1

EEAAAAEEAAPAAGEEAVLTLGTDNFDDAIAKHPFIVVEFYAPWCGHCKSLAPEYEKAAQLLSKHDPPIVLAKV

DANDEKNKPLAAKYEIQGFPTLKIFRNQGKNIQEYKGPREAEGIVDYLKKQVGPASKEIKAPEDASHLEDGKIHIVGVFAELSGPEFTNFLEVAEKLRSD

YDFGHTVHANHLPRGETAVERPLVRLFKPFDELVVDTKNFEVSALEAFIEASSTPKVVTFDKNPDNHPYLLKFFQGNSAKVMLFLNFSTGPYESFKSAYY

GAVEDFKDKEVKYLIGDIEASQGALQYFGLNADQAPLILIQDAESKKFLNSNIEADQIVSWLKEYFDGKLTPFRKSEPIPEANNEPVKVVVADNLDDVVF

KSGKNVLIEFYAPWCGHCKKLAPILDEAATTLQSEADVVIAKMDATANDVPGDFDVQGYPTLYFVTPSGKKVAYDGGRTADDIVEYIKKNKETAGQEAAA

ATEKAADPAATESLKDEL

>BdPDIL1-2_Bradi5g10610.3

EVEVAAVLEEAVLTLDVSNFSEVVGKLQFIVVEFYAPWCGHCKELAPEYEKAASMLRKHDPPVVLAKVDAYDEG

NKELKDKYEVHGYPAIKIIRNGGSDVSGYAGARNADGIVEYLKKQVGPASIELRSALDATRSIGDKGVVLVGIFPEFAGVEYENFMAVADKMRSDYDFFH

TSDASILPHGDQNVKGPLVRLFKPFDELFVDSQDFDKDAIKKFIEVSGFPTVVTFDDEPTNHKFLERYYSTPSAKAMLFLRFSDDRVEAFKSQMHEAARQ

LSGNNISFLIGDVSAAERAFQYFGLKESDIPLLLVIASTGKYLNPTMDPDQLIPWMKQYIYGNLTPYVKSEPIPKVNDQPVKVVVADNIDDIVFNSGKNV

LLEFYAPWCGHCRKLAPILEEVAVSFRNDEDIVIAKMDGTANDVPTDFVVEGYPALYFYSSSGGEILSYKGARTAEEIISFIKKNRGPKAGALEEVTQTD

AVQEEVTSTSSPSESVKDEL

>BdPDIL2-1_Bradi3g00210.1

DEDLDYIIHNAAADDLPADDEWLQEGSDDDQEESDPFHQGDIDETHVFLLTAANFSDFLSSRRHVMVEFYAPWC

GHCQALAPDYAAAASQLALLHQDVVALSKVDATEDADLAQKYDVQGFPTILFFIDGVPKDYTGERTKEAIVAWINKKLGPGVHNVTTVDEAEKIITGEDK

AVLAFLDSLSGAHSNELAAASRLEDTINFYQTSNPDVAKLFHIDPAAKRPSVVLLKKEEEKLTIYEGEFRASAIADFVSANKLPLITILTQETGPSIFDN

PIKKQILLFAVANESSEFLPIFKEVAKPFKGKLLFVFVERDNEEVGEPVANYFGITGQETTVLAYTGNEDAKKFFLDGEMSLDNIKKFAQDFLEDKLTPF

YKSEPIPEPNDEDVKIIVGKNLDQIVLDESKDVLLEIYAPWCGHCQSLEPTYNKLAKHLRGIDSLVIAKMDGTTNEHPRAKPDGFPTILFYPAGKKSFEP

MTFEGDRTVVEMYKFIKKHASIPFKLKRPDSSAARTERAESSGSTEGEKSSGSNLKDEL

>BdPDIL3-1_Bradi1g48460.1

RLDLGEDDDSEVLEALLAVDEEEEDEAPEGAKRAGGAEAVRRTQSMVLVLDNENARRAVEEHAELLLLGYAPWCER

SAQLMPRFAEAAAALRAMGSAVAFAKLDGERYPKAAADVGVSGFPTVLLFVNGTEHAYTGLHTKDALVTWVRKKTGAPVIRLQSRDSAEEFLKKDQTFAI

GLFKNYEGADHEEFVKAATTENEVQFVETNDRNVAKILFPGIASEEQFLGLVKSEPEKFEKFDGAFEENAILQFVELNKFPLITVFTDLNSGKVYGSPIK

LQVFTFAEAYDFEDLESLLQEVARGFKTKIMFIYVDTAEEKLAKPFLTLYGLEGDKPTVTAFDTSKGSKYLMEADINAKNLKEFCSGLLDGTLPPYFRSE

PVPQEKGLIGKVVGRTFDSSVLESPHNVFLEAHAPWCVDCEAISKNVEKLAKHFSGLDNLKFARIDASVNEHPKLQVNDYPTLLLYPAEDKSNPIKVSKK

LSLKDMAKFIKVKLHISDVDIKEKEPASDVEAVAATDSVKDEL

>BdPDIL4-1_Bradi2g12560.1

DGDDVVVLTEGTFEKEVGQDRGALVEFYAPWCGHCKKLAPEYEKLGASFKKARSVMIAKVDCDEHKSVCSKY

GVSGYPTIQWFPKGSLEPKKYEGQRTAEALAEFVNKEGGTNVKLATIPSSVVVLTPETFDSVVLDETKDVLVEFYAPWCGHCKHLAPIYEKLASAFKLDD

GVVIANVDADKYKDLGEKYGVTGFPTLKFFPKGNKAGEDYDGGRDLGDFTKFINEKCGTSRDTNGQLTSEAGRIASLDTLAKEFLSVASDKRKEVLSSIE

EEVAKLSGSAAKHGKVYVTIAKKILDKGNDYTKKETERLHRILEKSISPSKADEFIIKKNVLSTFSS

>BdPDIL4-2_Bradi2g35020.1

EGDEVLALTESTFDKEVGQDRAALVEFYAPWCGHCKKLAPEYEKLAASFKKAKSVLIAKVDCDEHKSVCS

KYGVSGYPTIQWFPKGSLEPKKYEGQRTAEALAEYVNSEAATNVKIAAVPSSVVVLTEETFDSVVLDETKDVLVEFYAPWCGHCKSLAPVYEKVASAFKL

EDGVVIANLDADKHTSLAEKYGVSGFPTLKFFPKGNKAGEEYEGGRDLEDFVKFINEKSGTSRDSKGQLTSEAGLVASLDALVKEFHSAADDKRKEVLSK

IEEEAAKLSGSAAKHGKIYVNAAKKIIEKGSDYTKKETERLHRMLEKSISPSKADEFVIKKNILAIFSS

>BdPDIL5-1_Bradi4g31830.1

LYSAGSPVLQLNPNNFKKVLNANGVVLVEFFAPWCGHCKQLTPTWEKAAGVLKGVATIAALDADAHKELAQQYGIQGF

PTIKVFIPGKPPVDYEGARDVKPIVNFALQQVKSLLKDRLDGKTSGGSSGKTSGGSSEKKTDTNESIELNSSNFDELVIKSKDLWIVEFFAPWCGHCKKL

APEWKRAAKNLKGQVKLGHVDCDSDKSLMSKYKVEGFPTILVFGADKESPFPYQGARAASAIESFALEQLEANSAPPEVSELTSSDVMEEKCASAAICFV

SFLPDILDSKAEGRNKYLELLLSVAEKFKKSPYSFVWTGAGKQADLEKQVGVGGYGYPAMVALNVKKGAYAPLRSAFQRDEIIEFVKEAGRGGKGNLPLD

GAPTVVQSGPWDGKDGEVIEEDEFSLEELMGDNSPPNDEL

>OaPDI_ABS11216.1

EEEEEVPASSNAGFPNYENYDDLEDDDSAASSEDGHESYSPPPVDEKDVVVLKESNFSDFIANNKYVLVE

FYAPWCGHCQALAPEYAEAATELKNGGEEVVLAKVDATEDGELAQKYEVQGYPTIYFFVEGIRKPYTGQR

TKDSIVSWLKKKTGPGLKNITTTEDAETILAAETKVVLGFLDALVGSSSDELAAASRLEEDVNFYQTSNP

DVAKLFHIDPQAKRPALVLIKKEAEKINHFGGQFTKAEISDFVYKNKLPLVTNFTRESAPLIFESPIKKQ

LILFTTSSDSEKILPTFQEAAKVFKGKLIFVYVELDNEDVGKPVSDYFGVQGDAPQVIAYTGNDDARKFK

LDGDVILSSIKLFGEKFLQDDLKPFFKSDPIPEKNDGDVKIVVGDNFDEIVLDESKDVLLEIYAPWCGHC

QMLEPTYNKLGKHLRGIDSLVIAKMDGTTNEHHRAKPDGFPTILFFPAGNKSFDPIAFDGDRTVVELYKF

LKKHATHPFKIQKPATSSPQTKGSGVSQDESSTSKDLKDEL

>ZmPDIL1-1_GRMZM2G091481_T01

EEEPAAAAEGEAVLTLDVDSFDEAVAKHPFMVVEFYAPWCGHCKKLAPEYENAAKALSKHDPPIVLAKVDANEEKNR

PLATKYEIQGFPTIKIFRDQGKNIQEYKGPREADGIVDYLKKQVGPASKEIKSPEDATALIDDKKIYIVGIFAEFSGTEFTNFMEVAEKLRSDYDFGHTL

HANHLPRGDAAVERPLVRLLKPFDELVVDSKDFDVAALMKFIDASTIPRVVTFDKNPDNHPYLMKFFQSSAPKAMLFLNFSTGPFDSFKSAYSAAAEEFK

DKEIKFLIGDIEASQGAFQYFGLKEDQTPLILIQDGDSKKFLKVHVEADQIVAWLKEYFDGKLTPFRKSEPIPEVNNEPVKVVVADNVHDFVFKSGKNVL

IEFYAPWCGHCKKLAPILDEAATTLQSDEEVVIAKMDATANDVPSEFDVQGYPTLYFVTPSGKVTSYDSGRTADDIVDFIKKSKETAGAATTTTTQAPPA

SEKAAAAEPVKDEL

>ZmPDIL1-2_GRMZM2G163421_T01

EEPAAAEAEAVLTLDVDSFDEAVAKHPFMVVEFYAPWCGHCKNLAPEYENAAKELSKHDPPIVLAKVDANEEKNRPL

ATKYEIQGFPTLKIFRNQGKNIQEYKGPREADGIVDYLKKQVGPASKEIKSAEGVAAHFDDKKIYIVGIFKEFSGTEFTNFMELAEKLSSDYDFGHTLHA

NHLPRGDASVEGPLIRLLKPFDDLVVDSKDFDVAALEKFIDASSTPRVVTFDNNPDNHPYLMKFFQSSAPKAMLFLNFSTGPLDSFKSVYYAAAEEFKDK

EIKFLIGDIEASQGAFQYFGLKEDQTPLILIQDGDSKKFLKDHIEADQIVSWLKEYFDGKLTPFKKSEPIPEVNNEPVKVVVADNIHDVVFKSGKNVLIE

FYAPWCGHCKKLAPILEEAATTLLSDEEVVIAKMDATANDVPSEFEVQGYPTMYFVTPSGKVTSYDSGRTADDIVDFINKSKETASAVQATATASGKAAD

AAEKTEPVKDEL

>ZmPDIL2-1_GRMZM2G134889_T01

AGSNMDEEVVDDLQYLIDNSDDIPTNDPDGWPEGDYDDDDLLFQDQDQDLTGHQPEIDETHVVVLAAANFSSFL

ASSHHVMVEFYAPWCGHCQELAPDYAAAAAHLAAHHHQAHLALAKVDATEETDLAQKYDVQGFPTILFFIDGVPRGYNGARTKEAIVDWINKKLGPAVQN

VTSVDEAQSILTGDDKAVLAFLDTLSGAHSDELAAASRLEDSINFYQTSTPDVAKLFHIDAAAKRPSVVLLKKEEEKLTFYDGEFKASAIAGFVSANKLP

LVTTLTQETSPSIFGNPIKKQILLFAVASESTKFLPIFKEAAKPFKGKLLFVFVERDSEEVGEPVADYFGITGQETTVLAYTGNEDARKFFLDGEVSLEA

IKDFAEGFLEDKLTPFYKSEPVPESNDGDVKIVVGKNLDLIVFDETKDVLLEIYAPWCGHCQSLEPTYNNLAKHLRSVDSLVVAKMDGTTNEHPRAKSDG

YPTILFYPAGKKSFEPITFEGERTVVDLYKFIKKHASIPFKLKRQESRTESTRAEGVKSSGTNSKDEL

>ZmPDIL2-2_GRMZM2G033829_T01

AGSNKAEEVDDLQYLIDNSEDIPPNDPDGWPEGGGGGDYDDDLLFQDQDQDLPDYEPQIDETHVVVLTAANFSSFL

AATRHVMVEFYAPWCGHCRELAPEYAAAAAHLAVHHNQTDLALAKADATEETDLAQRYDVQGFPTIILFIDGVPKDYNGARTKDAIVDWINKKLGPAVQD

VTSVHEAERILTGDDKAVIAFLDTLTGAHSDELAAASRLEDSINFYQTSIPDVAKLFHIDPAAKRPSIVLLKKEEEKLTFYDGKFKASAIADFVSANKLP

LVTTLTQETSPSIFGNAIKKQILLFAVASESSKFLSIFKEAAKPFKGKLLFVFVERDNDEVGEPVANYFGLTGQETTVLAYTGNEDARKFFLDGEVSLEA

IKDFAEGFLEDKLTPFYKSEPVPESNDGDVKIVVGKSLDVIVLDESKDVLLEIYAPWCGHCQSLEPTYNKLAKHLSGVDSLVIAKMDGTTNEHPRAKSDG

YPTILFYPAGKKSFEPVTFEGERTVVDMYRFIKKHASIPFKLKRQESRRESIQTDGVKDEL

>ZmPDIL3-1_GRMZM2G014076_T01

ARLDLDDDDDSGVLDELLAIDEEAERGGLLDAEGAGEAVRRAQSMVLALDNDNARRAVEDHAELLLLGYAPWCERSAQL

MPRFAEAAAALRAMGSAVAFAKLDGERYPKAAAAVGVKGFPTVLLFVNGTEHAYHGLHTKDAIVTWVRKKTGVPIIRLQSKDSAEEFLKKDMTFVIGLFK

NFEGADHEEFVKAATTDNEVQFVETSDTSVAKVLFPGITSEEKFVGLVKSEPEKFEKFDGKFEEKEILRFVELNKFPLITVFTELNSGKVYSSPIELQVF

TFAEAYDFEDLESMVEEIARAFKTKIMFIYVDTAEENLAKPFLTLYGLESEKKPTVTAFDTSNGAKYLMEADINANNLREFCLSLLDGTLPPYHKSEPLP

QEKGLIEKVVGRTFDSSVLESHQNVFLEVHTPWCVDCEAISKNVEKLAKHFSGSDNLKFARIDASVNEHPKLKVNNYPTLFLYLAEDKSNPIKLSKKSSV

KDMAKLIKEKLQIPDVETVAAPDNVKDEL

>ZmPDIL4-1_GRMZM2G128171_T03

DGDDVVALTESTFEKEVGKDRGALVEFYAPWCGHCKKLAPEYERLGASFKKAKSVLIAKVDCDEHKSLCSKY

GVSGYPTIQWFPKGSLEPKKYEGQRTAEALAEFLNTEGGTNVKLATIPSSVVVLTPETFDSIVLDETKDVLVEFYAPWCGHCKSLAPTYEKVASVFKLDE

GVVIANLDADKHRDLAEKYGVSGFPTLKFFPKGNKAGEDYDGDRDLVDFVKFINEKSGTSRDTKGQLTSEAGRIASLDVLAKEFLGASGDKRKEVLSSME

EEADKLSGSAARHGKVYVTIAKKILEKGNEYTEKETKRLDRILEKVGNAYLARCLMKHPLLGQLTVQI

>ZmPDIL4-2_GRMZM2G159369_T01

DEVVALTEADFEKEVGQDRGALVEFYAPWCGHCKKLAPEYEKLGASFKKAKSVLIAKVDCDEHKSVCSKYG

VSGYPTIQWFPKGSLEPKKYEGQRSVEALAEFVNSEAGTNVKIAAIPSSVVVLTSETFDSIVLDETKDVLVEFYAPWCGHCKHLAPIYEKLASVFKQDDG

VVIANIDADKHTDLAEKYGVSGFPTLKFFPKGNKAGEDYDGGRDLDDFVKFINEKCGTSRDPKGHLNQEAGLVPSLNPLVKEFLNAADDKRKEVLSKIEE

DVAKLSGSAAKHGKIYVTAAKKIIDKGSDYTKKETERLHRMLEKSISPSKADEFIVKKNILSIFSS

>ZmPDIL5-1_GRMZM2G389173_T01

LYSAGSPVLQLNPNNFKSKVLNSNGVVLVEFFAPWCGHCKQLAPAWEKAAGVLKGVATVAALDADAHQALAQEYGIKGF

PTIKVFSPGKPPVDYQGARDVKPIVEFALSQVKSLLRDRLSGKASAGSNGKTSGGSSEKSEPSASVELNSRNFDELVVKSKDLWIVEFFAPWCGHCKKLA

PEWKKAAKNLKGQVKLGHVDCDAEKSLMSKYKVEGFPTILVFGADKESPFPYQGARVASAIESFALEQLEANSGPAEVSELTGPDVMEEKCASAAICFVS

FLPDILDSKAEGRNKYLELLLSVAEKFKKSPYSFVWTAAGKQANLENQVGVGGYGYPAMVALNVKKGAYAPLRSAFQRDEIIEFVKEAGRGGKGNLPLND

APTVVASEPWDGKDGEVIEEDEFSLDELMGDSSSANDEL

>AePDIL1-1

EEAAAAEEAAAAPEAVLTLHADNFDDAIAKHPFILVEFYAPWCGHCKSLAPEYEKAAQLLSKLDPAIVLAKVDANDEKNKPLASKYEVQGFPTLKIFRNGGKNIQEYKGPREAEGIVEYLKKQVGPASKEIKAPEDATYLEDGKIHIVGVFTEFSGTEFTNFLEVAEKLRSDYDFGHTVHANHLPRGDAAVERPLVRLFKPFDELVVDSKDFDVSALEKFIDASSTPKVVTFDKNPDNHPYLLKFFQTNAPKAMLFLNFSTGPFESFKSAYYGAVEEFSGKDVKFLIGDIEASQGAFQYFGLKEDQAPLILIQDSDSKKFLKEQVEAGQIVAWLKDYFDGKLTPFRKSEPIPEANNEPVKVVVADNVHDVVFKSGKNVLIEFYAPWCGHCKKLAPILDEAAATLQSEEDVVIAKMDATANDVPSEFDVQGYPTLYFVTPSGKKVSYEGGGTADEIVDYIKKNKETAGQAAAADTEKAAEPAATEPLKDEL

>AePDIL2-1

AVPTSNPDIDLEYLIKNAGLDDPTPATTATDPEDDGAPDFPGLDADYDDEDLFGDDDGPEEDSSHPSAADEAHVLLLTAANFTSVLAARRHVMVEFYAPWCGHCRALAPHYAAAAAALAEQGVDVALAKVDATEDHDLAQAHGVQGYPTLLFFIDGVPRDYAGERTKDAIVAWISKKLGPAVQNLTTADEAEKIVTGDDVAVLAYLDHLSGAHSDELAAASRLEDTISFYQTTSPDVAKLFHIDPEAKRPSVVLLKKEEEKLTVFDGEFRASAIAEFVSANKIPLITTLTQETAPAIFDNPIKKQILLFAVAKESSKFLPIIKETAKSFKGKLLFVFVERDNEEVGEPVANYFGITGNETTVLAYTGNEDAKKFFFSGEISLDTIKEFAQDFLEDKLTPSYKSDPVPESNDEDVKVVVGKSLDQIVLDESKDVLLEVYAPWCGHCQSLEPIYNKLAKYLRGIDSLVIAKMDGTNNEHPRAKPDGFPTILFYPAGKKSFEPITFEGDRTVVEMYKFLKKHAAIPFKLKRPDSSAARTDGPGSTTEGEKSSGSNPKDEL

>AePDIL4-1

DGDEVLALTESTFEKEVGQDRGALVEFYAPWCGHCKKLAPEYEKLAASFKKAKSVLIAKVDCDEHKSVCSKYGVSGYPTIQWFPKGSLEPKKYEGQRTAEALTEYVNSEAATNVKIAAVPSSVVVLTEETFDSVVLDETKDVLVEFYAPWCGHCKSLAPIYEKVASVFKQDEGVVIANLDADKYTSLAEKYGVSGFPTLKFFPKGNKAGEEYESGRELDDFVKFINEKSGTSRDSKGQLTSEAGLVASLDALVKEFHGAADDKRKEILSKIEEEAAKLSGPAVKHGKIYVNVAKKILQKGSDYTKKETERLHRLLEKSISPSKADEFAIKKNILSAFSS

>AePDIL5-1

LYSAGSPVLQLNPNNFKKVLNANGVVLVEFFAPWCGHCKQLTPIWEKAAGVLKGVATVAALDADAHKELAQQYGIQGFPTIKVFLPGKPPVDYEGARDVKPIVNFALSQVKGLLRDRLDGKTSGGSSGKTSGGSREKKTEPNESVELNSSNFDELVVKSKDLWIVEFFAPWCGHCKKLAPEWKRAAKNLKGQVKLGHIDCDSDKSLMSKYKVEGFPTILVFGADKESPFPYQGARAASAIESFALEQLEANAAPPEVSELTSADVMEEKCASAAICFVSFLPDILDSMAEGRNKYLELLLSVAEKFKKSPYSFVWAGAGKQADLEKQVGVGGYGYPAMVALNVKKGAYAPLRSAFELAEITEFVKEAGRGGKGNLPLEGAPTVVQSEPWDGKDGEVIEEDEFSLEELMADSSAPNDEL

**E. Peptidylprolyl Cis-Trans Isomerase (PPIase) protein sequences**

>ctr10137_c0_g1_i1

GQVFRLSTVSNCDFRLLSVLFQVIKLYNLRTLNSLLITNKMNDADTYKSYLKRTVYVGGL

AEEVDDKVLRSAFIPFGDIVDVQMPLDYESEKHRGFAFVEFEQPEDALDSIDNMNEAEIF

GRTIRVNLAKPQKINRGSTRPVWSEDDWLVQYAGKTLEPKDDKKIEEDKTKDEAKNPQVY

LDIKIGKKDAGRIIIMLRADIVPRTAENFRCLCTHEKGFGYQNTTVHRIIPNFMCQGGDI

TNNNGTGGLSIYGKKFDDENFELKHTGPGVLSMANSGPNTNSSQFFICTARTEWLDNKHV

VFGHVLSGIDVMKKIEKCGTKAGLPTEKVIIGSCGQLA

>ctr11074_c0_g1_i1

MVNPRCFLDVSIGGEVEGRIVVELFNDVVPKTAENFRALCTGEKGIGPNTNVPLHYKGMC

FHRVIKGFMIQGGDISAGDGTGGESIYGSKFEDENFELKHERKGMLSMANAGPNTNGSQF

FITTTRTPHLDGKHVVFGKVLKGMGVVRSVEHVVTGENDRPTQEVVVVDCGEIAEGEDDG

VVNFFNDGDTLPDWPADLDVKPDEISWWMSSVDTIKGLGNEQYKKQDYKMALRKYRKALR

YLDVCWEKDDIDQEKSAALRKTKSQIFTNSSACKLKLGDLQGAILDSDFAMHDGDNAKAL

FRKGQAYMALNDLDAAVESFKKALELEPNDGGIKKEYAAAWKKVADRRDQEKKAYSKMFK

>ctr15543_c0_g1_i1

MKDSVMIHGAGGIPDKSWKPQFGILETSMGQITIELYWDHAPETCRNFAELCRRGYYNNT

KCHRIIRNFMIQGGDPTGTGRGGTSIYGTHFDDEIHDDLRHTGAGIVSMANSGPNTNGSQ

FFITLAPTQWLDKKHTIFGRVHSGMNVVKRMGMVETDKNDRPVDDVKILRGGIKM

>ctr15837_c0_g2_i1

MPTWNQIQSQLRNPQNPVVFFDISVGRTEIGRLIMELYADIVPKTSENFRQFCTGEHKKD

GIPFGYKGCCFHRIIKDFMIQGGDFVNGDGTGVMSIYGADTFSDENFKLNHDAPGLLSMA

NSGIDTNGCQFFITCANCNFLDGKHVVFGRVIDGLLVMRKIENVPTGPNNKPKIPIVISQ

CGQL

>ctr17099_c0_g1_i1

SISFIFTCLLVSKKSFEAYKMSNIYIQEPPTSGKVVLKTSFGDIEIELWSREAPKACRNF

VQLCTDGYYDGVLFHRIVKGFIAQGGDPTGTGAGGESIYGHPFKDEIHSRLRFNRRGLVA

MANAGKDDNGSQFFFTLGSTPELQTKHTIFGKVAGDTIFNLTKLNETLVDTEDKPVYDQK

ILKTIVLNSPFPDIVPRIEKKSEKTEKLSKKKEVVGVKNFKLLSFGEEAEEDEEELIDVV

KEFSGRPKSTHDVLQDPQLSSEPAIIDNELTDDITIPSSDQTSKISIDDVRSKLSSNSKN

KKSAPKSVEQTNEEDSDDYELGKDLKEEKKRKAEEIRKEINEIKSQLSKKKKDKKKPSLD

IDDIPSIPALPIDPLKQQYLENIKMYSSKKSEIPKKGSNREEFALGLLEQFKNKLHNAIQ

SSQEKPEKPTETVEEEDDDWKSHPLQFQKEDAVLAKDANKKDDDWFEIYDPRSAINKRKR

ELNKLNSKTDKDKKTKV

>ctr17583_c0_g1_i1

MGLPRVFFDMTADNQPSGRIVIELRSDVVPKTVENFRALCTGEKGFGYKGSSFHRIIPNF

MCQGGDFTNHDGTGGKSIYGRKFEDENFELKHTGPGVLSMANAGKNTNGSQFFITTVKTS

WLDNAHVVFGQVVEGMDVVKQLESYGSQSGKTSKKIVIADCGQLS

>ctr18375_c0_g1_i1

LALASIRRLSINSTFNSFSHLVATARPVTPFFSPFVKDFSSKAKKMGLPRCFFDMSIDNQ

PAGRIVIELRNDVVPKTAENFRCLCTGEKGFGYKGSSFHRVIPGFMCQGGDFTNHNGTGG

KSIYGNKFEDENFQLKHTEPGVMSMANAGPGTNGSQFFITTVKTSWLDNRHVVFGKVVEG

MDIVKKLESYGTQSGKTTKKITIANCGEM

>ctr18912_c0_g1_i1

MGVDTELVKTHVRPRCFMDVAVDNILLGRIVFELFDDFCPLTCENFRALCTGEKGLGKTT

GKPLHFQGVIFHRVVKSFMVQCGDFSTGNGTGGESIFGGTFPDENFDLKHDQPFLLSMAN

RGPDTNGSQFFITTQPTPHLDGIHVVFGRVVSGQPVVMQIEELGVDKNSRPLQDAKVVKC

GELILKSKIKQSVKSASDSETSSGSDDDKKRKRKKKKKEKESKKRTEKKLDRSKEEDDDA

EAEGQLHPLVSVSNIDPEEIPAGPPNRFLSRGGPSSMFLMKPQSDNDDRSKKRNRIRGVT

KSGRIVKGRGTLRFRTPSRSRSRSYTPPHWRQEQKKMITFNEFEKMEIERKEKEEEIKRR

EEARKKRHEEKEKREKGKNGQTPPGSPPKGSEEQNDKIQIDMMAKIEDVFQIIRDRNMKN

GSDNHDHKRTDQRLSDIKKKSKGENREKIQKMDKKFDRRSRSRSPKRRNDDSFRRDRVQR

DGNRYRKDDRHNNRFTQGKPRNRSRDREHFEKKKKNHRRSSSSSSEDRKNKKKVRNENSF

SLSPEVKD

>ctr19397_c0_g1_i1

MSEKREHEDDDKSIKSNASEDDDDECVGPSLSELTDTVEKKKRKILKYEHLYLENLPTCD

TYEKSYMHRDVITHIIITKTDFLITASCDGHVKFWKKTEELIEFVKHFRSHLTPVTDITD

NYNGTLMCTISSDQTVKVFDVINFDMINMIKLDYTPLSACWVHSKGDPIHTVAISSATEP

KIFIYDGKGVNIPLHVIEKIHTKPIVFVKYNFVFDVAISADKSGIIEYWSGAKTDYKFPK

CVQFDSKLDTDLFEFVRHKTHPTSLCFSDDGLKFATMSPDRKVRVFNLLTGKLSRVYDES

LARFSELQQKKQQIPNIEFIRRMAMERELDKTEISHTANLCFDESGHYLFYPTMLGVKVV

NIFNNTLVKIIGKPENLRALRVALFQGKAKKPKAAVTLEMEAATNPTLESSSSDPTLFCT

AYKKNRFYMFTKREPEDIKSIDADRDVFNERPSKDDIISNTEAACIQRLYDTATLHTVFG

DIQVALFKECQKTVENFCVHSKNGYYNGNIFHRVIKGFMIQTGDPTGTGLGGESIWGGEF

EDEIRSHLKHDRPYTLSMANAGPNTNGSQFFITLIPTPWLDNKHTVFGRVTKGMEVVQTI

CSAKTHPKTDKPHDDVQIINISLK

>ctr20383_c0_g1_i1

MGKRQHQKDKMYLTYTEWSTLYGGKRPGIEKPKFARLPFDHCCLSLVAFKTPYCDPDGNI

FEYEALLEYIKQFKHNPVTGKPIELKKLIKLNFHRNAAGEYHCPVLFKSLTKHSHIIAIK

TTGNVFSYEAIEQLNIKTKNWKDLLTDEPFLRKDMITLQDPTNLTKFNIAKFHHVVKKIK

VVDPDEEAQSKDPEGRLKSVSKTTRDILDTLDRDYKEPTKKVEVVAQKPDKLNAAHYSTG

RVAASFTSTAMVPVLEHESAIIEENEIRFERIKKKGYVRLTTNCGPLNLELFCKEVPKTC

ENFIKLCQKDYYDGTKFHRSIRNFMVQGGDPTGTGKGGESYWGQPFEDEFKQNLNHSGRG

VLSMANSGPDTNKSQFFITYRSCKHLDNKHTVFGRIVGGFETLNAIEEIEVDNKDRPITD

IIILKTHVFVDPFQEVDDKIAEERQEELINTPGTSMASTVQTEENKISSIAIPGVKLKTF

KSGVGKYINPSALQKNKIENEELPNKKKKVIGYDFHGFNNW

>ctr22690_c0_g1_i4

MWSRGFSFWIKGCTLFFLIVVISGLEPELGSTRVVFQTNYGDIEFGFFPTVAPKTVEHIF

KLVRLGGYNTNHFFRVDKGFVAQVADVTGGRSAPMNEEQKREAEKTVVGEFSEVKHVRGI

LSMGRYDDPDSGSSSFSILLGNAPHLDGKYAIFGKVTKGDETLTKLEQLPTRREGMFVMV

CLNSLSIFCVILFPRH

>ctr22690_c0_g1_i5

MWSRGFSFWIKGCTLFFLIVVISGLEPELGSTRVVFQTNYGDIEFGFFPTVAPKTVEHIF

KLVRLGGYNTNHFFRVDKGFVAQVADVTGGRSAPMNEEQKREAEKTVVGEFSEVKHVRGI

LSMGRYDDPDSGSSSFSILLGNAPHLDGKYAIFGKVTKGDETLTKLEQLPTRREGMFVMP

IERITILSSYYYDTETENCEQDRSILKRRLAASAVEVERQRMKCFP

>ctr22957_c0_g1_i1

MISTYKIMTLLKWAILPLTVFTIFVVMYSHASEDLQKKGPKVTDIVWFDIKMGSSEPQRV

EIGVFGATVPKTAQNFIELAKKPEGEGYKGSKFHRVIKDFMIQGGDFTKGDGTGGRSIFG

EKFADENFKLKHYGAGWLSMANAGKDTNGSQFFITTKQTSWLDGRHVVFGKVIKGMKTIR

AAEAAETDSRDKPVDGIVIVDSGHTVVPEPYPVAKTDAVE

>ctr22957_c0_g1_i2

LSCRVWSTRVFLTLLSPVKLLFNFGTYYICKSCMISTYKIMTFLKWAILPLTVFTIFVVM

YSQALEDPQKKGPKVTDIVWFDIKMGSSEPQRVEIGVFGATVPKTAKNFIELAKKPEGEG

YKGSKFHRVIKDFMIQGGDFTKGDGTGGRSIFGEKFADENFKLKHYGAGWLSMANAGKDT

NGSQFFITTKQTSWLDGRHVVFGKIIKGMKTIRAAESAETDSRDKPVENIVIVDSGHIVV

PEPYPVSKTD

>ctr22957_c0_g1_i3

LSCRVWSTRVFLTLLSPVKLLFNFGTYYICKSCMISTYKIMTLLKWAILPLTVFTIFVVM

YSHASEDLQKKGPKVTDIVWFDIKMGSSEPQRVEIGVFGATVPKTAQNFIELAKKPEGEG

YKGSKFHRVIKDFMIQGGDFTKGDGTGGRSIFGEKFADENFKLKHYGAGWLSMANAGKDT

NGSQFFITTKQTSWLDGRHVVFGKIIKGMKTIRAAESAETDSRDKPVENIVIVDSGHIVV

PEPYPVSKTD

>ctr24033_c0_g1_i1

MAAASAFTIPTLRLSNVTESRCRMGGLKRNPNLNLRLRGGCGMRVGVNVRERKGATVKVK

VRASSEEGDVQSKVTNKVYLDISIGNPVGKLAGRIVIGLYGDVVPQTAENFRALCTGEKG

FGYKGSTFHRVIKDFMIQGGDFDKGNGTGGKSIYGRTFKDENFKLTHTGPGVVSMANAGP

NTNGSQFFICTVKTPWLDQRHVVFGQVLEGMDIVRLIESQETDRGDRPRKKVTISDCGEL

PIA

>ctr24500_c0_g1_i2

MGRRQNDSDFGRFAIFILLLIGTISCSTVYLFLKVVLRPTSTAPVTVSEKGDGVGLVVND

DDDLRGSEEGQCCRGIEHLELWGEAVKWGSDFKVNSSEDCCMACKGMCKDDGGPCLCNSW

VFCSDREACGPRFGECWLKRQQDALNPDRRDSGDLVMWTSGFVFNKEEGIVGLETDHGIL

RVELLPECAPHSVAYILELLALPHCVGCQIHRAESRGSFWDSKGNHIEKAPYGPPFALIQ

GTLESYGSIFNDIPKEHCPAIRRGSVAWVGSGPEFFISLVDHEEWRKSYTVFGYVLSEDM

GILEKISQLPTKSEVWNNINVSVLENPVSVRFRRMNTKS

>ctr24674_c0_g1_i1

PTHRLLPVDVDSPTPDSDCLALNLDGLVVAWSWLLRQARKMRSASCAVRSLGVALLVLLG

FLVVAQCQDKKGPKVTDTVWFDIKIGEENVGRVEIGVFGKTVPKTAANFVELAKRGEGEG

YKGSKFHRVIKDFMIQGGDFTRGDGTGGKSIYGERFEDENFKLKHYGAGWLSMANAGKDT

NGSQFFITTKQTPWLDGRHVVFGKVIKGMSVVRRIESTSTDSRDRPSQDVIIVDCGVIHA

AEPFSVDKSDATE

>ctr24674_c0_g1_i2

PSGASCVSPVADPHRPDGRRPSRLRKMGFAAFVVVLVGVLAVAHCQEGPKVTDTVWFDIK

IGEQNVGRIEIGVFGKTVPKTAANFVELAKRGEGEGYKGSKFHRVIKDFMIQGGDFTRGD

GTGGRSIYGERFEDENFKLKHYGAGWLSMANAGKDTNGSQFFITTKQTPWLDGRHVVFGK

VIKGMSVVRRIESTSTDSRDRPSQDVIIVDCGVIHAAEPFSVDKSDATE

>ctr24674_c0_g1_i3

PSGASCVSPVADPHRPDGRRPSRLRKMGFAAFVVVLVGVLAVAHCQEGPKVTDTVWFDIK

IGEQNVGRIEIGVFGKTVPKTAANFVELAKRGEGEGYKGSKFHRVIKDFMIQGGDFTRGD

GTGGRSIYGERFEDENFKLKHYGAGWLSMANAGKDTNGSQFFITTKQTPWLDGRHVVFGK

VIKGMSVVRRIESSSTDARDRPSQDVTIVDCGVIHAAEPFSVEKDDATE

>ctr24700_c0_g1_i1

MEEHENGANGTEEEALIGPGPAPRSRPKRPLQFEKAYLDALPSANMYEKSYMHRDVVTHV

AVSPAEFFITGSADGHLKFWKKRPIGIEFAKHFRSHLGPIEGLAVSADGLLCCTISNDRS

VKIYDVVNFDMMVMIRLPYVPGAVEWVYKQGDVKAKLAISDRNSPFVHIYDARAGSNDPI

ISKEIHMAPIKVMKYNQLHDTVLSADAKGIIEYWDPATLQFPENEVNFKLKSDTNLFEIV

KCKTSVSAIEVSPDGKQFSVTSPDRRIRVFWFRTGKLRRVYDESLEVAQDLQRSDAPLYR

LEAIDFGRRMAVEKEIEKTESAPLPNAVFDESSNFLIYATLLGIKVVNLHTNKVARILGK

VENNDRFLRIALYQGDQSSKKVRKIPSVAANANESKEPLTDPTLLCCAFKKHRIYLFSRR

EPEEPEDATKGRDVFNEKPPADELLAVSDIGKAVTTSLPDNVILHTTMGDIHMKLYPEEC

PKTVENFTTHCRNGYYDNLIFHRVIKGFMIQTGDPLGDGTGGQSIWGREFEDEFHKSLRH

DRPFTVSMANAGPNTNGSQFFITTVATPWLDNKHTVFGRVAKGMDVVQAIEKVKTDKTDK

PYQDVKILNVTVPKA

>ctr24700_c0_g1_i2

MHAIKHMTCKYKSYLYLSFGVCLVSKFWSLSCISRREPEEPEDATKGRDVFNEKPPADEL

LAVSDIGKAVTTSLPDNVILHTTMGDIHMKLYPEECPKTVENFTTHCRNGYYDNLIFHRV

IKGFMIQTGDPLGDGTGGQSIWGREFEDEFHKSLRHDRPFTVSMANAGPNTNGSQFFITT

VATPWLDNKHTVFGRVAKGMDVVQAIEKVKTDKTDKPYQDVKILNVTVPKA

>ctr25168_c0_g1_i1

MGRRRCFLDICIGEELEGRIVVELYHDVVPKTCENFRALCTGEKGIGPNTGVPLHFKGSC

FHRVIKGSMIQGGDISAGDGTGGESIYGLKFEDENFELKHERKGMLSMANSAPNTNGSQF

FITTTRTSHLDGKHVVFGKVVKGMGVVRSIEHVAIGDDDRPVLDVKIVDCGEILEGEDDG

ISNFFKDGDTYPDWPADLDESPDELDWWMKSVDSIKSFGNEYYKKQDYKLALRKYRKALR

YLDICWEKDGIDEEKSSSLRKTKSQIFTNSSACKLKLGDLKGALLDTEFAMRDGDNNAKA

LFRQGQAYMALHDIDAAVESFKQAQTLEPNDAGIRRELAAARKKIADRRDQEKKAYSKMF

Q

>ctr25226_c0_g1_i11

MSQEKNPRVFFDVSIDGDPVERIVIQLFASVVPKTAENFRALCTGEKGIGESTGKPLHYK

GTCFHRIIKGFMAQGGDFSRGNGTGGESIYGGKFADENFKLAHDGPGVLSMANSGPNTNG

SQFFITFKRQPHLDGKHVVFGKVIKGMDILKKIEQVGTSDGKPTQPVKIVDCGEFSEAKS

RYTVEKEKGKRRKSGKSLTSDDSSDKKSRGKRKRSSKDTRKKRRYSTSDSDTSSDSYDSD

SESDSDSDSESESSDSDSSSSYGKHQKRKRSKRKHGKKRKVGRKQKRRSHHSRSRRSRHK

SRWSSGSSDSESESSSASGRSSGDEKADRHVSGRKTHADSKAQKNPDTGKRSCSLSQQNQ

PITGQGTDPKNRRTLDKQSHEEGELSPENGAFLSNGHDTQAEFSKPAKQHSYSDDSNHDR

GGSPGRSPARSPPRNSREVNQGRASLASPGEKASEPAEPKNSRGISKSPSPNGMPKRIKK

GRGFTERYAFARRYRTPSPEQSPRIYRYGDKNIRRNFDRNTSYRSYSERSPPRRFRSPPR

DRSCPRYQSGRSRSRSISRSPGRGRYRDQGRSRSPVRSPSPEDRRPPISDRLKSRLGPRS

DPRSPERGRSKSNSRSNGSSRSRSPDATPPKRYDKRTSLSRSRSSSPSGQKGLVSYGDAS

PDSDAR

>ctr25226_c0_g1_i13

MSQEKNPRVFFDVSIDGDPVERIVIQLFASVVPKTAENFRALCTGEKGIGESTGKPLHYK

GTCFHRIIKGFMAQGGDFSRGNGTGGESIYGGKFADENFKLAHDGPGVLSMANSGPNTNG

SQFFITFKRQPHLDGKHVVFGKVIKGMDILKKIEQVGTSDGKPTQPVKIVDCGEFSEAKS

RYTVEKEKGTCNRHGY

>ctr25226_c0_g1_i15

MSQEKNPRVFFDVSIDGDPVERIVIQLFASVVPKTAENFRALCTGEKGIGESTGKPLHYK

GTCFHRIIKGFMAQGGDFSRGNGTGGESIYGGKFADENFKLAHDGPGVLSMANSGPNTNG

SQFFITFKRQPHLDGKHVVFGKVIKGMDILKKIEQVGTSDGKPTQPVKIVDCGEFSEAKS

RYTVEKEKGKRRKSGKSLTSDDSSDKKSRGKRKRSSKDTRKKRRYSTSDSDTSSDSYDSD

SESDSDSDSESESSDSDSSSSYGKHQKRKRSKRKHGKKRKVGRKQKRRSHHSRSRRSRHK

SRWSSGSSDSESESSSASGRSSGDEKADRHVSGRKTHADSKAQKNPDTGKRSCSLSQQNQ

PITGQGTDPKNRRTLDKQSHEEGELSPENGAFLSNGHDTQAEFSKPAKQHSYSDDSNHDR

GGSPGRSPARSPPRNSREVNQGRASLASPGEKASEPAEPKNSRGISKSPSPNGMPKRIKK

GRGFTERYAFARRYRTPSPEQSPRIYRYGDKNIRRNFDRNTSYRSYSERSPPRRFRSPPR

DRSCPRYFLIRPSILMNRLAITYSSMHKLSILLII

>ctr25226_c0_g1_i2

MSQEKNPRVFFDVSIDGDPVERIVIQLFASVVPKTAENFRALCTGEKGIGESTGKPLHYK

GTCFHRIIKGFMAQGGDFSRGNGTGGESIYGGKFADENFKLAHDGPGVLSMANSGPNTNG

SQFFITFKRQPHLDGKHVVFGKVIKGMDILKKIEQVGTSDGKPTQPVKIVDCGEFSEAKS

RYTVEKEKGKRRKSGKSLTSDDSSDKKSRGKRKRSSKDTRKKRRYSTSDSDTSSDSYDSD

SESDSDSDSESESSDSDSSSSYGKHQKRKRSKRKHGKKRKVGRKQKRRSHHSRSRRSRHK

SRWSSGSSDSESESSSASGRSSGDEKADRHVSGRKTHADSKAQKNPDTGKRSCSLSQQNQ

PITGQGTDPKNRRTLDKQSHEEGELSPENGAFLSNGHDTQAEFSKPAKQHSYSDDSNHDR

GGSPGRSPARSPPRNSREVNQGRASLASPGEKASEPAEPKNSRGISKSPSPNGMPKRIKK

GRGFTERYAFARRYRTPSPEQSPRIYRYGDKNIRRNFDRNTSYRSYSERSPPRRFRSPPR

DRSCPSSIFFSE

>ctr25239_c0_g1_i1

MISRHFRTSATIKSFFPLHARKFHSSVINMTVKTFFQIRWEGPVLQADGRKGNVGEQTGT

INFNLYDQVVPTTVENFRALCTGEKGFGYKGSSFHRIIPQFMLQGGDFTKGDGTGGKSIY

GDKFKDENFKLTHKQPGLLSMANAGPNTNGSQFFITTVTTGWLDGKHVVFGEVADDESMK

VVKALEAVGSGSGAIKYKLKPTIIDCGELK

>ctr25252_c0_g1_i1

MGKKQHSKDRMFITKTEWATEWGGAKSKENRTPFKRLPFYCCALTFTPFEYPVGTADGSV

FDVMNITPYVMKYGKHPVTGAPLKPQDLIPLTFHKNSEGEYHCPVLNKVFTEFTHIVAVK

TTGNVFCYEAVKELNIKTKNWKELLTDEPFTKDDLITVQSPNALDSKVLLEFDHVKNNLK

VDDEELQKMSSDPTYNINMSGDIKQMLKELGTEKGKETALHGGGGGKAQKERAAALAAIL

AARSNVKEDSKSNPNKEAGAPQPFSIVDAASASVHGRSAAAAKASSGDKTAARIAMHMAG

DRAPVNGKMVKSRFTTGAASRSFTSTSFDPVTKNDFEYVKVEKNPKKKGYVQLHTTHGDL

NIELHCDITPRACENFITLCERGYYNGVAFHRNIRNFMIQGGDPTGTGRGGESIWGKPFK

DELHSKLVHSGRGVVSMANSGPHTNGSQFFILYKSANHLNFKHTVFGGVVGGLTTLAAME

KVPVDDNDRPLVSHVFFPCRCNRLSSCFSRFYSSQMLCV

>ctr25252_c0_g1_i2

MGKKQHSKDRMFITKTEWATEWGGAKSKENRTPFKRLPFYCCALTFTPFEYPVGTADGSV

FDVMNITPYVMKYGKHPVTGAPLKPQDLIPLTFHKNSEGEYHCPVLNKVFTEFTHIVAVK

TTGNVFCYEAVKELNIKTKNWKELLTDEPFTKDDLITVQSPNALDSKVLLEFDHVKNNLK

VDDEELQKMSSDPTYNINMSGDIKQMLKELGTEKGKETALHGGGGGKAQKERAAALAAIL

AARSNVKEDSKSNPNKEAGAPQPFSIVDAASASVHGRSAAAAKASSGDKTAARIAMHMAG

DRAPVNGKMVKSRFTTGAASRSFTSTSFDPVTKNDFEYVKVEKNPKKKGYVQLHTTHGDL

NIELHCDITPRACENFITLCERGYYNGVAFHRNIRNFMIQGGDPTGTGRGGESIWGKPFK

DELHSKLVHSGRGVVSMANSGPHTNGSQFFILYKSANHLNFKHTVFGGVVGGLTTLAAME

KVPVDDNDRPLEEIKITSVTVFINPYTEPDEEEEQENTEEKNAEDEDNDKVGSWYSNPGA

GTSELGGTGVGGGVGKYLKARNAQAISATATVDTGTAVVAKKRKVGVVSNEFKDFSAW

>ctr25252_c0_g1_i4

MGKKQHSKDRMFITKTEWATEWGGAKSKENRTPFKRLPFYCCALTFTPFEYPVGTADGSV

FDVMNITPYVMKYGKHPVTGAPLKPQDLIPLTFHKNSEGEYHCPVLNKVFTEFTHIVAVK

TTGNVFCYEAVKELNIKTKNWKELLTDEPFTKDDLITVQSPNALDSKVLLEFDHVKNNLK

VDDEELQKMSSDPTYNINMSGDIKQMLKELGTEKGKETALHGGGGGKAQKERAAALAAIL

AARSNVKEDSKSNPNKEAGAPQPFSIVDAASASVHGRSAAAAKASSGDKTAARIAMHMAG

DRAPVNGKMVKSRFTTGAASRSFTSTSFDPVTKNDFEYVKVEKNPKKKGYVQLHTTHGDL

NIELHCDITPRACENFITLCERGYYNGVAFHRNIRNFMIQGGDPTGTGRGGESIWGKPFK

DELHSKLVHSGRGVVSMANSGPHTNGSQFFILYKSANHLNFKHTVFGGVVGGLTTLAAME

KVPVDDNDRPLEEIKITSVTVFINPYTEPDEEEEQENTEEKNAEDEDNVCSRVFRIIRSG

YFFSPL

>ctr26423_c0_g1_i5

MSLLFCTLLLFGTLALIEAKKSKEDLKEVTNKVYFNVEIGGKEAGRIVIGLFGKAVPKTT

ENFRALCTGEKGVGKSGKPLHYQGSSFHRIIPSFMIQGGDFTHGNGMGGESIYGEKFADE

NFKLKHTGPGVLSMANAGPDTNGSQFFITTVTTSWLDGRHVVFGKVLSGMDVVYKIEAEG

TQSGTPKSKVVIADSGELPL

>ctr26897_c1_g2_i13

MARKKNPLVFMDVSIDGDPVERMVFELFYDVAPKTAENFRALCTGERGISSNSGKSLHYK

GSFFHQIVEGSFVQGGDFINRNGTGGESIYGSKFPDESPRLKHDAPGLLSMAIADRDTLG

SHFSITLKADHHLDRKHVVFGKLVQGHNVLKKIEDVGDEEGRPAVTVKIINCGEYNEDGK

KVNKSKFGKDGSSEANSHETRRKGKHKRSSKDKRKRRRYYSSESGSSSDSDMESSETDSD

SESDLSSSSDISSSSDDRRRKRKRSKKDKHRRGKRRDKRRDKRRRRRDKRSKRKSRRESG

SDSDSETNSDSSSEDEGIDAQLKGMKRKDHSQKNAAELKSPMVMEKDLPSVHQEKEELGM

PENEDKFSKENGERHSNGMGADHRSGRSEERQPDMMDDHSGKSRSTFLFSHTSVISYAYH

FFQIWYFIKS

>ctr27050_c0_g3_i1

MSVVLETTIGDLTIDLFIKERPHSCKNFLKLCKLKYYNFCLFHHVQSNFVAQTGDPTGTG

SGGQSVYGIVSGKDARYYEGEKKPKIKHDRPGLVSMVNCGDNLIGSQFFITLGEDTTCSL

DEHIVFGEIAEGHDVLLKLNETICDATHRPYQDIRITHTVILEDPYSDPDGLIVPCQSPE

PPLEVLQSDRIGADEDITEDIDIEELEEKRQEKEAQARATILEIVGDLPSADIAPPENVL

FVCKLNPVTSDDDLQIIFSRFGKIVSCEVIRDKKSGNSLQYAFVEFDNQKSCEDAYLKMD

NVLIDDRRIHVDFSQSVSKIKWLGKGRGVKYTDKDDEGKNISDKYSKYRNKKRDSYDSRN

NYIKNKISTHDSKRYRKDDKNYRYRESEKEERVPYTKYRRNEKDDNRRREDKTRTNFRNE

GERKTDRNERDGKNEKDDKNRRSNRDDRDKRSNRGDRTQRSDRHNRDSRR

>ctr27345_c1_g1_i1

DLLNSAHPKDKLSSFPKMLVHMRKTVSFFFFCTLALTLLAQTVQAINGPKITHKVYFDVT

HDDKPLGRIVMGLYGKTVPKTAENFRVLATGEKGEGYKYEGSTFHRVIQDFMIQGGDFTK

GDGTGGKSIYGDKFPDENFKLKHSKKGLLSMANAGPDTNGSQFFITTSTPSHLNGLHVVF

GEVLEGYDVVEKIQSVPKSYGDKPLKTVKIAKSGELPTTDEGTHAEL

>ctr27345_c3_g2_i22

MQLHFKTLKLLFLTSGRIVMGLFGNTVPKTVENFRALCTGEKGIGRSGKPLHYKGSTFHR

IIPSFMVQGGDFTRGDGRGGESIYGDKFADENFKLKHTGPGYLSMANSGQDTNGSQFFIT

TVKTSWLDGRHVVFGKVLSGMDVLYKIEAEGSESGSPKSKVVILDSGELTS

>ctr27355_c0_g1_i1

MWASAEGGAPEVTLETSMGAFTVELYYKHAPRTCRNFIELSRRGYYDNVKFHRIIKDFIV

QGGDPTGTGRGGESIYGAKFEDEIKRELKHTGAGILSMANAGANTNGSQFFITLAPCPSL

DGKHTIFGRVCSGMEIIKRLGSVQTDNSDRYVNYLST

>ctr27355_c0_g1_i10

MWASAEGGAPEVTLETSMGAFTVELYYKHAPRTCRNFIELSRRGYYDNVKFHRIIKDFIV

QGGDPTGTGRGGESIYGAKFEDEIKRELKHTGAGILSMANAGANTNGSQFFITLAPCPSL

DGKHTIFGRVCSGMEIIKRLGSVQTDNSDRPIHDVKILRTSVKD

>ctr27652_c1_g2_i7

MRRAIAFLVHPRFLILFLVLSIFIIFEFSGYKKVEEKIEEEPEVTHRVFLDVDIDKQRLG

RIVIGLYGQVVPKTVENFRALCTGEKGKSTEGLKLHYKGTPFHRIISGFMIQGGDIVHHD

GKASESIYGGTFPDENFKIKHSHAGVVSMANSGPDSNGSQFFITTVKASWLDGDHVVFGK

VIQGMDIVFAIEGGAGTYNGKPRKKVVIADCGEIPKSEWDEET

>ctr27784_c2_g1_i14

MARIKPQALLQQSKRKKGPSRISATTIIFYALILVLVAFFVFATYRHWSNRSSLQPETHL

SVSEGENAFVDSKKSELPGYAVLNTSKGSIIIELHKESAPEVVDEFIDLCQKGHFKGMLF

QRVIKHYVIQAGESHGTGAEDWNLRGKQHTSMKHEAFMLGTSKGKHVNKVFDLFITTAPI

PDLNEKLIVFGQVVKGEDVVQEIEEVDTDEHYKPKISIGILDVTLKQKI

>ctr27784_c2_g1_i20

MARIKPQALLQQSKRKKGPSRISATTIIFYALILVLVAFFVFATYRHWSNRSSLQPETHL

SVSEGENAFVDSKKSELPGYAVLNTSKGSIIIELHKESAPEVVDEFIDLCQKGHFKGMLF

QRVIKHYVIQAGESHGTGAEDWNLRGKQHTSMKHEAFMLGTSKGKHVNKVFDLFITTAPI

PDLNEKLIVFGQVVKGEDVVQVTESALFSLLCLHCGLWKFLFSLFFFPYFDLSIFPFNAN

FFLCWLLKIVCVKSISNKKTRVECVVQNNKGSIS

>ctr27784_c2_g1_i28

MGRIKPQALLQQSKRKKGPSRKSATAILFYAVILVIFALFLFATYRHWPGRTRFQQENYI

SVSEDEKAFVKSKKSDLPGYAVLTTSKGSIVVELYKESAPEVVDEFIDLCQKGHFKGMLF

QRVIKHYVIQAGESHGTGAEDWNLRGKQHTSMKHEAFMLGTSKGKHVNKVFDLFITTAPI

PDLNEKLIVFGQVVKGEDVVQEIEEVDTDEHYKPKISIGILDVTLKQKI

>ctr27856_c0_g2_i1

MSNPKVFFDILIGKMKARRVVMELFADVTPKTAENFRALCTGEKGIGTYGKPLHFKGSSF

HRIIPEFMCQGGDFTRGNGTGGESIYGSKFQDENFKLKHTGPGILSMANAGPNTNGSQFF

VCTTKTSWLDGKHVVFGKVIDGYSVVKEMEKVGSQSGKTLEPVVIEDCGQVVEN

>ctr27930_c3_g13_i1

MMDTKKDFEFEELIICGCCKQKFNETEFVPKELSCKHCFCLQCVKTTMLKGLEVYCINCW

KRTELDEQSPETLRTQKSILSLIRHLANVKVNKSVDKERKGENCHTHGMPFSFWCYNCQQ

LLCRACGTQSDHIGHSIKSNNDARDHLISEVQVETIAIAKLMSEIQNLFGHKRQFLLRVL

DACNTLKTQIELELNSGWTQNTNDLLQTNETLNSIKPTNLRTDDLYDLQLYLNRLEQVKQ

KVQNKYSEQFAHGQLEDIISSTNLLDFGMIKQSLSSLQSMALESYKSADLANPNTHIFFL

ANYCTAQLFTRYVLPKHVTQLVNGSEFAKNSPGNSSTTQYTTSLSSISSNEPVLITSQNS

PPPAILNNVPSIRNVNTYPMFYFNIEVNGTSFGRLVIETRPDVAPKMSKNFEVLTVGDTN

GCSYKGCSIFQCWDGESVITGDFELNNGRGGKSIYDESYFMPDDTKFPAVRGAVGMRRTQ

KRHDNMGMVGSQFRIILQEMRGFTAIFGHVVDGIDLVEKMASFGDQTGKPSKTFVISSCG

KV

>ctr28568_c0_g5_i4

MGRRNIEQNSTLSNRLILFVACFASCGVVYALLSAVLTGNRIRNSSVSEFGTLVERGGNG

LGSKSDGGCCRGIENLELWGSAVKWGSEFKFNSSEECCNACKSMCTGKDGPCLCDTWVFC

GNRQACGSKFGECWLKKQKDTLAPERQEGVPPGEIVGWTSGLIFGKGEGIIGLDTEYGTL

HIKLLPDCAPHSVAYILELLSLNHCAGCQFYRAESRGQSWDSEGNHVENAGFGPPYALIQ

GILEAQGTPFNKLPVEDCPLLRRGSVAWIGPGPEFFISLADHSEWKHEYTVFGSVLPEDL

NIAEKIATLPTIPDVWNNVNVSVLEKPVPLLLRRIQKSHLD

>ctr28930_c1_g3_i5

MSVTLHTNLGDIKCEIFCDEVPKASENFLALCASGYYDGTIFHRNIKGFMIQGGDPTGTG

KGGTSIWGKKFNDEIRESLKHNARGILSMANSGPNTNGSQFFITYAKQPHLNGLYTIFGR

VIHGFEVLDLMEKTQTGAGDRPLAEIRLNRVTIHANPLAG

>ctr28936_c0_g2_i2

MASTTFRLFTRRQLQYCSAPSHSYAGGLSSFLLYSTKPKAPTAKMSLPKVFFDMAADGEA

LGRITIELRSDVVPKTAENFRALCTGEKGFGYKGSIFHRVIPNFMCQGGDFTNHNGTGGK

SIYGNKFEDENFTLKHTGPGILSMANAGANTNGSQFFVTTVKTSWLDTKHVVFGAIVDGM

DVVKKIESYGTQSGKTTKKITVANCGQLS

>ctr28936_c0_g2_i3

MASTTFRLFTRRQLQYCSAPSHSYAGGLSSFLLYSTKSKASSTKMSLPKVFFDMSADGEQ

LGRITIELRSDVVPKTAENFRALCTGEKGFGYKGSIFHRVIPNFMCQGGDFTNHNGTGGK

SIYGNKFEDENFTLKHTGPGILSMANAGANTNGSQFFITTVKTSWLDTKHVVFGAIVDGM

DVVKKIESYGTQSGKTTKKITVANCGQLS

>ctr28936_c0_g3_i12

MASSLSTHLIQYHFQGNFSQGVSKVRRSHVVCSGPGSQFGYCKSLASRAHYAFRFPVTQK

SEARSTRYRRMSCVNAAADNVIELQAKVTTKCFFDVEVGGEPVGRIVLGLFGEVTPKTVE

NFRALCTGEKGYGYKGSSFHRIIKEFMIQGGDFTEGDGTGGISIYGPSFKDESFALKHIG

PGVLSMANAGPNTNGSQFFICTVKTPWLDNRHVVFGHVTEGMDVVKTLESQETSRLDTPR

KPCRIVNCGELPTDG

>ctr28936_c0_g4_i4

MAILLLFTKLVHCLTLFCYFQAVELQAKVTNKVFFDIQIEGEPTGRIVFGLFGDVVPKTV

ENFRALCTGEKGYGYKGSYFHRIIKDFMIQGGDFTEGNGTGGASIYGYKFEDESFSLKHV

GPGVLSMANAGPNTNGSQFFICTVKTPWLDNRHVVFGHVVDGFDVVKALESLEVSEYDKS

PRKACKIANSGELPLDS

>ctr28936_c0_g4_i6

MTCVNAQESAVELQAKVTNKVFFDIQIEGEPTGRIVFGLFGDVVPKTVENFRALCTGEKG

YGYKGSYFHRIIKDFMIQGGDFTEGNGTGGASIYGYKFEDESFSLKHVGPGVLSMANAGP

NTNGSQFFICTVKTPWLDNRHVVFGHVVDGFDVVKALESLEVSEYDKSPRKACKIANSGE

LPLDS

>ctr28936_c0_g7_i1

MANPKVFFDMTVDGQPAGRIVIELRKDVVPKTAENFRALCTGEKGYGYKGSVFHRVIPNF

MCQGGDFTNHNGTGGKSIYGAKFGDENFTLRHTGPGILSMANAGPNTNGSQFFITTVKTT

WLDGKHVVFGSVTEGMDVVKKLESYGTDTGETKKKVVVANCGEL

>ctr29069_c0_g1_i11

MSTVYVLEPPTKGKVVLNTTRGPLDIELWPKEAPKAVRNFVQLCLEGYYDNTIFHRIIKD

FLVQGGDPTGTGTGGESIYGAVFVDEFHSRLKFKHRGIVAMANAGTPNSNGSQFFITLDR

CDWLDRKHTIFGKVTGDTMYNLLRIGEVETDKDDRPLDPPPKILSIEVLWNPLEDIVPRT

LQKPHVEAKHDKESKELKKKGVKKLNLLSFGEEAEEEEKELALVKQKIKSSHDVLNDPRL

LREETSNNELSNTTRDLQLSVRDALNSKKEERQKDSEAGNMAHLDSSDDDDNEADFDARM

RMQILKKRKELGDHPPKPKLQNGRSSSENHDTSATR

>ctr29069_c0_g1_i13

MSTVYVLEPPTKGKVVLNTTRGPLDIELWPKEAPKAVRNFVQLCLEGYYDNTIFHRIIKD

FLVQGGDPTGTGTGGESIYGAVFADEFHSRLKFKHRGIVAMANAGTPNSNGSQFFITLDR

CDWLDRKHTIFGKVTGDTMYNLLRIGEVETDKDDRPLDPPPKILSIEVLWNPLEDIVPRT

LQKPHVEAKHDKESKELKKKGVKKLNLLSFGEEAEEEEKELALVKQKIKSSHDVLNDPRL

LREETSNNELSNTTRDLQLSVRDALNSKKEERQKDSEAGNMAHLDSSDDDDNEADFDARM

RMQILKKRKELGDHPPKPKLQNGRSSSENHDTSAARSNAVSVDEDQPKVEKLALKKKGVG

SEARAERIANADADLQLLNEAERGRQLQKQKKRRLQGREDEVLAKLEKFKSSLSTKATPT

AEDDGSAKEALSDWKDVTLKFAPEPGKDRMSRNEDPNDYVVHDPLLEKGKEKFNRMIAKQ

KRRGREWAGRSLT

>ctr29069_c0_g1_i2

MSTVYVLEPPTKGKVVLNTTRGPLDIELWPKEAPKAVRNFVQLCLEGYYDNTIFHRIIKD

FLVQGGDPTGTGTGGESIYGAVFADEFHSRLKFKHRGIVAMANAGTPNSNGSQFFITLDR

CDWLDRKHTIFGKVTGDTMYNLLRIGEVETDKDDRPLDPPPKILSIEVLWNPLEDIVPRT

LQKPHVEAKHDKESKELKKKGVKKLNLLSFGEEAEEEEKELALVKQKIKSSHDVLNDPRL

LREETSNNELVINTLL

>ctr29069_c0_g1_i3

MSTVYVLEPPTKGKVVLNTTRGPLDIELWPKEAPKAVRNFVQLCLEGYYDNTIFHRIIKD

FLVQGGDPTGTGTGGESIYGAVFADEFHSRLKFKHRGIVAMANAGTPNSNGSQFFITLDR

CDWLDRKHTIFGKVTGDTMYNLLRIGEVETDKDDRPLDPPPKILSIEVLWNPLEDIVPRT

LQKPHVEAKHDKESKELKKKGVKKLNLLSFGEEAEEEEKELALVKQKIKSSHDLLNDPRL

LREETSNNEPSNATRDLQLSVRDALNSKKEEHQKDSETGNLAHPDSSDDDDNEADFDARM

RMQILKKRKELGDLPSKPKLQNAGRSSSENHDTSATRSNAVSVDEDQPKVEKLALKKKGI

GSEARAERIANADADLQLLNDAERGRQLQKQKKRRLQGREDEVLAKLKKFKSSLSTKATP

TATAEDDGSAKEALSDWKDVTLKFAPEPGKDRMSRNEDPNDYVVHDPLLEKGKEKFNRMI

AKQKRQEREWAGRSLT

>ctr29069_c0_g1_i6

MSTVYVLEPPTKGKVVLNTTRGPLDIELWPKEAPKAVRNFVQLCLEGYYDNTIFHRIIKD

FLVQGGDPTGTGTGGESIYGAVFVDEFHSRLKFKHRGIVAMANAGSPNSNGSQFFITLDR

CDWLDRKHTIFGKVTGDTMYNLLRIGEVETNKDDRPLDPPPKILSIEVLWNPFEDIAPRT

LQKPQVEAKHDTESKELKKKGVKKLNLLSFGEEAEEEEKELALVKQKIKSSHDLLNDPRL

LREETSNNEPSNATRDLQLSVRDALNSKKEEHQKDSETGNLAHPDSSDDDDNEADFDARM

RMQILKKRKELGDLPSKPKLQNAGRSSSENHDTSATRSNAVSVDEDQPKVEKLALKKKGI

GSEARAERIANADADLQLLNDAERGRQLQKQKKRRLQGREDEVLAKLKKFKSSLSTKATP

TATAEDDGSAKEALSDWKDVTLKFAPEPGKDRMSRNEDPNDYVVHDPLLEKGKEKFNRMI

AKQKRQEREWAGRSLT

>ctr29069_c0_g1_i7

MSTVYVLEPPTKGKVVLNTTRGPLDIELWPKEAPKAVRNFVQLCLEGYYDNTIFHRIIKD

FLVQGGDPTGTGTGGESIYGAVFVDEFHSRLKFKHRGIVAMANAGSPNSNGSQFFITLDR

CDWLDRKHTIFGKVTGDTMYNLLRIGEVETNKDDRPLDPPPKILSIEVLWNPFEDIAPRT

LQKPQVEAKHDTESKELKKKGVKKLNLLSFGEEAEEEEKELALVKQKIKSSHDVLNDPRL

LREETSNNELSNTTRDLQLSVRDALNSKKEERQKDSEAGNMAHLDSSDDDDNEADFDARM

RMQILKKRKELGDHPPKPKLQNGRSSSENHDTSAARSNAVSVDEDQPKVEKLALKKKGVG

SEARAERIANADADLQLLNEAERGRQLQKQKKRRLQGREDEVLAKLEKFKSSLSTKATPT

AEDDGSAKEALSDWKDVTLKFAPEPGKDRMSRNEDPNDYVVHDPLLEKGKEKFNRMIAKQ

KRRGREWAGRSLT

>ctr29098_c0_g1_i3

MVGGFPYEVPEEYQNMPLLKGRAAVNMKVKVKDNPNFEECVFHIVLDGYNAPVTAGNFVD

LVERHFYDGMEIQRADGFVVQTGDPEGPAEGFIDPSTEKPRTVPLEIMVNGEKAPFYGAT

LEELGLYKAQTKLPFNAFGTMAMARDEFENNSGSSQVFWLLKESELTPSNANILDGRYAV

FGYVTENEDYLADLKVGDVIESIQVVSGLENLVNPSYKIAG

>ctr29582_c2_g1_i1

MAGSVGGSGGVEWHVRPPNPKNPIVFFDVTIGNIPAGRIKMELFADIAPKTAENFRQLCT

GEYRKAGLPVGYKGCQFHRVIKDFMIQAGDFVKGDGSGCVSIYGHKFDDENFTAKHTGPG

LLSMANSGPNTNGCQFFITCAKCDWLDNKHVVFGRVLGDGLLVVRKIENVATGPNNRPKL

PCVIAECGEM

>ctr29752_c0_g3_i1

MLQIPRVLRSSLQPFNLPTRTSLPSLSLFQLIPTSPSFPILKQQCRLSRRELTIFSNSCF

LLLLGAQPVYGSEAKAEESVANTSSIDQPQESVAKTSSSDQLQESIANTSSSDQPEENIV

NTSSSDEPQENLTTTPSCTERKPTKQVFLDISIDDEPAGRITVGLYGDDVPAGVDRFSKI

VSGAAGISYRRKEFVKIMPNYVQHRGLRSYGVDAELAKRTGSNFGADSLVEEWERIYEGC

PGTKNVAGSIGIIVRDPSKPPPKLKLVARRGKLEIDQEEVGTDPNGTEFVIVSKDSPELD

ASTLVIGRVVGGLEVVQRISQVKTVQENTSSPYFRVAKLIGDKRAVGAERGFNRPYSKVI

VTNCGLMK

>ctr30170_c0_g1_i12

KMSVLIVTSLGDLVIDLNTNKCPLTCKNFLKLCKIKYYNGCLFHTVQKDFTAQTGDPTGT

GTGGDSAYKFLYGEQARFFSDEIHIDLKHSKTGTVAMASAGENLNASQFYITLRDDLDYL

DGKHTVFGEVAEGFDTLTRINEAYVDEKGRPYKNIRIKHTYILEDPYDDPPQLAEFIPDA

SPEGKPKDEVDDEVRLEDDWVPMDEQLNPAELEEVIRAKEAHSRAVVLESIGDIPDAEIK

PPDNVLFVCKLNPVTEDEDLHTIFSRFGTVSSAEIIRDHKTGDSLCYAFIEFEDRQACEQ

AYFKMDNALIDDRRIHVDFSQSVAKLWSQYKRKGQKGKGGGCFKCGSTDHIAKDCNADGT

MKQPAKYILKDDNAQRGGDNARSVLWIACFCLRPSL

>ctr30170_c0_g1_i15

KMSVLIVTSLGDLVIDLNTNKCPLTCKNFLKLCKIKYYNGCLFHTVQKDFTAQTGDPTGT

GTGGDSAYKFLYGEQARFFSDEIHIDLKHSKTGTVAMASAGENLNASQFYITLRDDLDYL

DGKHTVFGEVAEGFDTLTRINEAYVDEKGRPYKNIRIKHTYILEDPYDDPPQLAEFIPDA

SPEGKPKDEVDDEVRLEDDWVPMDEQLNPAELEEVIRAKEAHSRAVVLESIGDIPDAEIK

PPDNVLFVCKLNPVTEDEDLHTIFSRFGTVSSAEIIRDHKTGDSLCYAFIEFEDRQACEQ

AYFKMDNALIDDRRIHVDFSQSVAKLWSQYKRKGQKGKGGGCFKCGSTDHIAKDCTADGT

MKQPAKYILKDDNAQRGGDNARYEMVFDGDNSESPKQDVKHRRHDSDERRGQKESSKEHR

NLRDQEMGGSNNRDRYGDRRGHWGNGVDKTRFERDVRDTRNLDPHADRKDRDRPMGRYRD

DDHRRKDDRYSRRDSDDSYLERRASRDDRRIMGSSHLERRDDRDCRKRPEDSRRQDVKVD

FGRRKRSPDDEEHKRTREAEDYKLRKRSPDDDGEYKSRREERGHRNRRHDTESDDYPHRR

HHGDRR

>ctr30170_c0_g1_i9

KMSVLIVTSLGDLVIDLNTNKCPLTCKNFLKLCKIKYYNGCLFHTVQKDFTAQTGDPTGT

GTGGDSAYKFLYGEQARFFSDEIHIDLKHSKTGTVAMASAGENLNASQFYITLRDDLDYL

DGKHTVFGEVAEGFDTLTRINEAYVDEKGRPYKNIRIKHTYILEDPYDDPPQLAEFIPDA

SPEGKPKDEVDDEVRLEDDWVPMDEQLNPAELEEVIRAKEAHSRAVVLESIGDIPDAEIK

PPDNVLFVCKLNPVTEDEDLHTIFSRFGTVSSAEIIRDHKTGDSLCYAFIEFEDRQACEQ

AYFKVCWGFKASFCK

>ctr30311_c0_g1_i1

MTVAGTPAGRIVMELFADTTPKTAENFRALCTGEKGVGRSGKPLHFKGSTFHRVIPNFMC

QGGDFTAGNGTGGESIYGAKFEDENFVKKHTGPGVLSMANAGPGTNGSQFFICTVKTEWL

DGKHVVFGQVVEGMDVVKEIEKVGSSSGKTSRPVLIADCGQLS

>ctr31571_c0_g1_i1

MNNSSVIAGNSVVFLDIEIAQEKIGRIVIELFNNVVPKTAENFRALCTGEKGIGLSGKPL

HLKGSSFHRAVPEFMIQGGDITAGNGSGGESIYGLFFEDENFELLHEEPGVLSMANTGHK

NTNNSQFFITTAPCSHLDGKNVVFGKVKKGFCVVQTISATTTNNDIPINPCIITDCGELS

SDSNTWNINENDTTNDVFPPFPEDWDIEPIDLDVIQICDVLNKIKESGNQFFSCNNYSCA

RRKYDKVLRYFEWYKSYHKNSKMDLNMLETIQTNTLLNLSTVHLKEGNYKISIELSEQVL

NIDCNNGKALFRLGKAYGSLNNYEKAIKYYKKALDIFPDEKNILIELKKVIQAQNQYLAT

>ctr44502_c0_g1_i1

TNKCIKVMGKPENIRPMKLALFQGKAKKSTAATTVEIEGSNNPTLEMIKPDPTLFCTAHK

KNRFFIFTRREPEDTKNQECDRDVFNEKPSKEDIISSTEATSMQKIYDTAIIHTALGDIH

VSLFSKDTPKTVENFCVHAKNNYYNSHIFHRIIKGFMVQTGDPTGTGTGGESIWGGEFND

EFKSHLKHDRPYTVSMANAGPNTNGSQFFITLTPTPWLDNKHTVFGRVVKGMEVVQNISQ

VKTNPKTDKPYDDIRIV

>ctr68387_c0_g1_i1

MSVTLHTDVGDIKIEVFCEECPKTAENFLALCASDYYNGCLFHRNIKGFIVQTGDPTHTG

KGGSSIWGRKFEDEFKENLKHKERGTLSMANNGPNTNSSQFFLTYAAQPNLDLKYTVFGR

VIDGFEALDELEKLPVNSKNFKPLTDVKIQYVSIHANPLAT

>ctr79517_c0_g1_i1

MSVTLHTDVGDMKVELFCEACPKACENFLALCASDYYNGSLFHRNIKGFIVQTGDPTHTG

KGGTSIWGRKFEDEFKDNLKHNVRGIISMANNGPNTNASQFFITYGPQPHLDLKYTIFGK

VIDGFEALEELEKLPVNPKNYKPLTETRLQSVTIHANPMAG

>ctr8964_c0_g1_i1

MRAFVILAIGLAVVCLGSAEKTDLQITDVVWFDIKLGDEKLERIEIGLFGKTVPRTVRNF

AELAKKKEGEGYKGSKFHRVIKDFMLQGGDFTRGDGTGGRSIYGEKFADENFKLKHYGAG

WVSMANAGKDTNGSQFFITTKQTPWLDRRHVVFGKVIKGMKSVRTIESTKTDSRDKPASD

VVIVDCGHKKLADTEQFITTPEPSEE
