# Supplementary material for: Transcriptomic profiling of the medicinal plant Clitoria ternatea: identification of potential genes in cyclotide biosynthesis
Source: Sci Rep. 2020 Jul 29;10:12658. doi: 10.1038/s41598-020-69452-7 (PMC7391643; doi:10.1038/s41598-020-69452-7)
Supplement: Supplementary file 2 — Supplementary Information [file 41598_2020_69452_MOESM2_ESM.pdf]

Supplementary Information:

Transcriptomic Profiling of the Medicinal Plant *Clitoria ternatea*: Identification of Potential Genes in Cyclotide Biosynthesis

Neha V. Kalmankar[1,2], Radhika Venkatesan[1], Padmanabhan Balaram[1,3], Ramanathan Sowdhamini[1*]

[1]National Centre for Biological Sciences (TIFR), GKVK Campus, Bangalore 560065, Karnataka, India.

[2]The University of Trans-Disciplinary Health Sciences and Technology (TDU), #74/2, Jarakabande Kaval, Post Attur, Via Yelahanka, Bangalore 560064, Karnataka, India

[3]Molecular Biophysics Unit, Indian Institute of Science, Bangalore 560012, Karnataka, India

*** Correspondence:**
Prof. Ramanathan Sowdhamini
mini@ncbs.res.in

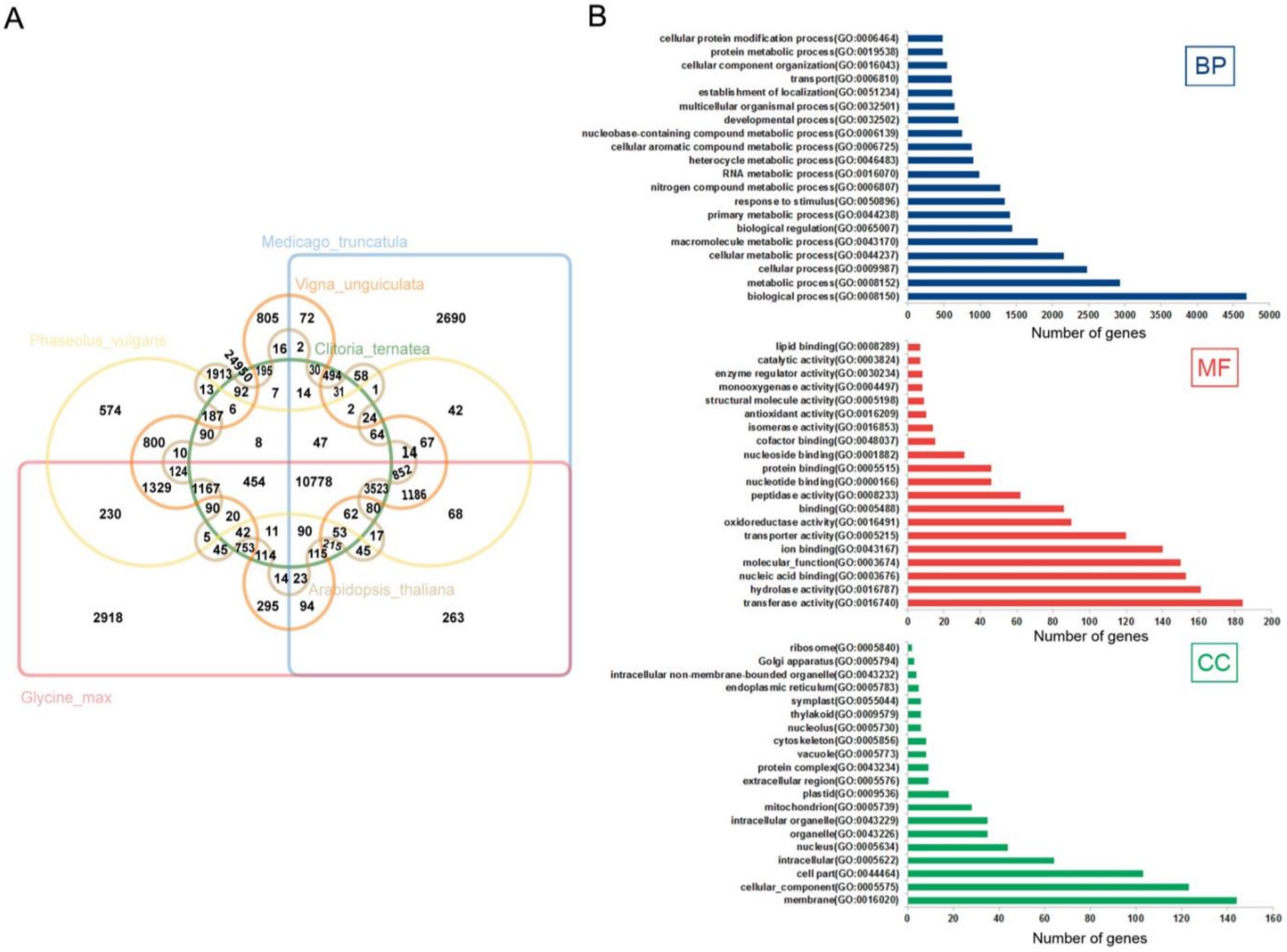

Supplementary Figure S1: Orthology analysis of *Clitoria ternatea* predicted proteome with other plant proteomes using OrthoVenn [52]. **(A)** Venn diagram showing shared and distinct protein clusters (orthogroups) between *C. ternatea* (green), *A. thaliana* (brown), *M. truncatula* (blue), *G. max* (red), *V. unguiculata* (orange) and *P. vulgaris* (yellow). **(B)** Horizontal bar chart depicting top 20 Gene Ontology (GO) annotation categories of *C. ternatea*-specific proteins. GO categories included BP = biological process, MF = molecular function, CC = cellular component. In *C. ternatea*, the highly represented GO categories of biological process were those of cellular process, macromolecular metabolic process, response to stimulus, etc. The highly represented GO categories of molecular function were those of transferase activity, nucleic acid binding, oxidoreductase activity, etc.

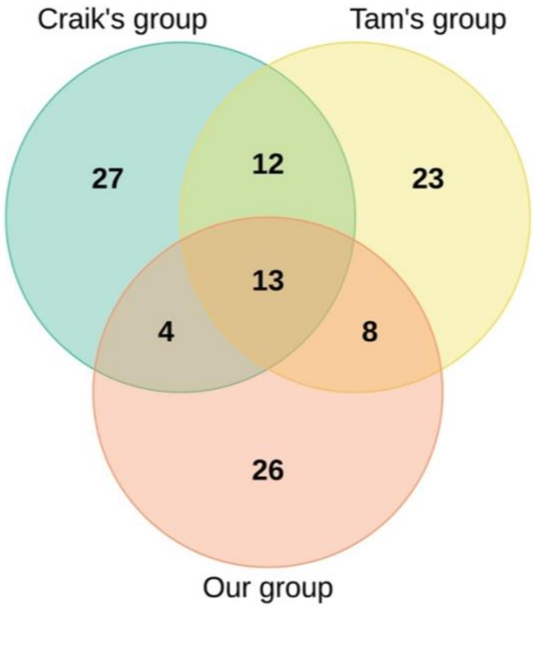

Supplementary Figure S2: Comparative Venn diagram of the number of cyclotides identified from *Clitoria ternatea* by Prof. David Craik's group, Prof. James Tam's group and our group (current study).

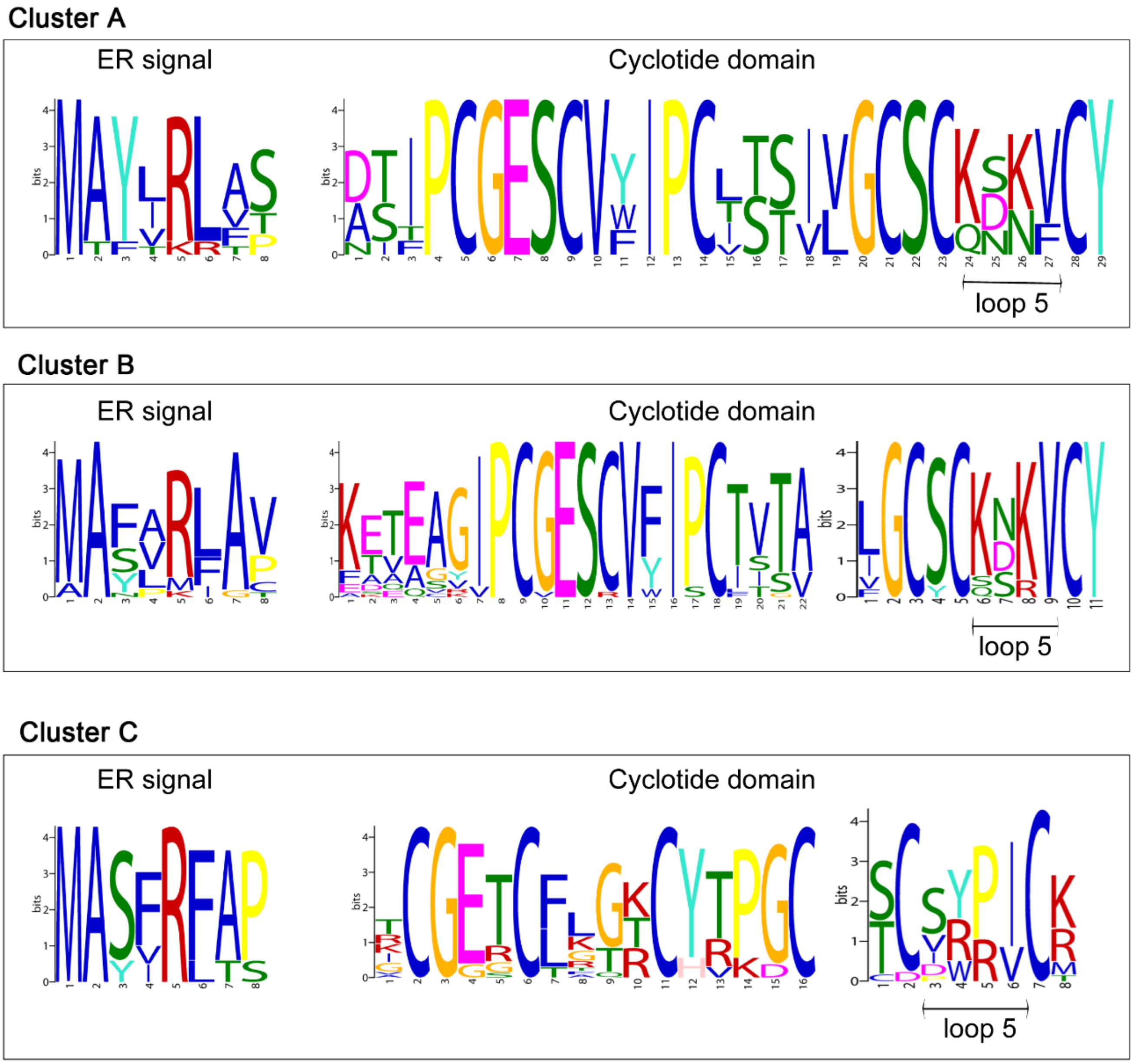

Supplementary Figure S3: MEME logos predicted for the cyclotide precursors distributed across three structural clades (A-C) in the cyclotide phylogeny (main text, Fig. 3) using MEME Suite [62]. (**A)** MEME logo for ER-signal peptide, cyclotide domain and loop 5 in cluster A (**B)** MEME logo for ER-signal peptide, cyclotide domain and loop 5 in cluster B **(C)** MEME logo for ER-signal peptide, cyclotide domain and loop 5 in cluster C.

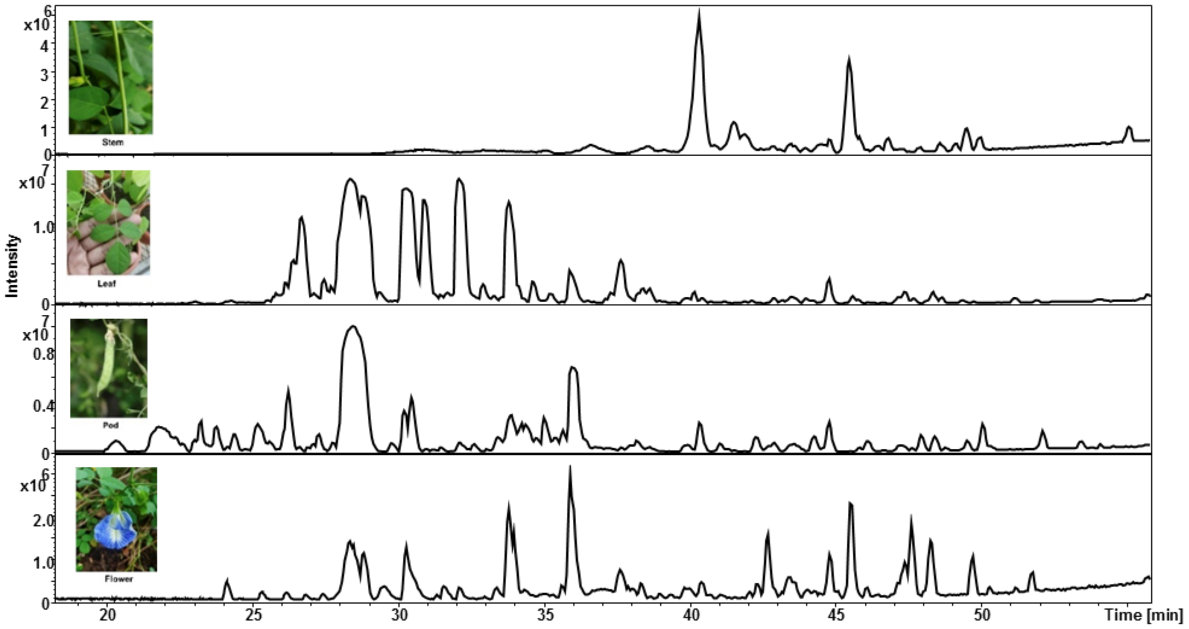

Supplementary Figure S4: Mass detected analytical LC ESI-MS profile (ion chromatogram) of the stems, leaves, pods and flower crude extract from *C. ternatea*. Masses corresponding to cyclotides (>3000 Da) were detected in the retention time range 25-55 min. Further fractionation of these extracts using semi-preparative HPLC is illustrated in Supplementary Fig. S5.

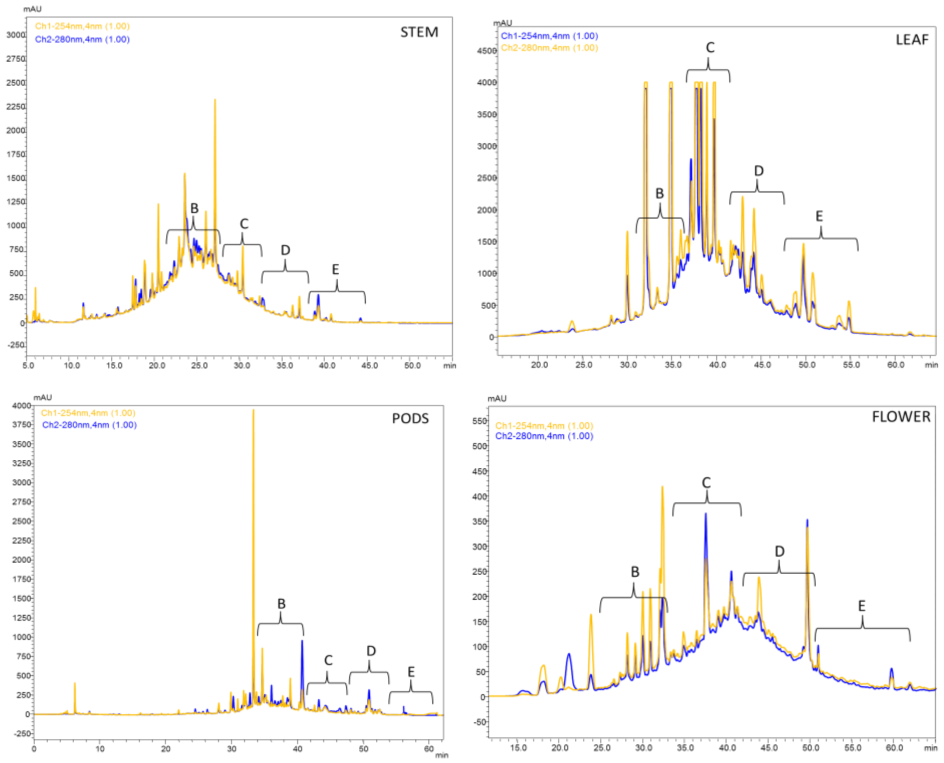

Supplementary Figure S5: Semi-prep HPLC profiles of cyclotide extracts obtained from leaf, pod, stem and flower tissues of *Clitoria ternatea*. Late-eluting peaks were separated into five fractions (A-E). Cyclotides were distributed in fractions B-E and these are labeled. For cyclotide transcript and peptide IDs, and molecular weight (monoisotopic masses) refer to Supplementary Table S6.

A

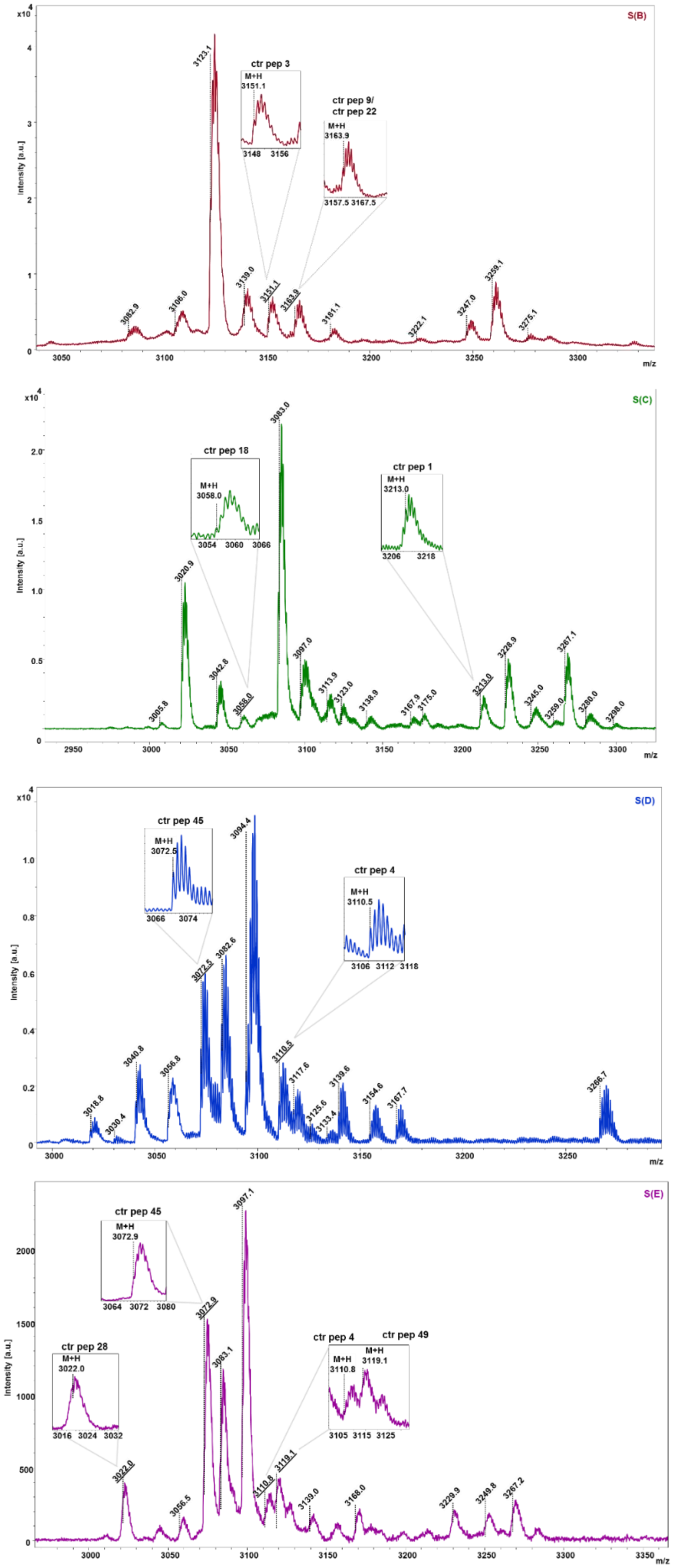

Supplementary Figure S6: MALDI-TOF mass-spectra of HPLC-purified cyclotide-rich fractions (B-E) from C. ternatea (A) stem, (B) leaf, (C) pod and (D) flower tissues. (Insets) The expanded isotopic multiplets for M+H masses that correspond to predicted cyclotide transcripts (peptide identifiers are marked) are shown.

B

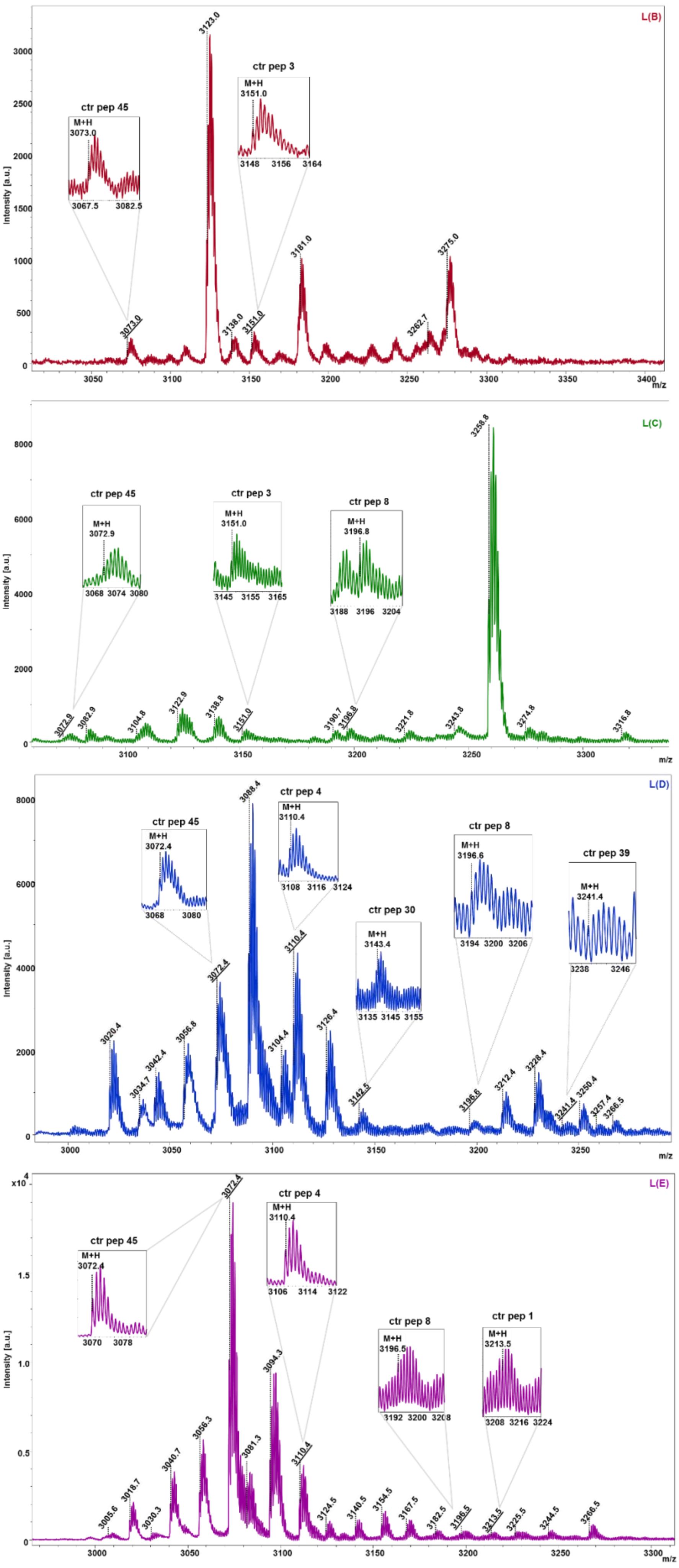
L(B)
ctr pep 45
M+H
3073.0
3067.5
3082.5
ctr pep 3
M+H
3151.0
3148
3156
3164
3123.0
3181.0
3275.0
3073.0
3138.0
3151.0
3262.7
Intensity [a.u.]
m/z
L(C)
ctr pep 45
M+H
3072.9
3068
3074
3080
ctr pep 3
M+H
3151.0
3145
3155
3165
ctr pep 8
M+H
3196.8
3188
3196
3204
3258.8
3072.9
3082.9
3104.8
3122.9
3138.8
3151.0
3190.7
3196.8
3221.8
3243.8
3274.8
3316.8
L(D)
ctr pep 45
M+H
3072.4
3068
3080
ctr pep 4
M+H
3110.4
3108
3116
3124
ctr pep 30
M+H
3143.4
3135
3145
3155
ctr pep 8
M+H
3196.6
3194
3200
3206
ctr pep 39
M+H
3241.4
3238
3246
3088.4
3072.4
3110.4
3020.4
3034.7
3042.4
3056.8
3104.4
3126.4
3142.5
3196.6
3212.4
3228.4
3241.4
3250.4
3257.4
3266.5
L(E)
x10^4
ctr pep 45
M+H
3072.4
3070
3078
ctr pep 4
M+H
3110.4
3106
3114
3122
ctr pep 8
M+H
3196.5
3192
3200
3208
ctr pep 1
M+H
3213.5
3208
3216
3224
3072.4
3094.3
3005.6
3018.7
3030.3
3040.7
3056.3
3081.3
3110.4
3124.5
3140.5
3154.5
3167.5
3182.5
3196.5
3213.5
3225.5
3244.5
3266.5

C

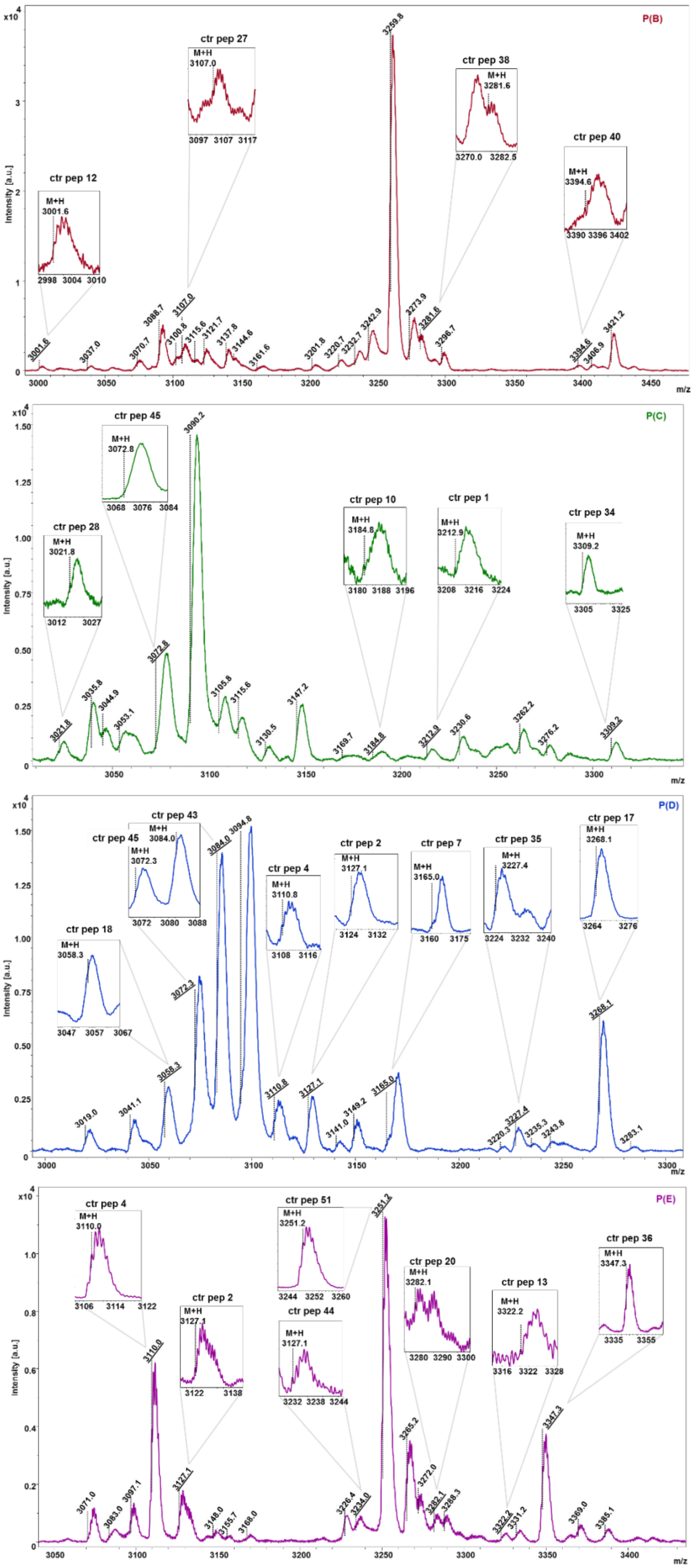
P(B)
Intensity [a.u.]
x10^4
m/z
ctr pep 12
M+H 3001.6
ctr pep 27
M+H 3107.0
ctr pep 38
M+H 3281.6
ctr pep 40
M+H 3394.6
3259.8
3421.2
P(C)
ctr pep 45
M+H 3072.8
ctr pep 28
M+H 3021.8
ctr pep 10
M+H 3184.8
ctr pep 1
M+H 3212.9
ctr pep 34
M+H 3309.2
3090.2
P(D)
ctr pep 43
M+H 3084.0
ctr pep 45
M+H 3072.3
ctr pep 18
M+H 3058.3
ctr pep 4
M+H 3110.8
ctr pep 2
M+H 3127.1
ctr pep 7
M+H 3165.0
ctr pep 35
M+H 3227.4
ctr pep 17
M+H 3268.1
3094.8
P(E)
ctr pep 4
M+H 3110.0
ctr pep 2
M+H 3127.1
ctr pep 51
M+H 3251.2
ctr pep 44
M+H 3127.1
ctr pep 20
M+H 3282.1
ctr pep 13
M+H 3322.2
ctr pep 36
M+H 3347.3

D

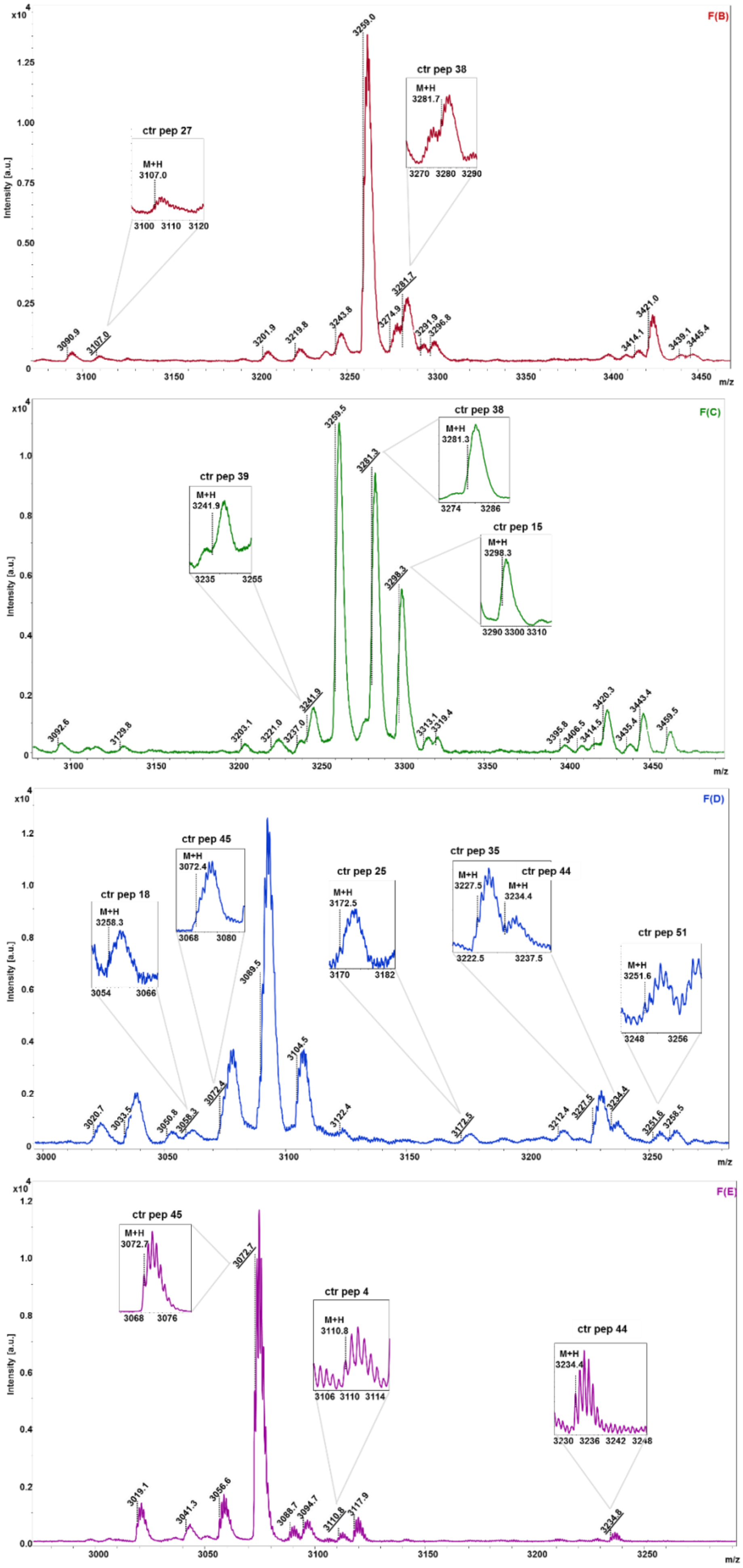

F(B)
x10^4
Intensity [a.u.]
m/z
ctr pep 27
M+H
3107.0
ctr pep 38
M+H
3281.7
3090.9
3107.0
3201.9
3219.8
3243.8
3259.0
3274.9
3281.7
3291.9
3296.8
3414.1
3421.0
3439.1
3445.4
F(C)
ctr pep 39
M+H
3241.9
ctr pep 38
M+H
3281.3
ctr pep 15
M+H
3298.3
3092.6
3129.8
3203.1
3221.0
3237.0
3241.9
3259.5
3281.3
3298.3
3313.1
3319.4
3395.8
3406.5
3414.5
3420.3
3435.4
3443.4
3459.5
F(D)
ctr pep 18
M+H
3258.3
ctr pep 45
M+H
3072.4
ctr pep 25
M+H
3172.5
ctr pep 35
M+H
3227.5
ctr pep 44
M+H
3234.4
ctr pep 51
M+H
3251.6
3020.7
3033.5
3050.8
3058.3
3072.4
3089.5
3104.5
3122.4
3172.5
3212.4
3227.5
3234.4
3251.6
3258.5
F(E)
ctr pep 45
M+H
3072.7
ctr pep 4
M+H
3110.8
ctr pep 44
M+H
3234.4
3019.1
3041.3
3056.6
3072.7
3088.7
3094.7
3110.8
3117.9
3234.8

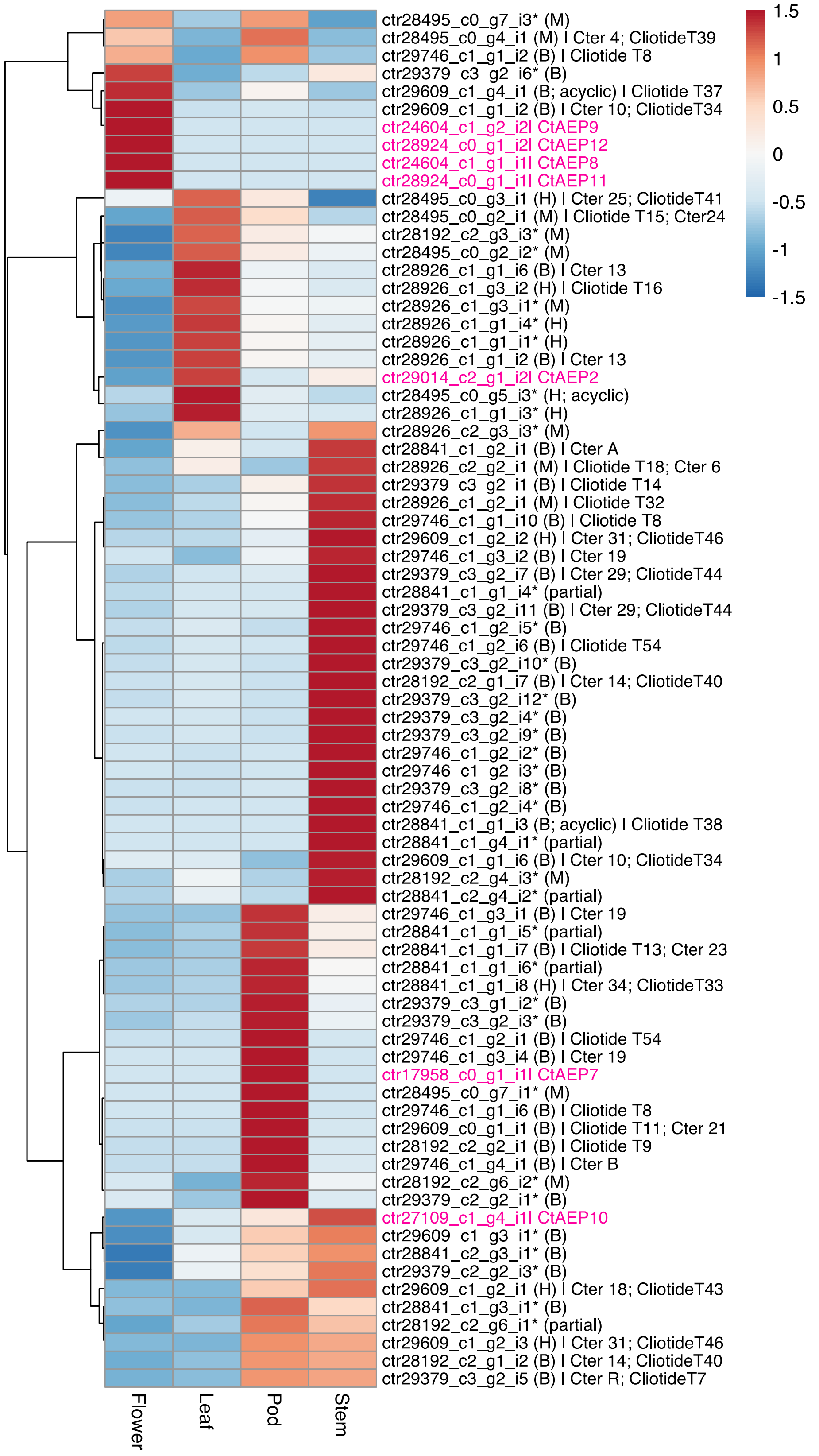

Supplementary Figure S7: Heatmap of differential expression of asparaginyl endopeptidase genes, (highlighted in pink font) across four tissues of *C. ternatea de novo* transcriptome. The heatmap was generated using average TPM values along with Pearson correlation coefficient clustering method and complete linkage on genes. Positive (red) and negative (blue) values correspond to up-and down-regulated clusters respectively.

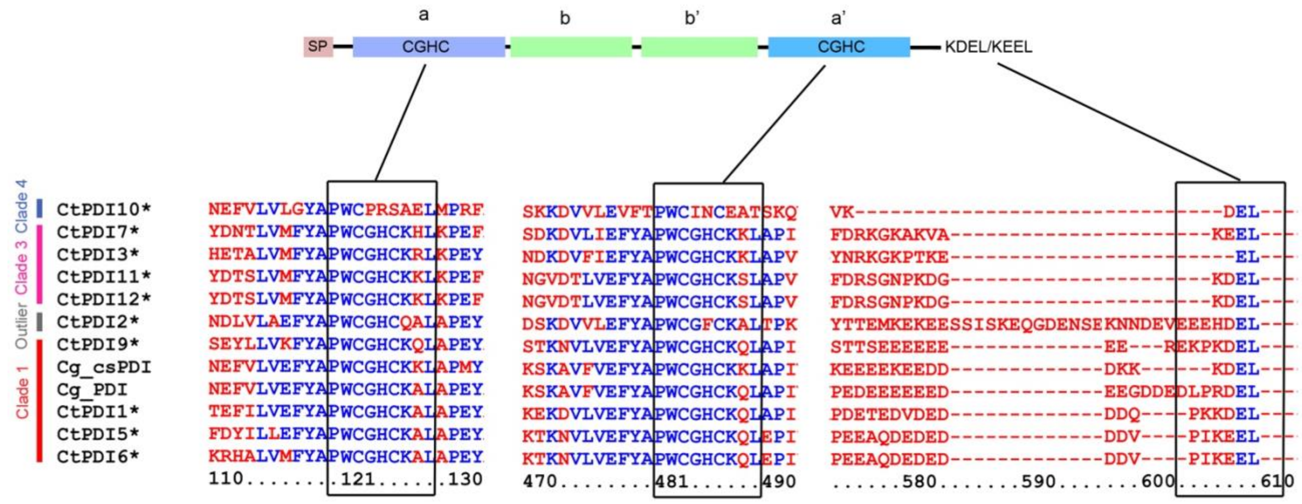

Supplementary Figure S8: Multiple sequence alignment of protein disulphide isomerase (PDIs) transcripts from *C. ternatea* transcriptome, *Conus geographus* conotoxin-specific PDI (Cg_csPDI; GenBank accession no. KT874567) and *Conus geographus* canonical PDI (Cg_PDI; GenBank accession no. KT874559). Domain architecture of canonical PDI sequences is displayed on top. **a-** and **a'-** domains containing the active site "CXXC" motif, and the C-terminal ER-retention signal is highlighted.

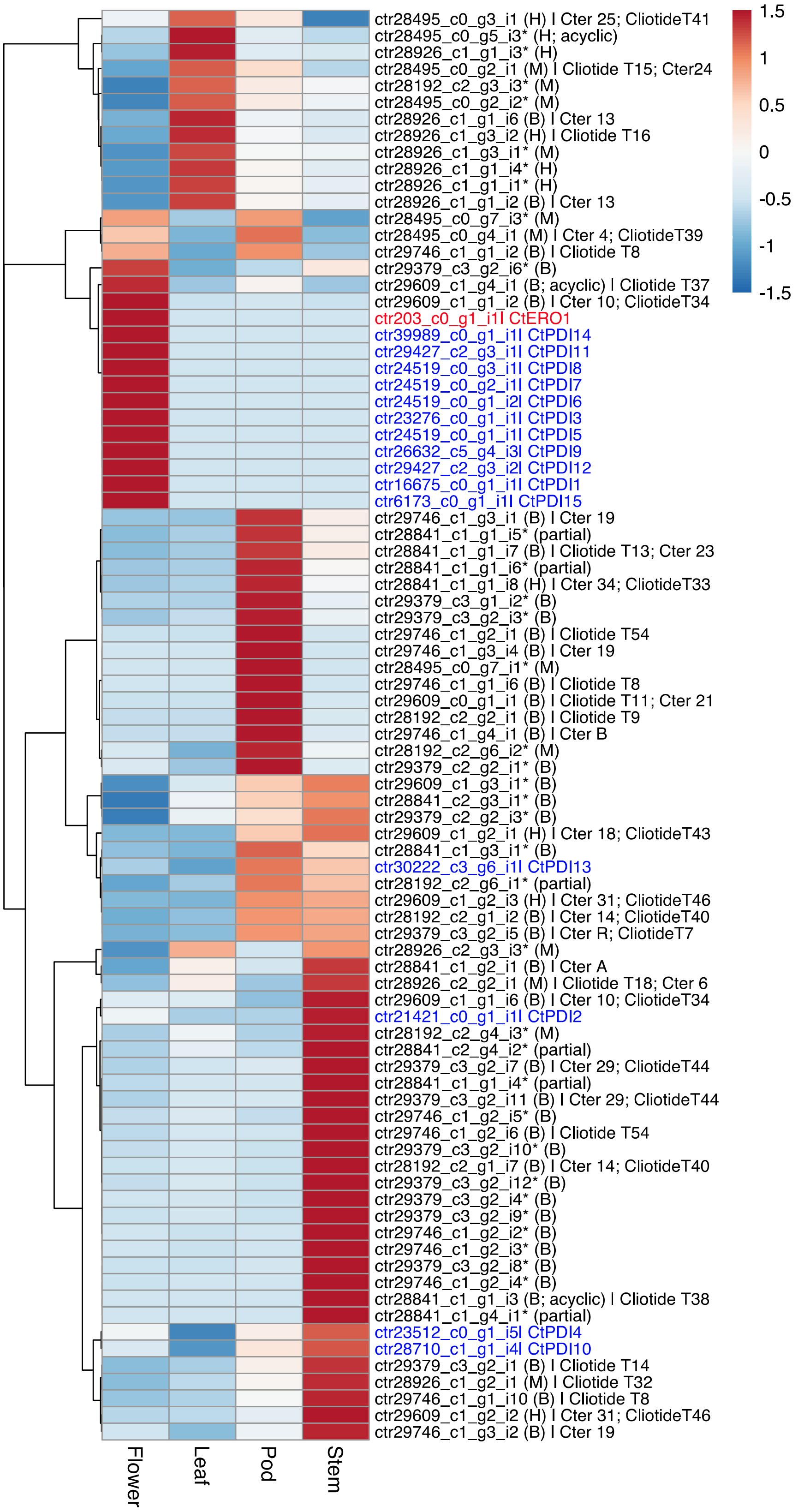

Supplementary Figure S9: Heatmap of differential expression of protein disulphide genes (highlighted in blue font) and ER-oxidoreductin 1 gene (highlighted in red font) across four tissues of *C. ternatea de novo* transcriptome. The heatmap was generated using average TPM values along with Pearson correlation coefficient clustering method and complete linkage on genes. Positive (red) and negative (blue) values correspond to up-and down-regulated clusters respectively.

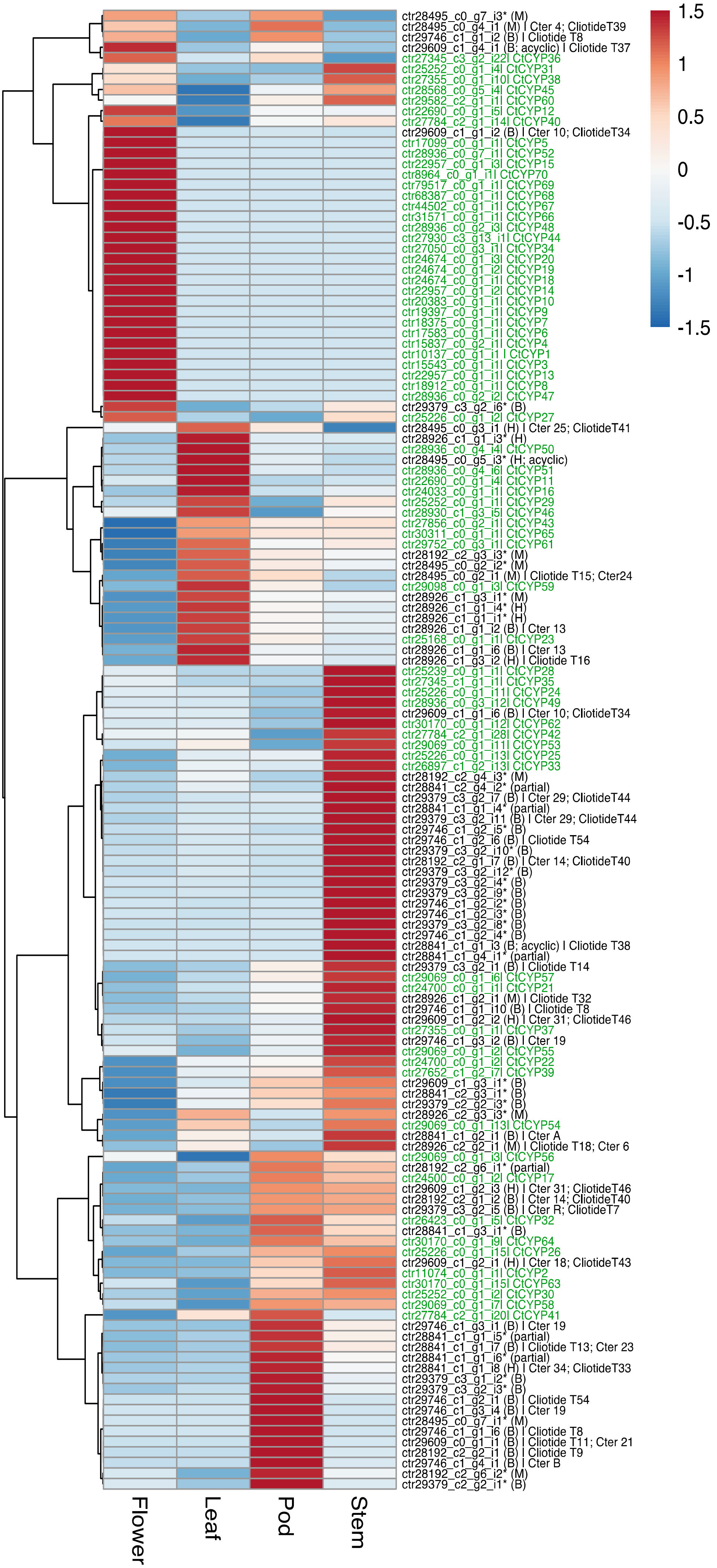

Supplementary Figure S10: Heatmap of differential expression of peptidyl prolyl *cis-trans* isomerase (PPIase) across four tissues of *C. ternatea de novo* transcriptome. The heatmap was generated using average TPM values along with Pearson correlation coefficient clustering method and complete linkage on genes. Positive (red) and negative (blue) values correspond to up-and down-regulated clusters respectively.

Supplementary Table S1: Raw reads summary

Sl. No	Sample	Read orientation	Mean read quality (Phred score)	Number of reads	% GC	% Q < 10	% Q 10–20	% Q 20–30	% Q > 30	Number of bases (MB)	Mean read length (bp)
1	C.ternatea-Leaf-1-A	R1	33.51	16868898	44.42	0.02	8.87	8.35	82.76	1686.89	100
		R2	34.68	16868898	44.63	0.32	5.38	5.09	89.21	1686.89	100
2	C.ternatea-Leaf-1-B	R1	33.62	15579380	46.85	0.02	8.49	8.11	83.38	1557.94	100
		R2	34.70	15579380	46.91	0.32	5.23	5.09	89.36	1557.94	100
3	C.ternatea-Leaf-2-A	R1	34.04	19273240	44.64	0.02	7.28	7.10	85.60	1927.32	100
		R2	34.62	19273240	44.74	0.32	5.51	5.24	88.92	1927.32	100
4	C.ternatea-Leaf-2-B	R1	33.88	17517155	44.94	0.02	7.76	7.46	84.76	1751.72	100
		R2	34.65	17517155	45.09	0.32	5.43	5.16	89.09	1751.72	100
5	C.ternatea-Flower-1-A	R1	34.09	17695012	42.66	0.02	7.21	6.99	85.78	1769.50	100
		R2	34.77	17695012	42.54	0.30	5.17	4.89	89.64	1769.50	100
6	C.ternatea-Flower-1-B	R1	34.01	16868324	43.82	0.02	7.38	7.23	85.37	1686.83	100
		R2	34.89	16868324	43.57	0.30	4.71	4.68	90.31	1686.83	100
7	C.ternatea-Flower-2-A	R1	33.74	23074480	43.62	0.02	8.26	7.80	83.92	2307.45	100
		R2	35.08	23074480	43.52	0.30	4.22	4.16	91.32	2307.45	100
8	C.ternatea-Flower-2-B	R1	33.66	18826377	43.53	0.02	8.52	7.97	83.48	1882.64	100
		R2	35.01	18826377	43.45	0.30	4.43	4.34	90.93	1882.64	100
9	C.ternatea-Pod-1-A	R1	33.64	18422283	43.81	0.02	8.55	8.04	83.38	1842.23	100
		R2	35.14	18422283	43.91	0.30	4.02	4.08	91.61	1842.23	100
10	C.ternatea-Pod-1-B	R1	33.85	17781619	43.47	0.02	7.91	7.55	84.52	1778.16	100
		R2	34.92	17781619	43.70	0.30	4.64	4.58	90.48	1778.16	100
11	C.ternatea-Pod-2-A	R1	34.08	21853026	44.44	0.02	7.17	7.02	85.79	2185.30	100
		R2	34.59	21853026	44.52	0.32	5.59	5.32	88.77	2185.30	100
12	C.ternatea-Pod-2-B	R1	33.56	19212475	44.40	0.02	8.71	8.21	83.06	1921.25	100
		R2	34.69	19212475	44.54	0.32	5.31	5.08	89.29	1921.25	100
13	C.ternatea-Stem-1-A	R1	33.78	21671955	43.10	0.02	8.15	7.68	84.15	2167.20	100
		R2	34.91	21671955	43.11	0.30	4.71	4.58	90.41	2167.20	100
14	C.ternatea-Stem-1-B	R1	33.41	16434353	43.22	0.02	9.22	8.61	82.15	1643.44	100
		R2	34.90	16434353	43.29	0.30	4.74	4.62	90.34	1643.44	100
15	C.ternatea-Stem-2-A	R1	33.76	17054426	43.45	0.02	8.21	7.73	84.03	1705.44	100
		R2	34.83	17054426	43.41	0.30	4.94	4.76	90.00	1705.44	100
16	C.ternatea-Stem-2-B	R1	33.95	14732524	43.43	0.02	7.64	7.30	85.04	1473.25	100
		R2	34.92	14732524	43.37	0.30	4.68	4.58	90.44	1473.25	100

Supplementary Table S2: **TPM values across biological replicates of all the transcripts discussed in this work.**

(a) Cyclotide precursor genes								
	TPM Values a							
**Transcript_ID**	**Flower_1**	**Flower_2**	**Leaf_1**	**Leaf_2**	**Pod_1**	**Pod_2**	**Stem_1**	**Stem_2**
ctr28192_c2_g1_i2	0.15	0.99	2.25	2.08	40.06	4.84	24.53	17.51
ctr28192_c2_g1_i7	1.43	1.74	7.00	12.52	2.78	0.66	159.18	123.67
ctr28192_c2_g2_i1	0.30	0.10	0.25	0.00	22.65	0.53	1.07	0.97
ctr28192_c2_g3_i3	63.79	116.87	14276.88	28807.20	12415.99	13892.23	11362.00	10765.10
ctr28192_c2_g4_i3	121.51	220.90	870.84	1119.08	131.09	271.43	3333.16	3225.11
ctr28192_c2_g6_i1	713.84	753.83	491.17	1936.65	3590.99	4684.75	3270.64	3624.38
ctr28192_c2_g6_i2	2.07	5.47	0.00	0.57	20.54	11.64	6.34	4.93
ctr28495_c0_g2_i1	114.66	96.79	1125.24	2203.40	1139.05	1097.28	437.42	349.29
ctr28495_c0_g2_i2	18.02	31.49	1331.48	2848.55	1252.17	1250.82	1056.21	908.77
ctr28495_c0_g3_i1	0.00	0.51	0.66	0.24	0.52	0.10	0.00	0.16
ctr28495_c0_g4_i1	17.17	26.48	0.00	0.00	49.41	8.03	1.37	0.00
ctr28495_c0_g5_i3	0.26	1.06	35.22	61.27	6.27	10.18	2.18	1.60
ctr28495_c0_g7_i1	0.00	0.00	0.00	0.00	404.51	0.00	0.00	0.00
ctr28495_c0_g7_i3	19707.33	24483.00	5966.39	8060.99	21976.51	22768.29	2822.98	4884.34
ctr28841_c1_g1_i3	0.16	0.00	0.52	2.44	1.77	0.85	4702.12	4642.01
ctr28841_c1_g1_i4	0.00	1.04	3.09	0.45	2.75	1.36	32.03	23.04
ctr28841_c1_g1_i5	2.81	3.23	15.57	4.53	115.08	91.97	41.25	52.81
ctr28841_c1_g1_i6	0.00	0.20	0.55	0.00	6.08	4.33	1.90	1.86
ctr28841_c1_g1_i7	0.04	0.56	32.18	1.73	316.81	389.80	176.13	156.18
ctr28841_c1_g1_i8	35.85	50.21	64.44	77.43	511.29	637.77	216.41	206.34
ctr28841_c1_g2_i1	954.63	1697.22	1805.76	5792.96	3858.11	1108.97	7744.54	5133.19
ctr28841_c1_g3_i1	0.37	1.77	0.00	0.00	34.18	8.52	14.63	14.14
ctr28841_c1_g4_i1	0.00	0.00	0.00	2.48	2.39	1.18	1780.64	1597.27
ctr28841_c2_g3_i1	174.77	207.17	494.23	714.35	1276.97	409.45	989.62	959.27
ctr28841_c2_g4_i2	17.70	55.80	657.34	766.34	63.69	248.89	3625.87	3362.48
ctr28926_c1_g1_i1	4.34	11.84	1995.71	3296.19	1509.06	1037.23	1106.65	818.95
ctr28926_c1_g1_i2	5.95	7.86	1636.11	3471.18	1491.08	949.49	1139.77	782.01
ctr28926_c1_g1_i3	0.97	1.36	92.17	211.71	24.68	40.67	28.52	25.30
ctr28926_c1_g1_i4	0.46	1.78	386.32	623.61	276.08	194.26	189.91	150.18
ctr28926_c1_g1_i6	0.00	0.00	93.82	181.43	62.20	32.33	41.04	24.44
ctr28926_c1_g2_i1	926.54	1176.53	1234.29	2029.37	4573.74	1627.36	6406.79	6228.02
ctr28926_c1_g3_i1	0.00	4.95	604.35	1014.65	465.62	291.31	395.62	303.25
ctr28926_c1_g3_i2	18.62	49.18	15340.37	25515.53	9048.21	6944.78	5877.14	4467.25
ctr28926_c2_g2_i1	1025.50	1540.48	8128.47	8429.72	793.87	2785.64	17031.04	16555.49
ctr28926_c2_g3_i3	17.02	38.97	1087.79	2135.36	666.29	467.58	1602.96	1849.74
ctr29379_c2_g2_i1	49.88	46.88	5.57	9.14	508.35	9.55	64.29	36.91

ctr29379_c2_g2_i3	3308.42	4934.28	8834.73	15289.47	24960.81	7186.21	22390.58	18947.53
ctr29379_c3_g1_i2	0.00	0.00	0.69	0.00	49.86	32.18	10.14	7.80
ctr29379_c3_g2_i1	0.00	0.19	31.66	1.11	115.96	88.42	277.25	194.59
ctr29379_c3_g2_i10	14.31	42.64	103.34	134.18	76.02	26.89	1860.99	1793.70
ctr29379_c3_g2_i11	0.00	0.11	18.00	0.86	8.81	9.21	111.96	76.00
ctr29379_c3_g2_i12	0.10	0.51	4.21	11.34	1.97	0.64	105.80	90.28
ctr29379_c3_g2_i3	23.77	33.05	47.68	76.59	444.73	467.21	129.86	146.67
ctr29379_c3_g2_i4	36.35	89.41	94.88	77.66	62.29	15.07	1950.98	1642.10
ctr29379_c3_g2_i5	0.00	0.66	2.33	0.00	21.44	20.01	16.48	23.11
ctr29379_c3_g2_i6	43.19	75.73	1.23	2.07	8.28	14.06	36.27	28.53
ctr29379_c3_g2_i7	0.00	0.00	0.30	0.00	0.44	0.11	2.68	1.71
ctr29379_c3_g2_i8	0.78	2.79	0.71	0.75	12.55	26.99	879.66	897.28
ctr29379_c3_g2_i9	24.40	65.07	154.00	161.72	27.91	5.60	4096.25	3599.57
ctr29609_c0_g1_i1	0.00	0.00	0.00	0.00	4.58	0.00	0.26	0.00
ctr29609_c1_g1_i2	271.63	129.61	0.00	0.00	1.73	6.31	0.00	0.05
ctr29609_c1_g1_i6	84.65	114.35	47.88	148.06	111.56	38.14	219.52	162.14
ctr29609_c1_g2_i1	0.00	0.09	0.00	0.00	8.47	12.75	14.09	14.57
ctr29609_c1_g2_i2	0.00	0.00	0.00	0.08	0.54	0.52	1.18	4.76
ctr29609_c1_g2_i3	1.07	4.47	0.00	0.14	140.75	410.64	257.77	246.43
ctr29609_c1_g3_i1	111.64	177.65	276.92	402.06	924.62	244.13	748.29	639.81
ctr29609_c1_g4_i1	0.59	0.57	0.00	0.00	0.44	0.00	0.00	0.00
ctr29746_c1_g1_i10	0.41	1.10	0.66	0.94	0.56	1.70	1.80	1.86
ctr29746_c1_g1_i2	0.23	0.18	0.03	0.07	0.01	0.43	0.01	0.13
ctr29746_c1_g1_i6	0.00	0.00	0.00	0.00	6.66	0.00	0.00	0.00
ctr29746_c1_g2_i1	0.00	0.00	0.00	0.00	64.10	11.36	1.29	0.63
ctr29746_c1_g2_i2	0.00	12.00	4.42	2.49	3.81	7.95	75.05	115.34
ctr29746_c1_g2_i3	0.00	0.03	0.00	0.00	0.00	0.01	2.35	0.02
ctr29746_c1_g2_i4	59.61	120.93	95.79	109.62	116.65	92.43	1301.15	1268.54
ctr29746_c1_g2_i5	0.30	0.30	14.69	15.33	0.01	0.26	160.15	158.35
ctr29746_c1_g2_i6	0.18	0.69	9.99	7.96	5.43	4.90	128.24	96.52
ctr29746_c1_g3_i1	0.00	0.00	0.00	0.00	0.00	8.07	1.32	2.20
ctr29746_c1_g3_i2	109.60	132.80	0.00	0.00	520.81	0.03	1130.27	579.50
ctr29746_c1_g3_i3	0.00	0.00	0.00	0.00	0.00	0.00	0.00	0.00
ctr29746_c1_g3_i4	0.67	1.76	0.28	0.11	6.80	4997.39	1.75	0.45
ctr29746_c1_g4_i1	0.23	0.58	0.14	0.12	73.57	1.82	2.49	4.09
**(b) Asparaginyl endopeptidase**								
	**TPM Values** [a]							
**Transcript_ID**	**Flower_1**	**Flower_2**	**Leaf_1**	**Leaf_2**	**Pod_1**	**Pod_2**	**Stem_1**	**Stem_2**
ctr17958_c0_g1_i1	0.00	0.00	0.00	0.00	26.90	0.00	0.00	0.00
ctr24604_c1_g1_i1	10.58	0.19	0.00	0.00	0.00	0.00	0.00	0.00
ctr24604_c1_g2_i2	94.13	1.87	0.00	0.00	0.00	0.00	0.09	0.02

ctr27109_c1_g4_i1	0.59	2.86	2.72	4.64	6.56	4.25	7.77	8.07
ctr28924_c0_g1_i1	0.40	0.15	0.00	0.00	0.00	0.00	0.00	0.00
ctr28924_c0_g1_i2	5.20	0.06	0.00	0.00	0.00	0.00	0.00	0.00
ctr29014_c2_g1_i2	4.36	7.73	38.06	57.99	6.27	27.62	24.39	30.54

(c) Protein disulphide isomerase

	TPM Values [a]							
**Transcript_ID**	**Flower_1**	**Flower_2**	**Leaf_1**	**Leaf_2**	**Pod_1**	**Pod_2**	**Stem_1**	**Stem_2**
ctr16675_c0_g1_i1	0.62	2.36	0.00	0.00	0.00	0.00	0.01	0.00
ctr21421_c0_g1_i1	0.10	0.16	0.00	0.00	0.00	0.01	0.55	0.38
ctr23276_c0_g1_i1	0.20	1.86	0.00	0.00	0.00	0.00	0.00	0.00
ctr23512_c0_g1_i5	51.39	86.65	51.30	38.27	91.22	56.33	90.71	99.42
ctr24519_c0_g1_i1	8.75	0.00	0.00	0.00	0.00	0.00	0.00	0.00
ctr24519_c0_g1_i2	0.06	0.21	0.00	0.00	0.00	0.00	0.00	0.00
ctr24519_c0_g2_i1	7.62	0.12	0.00	0.00	0.00	0.00	0.00	0.00
ctr24519_c0_g3_i1	4.98	0.08	0.00	0.00	0.00	0.00	0.00	0.00
ctr26632_c5_g4_i3	36.49	0.44	0.00	0.00	0.00	0.00	0.00	0.00
ctr28710_c1_g1_i4	8.79	16.31	7.67	6.54	18.05	16.16	23.41	24.20
ctr29427_c2_g3_i1	2.75	0.71	0.00	0.00	0.00	0.00	0.00	0.00
ctr29427_c2_g3_i2	111.03	1.58	0.00	0.00	0.00	0.00	0.02	0.00
ctr30222_c3_g6_i1	17.28	24.59	19.32	13.92	59.02	24.03	33.87	38.37
ctr39989_c0_g1_i1	0.10	0.85	0.00	0.00	0.00	0.00	0.00	0.00
ctr6173_c0_g1_i1	25.91	0.72	0.00	0.00	0.00	0.00	0.00	0.04

(d) Endoplasmic reticulum oxidoreductin-1

	TPM Values [a]							
**Transcript_ID**	**Flower_1**	**Flower_2**	**Leaf_1**	**Leaf_2**	**Pod_1**	**Pod_2**	**Stem_1**	**Stem_2**
ctr203_c0_g1_i1	4.87	0.09	0.00	0.00	0.00	0.00	0.00	0.00

(e) Peptidylprolyl cis-trans isomerase/Cyclophilin

	TPM Values [a]							
**Transcript_ID**	**Flower_1**	**Flower_2**	**Leaf_1**	**Leaf_2**	**Pod_1**	**Pod_2**	**Stem_1**	**Stem_2**
ctr10137_c0_g1_i1	10.99	0.21	0.00	0.00	0.00	0.00	0.00	0.00
ctr11074_c0_g1_i1	1.87	3.38	3.40	2.63	12.51	5.24	11.56	12.96
ctr15543_c0_g1_i1	20.08	0.30	0.00	0.00	0.00	0.00	0.00	0.00
ctr15837_c0_g2_i1	14.42	0.24	0.00	0.00	0.00	0.00	0.00	0.00
ctr17099_c0_g1_i1	6.93	0.06	0.00	0.00	0.00	0.00	0.02	0.00
ctr17583_c0_g1_i1	8.72	0.00	0.00	0.00	0.00	0.00	0.00	0.00
ctr18375_c0_g1_i1	17.46	0.26	0.00	0.00	0.00	0.00	0.00	0.00
ctr18912_c0_g1_i1	7.73	0.19	0.00	0.00	0.00	0.00	0.01	0.00
ctr19397_c0_g1_i1	6.46	0.07	0.00	0.00	0.00	0.00	0.00	0.00
ctr20383_c0_g1_i1	6.71	0.14	0.00	0.00	0.00	0.00	0.00	0.00
ctr22690_c0_g1_i4	0.01	0.04	0.27	0.00	0.00	0.02	0.03	0.01

ctr22690_c0_g1_i5	31.89	54.30	12.75	14.91	32.25	21.40	24.71	26.77
ctr22957_c0_g1_i1	104.57	1.79	0.00	0.00	0.00	0.00	0.08	0.00
ctr22957_c0_g1_i2	0.00	1.25	0.00	0.00	0.00	0.00	0.00	0.00
ctr22957_c0_g1_i3	0.00	0.00	0.00	0.00	0.00	0.00	0.00	0.00
ctr24033_c0_g1_i1	7.12	9.03	44.21	40.12	6.59	16.25	16.87	15.65
ctr24500_c0_g1_i2	0.35	0.90	0.73	0.92	2.23	2.50	1.85	2.21
ctr24674_c0_g1_i1	0.31	2.57	0.00	0.00	0.00	0.00	0.00	0.00
ctr24674_c0_g1_i2	0.80	0.10	0.00	0.00	0.00	0.00	0.00	0.00
ctr24674_c0_g1_i3	0.04	0.46	0.00	0.00	0.00	0.00	0.00	0.00
ctr24700_c0_g1_i1	6.19	10.33	9.11	9.50	11.49	9.98	16.20	16.41
ctr24700_c0_g1_i2	0.37	0.88	1.19	0.92	1.01	1.24	1.59	1.73
ctr25168_c0_g1_i1	0.44	0.25	4.36	2.97	2.15	1.87	1.12	1.49
ctr25226_c0_g1_i11	0.22	0.27	0.19	0.27	0.07	0.31	0.48	0.48
ctr25226_c0_g1_i13	0.10	0.27	0.39	0.40	0.26	0.58	0.93	0.93
ctr25226_c0_g1_i15	0.48	0.63	0.95	0.71	2.24	1.85	1.70	2.79
ctr25226_c0_g1_i2	0.07	0.48	0.29	0.10	0.27	0.09	0.28	0.20
ctr25239_c0_g1_i1	0.12	0.95	0.00	0.00	0.00	0.00	4.07	2.70
ctr25252_c0_g1_i1	0.00	0.04	0.26	0.00	0.00	0.00	0.14	0.00
ctr25252_c0_g1_i2	5.36	12.11	6.21	7.77	10.62	21.37	15.60	18.25
ctr25252_c0_g1_i4	0.52	0.35	0.38	0.37	0.32	0.42	0.41	0.56
ctr26423_c0_g1_i5	50.51	94.82	35.42	28.06	314.06	122.13	157.35	157.42
ctr26897_c1_g2_i13	9.15	13.86	15.24	13.09	9.66	17.12	18.76	21.61
ctr27050_c0_g3_i1	5.44	0.07	0.00	0.00	0.00	0.00	0.00	0.00
ctr27345_c1_g1_i1	0.09	0.16	0.00	0.00	0.00	0.00	0.80	0.53
ctr27345_c3_g2_i22	0.00	0.86	0.09	0.22	0.21	0.40	0.00	0.11
ctr27355_c0_g1_i1	0.00	0.01	0.00	0.00	0.02	0.01	0.14	0.00
ctr27355_c0_g1_i10	0.28	0.08	0.02	0.00	0.09	0.00	0.11	0.43
ctr27652_c1_g2_i7	7.87	12.73	14.52	16.90	18.65	17.46	22.17	25.15
ctr27784_c2_g1_i14	20.03	39.14	15.16	14.54	13.57	32.44	23.38	26.48
ctr27784_c2_g1_i20	0.00	0.00	0.04	0.14	0.26	0.01	0.06	0.02
ctr27784_c2_g1_i28	0.92	2.94	1.41	2.62	0.41	1.62	2.91	4.01
ctr27856_c0_g2_i1	0.01	0.17	0.31	0.58	0.21	0.47	0.51	0.21
ctr27930_c3_g13_i1	9.45	0.22	0.00	0.00	0.00	0.00	0.00	0.00
ctr28568_c0_g5_i4	10.09	17.36	2.86	2.33	10.11	8.67	14.34	15.24
ctr28930_c1_g3_i5	15.99	24.98	21.42	28.31	17.68	18.46	21.04	21.36
ctr28936_c0_g2_i2	176.41	4.22	0.00	0.00	0.00	0.00	0.19	0.03
ctr28936_c0_g2_i3	0.00	1.55	0.00	0.00	0.00	0.00	0.00	0.00
ctr28936_c0_g3_i12	5.00	8.63	6.02	6.52	5.61	3.26	24.88	18.19
ctr28936_c0_g4_i4	2.37	6.50	129.69	101.54	10.68	33.68	7.69	12.97
ctr28936_c0_g4_i6	15.04	27.94	473.64	394.74	54.54	109.57	32.12	34.68
ctr28936_c0_g7_i1	30.50	0.93	0.00	0.00	0.00	0.00	0.00	0.00
ctr29069_c0_g1_i11	0.00	0.05	0.00	0.09	0.00	0.02	0.02	0.15

ctr29069_c0_g1_i13	1.89	3.45	3.20	3.96	2.71	3.14	3.61	4.05
ctr29069_c0_g1_i2	0.00	0.03	0.00	0.00	0.03	0.00	0.02	0.07
ctr29069_c0_g1_i3	0.67	1.54	1.00	0.43	1.41	1.46	1.44	1.08
ctr29069_c0_g1_i6	0.99	2.41	1.18	2.81	2.19	3.20	3.21	4.31
ctr29069_c0_g1_i7	0.54	1.70	1.21	0.47	2.10	1.59	2.33	1.20
ctr29098_c0_g1_i3	3.57	5.54	14.52	14.03	5.82	12.00	5.07	5.85
ctr29582_c2_g1_i1	21.72	44.41	21.98	21.78	33.64	35.07	43.46	42.50
ctr29752_c0_g3_i1	0.12	0.18	0.61	0.49	0.42	0.29	0.53	0.25
ctr30170_c0_g1_i12	0.18	0.51	0.70	0.11	0.05	0.23	2.05	1.04
ctr30170_c0_g1_i15	3.38	5.41	1.62	4.40	5.13	8.18	7.93	8.59
ctr30170_c0_g1_i9	0.00	0.11	0.00	0.07	0.04	0.46	0.12	0.28
ctr30311_c0_g1_i1	1.29	2.95	8.95	9.71	7.41	7.59	8.07	6.74
ctr31571_c0_g1_i1	5.49	0.10	0.00	0.00	0.00	0.00	0.00	0.00
ctr44502_c0_g1_i1	1.00	0.00	0.00	0.00	0.00	0.00	0.00	0.00
ctr68387_c0_g1_i1	11.61	0.17	0.00	0.00	0.00	0.00	0.00	0.00
ctr79517_c0_g1_i1	0.06	0.54	0.00	0.00	0.00	0.00	0.00	0.00
ctr8964_c0_g1_i1	12.15	0.37	0.00	0.00	0.00	0.00	0.00	0.00

[a] TPM value is averaged for technical replicates per biological replicate per plant tissue.

Supplementary Table S3: Summary of *de novo* transcriptome assembly.

**Assembly details**	**Summary statistics**
[a]Total size of transcriptome	402,275,420 bp
[a]Total number of reads	281,871,893
Reads mapped to transcriptome assembly	94.44%
Mean contig length	1441.89 bp
Median contig length	988 bp
Contig N50	2437 bp
Total trinity genes	120,067
Total trinity transcripts	278,991
[a]Percent GC	38.92
[a]Average number of reads in Leaf tissue replicates	16.6 million
[a]Average number of reads in Pod tissue replicates	18.57 million
[a]Average number of reads in Stem tissue replicates	16.82 million
[a]Average number of reads in Flower tissue replicates	18.45 million

[a]after quality filtering with Trimmomatic

Supplementary Table S4: Top 20 enriched GO terms from highly expressed transcripts

**Count**	**GO term**	**Class**	**P-value**
240	regulation of transcription, DNA-templated	BP	7.70E-24
149	RNA splicing	BP	1.05E-04
148	metal ion binding	MF	1.97E-04
137	transmembrane transport	BP	4.89E-11
129	rRNA processing	BP	1.10E-04
120	protein ubiquitination	BP	1.98E-12
117	response to salt stress	BP	2.33E-05
92	response to water deprivation	BP	1.11E-06
90	viral process	BP	1.73E-04
84	recognition of pollen	BP	3.54E-04
76	lipid catabolic process	BP	1.46E-07
76	tricarboxylic acid cycle	BP	5.25E-05
75	defense response	BP	8.38E-20
73	response to wounding	BP	1.56E-04
70	response to oxidative stress	BP	2.95E-05
65	plasma membrane	CC	1.04E-11
64	DNA recombination	BP	1.48E-14
61	serine-type endopeptidase activity	MF	2.79E-04
54	oxidoreductase activity	MF	6.54E-04
48	DNA integration	BP	8.19E-08

Supplementary Table S5: Cyclotide transcript sequences determined in the current transcriptome assembly of *C. ternatea*.

Sl. No.	Transcript ID	GenBank Accession Numbers	Cyclotide Mature Domain Sequence	Topology	[a]Tissue Abundance	Previous Evidence (“Cter” – Craik group) (“Cliotide – Tam group)
1	ctr28192_c2_g1_i2/ ctr28192_c2_g1_i7	MT468661 MT468662	---GIPCGESCVFIPCTITALLGCSCKS-KVCYKN	B	P/S	Cter 14; CliotideT40
2	ctr28192_c2_g2_i1	MT468663	---GIPCGESCVFIPCLTT-VVGCSCKN-KVCYNN	B	P	Cliotide T9
3*	ctr28192_c2_g3_i3	MT468664	--GRPTCGETCFKTKCYTP---GCSCS-YPICKKN	M	L	
4*	ctr28192_c2_g4_i3	MT468665	-GDALKCGETCFGGTCYTP---GCSCD-YPICKKN	M	S	
5*	ctr28192_c2_g6_i1	MT468666	-----------FKTKCYTP---GCSCS-YPVCKRN	partial	P	
6*	ctr28192_c2_g6_i2	MT468667	-GCLPICGETCFKTKCYTK---GCSCS-YPICKKN	M; unusual	P	
7	ctr28495_c0_g2_i1	MT468668	--GLPICGETCFKTKCYTK---GCSCS-YPVCKRN	M	L	Cliotide T15; Cter24
8*	ctr28495_c0_g2_i2	MT468669	--GRPTCGETCFKTKCYTK---GCSCS-YPVCKRN	M	L	
9	ctr28495_c0_g3_i1	MT468670	-GNPIVCGETCFFQKCYTP---GCSCD-AVICTNN	H	L	Cter 25; CliotideT41
10	ctr28495_c0_g4_i1	MT468671	-GDPLACGETCFGGTCYTP---GCVCDPWPICTKN	M	P	Cter 4; CliotideT39
11*	ctr28495_c0_g5_i3	MT468672	-ANIPMTCGPCLTDECWTP---GCEYH-CKYCKNS	H; unusual; acyclic	L	
12*	ctr28495_c0_g7_i1/ ctr28495_c0_g7_i3	MT468673/ MT468674	---LPTCGETCTLGTCYVP---DCSCS-WPICMKN	M	P/F	
13	ctr28841_c1_g1_i3	MT468675	---KIPCGESCVWIPCFTS-AFGCYCQS-KVCYHS	B; acyclic	S	Cliotide T38
14*	ctr28841_c1_g1_i4/ ctr28841_c1_g1_i5/ ctr28841_c1_g1_i6	MT468676/ MT468677/ MT468678	---------SCVWIPCITG-AIGCSCKN-RVCYRN	partial	S/P/P	
15	ctr28841_c1_g1_i7	MT468679	--DTTPCGESCVWIPCVSS-IVGCSCQN-KVCYQN	B	P	Cliotide T13; Cter 23
16	ctr28841_c1_g1_i8	MT468680	--GFNSCSEACVYLPCFSK---GCSCFK-RQCYKN	H	P	Cter 34; CliotideT33
17	ctr28841_c1_g2_i1	MT468681	--GVIPCGESCVFIPCIST-VIGCSCKN-KVCYRN	B	S	Cter A
18*	ctr28841_c1_g3_i1	MT468682	---GISCGESCVFIPCITG-IAGCSCKN-KVCYLN	B	P	
19*	ctr28841_c1_g4_i1	MT468683	--------ESCVWIPCLTG-YFGCYCQS-KVCYHN	partial	S	
20*	ctr28841_c2_g3_i1	MT468684	--CVIPCGESRVFIPCITG-AIGCSCKS-KVCYRN	B; unusual	P	
21*	ctr28841_c2_g4_i2	MT468685	--ARIPCGESCVWIPCTITALVGCACHE-K-----	partial	S	

22*	ctr28926_c1_g1_i1/ ctr28926_c1_g1_i4	MT468686/ MT468689	-GSITGCGGGCLLGRCYRP---GCTCV-RRICRRN	H	L/L	
23	ctr28926_c1_g1_i2/ ctr28926_c1_g1_i6	MT468687/ MT468690	-GSAIRCGERCLLGRCHRP---GCTCI-RRICRRN	B	L/L	Cter 13
24*	ctr28926_c1_g1_i3	MT468688	-GSAIRCGERCLLGRCHRP---GCTCV-RRICRRN	H	L	
25	ctr28926_c1_g2_i1	MT468691	-GDLFKCGETCFGGTCYTP---GCSCD-YPICKNN	M	S	Cliotide T32
26*	ctr28926_c1_g3_i1	MT468692	GSSIVTCGETCLRGKCYTP---GCTCV-RPICKKN	M	L	
27	ctr28926_c1_g3_i2	MT468693	-GSVIGCGETCLRGRCYTP---GCTCD-HGICKKN	H	L	Cliotide T16
28	ctr28926_c2_g2_i1	MT468694	--GLPICGETCFTGTCYTP---GCTCS-YPVCKKN	M	S	Cliotide T18; Cter 6
29*	ctr28926_c2_g3_i3	MT468695	-GDPFKCGESCFAGKCYTP---GCTCS-RPICKKN	M	L	
30*	ctr29379_c2_g2_i1/ ctr29379_c2_g2_i3	MT468696/ MT468697	---GSPCGESCVFIPCIST-VIGCSCKN-KVCYRN	B	P/P	
31*	ctr29379_c3_g1_i2	MT468698	---GIPCGESCVYIPCTVTALLGCSCKN-KVCFRN	B	P	
32	ctr29379_c3_g2_i1	MT468699	--DTIPCGESCVWIPCISS-ILGCSCKD-KVCYHN	B	S	Cliotide T14
33*	ctr29379_c3_g2_i10/ ctr29379_c3_g2_i12	MT468700/ MT468702	---GIPCGESCVFISCTVTALLGCSCKD-KVCYKN	B	S/S	
34*	ctr29379_c3_g2_i3/ ctr29379_c3_g2_i4/ ctr29379_c3_g2_i9/ ctr29746_c1_g2_i4	MT468703/ MT468704/ MT468709/ MT468724	--DTIPCGESCVFIPCTSS-VLGCSCKD-KFCYNN	B	P/S/S/S	
35	ctr29379_c3_g2_i5	MT468705	---GIPCGESCVFIPCTVTALLGCSCKD-KVCYKN	B	S	Cter R; CliotideT7
36*	ctr29379_c3_g2_i6	MT468706	--NIFPCGESCVYIPCITS-IVGCSCQN-KVCYNN	B	F	
37	ctr29379_c3_g2_i7/ ctr29379_c3_g2_i11	MT468707/ MT468701	---GALCDERCTYVPCISA-ARGCSCNIHRVCSMN	B	S/S	Cter 29; CliotideT44
38*	ctr29379_c3_g2_i8	MT468708	--GGTACGESCIYLPCISGVFEGCSCQN-KACYKN	B	S	
39	ctr29609_c0_g1_i1	MT468710	---GIPCGESCVFIPCTITALLGCSCKD-KVCYKN	B	P	Cliotide T11; Cter 21
40	ctr29609_c1_g1_i2/ ctr29609_c1_g1_i6	MT468711/ MT468712	--SYIPCGESCVYIPCTVTALLGCSCSN-KVCYKN	B	F/S	Cter 10; CliotideT34
41	ctr29609_c1_g2_i1	MT468713	---DLICSSTCLHTPCKAS---VCYCK-NAVCYKN	H	P	Cter 18; CliotideT43
42	ctr29609_c1_g2_i2/ ctr29609_c1_g2_i3	MT468714/ MT468715	---DLQCAETCVHSPCIGP----CYCKHGVICYKN	H	S/P	Cter 31; CliotideT46

43*	ctr29609_c1_g3_i1	MT468716	---GIPCGESCVFLPCFI--IPGCSCKD-KVCYLN	B	P	
44	ctr29609_c1_g4_i1	MT468717	--VDGFCLETCVILPCFSS-VAGCYCHG-STCMRG	B; acyclic	F	Cliotide T37
45	ctr29746_c1_g1_i2/<br>ctr29746_c1_g1_i6/<br>ctr29746_c1_g1_i10	MT468720/<br>MT468719/<br>MT468718	---GIPCGESCVFIPCISS-VVGCSCKS-KVCYNN	B	P/P/S	Cliotide T8
46	ctr29746_c1_g2_i1/<br>ctr29746_c1_g2_i6	MT468721/<br>MT468726	---GIPCGESCVYIPCTVTALLGCSCKN-KVCYRN	B	P/S	Cliotide T54
47*	ctr29746_c1_g2_i2	MT468722	---GIPCVESCVFIPCTVTALLGCSCKD-KVCYKN	B	S	
48*	ctr29746_c1_g2_i3/<br>ctr29746_c1_g2_i5	MT468723/<br>MT468725	---GVPCGESCVYIPCTVTALLGCSCKN-KVCYRN	B	S/S	
49	ctr29746_c1_g3_i1/<br>ctr29746_c1_g3_i2/<br>ctr29746_c1_g3_i4	MT468727/<br>MT468728/<br>MT468730	---SIPCGESCVYIPCLTT-IVGCSCKS-NVCYSN	B	P/S/P	Cter 19
50*	ctr29746_c1_g3_i3	MT468729	-------------ALPIST-IVGCSCKS-NVCYSN	partial	F	
51	ctr29746_c1_g4_i1	MT468731	---GVPCAESCVWIPCTVTALLGCSCKD-KVCYLN	B	P	Cter B

*Precursor sequences with novel cyclotides domains identified in the current study.

[a]Tissue abundance in the current transcriptome assembly using untransformed TPM values. P: pod; L: leaf; S: stem; F: flower.

Supplementary Table S6: Identification of cyclotide sequences in purified fractions of *C. ternatea* stems, leaves, pods and flowers crude extracts by MALDI-TOF MS (See Supplementary Fig. S

Sl. No.	Transcript ID	Peptide ID	Calc. Mass (Monoisotopic) [Da]	[a]Evidence in Tissue Fractions
1	ctr28192_c2_g1_i2/ ctr28192_c2_g1_i7	ctr pep 1	3212.5	S(C), L(E), P(C)
2	ctr28192_c2_g2_i1	ctr pep 2	3126.4	P(D), P(E)
3*	ctr28192_c2_g3_i3	ctr pep 3	3150.4	S(B), L(B), L(C)
4*	ctr28192_c2_g4_i3	ctr pep 4	3109.3	S(D), S(E), L(D), L(E), P(D), P(E), F(E)
5*	ctr28192_c2_g6_i1	ctr pep 5	2262.0	
6*	ctr28192_c2_g6_i2	ctr pep 6	3253.5	
7	ctr28495_c0_g2_i1	ctr pep 7	3164.5	P(D)
8*	ctr28495_c0_g2_i2	ctr pep 8	3195.4	L(C), L(D), L(E)
9	ctr28495_c0_g3_i1	ctr pep 9	3162.3	S(B)
10	ctr28495_c0_g4_i1	ctr pep 10	3183.3	P(C)
11*	ctr28495_c0_g5_i3	ctr pep 11	3342.4	
12*	ctr28495_c0_g7_i1/ ctr28495_c0_g7_i3	ctr pep 12	3000.2	P(B)
13	ctr28841_c1_g1_i3	ctr pep 13	3321.4	P(E)
14*	ctr28841_c1_g1_i4/ ctr28841_c1_g1_i5/ ctr28841_c1_g1_i6	ctr pep 14	2623.2	
15	ctr28841_c1_g1_i7	ctr pep 15	3298.4	F(C)
16	ctr28841_c1_g1_i8	ctr pep 16	3247.4	
17	ctr28841_c1_g2_i1	ctr pep 17	3267.6	P(D)
18*	ctr28841_c1_g3_i1	ctr pep 18	3057.4	S(C), P(D), F(D)
19*	ctr28841_c1_g4_i1	ctr pep 19	2880.2	
20*	ctr28841_c2_g3_i1	ctr pep 20	3281.6	P(E)
21*	ctr28841_c2_g4_i2	ctr pep 21	2837.3	
22*	ctr28926_c1_g1_i1/ ctr28926_c1_g1_i4	ctr pep 22	3162.5	S(B)
23	ctr28926_c1_g1_i2/ ctr28926_c1_g1_i6	ctr pep 23	3390.7	
24*	ctr28926_c1_g1_i3	ctr pep 24	3376.7	
25	ctr28926_c1_g2_i1	ctr pep 25	3171.3	F(D)
26*	ctr28926_c1_g3_i1	ctr pep 26	3252.5	
27	ctr28926_c1_g3_i2	ctr pep 27	3106.4	P(B), F(B)
28	ctr28926_c2_g2_i1	ctr pep 28	3021.3	S(E), P(C)
29*	ctr28926_c2_g3_i3	ctr pep 29	3175.4	
30*	ctr29379_c2_g2_i1/ ctr29379_c2_g2_i3	ctr pep 30	3142.4	L(D)
31*	ctr29379_c3_g1_i2	ctr pep 31	3253.5	
32	ctr29379_c3_g2_i1	ctr pep 32	3348.5	
33*	ctr29379_c3_g2_i10/ ctr29379_c3_g2_i12	ctr pep 33	3216.5	
34*	ctr29379_c3_g2_i3/ ctr29379_c3_g2_i4/ ctr29379_c3_g2_i9/ ctr29746_c1_g2_i4	ctr pep 34	3308.4	P(C)
35	ctr29379_c3_g2_i5	ctr pep 35	3226.5	P(D), F(D)

36*	ctr29379_c3_g2_i6	ctr pep 36	3346.5	P(E)
37	ctr29379_c3_g2_i7/ ctr29379_c3_g2_i11	ctr pep 37	3307.4	
38*	ctr29379_c3_g2_i8	ctr pep 38	3280.4	P(B), F(B), F(C)
39	ctr29609_c0_g1_i1	ctr pep 39	3240.5	L(D), F(C)
40	ctr29609_c1_g1_i2/ ctr29609_c1_g1_i6	ctr pep 40	3393.5	P(B)
41	ctr29609_c1_g2_i1	ctr pep 41	3042.4	
42	ctr29609_c1_g2_i2/ ctr29609_c1_g2_i3	ctr pep 42	3059.3	
43*	ctr29609_c1_g3_i1	ctr pep 43	3083.4	P(D)
44	ctr29609_c1_g4_i1	ctr pep 44	3233.4	F(D), F(E)
45	ctr29746_c1_g1_i2/ ctr29746_c1_g1_i6/ ctr29746_c1_g1_i10	ctr pep 45	3071.4	S(D), S(E), L(B), L(C), L(D), P(C), P(D), F(D), F(E)
46	ctr29746_c1_g2_i1/ ctr29746_c1_g2_i6	ctr pep 46	3269.5	
47*	ctr29746_c1_g2_i2	ctr pep 47	3268.6	
48*	ctr29746_c1_g2_i3/ ctr29746_c1_g2_i5	ctr pep 48	3255.5	
49	ctr29746_c1_g3_i1/ ctr29746_c1_g3_i2/ ctr29746_c1_g3_i4	ctr pep 49	3118.4	S(E)
50*	ctr29746_c1_g3_i3	ctr pep 50	2038.0	
51	ctr29746_c1_g4_i1	ctr pep 51	3250.5	P(E), F(D)

*Precursor sequences with novel cyclotides domains identified in the current study.

[a]Stems: S; Leaves: L; Pods: P; Flowers: F. Letters within parentheses indicate fraction ID (Refer to Supplementary Fig. S)

Supplementary Table S7: AEP transcript sequences determined in the current transcriptome assembly of *C. ternatea*.

GenBank Accession Numbers	Transcript ID	*Gene Name	AA Length	[a]Tissue Abundance
MT468732	ctr17958_c0_g1_i1	CtAEP7	442	P
MT468733	ctr24604_c1_g1_i1	CtAEP8	457	F
MT468734	ctr24604_c1_g2_i2	CtAEP9	448	F
MT468735	ctr27109_c1_g4_i1	CtAEP10	376	S
MT468736	ctr28924_c0_g1_i1	CtAEP11	451	F
MT468737	ctr28924_c0_g1_i2	CtAEP12	478	F
MT468738	ctr29014_c2_g1_i2	CtAEP2	465	L

*CtAEP sequences identified in the current study.

[a]Tissue abundance in the current transcriptome assembly using untransformed TPM values. P: pod; L: leaf; S: stem; F: flower.

Supplementary Table S8: PDI transcript sequences determined in the current transcriptome assembly of *C. ternatea.*

GenBank Accession Numbers	Transcript ID	*Gene Name	Length	aDomain Composit-ion	Active Site Motifs	C-terminal Retention Motif	bTissue Abund-ance
MT468739	ctr16675_c0_g1_i1	CtPDI1	479	a-b-b’-a’	CGHC, CGHC	KDEL	F
MT468740	ctr21421_c0_g1_i1	CtPDI2	487	a-b-b’-a’	CGHC, CGFC	HDEL	S
MT468741	ctr23276_c0_g1_i1	CtPDI3	470	a-b-b’-a’	CGHC, CGHC	KEEL	F
MT468742	ctr23512_c0_g1_i5	CtPDI4	336	a°-a-d	CGHC, CGHC	STYV	S
MT468743	ctr24519_c0_g1_i1	CtPDI5	477	a-b-b’-a’	CGHC, CGHC	KEEL	F
MT468744	ctr24519_c0_g1_i2	CtPDI6	562	a-b-b’-a’	CGHC, CGHC	KEEL	F
MT468745	ctr24519_c0_g2_i1	CtPDI7	473	a-b-b’-a’	CGHC, CGHC	KEEL	F
MT468746	ctr24519_c0_g3_i1	CtPDI8	413	a°-a-b	CGHC, CGHC	KEEL	F
MT468747	ctr26632_c5_g4_i3	CtPDI9	487	a-b-b’-a’	CGHC, CGHC	KDEL	F
MT468748	ctr28710_c1_g1_i4	CtPDI10	498	c-a-b-b’-a’	CPRS, CINC	KDEL	S
MT468749	ctr29427_c2_g3_i1	CtPDI11	470	a-b-b’-a’	CGHC, CGHC	KDEL	F
MT468750	ctr29427_c2_g3_i2	CtPDI12	470	a-b-b’-a’	CGHC, CGHC	KDEL	F
MT468751	ctr30222_c3_g6_i1	CtPDI13	410	a°-a-b	CGHC, CGHC	KDQI	P
MT468752	ctr39989_c0_g1_i1	CtPDI14	415	a°-a-b	CGHC, CGHC	KDEL	F
MT468753	ctr6173_c0_g1_i1	CtPDI15	416	a°-a-b	CGHC, CGHC	KEEL	L

*CtPDI sequences identified in the current study.

aPutative domain composition include the a-type (a, a’ a°) and b-type (b, b’) domains, N-terminal acidic domain (c) and Erp29c domain (d).

bTissue abundance in the current transcriptome assembly using untransformed TPM values. P: pod; L: leaf; S: stem; F: flower.

Supplementary Table S9: PPIase transcript sequences determined in the current transcriptome assembly of *C. ternatea*.

GenBank Accession Numbers	Transcript ID	*Gene Name	AA length	[a]Domain Type	[b]Predicted localization					[c]Tissue Abundance
					Deep-Loc	Yloc	Sher-Loc	CELLO	Multi-Loc	
MT468755	ctr10137_c0_g1_i1	CtCYP1	338	MD	M	ER	ER	Cy	G	F
MT468756	ctr11074_c0_g1_i1	CtCYP2	360	MD	Cy	Cy	Cy	ER	Cy	S
MT468757	ctr15543_c0_g1_i1	CtCYP3	175	SD	Cy	Cy	Cy	Cy	Cy	F
MT468758	ctr15837_c0_g2_i1	CtCYP4	184	SD	Cy	Cy	Cy	Cy	Cy	F
MT468759	ctr17099_c0_g1_i1	CtCYP5	497	SD	N	Cy	Pr	N	Pr	F
MT468760	ctr17583_c0_g1_i1	CtCYP6	165	SD	Cy	Cy	Cy	Cy	Cy	F
MT468761	ctr18375_c0_g1_i1	CtCYP7	209	SD	P	M	M	Cy	M	F
MT468762	ctr18912_c0_g1_i1	CtCYP8	548	MD	N	Cy	N	N	N	F
MT468763	ctr19397_c0_g1_i1	CtCYP9	624	MD	N	Cy	Cy	Cy	Cy	F
MT468764	ctr20383_c0_g1_i1	CtCYP10	521	MD	N	Cy	Cy	Cy	Cy	F
MT468765	ctr22690_c0_g1_i4	CtCYP11	196	SD	E	PM	E	PM	ER	L
MT468766	ctr22690_c0_g1_i5	CtCYP12	226	SD	ER	ER	V	Cy	V	F
MT468767	ctr22957_c0_g1_i1	CtCYP13	220	SD	ER	ER	ER	Cy	ER	F
MT468768	ctr22957_c0_g1_i2	CtCYP14	250	SD	P	ER	ER	Cy	ER	F
MT468769	ctr22957_c0_g1_i3	CtCYP15	250	SD	ER	ER	ER	Cy	ER	F
MT468770	ctr24033_c0_g1_i1	CtCYP16	243	SD	P	M	Cy	Cy	Ch	L
MT468771	ctr24500_c0_g1_i2	CtCYP17	339	SD	G	G	E	Cy	E	P
MT468772	ctr24674_c0_g1_i1	CtCYP18	253	SD	P	ER	ER	Cy	ER	F
MT468773	ctr24674_c0_g1_i2	CtCYP19	229	SD	ER	Cy	ER	Cy	ER	F
MT468774	ctr24674_c0_g1_i3	CtCYP20	229	SD	ER	Cy	ER	Cy	ER	F
MT468775	ctr24700_c0_g1_i1	CtCYP21	615	MD	N	Cy	Cy	Cy	Cy	S
MT468776	ctr24700_c0_g1_i2	CtCYP22	231	SD	M	ER	ER	Pr	ER	S
MT468777	ctr25168_c0_g1_i1	CtCYP23	361	MD	Cy	Cy	Cy	Cy	Cy	L
MT468778	ctr25226_c0_g1_i11	CtCYP24	666	SD	N	Cy	N	N	N	S
MT468779	ctr25226_c0_g1_i13	CtCYP25	196	SD	Cy	Cy	Cy	Cy	Cy	S
MT468780	ctr25226_c0_g1_i15	CtCYP26	575	SD	N	Cy	N	N	N	S
MT468781	ctr25226_c0_g1_i2	CtCYP27	552	SD	N	Cy	N	N	N	F
MT468782	ctr25239_c0_g1_i1	CtCYP28	210	SD	M	M	M	Cy	M	S
MT468783	ctr25252_c0_g1_i1	CtCYP29	519	MD	Cy	Cy	Cy	M	Cy	L
MT468784	ctr25252_c0_g1_i2	CtCYP30	598	MD	N	Cy	Cy	Cy	Cy	P

MT468785	ctr25252_c0_g1_i4	CtCYP31	546	MD	Cy	Cy	Cy	Cy	Cy	P
MT468786	ctr26423_c0_g1_i5	CtCYP32	200	SD	ER	ER	ER	Cy	ER	P
MT468787	ctr26897_c1_g2_i13	CtCYP33	430	SD	N	Cy	N	N	N	S
MT468788	ctr27050_c0_g3_i1	CtCYP34	470	MD	N	Cy	N	N	N	F
MT468789	ctr27345_c1_g1_i1	CtCYP35	227	SD	P	ER	ER	E	ER	S
MT468790	ctr27345_c3_g2_i22	CtCYP36	171	SD	Cy	ER	ER	M	ER	F
MT468791	ctr27355_c0_g1_i1	CtCYP37	157	SD	Cy	Cy	Cy	M	Cy	S
MT468792	ctr27355_c0_g1_i10	CtCYP38	164	SD	Cy	Cy	Cy	M	Cy	F
MT468793	ctr27652_c1_g2_i7	CtCYP39	223	SD	ER	ER	ER	Cy	ER	S
MT468794	ctr27784_c2_g1_i14	CtCYP40	229	SD	M	ER	M	M	G	F
MT468795	ctr27784_c2_g1_i20	CtCYP41	274	SD	M	PM	M	PM	G	P
MT468796	ctr27784_c2_g1_i28	CtCYP42	229	SD	ER	G	M	Cy	M	S
MT468797	ctr27856_c0_g2_i1	CtCYP43	174	SD	Cy	M	Cy	Cy	Cy	L
MT468798	ctr27930_c3_g13_i1	CtCYP44	542	MD	Cy	N	Cy	N	Pr	F
MT468799	ctr28568_c0_g5_i4	CtCYP45	341	SD	G	PM	PM	E	PM	F
MT468800	ctr28930_c1_g3_i5	CtCYP46	160	SD	Cy	Cy	Cy	M	Cy	L
MT468801	ctr28936_c0_g2_i2	CtCYP47	209	SD	M	Ch	M	M	M	F
MT468802	ctr28936_c0_g2_i3	CtCYP48	209	SD	M	Ch	M	PM	M	F
MT468803	ctr28936_c0_g3_i12	CtCYP49	255	SD	P	Ch	Cy	Cy	Ch	S
MT468804	ctr28936_c0_g4_i4	CtCYP50	197	SD	E	ER	ER	Cy	ER	L
MT468805	ctr28936_c0_g4_i6	CtCYP51	185	SD	P	Cy	Cy	Cy	Cy	L
MT468806	ctr28936_c0_g7_i1	CtCYP52	164	SD	Cy	Cy	Cy	Cy	Cy	F
MT468807	ctr29069_c0_g1_i11	CtCYP53	336	SD	N	Cy	Cy	N	Cy	S
MT468808	ctr29069_c0_g1_i13	CtCYP54	493	SD	N	Cy	N	N	N	L
MT468809	ctr29069_c0_g1_i2	CtCYP55	256	SD	Cy	Cy	Cy	Cy	Cy	S
MT468810	ctr29069_c0_g1_i3	CtCYP56	496	SD	N	Cy	N	N	N	P
MT468811	ctr29069_c0_g1_i6	CtCYP57	496	SD	N	Cy	N	N	N	S
MT468812	ctr29069_c0_g1_i7	CtCYP58	493	SD	N	Cy	N	N	N	P
MT468813	ctr29098_c0_g1_i3	CtCYP59	221	SD	Cy	Cy	Cy	Cy	Cy	L
MT468814	ctr29582_c2_g1_i1	CtCYP60	190	SD	Cy	Cy	Cy	Cy	Cy	F
MT468815	ctr29752_c0_g3_i1	CtCYP61	368	MD	P	Ch	M	N	M	L
MT468816	ctr30170_c0_g1_i12	CtCYP62	396	MD	N	Cy	N	Cy	N	S
MT468817	ctr30170_c0_g1_i15	CtCYP63	606	MD	N	Cy	N	N	N	S

MT468818	ctr30170_c0_g1_i9	CtCYP64	315	MD	Cy	Cy	Cy	Cy	Cy	P
MT468819	ctr30311_c0_g1_i1	CtCYP65	163	SD	Cy	Ch	Cy	Cy	Cy	L
MT468820	ctr31571_c0_g1_i1	CtCYP66	360	MD	Cy	Cy	Cy	PM	Cy	F
MT468821	ctr44502_c0_g1_i1	CtCYP67	257	SD	Cy	Cy	Cy	Cy	Cy	F
MT468822	ctr68387_c0_g1_i1	CtCYP68	161	SD	Cy	Cy	Cy	Cy	Cy	F
MT468823	ctr79517_c0_g1_i1	CtCYP69	161	SD	Cy	Cy	Cy	E	Cy	F
MT468824	ctr8964_c0_g1_i1	CtCYP70	206	SD	ER	ER	ER	Cy	ER	F

[*]CtCYP sequences identified in the current study.

[a]SD: single-domain; MD: multi-domain

[b]Cy: cytosol; Ch: chloroplast; E: extra cellular; N: nucleus; M: mitochondria; V: vacuole; P: plastid; Pr: peroxisomal; ER: endoplasmic reticulum; G: Golgi apparatus; PM: plasma membrane

[c]Tissue abundance in the current transcriptome assembly using untransformed TPM values. P: pod; L: leaf; S: stem; F: flower.
