# Supplementary material for: Transcriptomic profiling of the medicinal plant Clitoria ternatea: identification of potential genes in cyclotide biosynthesis
Source: Sci Rep. 2020 Jul 29;10:12658. doi: 10.1038/s41598-020-69452-7 (PMC7391643; doi:10.1038/s41598-020-69452-7)
Supplement: Supplementary file 3 — Supplementary Data 2 [file 41598_2020_69452_MOESM3_ESM.docx]

**1. Clitoria ternatea cyclotide precursor, internal, ctr28192_c2_g6_i1, 3-224(+)**

**Query string:** TTCAAAACTAAGTGTTACACGCCAGGTTGTTCATGTTCATATCCAGTTTGCAAGAGAAAC

**Reads:**

>1

TTCAAAACTAAGTGTTACACGCCAGGTTGTTCATGTTCATATCCAGTTTGCAAGAGAAACCATATAATTGCAATTGAAGCGAAGACAGTGGATGAACATC

>2

CCTATCTGTGGAGAAACTTGTTTCAAAACTAAGTGTTACACGCCAGGTTGTTCATGTTCATATCCAGTTTGCAAGAGAAACCATATAATTGCAATTGAAG

>3

CTTGTTTCAAAACTAAGTGTTACACGCCAGGTTGTTCATGTTCATATCCAGTTTGCAAGAGAAACCATATAATTGCAATTGAAGCGAAGACAGTAGATCG

>4

GAAGGGGGTTTTCCTATCTGGGGAGAAACTTGTTTCAAAACTAAGTGTTACACGCCAGGTTGTTCATGTTCATATCCAGTTTGCAAGAGAAACTATATAA

>5

GGGTTTTCCTATCTGTGGAGAAACTTGTTTCAAAACTAAGTGTTACACGCCAGGTTGTTCATGTTCATATCCAGTTTGCAAGAGAAACCATATAATTGCA

>6

GAAGACAGAAGGGGGTTTTCCTATCTGTGGAGAAACTTGTTTCAAAACTAAGTGTTACACGCCAGGTTGTTCATGTTCATATCCAGTTTGCAAGAGAAAC

>7

CTTGTTTCAAAACTAAGTGTTACACGCCAGGTTGTTCATGTTCATATCCAGTTTGCAAGAGAAACCATATAATTGCAATTGAAGCGAAGACAGTGGATGA

>8

AGGGGGTTTTCCTATCTGTGGAGAAACTTGTTTCAAAACTAAGTGTTACACGCCAGGTTGTTCATGTTCATATCCAGTTTGCAAGAGAAACCATATAATT

>9

AGGGGGTTTTCCTATCTGTGGAGAAACTTGTTTCAAAACTAAGTGTTACACGCCAGGTTGTTCATGTTCATATCCAGTTTGCAAGAGAAACCATATAATT

>10

CTTGTTTCAAAACTAAGTGTTACACGCCAGGTTGTTCATGTTCATATCCAGTTTGCAAGAGAAACCATATAATTGCAATTGAAGCGAAGACAGTGGATGA

>11

ATCTGTGGAGAAACTTGTTTCAAAACTAAGTGTTACACGCCAGGTTGTTCATGTTCATATCCAGTTTGCAAGAGAAACCATGTAATTGCAATTGAAACGA

>12

GAAGACAGAAGGGGGTTTTCCTATCTGTGGAGAAACTTGTTTCAAAACTAAGTGTTACACGCCAGGTTGTTCATGTTCATATCCAGTTTGCAAGAGAAAC

>13

CTTGTTTCAAAACTAAGTGTTACACGCCAGGTTGTTCATGTTCATATCCAGTTTGCAAGAGAAACCATATCATTGCAATTGAAGCGAAGACAGTGGATGA

>14

CTTGTTTCAAAACTAAGTGTTACACGCCAGGTTGTTCATGTTCATATCCAGTTTGCAAGAGAAACCATATAATTGCAATTGAAGAGAAGACAGTGGATGA

>15

CAGAAGGGGGTTTTCCTATCTGTGGAGAAACTTGTTTCAAAACTAAGTGTTACACGCCAGGTTGTTCATGTTCATATCCAGTTTGCAAGAGAAACCATAT

>16

CCTATCTGTTGAGAAACTTGTTTCAAAACTAAGTGTTACACGCCAGGTTGTTCATGTTCATATCCAGTTTGCAAGAGAAACCATATAATTGCAATTGAAG

>17

AGGGGGTTTTCCTATCTGTGGAGAAACTTGTTTCAAAACTAAGTGTTACACGCCAGGTTGTTCATGTTCATATCCAGTTTGCAAGAGAAACCATATAATT

>18

TTTCAAAACTAAGTGTTACACGCCAGGTTGTTCATGTTCATATCCAGTTTGCAAGAGAAACCATATAATTGCAATTGAAGCGAAGACAGTGGATGAACAT

>19

GAGAAACTTGTTTCAAAACTAAGTGTTACACGCCAGGTTGTTCATGTTCATATCCAGTTTGCAAGAGAAACCATATAATTGCAATTGAAGCGAAGACAGT

>20

GGAGAAACTTGTTTCAAAACTAAGTGTTACACGCCAGGTTGTTCATGTTCATATCCAGTTTGCAAGAGAAACCATATAATTGCAATTGAAGCGAAGATCG

>21

CTTGTTTCAAAACTAAGTGTTACACGCCAGGTTGTTCATGTTCATATCCAGTTTGCAAGAGAAACCATATAATTGCAATTGAAGCGAAGACAGTGGATGA

>22

CAGAAGGGGGTTTTCCTATCTGTGGAGAAACTTGTTTCAAAACTAAGTGTTACACGCCAGGTTGTTCATGTTCATATCCAGTTTGCAAGAGAAACCATAT

>23

GGGGTTTTCCTATCTGTGGAGAAACTTGTTTCAAAACTAAGTGTTACACGCCAGGTTGTTCATGTTCATATCCAGTTTGCAAGAGAAACCATATAATTGC

>24

CTTGTTTCAAAACTAAGTGTTACACGCCAGGTTGTTCATGTTCATATCCAGTTTGCAAGAGAAACCATATAATTGCAATTGAAGCGAAGAGATCGGAAGA

>25

CTTGTTTCAAAACTAAGTGTTACACGCCAGGTTGTTCATGTTCATATCCAGTTTGCAAGAGAAACCATATAATTGCAATTGAAGCGAAGACAGTGGATGA

>26

CTTGTTTCAAAACTAAGTGTTACACGCCAGGTTGTTCATGTTCATATCCAGTTTGCAAGAGAAACCATATAATTGCAATTGAAGCGAAGACAGTGGATGA

>27

CTTGTTTCAAAACTAAGTGTTACACGCCAGGTTGTTCATGTTCATATCCAGTTTGCAAGAGAAACCATATAATTGCAATTGAAGCGAAGACAGTGGATGA

>28

GACAGAAGGGGGTTTTCCTATCTGTGGAGAAACTTGTTTCAAAACTAAGTGTTACACGCCAGGTTGTTCATGTTCATATCCAGTTTGCAAGAGAAACCAT

>29

CTTGTTTCAAAACTAAGTGTTACACGCCAGGTTGTTCATGTTCATATCCAGTTTGCAAGAGAAACCATATAATTGCAATTGAAGCGAAGACAGTGGATGA

>30

TTCAAAACTAAGTGTTACACGCCAGGTTGTTCATGTTCATATCCAGTTTGCAAGAGAAACCATATAATTGCAATTGAAGCGAAGACAGTGGATGAACATC

>31

GGGGGTTTTCCTATCTGTGGAGAAACTTGTTTCAAAACTAAGTGTTACACGCCAGGTTGTTCATGTTCATATCCAGTTTGCAAGAGAAACCATATAATTG

>32

GAAACTTGTTTCAAAACTAAGTGTTACACGCCAGGTTGTTCATGTTCATATCCAGTTTGCAAGAGAAACCATATAATTGCAATTGAAGCGAAGACAGTGG

>33

GGGGTTTTCCTATCTGTGGAGAAACTTGTTTCAAAACTAAGTGTTACACGCCAGGTTGTTCATGTTCATATCCAGTTTGCAAGAGAAACCATATAATTGC

>34

GAAACTTGTTTCAAAACTAAGTGTTACACGCCAGGTTGTTCATGTTCATATCCAGTTTGCAAGAGAAACCATATAATTGCAATTGAAGCGAAGACAGTGG

>35

TTCAAAACTAAGTGTTACACGCCAGGTTGTTCATGTTCATATCCAGTTTGCAAGAGAAACCATATAATTGCAATTGAAGCGAAGACAGTGGATGAACATC

>36

AGGGGGTTTTCCTATCTGTGGAGAAACTTGTTTCAAAACTAAGTGTTACACGCCAGGTTGTTCATGTTCATATCCAGTTTGCAAGAGAAACCATATAATT

>37

GGGGGTTTTCCTATCTGTGGAGAAACTTGTTTCAAAACTAAGTGTTACACGCCAGGTTGTTCATGTTCATATCCAGTTTGCAAGAGAAACTAGATCGGAA

>38

CCTATCTGTGGAGAAACTTGTTTCAAAACTAAGTGTTACACGCCAGGTTGTTCATGTTCATATCCAGTTTGCAAGAGAAACCATATAATTGCAATTGAAG

>39

CTTGTTTCAAAACTAAGTGTTACACGCCAGGTTGTTCATGTTCATATCCAGTTTGCAAGAGAAACCATATAATTGCAATTGAAGCGAAGACAGTGGATGA

>40

AACTTGTTTCAAAACTAAGTGTTACACGCCAGGTTGTTCATGTTCATATCCAGTTTGCAAGAGAAACCATATAATTGCAATTGAAGCGAAGACAGTAGAT

>41

GAGAAACTTGTTTCAAAACTAAGTGTTACACGCCAGGTTGTTCATGTTCATATCCAGTTTGCAAGAGAAACCATATAATTGCAATTGAAGCGAAGACAGT

>42

CTTGTTTCAAAACTAAGTGTTACACGCCAGGTTGTTCATGTTCATATCCAGTTTGCAAGAGAAACCATATAATTGCAATTGAAGCGAAGACAGTGGATGA

>43

CTTGTTTCAAAACTAAGTGTTACACGCCAGGTTGTTCATGTTCATATCCAGTTTGCAAGAGAAACCATATAATTGCAATTGAAGCGAAGACAGTGGATGA

>44

GAAACTTGTTTCAAAACTAAGTGTTACACGCCAGGTTGTTCATGTTCATATCCAGTTTGCAAGAGAAACCATATAATTGCAATTGAAGCGAAGACAGTGG

>45

CCTATCTGTGGAGAAACTTGTTTCAAAACTAAGTGTTACACGCCAGGTTGTTCATGTTCATATCCAGTTTGCAAGAGAAACCATATAATTGCAATTGAAG

>46

AGACAGAAGGGGGTTTTCCTATCTGTGGAGAAACTTGTTTCAAAACTAAGTGTTACACGCCAGGTTGTTCATGTTCATATCCAGTTTGCAAGAGAAACCA

>47

AGAAACTTGTTTCAAAACTAAGTGTTACACGCCAGGTTGTTCATGTTCATATCCAGTTTGCAAGAGAAACCATATAATTGCAATTGAAGCGAAGACAGTG

>48

AGGGGGTTTTCCTATCTGTGGAGAAACTTGTTTCAAAACTAAGTGTTACACGCCAGGTTGTTCATGTTCATATCCAGTTTGCAAGAGAAACCATATAATT

>49

TTCAAAACTAAGTGTTACACGCCAGGTTGTTCATGTTCATATCCAGTTTGCAAGAGAAACCATATAATTGCAATTGAAGCGAAGACAGTGGATGAACATC

>50

CTTGTTTCAAAACTAAGTGTTACACGCCAGGTTGTTCATGTTCATATCCAGTTTGCAAGAGAAACCATATAATTGCAATTGAAGCGAAGACAGTGGATGA

>51

GGTTTTCCTATCTGTGGAGAAACTTGTTTCAAAACTAAGTGTTACACGCCAGGTTGTTCATGTTCATATCCAGTTTGCAAGAGAAACCATATAATTGCAA

**CDS to Protein Translation (only cyclotide-like transcripts):**

>1_1

FKTKCYTPGCSCSYPVCKRNHIIAIEAKTVDEHX

>2_1

PICGETCFKTKCYTPGCSCSYPVCKRNHIIAIEX

>3_3

CFKTKCYTPGCSCSYPVCKRNHIIAIEAKTVDR

>4_1

EGGFPIWGETCFKTKCYTPGCSCSYPVCKRNYIX

>5_2

GFPICGETCFKTKCYTPGCSCSYPVCKRNHIIA

>6_2

KTEGGFPICGETCFKTKCYTPGCSCSYPVCKRN

>7_3

CFKTKCYTPGCSCSYPVCKRNHIIAIEAKTVDX

>8_2

GGFPICGETCFKTKCYTPGCSCSYPVCKRNHII

>9_2

GGFPICGETCFKTKCYTPGCSCSYPVCKRNHII

>10_3

CFKTKCYTPGCSCSYPVCKRNHIIAIEAKTVDX

>11_1

ICGETCFKTKCYTPGCSCSYPVCKRNHVIAIETX

>12_2

KTEGGFPICGETCFKTKCYTPGCSCSYPVCKRN

>13_3

CFKTKCYTPGCSCSYPVCKRNHIIAIEAKTVDX

>14_3

CFKTKCYTPGCSCSYPVCKRNHIIAIEEKTVDX

>15_3

EGGFPICGETCFKTKCYTPGCSCSYPVCKRNHX

>16_1

PIC*ETCFKTKCYTPGCSCSYPVCKRNHIIAIEX

>17_2

GGFPICGETCFKTKCYTPGCSCSYPVCKRNHII

>18_2

FKTKCYTPGCSCSYPVCKRNHIIAIEAKTVDEH

>19_3

ETCFKTKCYTPGCSCSYPVCKRNHIIAIEAKTV

>20_1

GETCFKTKCYTPGCSCSYPVCKRNHIIAIEAKIX

>21_3

CFKTKCYTPGCSCSYPVCKRNHIIAIEAKTVDX

>22_3

EGGFPICGETCFKTKCYTPGCSCSYPVCKRNHX

>23_3

GFPICGETCFKTKCYTPGCSCSYPVCKRNHIIA

>24_3

CFKTKCYTPGCSCSYPVCKRNHIIAIEAKRSEX

>25_3

CFKTKCYTPGCSCSYPVCKRNHIIAIEAKTVDX

>26_3

CFKTKCYTPGCSCSYPVCKRNHIIAIEAKTVDX

>27_3

CFKTKCYTPGCSCSYPVCKRNHIIAIEAKTVDX

>28_2

TEGGFPICGETCFKTKCYTPGCSCSYPVCKRNH

>29_3

CFKTKCYTPGCSCSYPVCKRNHIIAIEAKTVDX

>30_1

FKTKCYTPGCSCSYPVCKRNHIIAIEAKTVDEHX

>31_1

GGFPICGETCFKTKCYTPGCSCSYPVCKRNHIIX

>32_1

ETCFKTKCYTPGCSCSYPVCKRNHIIAIEAKTVX

>33_3

GFPICGETCFKTKCYTPGCSCSYPVCKRNHIIA

>34_1

ETCFKTKCYTPGCSCSYPVCKRNHIIAIEAKTVX

>35_1

FKTKCYTPGCSCSYPVCKRNHIIAIEAKTVDEHX

>36_2

GGFPICGETCFKTKCYTPGCSCSYPVCKRNHII

>37_1

GGFPICGETCFKTKCYTPGCSCSYPVCKRN*IGX

>38_1

PICGETCFKTKCYTPGCSCSYPVCKRNHIIAIEX

>39_3

CFKTKCYTPGCSCSYPVCKRNHIIAIEAKTVDX

>40_2

TCFKTKCYTPGCSCSYPVCKRNHIIAIEAKTVD

>41_3

ETCFKTKCYTPGCSCSYPVCKRNHIIAIEAKTV

>42_3

CFKTKCYTPGCSCSYPVCKRNHIIAIEAKTVDX

>43_3

CFKTKCYTPGCSCSYPVCKRNHIIAIEAKTVDX

>44_1

ETCFKTKCYTPGCSCSYPVCKRNHIIAIEAKTVX

>45_1

PICGETCFKTKCYTPGCSCSYPVCKRNHIIAIEX

>46_3

TEGGFPICGETCFKTKCYTPGCSCSYPVCKRNX

>47_2

ETCFKTKCYTPGCSCSYPVCKRNHIIAIEAKTV

>48_2

GGFPICGETCFKTKCYTPGCSCSYPVCKRNHII

>49_1

FKTKCYTPGCSCSYPVCKRNHIIAIEAKTVDEHX

>50_3

CFKTKCYTPGCSCSYPVCKRNHIIAIEAKTVDX

>51_1

GFPICGETCFKTKCYTPGCSCSYPVCKRNHIIAX

**Multiple sequence alignment:**

4_1 --EG**GFPIWGETCFKTKCYTPGCSCSYPVCKRN**YIX----------- 34

15_3 --EG**GFPICGETCFKTKCYTPGCSCSYPVCKRN**HX------------ 33

22_3 --EG**GFPICGETCFKTKCYTPGCSCSYPVCKRN**HX------------ 33

6_2 KTEG**GFPICGETCFKTKCYTPGCSCSYPVCKRN**-------------- 33

12_2 KTEG**GFPICGETCFKTKCYTPGCSCSYPVCKRN**-------------- 33

28_2 -TEG**GFPICGETCFKTKCYTPGCSCSYPVCKRN**H------------- 33

46_3 -TEG**GFPICGETCFKTKCYTPGCSCSYPVCKRN**X------------- 33

37_1 ---G**GFPICGETCFKTKCYTPGCSCSYPVCKRN***IGX---------- 33

5_2 ----**GFPICGETCFKTKCYTPGCSCSYPVCKRN**HIIA---------- 33

23_3 ----**GFPICGETCFKTKCYTPGCSCSYPVCKRN**HIIA---------- 33

33_3 ----**GFPICGETCFKTKCYTPGCSCSYPVCKRN**HIIA---------- 33

51_1 ----**GFPICGETCFKTKCYTPGCSCSYPVCKRN**HIIAX--------- 34

8_2 ---G**GFPICGETCFKTKCYTPGCSCSYPVCKRN**HII----------- 33

9_2 ---G**GFPICGETCFKTKCYTPGCSCSYPVCKRN**HII----------- 33

17_2 ---G**GFPICGETCFKTKCYTPGCSCSYPVCKRN**HII----------- 33

31_1 ---G**GFPICGETCFKTKCYTPGCSCSYPVCKRN**HIIX---------- 34

36_2 ---G**GFPICGETCFKTKCYTPGCSCSYPVCKRN**HII----------- 33

48_2 ---G**GFPICGETCFKTKCYTPGCSCSYPVCKRN**HII----------- 33

11_1 -------**ICGETCFKTKCYTPGCSCSYPVCKRN**HVIAIETX------ 34

2_1 ------**PICGETCFKTKCYTPGCSCSYPVCKRN**HIIAIEX------- 34

38_1 ------**PICGETCFKTKCYTPGCSCSYPVCKRN**HIIAIEX------- 34

45_1 ------**PICGETCFKTKCYTPGCSCSYPVCKRN**HIIAIEX------- 34

16_1 ------**PIC*****ETCFKTKCYTPGCSCSYPVCKRN**HIIAIEX------- 33

24_3 ------------**CFKTKCYTPGCSCSYPVCKRN**HIIAIEAKRSEX-- 33

20_1 ---------**GETCFKTKCYTPGCSCSYPVCKRN**HIIAIEAKIX---- 34

1_1 -------------**FKTKCYTPGCSCSYPVCKRN**HIIAIEAKTVDEHX 34

18_2 -------------**FKTKCYTPGCSCSYPVCKRN**HIIAIEAKTVDEH- 33

30_1 -------------**FKTKCYTPGCSCSYPVCKRN**HIIAIEAKTVDEHX 34

35_1 -------------**FKTKCYTPGCSCSYPVCKRN**HIIAIEAKTVDEHX 34

49_1 -------------**FKTKCYTPGCSCSYPVCKRN**HIIAIEAKTVDEHX 34

19_3 ----------**ETCFKTKCYTPGCSCSYPVCKRN**HIIAIEAKTV---- 33

32_1 ----------**ETCFKTKCYTPGCSCSYPVCKRN**HIIAIEAKTVX--- 34

34_1 ----------**ETCFKTKCYTPGCSCSYPVCKRN**HIIAIEAKTVX--- 34

41_3 ----------**ETCFKTKCYTPGCSCSYPVCKRN**HIIAIEAKTV---- 33

44_1 ----------**ETCFKTKCYTPGCSCSYPVCKRN**HIIAIEAKTVX--- 34

47_2 ----------**ETCFKTKCYTPGCSCSYPVCKRN**HIIAIEAKTV---- 33

14_3 ------------**CFKTKCYTPGCSCSYPVCKRN**HIIAIEEKTVDX-- 33

3_3 ------------**CFKTKCYTPGCSCSYPVCKRN**HIIAIEAKTVDR-- 33

7_3 ------------**CFKTKCYTPGCSCSYPVCKRN**HIIAIEAKTVDX-- 33

10_3 ------------**CFKTKCYTPGCSCSYPVCKRN**HIIAIEAKTVDX-- 33

13_3 ------------**CFKTKCYTPGCSCSYPVCKRN**HIIAIEAKTVDX-- 33

21_3 ------------**CFKTKCYTPGCSCSYPVCKRN**HIIAIEAKTVDX-- 33

25_3 ------------**CFKTKCYTPGCSCSYPVCKRN**HIIAIEAKTVDX-- 33

26_3 ------------**CFKTKCYTPGCSCSYPVCKRN**HIIAIEAKTVDX-- 33

27_3 ------------**CFKTKCYTPGCSCSYPVCKRN**HIIAIEAKTVDX-- 33

29_3 ------------**CFKTKCYTPGCSCSYPVCKRN**HIIAIEAKTVDX-- 33

39_3 ------------**CFKTKCYTPGCSCSYPVCKRN**HIIAIEAKTVDX-- 33

42_3 ------------**CFKTKCYTPGCSCSYPVCKRN**HIIAIEAKTVDX-- 33

43_3 ------------**CFKTKCYTPGCSCSYPVCKRN**HIIAIEAKTVDX-- 33

50_3 ------------**CFKTKCYTPGCSCSYPVCKRN**HIIAIEAKTVDX-- 33

40_2 -----------**TCFKTKCYTPGCSCSYPVCKRN**HIIAIEAKTVD--- 33

********************

**Possible sequence: GFPICGETCFKTKCYTPGCSCSYPVCKRN**

**2. Clitoria ternatea cyclotide precursor, 5prime_partial, ctr28841_c1_g1_i4, 366-641(-)**

**Query string:** TCTTGTGTATGGATTCCATGTATAACCGGAGCTATCGGTTGTTCTTGCAAAAATAGAGTC

**Reads:**

>1

CTTGTGGAGAATCTTGTGTATGGATTCCATGTATAACCGGAGCTATCGGTTGTTCTTGCAAAAATAGAGTCTGCTACCGAAATCATGTCATTGCATCTGA

>2

CTGAAGCCGGTATTCCTTGTGGAGAATCTTGTGTATGGATTCCATGTATAACCGGAGCTATCGGTTGTTCTTGCAAAAATAGAGTCTGCTACCGAAATCA

>3

ATCTTGTGTATGGATTCCATGTATAACCGGAGCTATCGGTTGTTCTTGCAAAAATAGAGTCTGCTACCGAAATCATGTCATTGCATCTGAGGCAAAGACA

>4

CCGGTATTCCTTGTGGAGAATCTTGTGTATGGATTCCATGTATAACCGGAGCTATCGGTTGTTCTTGCAAAAATAGAGTCTGCTACCGAAATCATGTCAT

>5

AGCCGGTATTCCTTGTGGAGAATCTTGTGTATGGATTCCATGTATAACCGGAGCTATCGGTTGTTCTTGCAAAAATAGAGTCTGCTACCGAAATCATGTC

>6

CTGAAGCCGGTATTCCTTGTGGAGAATCTTGTGTATGGATTCCATGTATAACCGGAGCTATCGGTTGTTCTTGCAAAAATAGAGTCTGCTACCGAAATCA

>7

CTGAAGCCGGTATTCCTTGTGGAGAATCTTGTGTATGGATTCCATGTATAACCGGAGCTATCGGTTGTTCTTGCAAAAATAGAGTCTGCTACCGATAGAT

>8

CTCGTGGAGAATCTTGTGTATGGATTCCATGTATAACCGGAGCTATCGGTTGTTCTTGCAAAAATAGAGTCTGCTACCGAAATCATATCATTGCATCTGA

>9

AGAATCTTGTGTATGGATTCCATGTATAACCGGAGCTATCGGTTGTTCTTGCAAAAATAGAGTCTGCTACCGAAATCATGTCATTGCATCTGAGGCAAAG

>10

GAGAATCTTGTGTATGGATTCCATGTATAACCGGAGCTATCGGTTGTTCTTGCAAAAATAGAGTCTGCTACCGAAATCATGTCATTGCATCTGAGGCAAA

>11

CTTTTGGAGAATCTTGTGTATGGATTCCATGTATAACCGGAGCTATCGGTTGTTCTTGCAAAAATAGAGTCTGCTACCGAAATCATGTCATTGCATCTGA

>12

CTGAAGCCGGTATTCCTTGTGGAGAATCTTGTGTATGGATTCCATGTATAACCGGAGCTATCGGTTGTTCTTGCAAAAATAGAGTCTGCTACCGAAATCA

>13

GGTATTCCTTGTGGAGAATCTTGTGTATGGATTCCATGTATAACCGGAGCTATCGGTTGTTCTTGCAAAAATAGAGTCTGCTACCGAAATCAAGATCGGA

>14

GGTATTCCTTGTGGAGAATCTTGTGTATGGATTCCATGTATAACCGGAGCTATCGGTTGTTCTTGCAAAAATAGAGTCTGCTACCGAAATCATGTCATTG

>15

CTGAAGCCGGTATTCCTTGTGGAGAATCTTGTGTATGGATTCCATGTATAACCGGAGCTATCGGTTGTTCTTGCAAAAATAGAGTCTGCTACCGAAATCA

**CDS to Protein Translation (only cyclotide-like transcripts):**

>1_3

CGESCVWIPCITGAIGCSCKNRVCYRNHVIASX

>2_3

EAGIPCGESCVWIPCITGAIGCSCKNRVCYRNX

>4_3

GIPCGESCVWIPCITGAIGCSCKNRVCYRNHVX

>5_2

AGIPCGESCVWIPCITGAIGCSCKNRVCYRNHV

>6_3

EAGIPCGESCVWIPCITGAIGCSCKNRVCYRNX

>7_3

EAGIPCGESCVWIPCITGAIGCSCKNRVCYR*X

>8_3

RGESCVWIPCITGAIGCSCKNRVCYRNHIIASX

>9_2

ESCVWIPCITGAIGCSCKNRVCYRNHVIASEAK

>10_3

ESCVWIPCITGAIGCSCKNRVCYRNHVIASEAX

>11_3

FGESCVWIPCITGAIGCSCKNRVCYRNHVIASX

>12_3

EAGIPCGESCVWIPCITGAIGCSCKNRVCYRNX

>13_1

GIPCGESCVWIPCITGAIGCSCKNRVCYRNQDRX

>14_1

GIPCGESCVWIPCITGAIGCSCKNRVCYRNHVIX

>15_3

EAGIPCGESCVWIPCITGAIGCSCKNRVCYRNX

**Multiple sequence alignment:**

13_1 --GIPCGE**SCVWIPCITGAIGCSCKNRVCYRN**QDRX---- 34

2_3 EAGIPCGE**SCVWIPCITGAIGCSCKNRVCYRN**X------- 33

6_3 EAGIPCGE**SCVWIPCITGAIGCSCKNRVCYRN**X------- 33

12_3 EAGIPCGE**SCVWIPCITGAIGCSCKNRVCYRN**X------- 33

15_3 EAGIPCGE**SCVWIPCITGAIGCSCKNRVCYRN**X------- 33

7_3 EAGIPCGE**SCVWIPCITGAIGCSCKNRVCYR***X------- 32

4_3 --GIPCGE**SCVWIPCITGAIGCSCKNRVCYRN**HVX----- 33

5_2 -AGIPCGE**SCVWIPCITGAIGCSCKNRVCYRN**HV------ 33

14_1 --GIPCGE**SCVWIPCITGAIGCSCKNRVCYRN**HVIX---- 34

9_2 -------E**SCVWIPCITGAIGCSCKNRVCYRN**HVIASEAK 33

10_3 -------E**SCVWIPCITGAIGCSCKNRVCYRN**HVIASEAX 33

1_3 -----CGE**SCVWIPCITGAIGCSCKNRVCYRN**HVIASX-- 33

11_3 -----FGE**SCVWIPCITGAIGCSCKNRVCYRN**HVIASX-- 33

8_3 -----RGE**SCVWIPCITGAIGCSCKNRVCYRN**HIIASX-- 33

**Possible sequence: GIPCGESCVWIPCITGAIGCSCKNRVCYRN**

**3. >Clitoria ternatea cyclotide precursor, internal, ctr28841_c1_g4_i1, 3-266(-)**

**Query string:** GAATCTTGTGTATGGATTCCATGTCTCACGGGATATTTTGGCTGTTATTGTCAAAGCAAA

**Reads:**

>1

GTTTGTGGCAAAGAAGACGGAAGCTAAAATTCCTTGCGGAGAATCTTGTGTATGGATTCCATGTCTCACGGGATATTTTGGCTGTTATTGTCAAAGCAAA

>2

CGGAAGCTAAAATTCCTTGCGGAGAATCTTGTGTATGGATTCCATGTCTCACGGGATATTTTGGCTGTTATTGTCAAAGCAAAGTTTGTTATCATAACTC

>3

AAAATTCCTTGCGGAGAATCTTGTGTATGGATTCCATGTCTCACGGGATATTTTGGCTGTTATTGTCAAAGCAAAGTTTGTTATCATAACTCTCATATTG

>4

TCCTTGCGGAGAATCTTGTGTATGGATTCCATGTCTCACGGGATATTTTGGCTGTTATTGTCAAAGCAAAGTTTGTTATCATAACTCTCATATTGCATCA

>5

GGCAAAGAAGACGGAAGCTAAAATTCCTTGCGGAGAATCTTGTGTATGGATTCCATGTCTCACGGGATATTTTGGCTGTTATTGTCAAAGCAAAGTTTGT

>6

GGAGAATCTTGTGTATGGATTCCATGTCTCACGGGATATTTTGGCTGTTATTGTCAAAGCAAAGTTTGTTATCATAACTCTCATATTGCATCAACTGCAA

>7

AAGAAGACGGAAGCTAAAATTCCTTGCGGAGAATCTTGTGTATGGATTCCATGTCTCACGGGATATTTTGGCTGTTATTGTCAAAGCAAAGTTTGTTATC

>8

CAAAGAAGACGGAAGCTAAAATTCCTTGCGGAGAATCTTGTGTATGGATTCCATGTCTCACGGGATATTTTGGCTGTTATTGTCAAAGCAAAGTTTGTTA

>9

AGAAGACGGAAGCTAAAATTCCTTGCGGAGAATCTTGTGTATGGATTCCATGTCTCACGGGATATTTTGGCTGTTATTGTCAAAGCAAAGTTTGTTATCA

>10

GGAAGCTAAAATTCCTTGCGGAGAATCTTGTGTATGGATTCCATGTCTCACGGGATATTTTGGCTGTTATTGTCAAAGCAAAGTTTGTTATCATAACTCT

>11

TAAAATTCCTTGCGGAGAATCTTGTGTATGGATTCCATGTCTCACGGGATATTTTGGCTGTTATTGTCAAAGCAAAGTTTGTTATCATAACTCTCATATT

>12

GCTAAAATTCCTTGCGGAGAATCTTGTGTATGGATTCCATGTCTCACGGGATATTTTGGCTGTTATTGTCAAAGCAAAGTTTGTTATCATAACTCTCATA

>13

GCAAAGAAGACGGAAGCTAAAATTCCTTGCGGAGAATCTTGTGTATGGATTCCATGTCTCACGGGATATTTTGGCTGTTATTGTCAAAGCAAAGTTTGTT

>14

GAGAATCTTGTGTATGGATTCCATGTCTCACGGGATATTTTGGCTGTTATTGTCAAAGCAAAGTTTGTTATCATAACTCTCATATTGCATCAACTGCAAA

>15

CGGAAGCTAAAATTCCTTGCGGAGAATCTTGTGTATGGATTCCATGTCTCACGGGATATTTTGGCTGTTATTGTCAAAGCAAAGTTTCTTATCATAACTC

>16

AAAGAAGACGGAAGCTAAAATTCCTTGCGGAGAATCTTGTGTATGGATTCCATGTCTCACGGGATATTTTGGCTGTTATTGTCAAAGCAAAGTTTGTTAT

>17

AGAATCTTGTGTATGGATTCCATGTCTCACGGGATATTTTGGCTGTTATTGTCAAAGCAAAGTTTGTTATCATAACTCTCATATTGCATCAACTGCAAAG

>18

CTAAAATTCCTTGCGGAGAATCTTGTGTATGGATTCCATGTCTCACGGGATATTTTGGCTGTTATTGTCAAAGCAAAGTTTGTTATCATAACTCTCATAT

>19

AGACGGAAACTAAAATTCCTTGCGGAGAATCTTGTGTATGGATTCCATGTCTCACGGGATATTTTGGCTGTTATTGTCAAAGCAAAGTTTGTTATCATAA

>20

GAAGACGGAAGCTAAAATTCCTTGCGGAGAATCTTGTGTATGGATTCCATGTCTCACGGGATATTTTGGCTGTTATTGTCAAAGCAAAGTTTGTTATCAT

>21

ATTCCTTGCGGAGAATCTTGTGTATGGATTCCATGTCTCACGGGATATTTTGGCTGTTATTGTCAAAGCAAAGTTTGTTATCATAACTCTCATATTGCAT

>22

GACGGAAGCTAAAATTCCTTGCGGAGAATCTTGTGTATGGATTCCATGTCTCACGGGATATTTTGGCTGTTATTGTCAAAGCAAAGTTTGTTATCATAAC

>23

TGCGGAGAATCTTGTGTATGGATTCCATGTCTCACGGGATATTTTGGCTGTTATTGTCAAAGCAAAGTTTGTTATCATAACTCTCATATTGCATCAACTG

>24

AGAAGACGGAATCTAAAATTCCTTGCGGAGAATCTTGTGTATGGATTCCATGTCTCACGGGATATTTTGGCTGTTATTGTCAAAGCAAAGTTTGTTATCA

>25

GTTTGTGGCAAAGAAGACGGAAGCTAAAATTCCTTGCGAAGAATCTTGTGTATGGATTCCATGTCTCACGGGATATTTTGGCTGTTATTGTCAAAGCAAA

>26

CGGAAGGTAAAATTCCTTGCGGAGAATCTTGTGTATGGATTCCATGTCTCACGGGATATTTTGGCTGTTATTGTCAAAGCAAAGTTTGTTATCATAACTC

>27

ACGGAAACTAAAATTTCTTGTGGAGAATCTTGTGTATGGATTCCATGTCTCACGGGATATTTTGGCTGTTATTGTCAAAGCAAAGTTTGTTATCATAACT

>28

CGGAAGCTAAAATTCCTTGCGGAGAATCTTGTGTATGGATTCCATGTCTCACGGGATATTTTGGCTGTTATTGTCAAAGCAAAGTTTGTTATCATAGATC

>29

CTTGCGGAGAATCTTGTGTATGGATTCCATGTCTCACGGGATATTTTGGCTGTTATTGTCAAAGCAAAGTTTGTTATCATAACTCTCATATTGCATCAAC

>30

AGCTAAAATTCCTTGCGGAGAATCTTGTGTATGGATTCCATGTCTCACGGGATATTTTGGCTGTTATTGTCAAAGCAAAGTTTGTTATCATAACTCTCAT

>31

AAGCTAAAATTCCTTGCGGAGAATCTTGTGTATGGATTCCATGTCTCACGGGATATTTTGGCTGTTATTGTCAAAGCAAAGTTTGTTATCATAACTCTCA

>32

TAAAATTCCTTACGGAGAATCTTGTGTATGGATTCCATGTCTCACGGGATATTTTGGCTGTTATTGTCAAAGCAAAGTTTGTTATCATAACTCTCATATT

>33

AATTCCTTGCGGAGAATCTTGTGTATGGATTCCATGTCTCACGGGATATTTTGGCTGTTATTGTCAAAGCAAAGTTTGTTATCATAACTCTAATATTGCA

>34

GAATCTTGTGTATGGATTCCATGTCTCACGGGATATTTTGGCTGTTATTGTCAAAGCAAAGTTTGTTATCATAACTCTCATATTGCATCAACTGCAAAGA

>35

AGACGGAAGCTAAAATTCCTTGCGGAGAATCTTGTGTATGGATTCCATGTCTCACGGGATATTTTGGCTGTTATTGTCAAAGCAAAGTTTGTTATCATAA

>36

GTGGCAAAGAAGACGGAAGCTAAAATTCCTTGCGGAGAATCTTGTGTATGGATTCCATGTCTCACGGGATATTTTGGCTGTTATTGTCAAAGCAAAGTTT

>37

TGGCAAAGAAGACGGAAGCTAAAATTCCTTGCGGAGAATCTTGTGTATGGATTCCATGTCTCACGGGATATTTTGGCTGTTATTGTCAAAGCAAAGTTTG

>38

AAATTCCTTGCGGAGAATCTTGTGTATGGATTCCATGTCTCACGGGATATTTTGGCTGTTATTGTCAAAGCAAAGTTTGTTATTATAACTCTCATATTGC

>39

CGGAGAATCTTGTGTATGGATTCCATGTCTCACGGGATATTTTGGCTGTTATTGTCAAAGCAAAGTTTGTTATCATAACTCTCATATTGCATCAACTGCA

>40

AATTCCTTGCGGAGAATCTTGTGTATGGATTCCATGTCTCACGGGATATTTTGGCTGTTATTGTCAAAGCAAAGTTTGTTATCATAACTCTCATATTGCA

>41

CGGAAGCTAAAATTCCTTGCGGAGAATCTTGTGTATGGATTCCATGTCTCACGGGATATTTTGGCTGTTATTGTCAAAGCAAAGGTTGTTATCATAGATC

>42

ACGGAAGCTAAAATTCCTTGCGGAGAATCTTGTGTATGGATTCCATGTCTCACGGGATATTTTGGCTGTTATTGTCAAAGCAAAGTTTGTTATCATAACT

>43

AGAAGACGGAAGCTAATTCCTTGCGGAGAATCTTGTGTATGGATTCCATGTCTCACGGGATATTTTGGCTGTTATTGTCAAAGCAAAGTTTGTTATCATA

>44

TTTGTGGCAAAGAAGACGGAAGCTAAAATTCCTTGCGGAGAATCTTGTGTATGGATTCCATGTCTCACGGGATATTTTGGCTGTTATTGTCAAAGCAAAG

>45

AAGACGGAAGCTAAAATTCCTTGCGGAGAATCTTGTGTATGGATTCCATGTCTCACGGGATATTTTGGCTGTTATTGTCAAAGCAAAGTTTGTTATCATA

>46

GGCAAAGAAGACGGAAGCTAAAATTCCTTGCGGAGAATCTTGTGTATGGATTCCATGTCTCACGGGATATTTTGGCTGTTATTGTCAAAGCAAAGAGATC

>47

TAAGCTAAAATTCCTTGCGGAGAATCTTGTGTATGGATTCCATGTCTCACGGGATATTTTGGCTGTTATTGTCAAAGCAAAGTTTGTTATCATAACTCTC

>48

CCTTGCGGAGAATCTTGTGTATGGATTCCATGTCTCACGGGATATTTTGGCTGTTATTGTCAAAGCAAAGTTTGTTATCATAACTCTCATATTGCATCAA

>49

ACGGAAGCTAAAATTTCTTGCGGAGAATCTTGTGTATGGATTCCATGTCTCACGGGATATTTTGGCTGTTATTGTCAAAGCAAAGTTTGTTATCATAACT

>50

GGAAGCTAAAATTCCTTGCGGAGAATCTTGTGTATGGATTCCATGTCTCACGGGATATTTTGGCTGTTATTGTCAAAGCAAAGTTTGTTTTCATAACTCT

>51

CGGAAGCTAAAATTCCTTGCGGAGAATCTTGTGTATGGATTCCATGTCTCACGGGATATTTTGGCTGTTATTGTCAAAGCAAAGTTTGTTATCCTAACTC

>52

AGAAGACGGAAACTAAAATTCCTTGCGGAGAATCTTGTGTATGGATTCCATGTCTCACGGGATATTTTGGCTGTTATTGTCAAAGCAAAGTTTGTTATCA

>53

GAAGACGGAAGCTAAAATTCCTTTCGGAGAATCTTGTGTATGGATTCCATGTCTCACGGGATATTTTGGCTGTTATTGTCAAAGCAAAGTTTGTTATCAT

>54

GTTTGTAGCAAAGAAGACGGAAGCTAAAATTCCTTGCGGAGAATCTTGTGTATGGATTCCATGTCTCACGGGATATTTTGGCTGTTATTGTCAAAGCAAA

>55

GACGGAAGCTAAAATTCCTTGCGGAGAATCTTGTGTATGGATTCCATGTCTCACGGGATATTTTGGCTGTTATTGTCAAAGCAAAGTTGTTATCATAACT

>56

AGAATCTTGTGTATGGATTCCATGTCTCACGGGATATTTTGGCTGTTATTGTCAAAGCAAACTTTGTTATCATAACTCTCATATTGCATCAACTGCAAAG

>57

GAAGCTAAAATTCCTTGCGGAGAATCTTGTGTATGGATTCCATGTCTCACGGGATATTTTGGCTGTTATTGTCAAAGCAAAGTTTGTTATCATAACTCTC

>58

AAAAAAGACGGAAGCTAAAATTCCTTGCGGAGAATCTTGTGTATGGATTCCATGTCTCACGGGATATTTTGGCTGTTATTGTCAAAGCAAAGTTTGTTAT

>59

GAAGACGGAAGCTAAAATTCCTTGCGGAGAATCTTGTGTATGGATTCCATGTCTCACGGGATATTTTGGCTGTTATTGTCAAAGCAAAGAGATCGGAAGA

>60

AGAAGACGGAAGCTAAAATTCATTGCGGAGAATCTTGTGTATGGATTCCATGTCTCACGGGATATTTTGGCTGTTATTGTCAAAGCAAAGTTTGTTATCA

>61

AAATTCCTTGCGGAGAATCTTGTGTATGGATTCCATGTCTCACGGGATATTTTGGCTGTTATTGTCAAAGCAAAGTTTGTTATCATAACTCTCATATTGC

>62

AGAAGAAGGAAGCTAAAATTCCTTGCGGAGAATCTTGTGTATGGATTCCATGTCTCACGGGATATTTTGGCTGTTATTGTCAAAGCAAAGTTTGTTATCA

>63

TTGTGGCAAAGAAGACGGAAGCTAAAATTCCTTGCGGAGAATCTTGTGTATGGATTCCATGTCTCACGGGATATTTTGGCTGTTATTGTCAAAGCAAAGT

>64

TGCGGAGAATCTTGTGTATGGATTCCATGTCTCACGGGATATTTTGGCTGTTATTGTCAAAGCAAAGTTTGTTATCATAACTATCATATTGTATCAACTG

>65

GACGGAAGCTAAAATTCCTTGCGGAGAATCTTGTGTATGGATTCCATGTCTCACGGGATATTTTGGCTGTTATTGTCAAAGCAAAGATTGTTATCATAAC

>66

GGAGGCTAAAATTCCTTGCGGAGAATCTTGTGTATGGATTCCATGTCTCACGGGATATTTTGGCTGTTATTGTCAAAGCAAAGTTTGTTATCATAACTCT

>67

GGCAAAGAAGACGGACGCTAGAATTCCTTGCGGAGAATCTTGTGTATGGATTCCATGTCTCACGGGATATTTTGGCTGTTATTGTCAAAGCAAAGTTTGT

>68

GAGAATCTTGTGTATGGATTCCATGTCTCACGGGATATTTTGGCTGTTATTGTCAAAGCAAAGTTTGTTATCATCACTCTCATATTGCATCAACTGCAAA

>69

GAATCTTGTGTATGGATTCCATGTCTCACGGGATATTTTGGCTGTTATTGTCAAAGCAAAGTTTGTTAACATAACTCTCATATTGCATCAACTGCAAAGA

>70

GTTTGTGGCAAAGGAGACGGAAGCTAAAATTCCTTGCGGAGAATCTTGTGTATGGATTCCATGTCTCACGGGATATTTTGGCTGTTATTGTCAAAGCAAA

>71

CTAAAATTCCTTGCGGAGAATCTTGTGTATGGATTCCATGTCTCACGGGATATTTTGGCTGTTATTGTCAAAGCAAAGTTTGTTATCATAACTCTAATAT

**CDS to Protein Translation (only cyclotide-like transcripts):**

>1_2

FVAKKTEAKIPCGESCVWIPCLTGYFGCYCQSK

>10_2

EAKIPCGESCVWIPCLTGYFGCYCQSKVCYHNS

>11_2

KIPCGESCVWIPCLTGYFGCYCQSKVCYHNSHI

>12_1

AKIPCGESCVWIPCLTGYFGCYCQSKVCYHNSHX

>13_1

AKKTEAKIPCGESCVWIPCLTGYFGCYCQSKVCX

>14_3

ESCVWIPCLTGYFGCYCQSKVCYHNSHIASTAX

>15_3

EAKIPCGESCVWIPCLTGYFGCYCQSKVSYHNS

>16_2

KKTEAKIPCGESCVWIPCLTGYFGCYCQSKVCY

>17_2

ESCVWIPCLTGYFGCYCQSKVCYHNSHIASTAK

>18_3

KIPCGESCVWIPCLTGYFGCYCQSKVCYHNSHX

>19_3

TETKIPCGESCVWIPCLTGYFGCYCQSKVCYHX

>2_3

EAKIPCGESCVWIPCLTGYFGCYCQSKVCYHNS

>20_2

KTEAKIPCGESCVWIPCLTGYFGCYCQSKVCYH

>21_1

IPCGESCVWIPCLTGYFGCYCQSKVCYHNSHIAX

>22_2

TEAKIPCGESCVWIPCLTGYFGCYCQSKVCYHN

>23_1

CGESCVWIPCLTGYFGCYCQSKVCYHNSHIASTX

>24_3

KTESKIPCGESCVWIPCLTGYFGCYCQSKVCYX

>25_2

FVAKKTEAKIPCEESCVWIPCLTGYFGCYCQSK

>26_3

EGKIPCGESCVWIPCLTGYFGCYCQSKVCYHNS

>27_1

TETKISCGESCVWIPCLTGYFGCYCQSKVCYHNX

>28_3

EAKIPCGESCVWIPCLTGYFGCYCQSKVCYHRS

>29_3

CGESCVWIPCLTGYFGCYCQSKVCYHNSHIAST

>3_1

KIPCGESCVWIPCLTGYFGCYCQSKVCYHNSHIX

>30_2

AKIPCGESCVWIPCLTGYFGCYCQSKVCYHNSH

>31_3

AKIPCGESCVWIPCLTGYFGCYCQSKVCYHNSX

>32_2

KIPYGESCVWIPCLTGYFGCYCQSKVCYHNSHI

>33_2

IPCGESCVWIPCLTGYFGCYCQSKVCYHNSNIA

>34_1

ESCVWIPCLTGYFGCYCQSKVCYHNSHIASTAKX

>35_3

TEAKIPCGESCVWIPCLTGYFGCYCQSKVCYHX

>36_1

VAKKTEAKIPCGESCVWIPCLTGYFGCYCQSKVX

>37_3

AKKTEAKIPCGESCVWIPCLTGYFGCYCQSKVX

>38_3

IPCGESCVWIPCLTGYFGCYCQSKVCYYNSHIA

>39_2

GESCVWIPCLTGYFGCYCQSKVCYHNSHIASTA

>4_2

PCGESCVWIPCLTGYFGCYCQSKVCYHNSHIAS

>40_2

IPCGESCVWIPCLTGYFGCYCQSKVCYHNSHIA

>41_3

EAKIPCGESCVWIPCLTGYFGCYCQSKGCYHRS

>42_1

TEAKIPCGESCVWIPCLTGYFGCYCQSKVCYHNX

>43_1

RRRKLIPCGESCVWIPCLTGYFGCYCQSKVCYHX

>44_1

FVAKKTEAKIPCGESCVWIPCLTGYFGCYCQSKX

>45_1

KTEAKIPCGESCVWIPCLTGYFGCYCQSKVCYHX

>46_2

AKKTEAKIPCGESCVWIPCLTGYFGCYCQSKEI

>47_1

*AKIPCGESCVWIPCLTGYFGCYCQSKVCYHNSX

>48_1

PCGESCVWIPCLTGYFGCYCQSKVCYHNSHIASX

>49_1

TEAKISCGESCVWIPCLTGYFGCYCQSKVCYHNX

>5_2

AKKTEAKIPCGESCVWIPCLTGYFGCYCQSKVC

>50_2

EAKIPCGESCVWIPCLTGYFGCYCQSKVCFHNS

>51_3

EAKIPCGESCVWIPCLTGYFGCYCQSKVCYPNS

>52_3

KTETKIPCGESCVWIPCLTGYFGCYCQSKVCYX

>53_2

KTEAKIPFGESCVWIPCLTGYFGCYCQSKVCYH

>54_2

FVAKKTEAKIPCGESCVWIPCLTGYFGCYCQSK

>55_2

TEAKIPCGESCVWIPCLTGYFGCYCQSKVVIIT

>56_2

ESCVWIPCLTGYFGCYCQSKLCYHNSHIASTAK

>57_1

EAKIPCGESCVWIPCLTGYFGCYCQSKVCYHNSX

>58_2

KKTEAKIPCGESCVWIPCLTGYFGCYCQSKVCY

>59_2

KTEAKIPCGESCVWIPCLTGYFGCYCQSKEIGR

>6_1

GESCVWIPCLTGYFGCYCQSKVCYHNSHIASTAX

>60_3

KTEAKIHCGESCVWIPCLTGYFGCYCQSKVCYX

>61_3

IPCGESCVWIPCLTGYFGCYCQSKVCYHNSHIA

>62_3

KKEAKIPCGESCVWIPCLTGYFGCYCQSKVCYX

>63_3

VAKKTEAKIPCGESCVWIPCLTGYFGCYCQSKV

>64_1

CGESCVWIPCLTGYFGCYCQSKVCYHNYHIVSTX

>65_2

TEAKIPCGESCVWIPCLTGYFGCYCQSKDCYHN

>66_2

EAKIPCGESCVWIPCLTGYFGCYCQSKVCYHNS

>67_2

AKKTDARIPCGESCVWIPCLTGYFGCYCQSKVC

>68_3

ESCVWIPCLTGYFGCYCQSKVCYHHSHIASTAX

>69_1

ESCVWIPCLTGYFGCYCQSKVC*HNSHIASTAKX

>7_1

KKTEAKIPCGESCVWIPCLTGYFGCYCQSKVCYX

>70_2

FVAKETEAKIPCGESCVWIPCLTGYFGCYCQSK

>71_3

KIPCGESCVWIPCLTGYFGCYCQSKVCYHNSNX

>8_3

KKTEAKIPCGESCVWIPCLTGYFGCYCQSKVCX

>9_3

KTEAKIPCGESCVWIPCLTGYFGCYCQSKVCYX

**Multiple sequence alignment:**

55_2 -----TEA**KIPCGESCVWIPCLTGYFGCYCQSKVVIIT**--------- 33

59_2 ----KTEA**KIPCGESCVWIPCLTGYFGCYCQSKEIGR**---------- 33

46_2 --AKKTEA**KIPCGESCVWIPCLTGYFGCYCQSKEI**------------ 33

70_2 FVAKETEA**KIPCGESCVWIPCLTGYFGCYCQSK**-------------- 33

1_2 FVAKKTEA**KIPCGESCVWIPCLTGYFGCYCQSK**-------------- 33

44_1 FVAKKTEA**KIPCGESCVWIPCLTGYFGCYCQSKX**------------- 34

54_2 FVAKKTEA**KIPCGESCVWIPCLTGYFGCYCQSK**-------------- 33

25_2 FVAKKTEA**KIPCEESCVWIPCLTGYFGCYCQSK**-------------- 33

67_2 --AKKTDA**RIPCGESCVWIPCLTGYFGCYCQSKVC**------------ 33

63_3 -VAKKTEA**KIPCGESCVWIPCLTGYFGCYCQSKV**------------- 33

36_1 -VAKKTEA**KIPCGESCVWIPCLTGYFGCYCQSKVX**------------ 34

37_3 --AKKTEA**KIPCGESCVWIPCLTGYFGCYCQSKVX**------------ 33

8_3 ---KKTEA**KIPCGESCVWIPCLTGYFGCYCQSKVCX**----------- 33

13_1 --AKKTEA**KIPCGESCVWIPCLTGYFGCYCQSKVCX**----------- 34

5_2 --AKKTEA**KIPCGESCVWIPCLTGYFGCYCQSKVC**------------ 33

56_2 -------------**ESCVWIPCLTGYFGCYCQSKLCYHN**SHIASTAK- 33

69_1 -------------**ESCVWIPCLTGYFGCYCQSKVC*****HN**SHIASTAKX 33

17_2 -------------**ESCVWIPCLTGYFGCYCQSKVCYHN**SHIASTAK- 33

34_1 -------------**ESCVWIPCLTGYFGCYCQSKVCYHN**SHIASTAKX 34

64_1 -----------**CGESCVWIPCLTGYFGCYCQSKVCYHN**YHIVSTX-- 34

68_3 -------------**ESCVWIPCLTGYFGCYCQSKVCYHH**SHIASTAX- 33

23_1 -----------**CGESCVWIPCLTGYFGCYCQSKVCYHN**SHIASTX-- 34

29_3 -----------**CGESCVWIPCLTGYFGCYCQSKVCYHN**SHIAST--- 33

39_2 ------------**GESCVWIPCLTGYFGCYCQSKVCYHN**SHIASTA-- 33

14_3 -------------**ESCVWIPCLTGYFGCYCQSKVCYHN**SHIASTAX- 33

6_1 ------------**GESCVWIPCLTGYFGCYCQSKVCYHN**SHIASTAX- 34

43_1 ----RRRK**LIPCGESCVWIPCLTGYFGCYCQSKVCYHX**--------- 34

51_3 ------EA**KIPCGESCVWIPCLTGYFGCYCQSKVCYPN**S-------- 33

50_2 ------EA**KIPCGESCVWIPCLTGYFGCYCQSKVCFHN**S-------- 33

41_3 ------EA**KIPCGESCVWIPCLTGYFGCYCQSKGCYHR**S-------- 33

28_3 ------EA**KIPCGESCVWIPCLTGYFGCYCQSKVCYHR**S-------- 33

26_3 ------EG**KIPCGESCVWIPCLTGYFGCYCQSKVCYHN**S-------- 33

15_3 ------EA**KIPCGESCVWIPCLTGYFGCYCQSKVSYHN**S-------- 33

10_2 ------EA**KIPCGESCVWIPCLTGYFGCYCQSKVCYHN**S-------- 33

2_3 ------EA**KIPCGESCVWIPCLTGYFGCYCQSKVCYHN**S-------- 33

57_1 ------EA**KIPCGESCVWIPCLTGYFGCYCQSKVCYHN**SX------- 34

66_2 ------EA**KIPCGESCVWIPCLTGYFGCYCQSKVCYHN**S-------- 33

31_3 -------A**KIPCGESCVWIPCLTGYFGCYCQSKVCYHN**SX------- 33

47_1 ------*A**KIPCGESCVWIPCLTGYFGCYCQSKVCYHN**SX------- 33

65_2 -----TEA**KIPCGESCVWIPCLTGYFGCYCQSKDCYHN**--------- 33

22_2 -----TEA**KIPCGESCVWIPCLTGYFGCYCQSKVCYHN**--------- 33

42_1 -----TEA**KIPCGESCVWIPCLTGYFGCYCQSKVCYHN**X-------- 34

27_1 -----TET**KISCGESCVWIPCLTGYFGCYCQSKVCYHN**X-------- 34

49_1 -----TEA**KISCGESCVWIPCLTGYFGCYCQSKVCYHN**X-------- 34

53_2 ----KTEA**KIPFGESCVWIPCLTGYFGCYCQSKVCYH**---------- 33

19_3 -----TET**KIPCGESCVWIPCLTGYFGCYCQSKVCYHX**--------- 33

35_3 -----TEA**KIPCGESCVWIPCLTGYFGCYCQSKVCYHX**--------- 33

20_2 ----KTEA**KIPCGESCVWIPCLTGYFGCYCQSKVCYH**---------- 33

45_1 ----KTEA**KIPCGESCVWIPCLTGYFGCYCQSKVCYHX**--------- 34

24_3 ----KTES**KIPCGESCVWIPCLTGYFGCYCQSKVCYX**---------- 33

52_3 ----KTET**KIPCGESCVWIPCLTGYFGCYCQSKVCYX**---------- 33

62_3 ----KKEA**KIPCGESCVWIPCLTGYFGCYCQSKVCYX**---------- 33

60_3 ----KTEA**KIHCGESCVWIPCLTGYFGCYCQSKVCYX**---------- 33

9_3 ----KTEA**KIPCGESCVWIPCLTGYFGCYCQSKVCYX**---------- 33

16_2 ---KKTEA**KIPCGESCVWIPCLTGYFGCYCQSKVCY**----------- 33

58_2 ---KKTEA**KIPCGESCVWIPCLTGYFGCYCQSKVCY**----------- 33

7_1 ---KKTEA**KIPCGESCVWIPCLTGYFGCYCQSKVCYX**---------- 34

71_3 --------**KIPCGESCVWIPCLTGYFGCYCQSKVCYHN**SNX------ 33

32_2 --------**KIPYGESCVWIPCLTGYFGCYCQSKVCYHN**SHI------ 33

11_2 --------**KIPCGESCVWIPCLTGYFGCYCQSKVCYHN**SHI------ 33

3_1 --------**KIPCGESCVWIPCLTGYFGCYCQSKVCYHN**SHIX----- 34

30_2 -------A**KIPCGESCVWIPCLTGYFGCYCQSKVCYHN**SH------- 33

12_1 -------A**KIPCGESCVWIPCLTGYFGCYCQSKVCYHN**SHX------ 34

18_3 --------**KIPCGESCVWIPCLTGYFGCYCQSKVCYHN**SHX------ 33

4_2 ----------**PCGESCVWIPCLTGYFGCYCQSKVCYHN**SHIAS---- 33

48_1 ----------**PCGESCVWIPCLTGYFGCYCQSKVCYHN**SHIASX--- 34

38_3 ---------**IPCGESCVWIPCLTGYFGCYCQSKVCYYN**SHIA----- 33

21_1 ---------**IPCGESCVWIPCLTGYFGCYCQSKVCYHN**SHIAX---- 34

40_2 ---------**IPCGESCVWIPCLTGYFGCYCQSKVCYHN**SHIA----- 33

61_3 ---------**IPCGESCVWIPCLTGYFGCYCQSKVCYHN**SHIA----- 33

33_2 ---------**IPCGESCVWIPCLTGYFGCYCQSKVCYHN**SNIA----- 33

********************

**Possible sequence:** **KIPCGESCVWIPCLTGYFGCYCQSKVCYHN**

**4. >Clitoria ternatea cyclotide precursor, 3prime_partial, ctr28841_c2_g4_i2, 2-154(-)**

**Query string:** CTTGTGTATGGATTCCATGTACTATAACAGCGCTTGTTGGTTGTGCATGCCATGAGAAA

**Reads:**

>1

CTTGTGTATGGATTCCATGTACTATAACAGCGCTTGTTGGTTGTGCATGCCATGAGAAAGTTTGCTATAAGTCCTCTAGCATTGCATCTACTGCAAAGAC

>2

GTGGAGAATCTTGTGTATGGATTCCATGTACTATAACAGCGCTTGTTGGTTGTGCATGCCATGAGAAAGTTTGCTATAAGTCCTCTAGCATTGCATCTAC

>3

AGAATCTTGTGTATGGATTCCATGTACTATAACAGCGCTTGTTGGTTGTGCATGCCATGAGAAAGTTTGCTATAAGTCCTCTAGCATTGCATCTACTGCA

>4

GAAAACAGGAGCTGCTCGTATTCCTTGTGGAGAAGCTTGTGTATGGATTCCATGTACTATAACAGCGCTTGTTGGTTGTGCATGCCATGAGAAAGTTTGC

>5

TGTGAAGAAAACAGGAGCTGCTCGTATTCCTTGTGGAGAATCTTGTGTATGGATTCCATGTACTATAACAGCGCTTGTTGGTTGTGCATGCCATGAGAAA

>6

CTTGTGTATGGATTCCATGTACTATAACAGCGCTTGTTGGTTGTGCATGCCATGAGAAAGTTTGCTATAAGTCCTCTAGCATTGCATCTACTGCAAAGAC

>7

CTCGTATTCCTTGTGGAGAATCTTGTGTATGGATTCCATGTACTATAACAGCGCTTGTTGGTTGTGCATGCCATGAGAAAGAGATCGGAAGAGCGTCGTG

>8

GAAGAAAACAGGAGCTGCTCGTATTCCTTGTGGAGAATCTTGTGTATGGATTCCATGTACTATAACAGCGCTTGTTGGTTGTGCATGCCATGAGAAAGTT

>9

AGCTGCTCGTATTCCTTGTGGAGAATCTTGTGTATGGATTCCATGTACTATAACAGCGCTTGTTGGTTGTGCATGCCATGAGAAAGTTTGCTATACGTCC

>10

GAAGAAAACAGGAGCTGCTCGTATTCCTTGTGGAGAATCTTGTGTATGGATTCCATGTACTATAACAGCGCTTGTTGGTTGTGCATGCCATGAGAAAGTT

>11

GAGAATCTTGTGTATGGATTCCATGTACTATAACAGCGCTTGTTGGTTGTGCATGCCATGAGAAAGTTTGCTATAAGTCCTCTAGCATTGCATCTACTGC

>12

AGAAAACAGGAGCTGCTCGTATTCCTTGTGGAGAATCTTGTGTATGGATTCCATGTACTATAACAGCGCTTGTTGGTTGTGCATGCCATGAGAAAGTTTG

>13

TATTCCTTGTGGAGAATCTTGTGTATGGATTCCATGTACTATAACAGCGCTTGTTGGTTGTGCATGCCATGAGAAAGTTTGCTATAAGTCCTCTAGCATT

>14

TGTGAAGAAAACAGGAGCTGCTCGTATTCCTTGTGGAGAATCTTGTGTATGGATTCCATGTACTATAACAGCGCTTGTTGGTTGTGCATGCCATGAGAAA

>15

AACAGGAGCTGCTCGTATTCCTTGTGGAGAATCTTGTGTATGGATTCCATGTACTATAACAGCGCTTGTTGGTTGTGCATGCCATGAGAAAGTTTGCTAT

>16

CTTGTGTATGGATTCCATGTACTATAACAGCGCTTGTTGGTTGTGCATGCCATGAGAAAGTTTGCTATAAGTCCTCTAGCATTGCATCTACTGCAAAGAC

>17

CTCGTATTCCTTGTGGAGAATCTTGTGTATGGATTCCATGTACTATAACAGCGCTTGTTGGTTGTGCATGCCATGAGAAAGTTTGCTATAAGTCCTCTAG

>18

ATCTTGTGTATGGATTCCATGTACTATAACAGCGCTTGTTGGTTGTGCATGCCATGAGAAAGTTTGCTATAAGTCCTCTAGCATTGCATCTACTGCAAAG

>19

CTCGTATTCCTTGTGGAGAATCTTGTGTATGGATTCCATGTACTATAACAGCGCTTGTTGGTTGTGCATGCCATGAGAAAGTTTGCTATAAGTCCTCTAG

>20

TGTGAAGAAAACAGGAGCTGCTCGTATTCCTTGTGGAGAATCTTGTGTATGGATTCCATGTACTATAACAGCGCTTGTTGGTTGTGCATGCCATGAGAAA

>21

TGTGAAGAAAACAGGAGCTGCTCGTATTCCTTGTGGAGAATCTTGTGTATGGATTCCATGTACTATAACAGCGCTTGTTGGTTGTGCATGCCATGAGAAA

>22

GTGAAGAAAACAGGAGCTGCTCGTATTCCTTGTGGAGAATCTTGTGTATGGATTCCATGTACTATAACAGCGCTTGTTGGTTGTGCATGCCATGAGAAAG

>23

CTTGTGTATGGATTCCATGTACTATAACAGCGCTTGTTGGTTGTGCATGCCATGAGAAAGTTTGCTATAAGTCCTCTAGCATTGCATCTACTGCAAAGAC

>24

AGGAGCTGCTCGTATTCCTTGTGGAGAATCTTGTGTATGGATTCCATGTACTATAACAGCGCTTGTTGGTTGTGCATGCCATGAGAAAGTTTGCTATAAG

>25

CTTGTGTATGGATTCCATGTACTATAACAGCGCTTGTTGGTTGTGCATGCCATGAGAAAGTTTGCTATAAGTCCTCTAGCATTGCATCTACTGCAAAGAC

>26

CGTATTCCTTGTGGAGAATCTTGTGTATGGATTCCATGTACTATAACAGCGCTTGTTGGTTGTGCATGCCATGAGAAAGTTTGCTATAAGTCCTCTAGCA

>27

GGAGAATCTTGTGTATGGATTCCATGTACTATAACAGCGCTTGTTGGTTGTGCATGCCATGAGAAAGTTTGCTATAAGTCCTCTAGCATTGCATCTACTG

>28

AAACAGGAGCTGCTCGTATTCCTTGTGGAGAATCTTGTGTATGGATTCCATGTACTATAACAGCGCTTGTTGGTTGTGCATGCCATGAGAAAGTTTGCTA

>29

AGAAAACAGGAGCTGCTCGTATTCCTTGTGGAGAATCTTGTGTATGGATTCCATGTACTATAACAGCGCTTGTTGGTTGTGCATGCCATGAGAAAGTTTG

>30

AGAATCTTGTGTATGGATTCCATGTACTATAACAGCGCTTGTTGGTTGTGCATGCCATGAGAAAGTTTGCTATAAGTCCTCTAGCATTGCATCTACTGCA

>31

GGAGAATCTTGTGTATGGATTCCATGTACTATAACAGCGCTTGTTGGTTGTGCATGCCATGAGAAAGTTTGCTATAAGTCCTCTAGCATTGCATCTACTG

>32

TGTGAAGAAAACAGGAGCTGCTCGTATTCCTTGTGGAGAATCTTGTGTATGGATTCCATGTACTATAACAGCGCTTGTTGGTTGTGCATGCCATGAGAAA

>33

AGAAAACAGGAGCTGCTCGTATTACTTGTGGAGAATCTTGTGTATGGATTCCATGTACTATAACAGCGCTTGTTGGTTGTGCATGCCATGAGAAAGTTTG

>34

GAGCTGCTCGTATTCCTTGTGGAGAATCTTGTGTATGGATTCCATGTACTATAACAGCGCTTGTTGGTTGTGCATGCCATGAGAAAGTTTGCTATAAGTC

>35

GAAAACAGGAGCTGCTCGTATTCCTTGTGGAGAATCTTGTGTATGGATTCCATGTACTATAACAGCGCTTGTTGGTTGTGCATGCCATGAGAAAGTTTGC

>36

AGAAAACAGGAGCTGCTCGTATTCCTTGTGGAGAATCTTGTGTATGGATTCCATGTACTATAACAGCGCTTGTTGGTTGTGCATGCCATGAGAAAGTTTG

>37

AGAAAACAGGAGCTGCTCGTATTCCTTGTGGAGAATCTTGTGTATGGATTCCATGTACTATAACAGCGCTTGTTGGTTGTGCATGCCATGAGAAAGTTTG

>38

GAAAACAGGAGCTGCTCGTATTCCTTGTGGAGAATCTTGTGTATGGATTCCATGTACTATAACAGCGCTTGTTGGTTGTGCATGCCATGAGAAAGTTTGC

>39

CTTGTGTATGGATTCCATGTACTATAACAGCGCTTGTTGGTTGTGCATGCCATGAGAAAGTTTGCTATAAGTCCTCTAGCATTGCATCTACTGCAAAGAC

>40

CTACTGAAAACAGGAGCTGCTCGTATTCCTTGTGGAGAATCTTGTGTATGGATTCCATGTACTATAACAGCGCTTGTTGGTTGTGCATGCCATGAGAAAG

>41

CTCGTATTCCTTGTGGAGAATCTTGTGTATGGATTCCATGTACTATAACAGCGCTTGTTGGTTGTGCATGCCATGAGAAAGTTTGCTATAAGTCCTCTAG

>42

GAAGAAAACAGGAGCTGCTCGTATTCCTTGTGGAGAATCTTGTGTATGGATTCCATGTACTATAACAGCGCTTGTTGGTTGTGCATGCCATGAGAAAGTT

>43

CTTGTGTATGGATTCCATGTACTATAACAGCGCTTGTTGGTTGTGCATGCCATGAGAAAGTTTGCTATAAGTCCTCTAGCATTGCATCTACTGCAAAGAC

>44

CTCGTATTCCTTGTGGAGAATCTTGTGTATGGATTCCATGTACTATAACAGCGCTTGTTGGTTGTGCATGCCATGAGAAAGTTTGCTATAAGTCCTCTAG

>45

CTTGTGTATGGATTCCATGTACTATAACAGCGCTTGTTGGTTGTGCATGCCATGAGAAAGTTTGCTATAAGTCCTCTAGCATTGCATCTACTGCAAAGAC

>46

AGAAAACAGGAGCTGCTCGTATTCCTTGTGGAGAATCTTGTGTATGGATTCCATGTACTATAACAGCGCTTGTTGGTTGTGCATGCCATGAGAAAGTTTG

>47

CTTGTGTATGGATTCCATGTACTATAACAGCGCTTGTTGGTTGTGCATGCCATGAGAAAGTTTGCTATAAGTCCTCTAGCATTGCATCTACTGCAAAGAC

>48

CTTGTGTATGGATTCCATGTACTATAACAGCGCTTGTTGGTTGTGCATGCCATGAGAAAGTTTGCTATAAGTCCTCTAGCATTGCATCTACTGCAAAGAC

>49

AAAACAGGAGCTGCTCGTATTCCTTGTGGAGAATCTTGTGTATGGATTCCATGTACTATAACAGCGCTTGTTGGTTGTGCATGCCATGAGAAAGTTTGCT

>50

AGAAAACAGGAGCTGCTCGTATTCCTTGTGGAGAATCTTGTGTATGGATTCCATGTACTATAACAGCGCTTGTTGGTTGTGCATGCCATGAGAAAGTTTG

>51

GCTCGTATTCCTTGTGGAGAATCTTGTGTATGGATTCCATGTACTATAACAGCGCTTGTTGGTTGTGCATGCCATGAGAAAGTTTGCTATAAGTCCTCTA

>52

AACAGGAGCTGCTCGTATTCCTTGTGGAGAATCTTGTGTATGGATTCCATGTACTATAACAGCGCTTGTTGGTTGTGCATGCCATGAGAAAGTTTGCTAT

>53

CAGGAGCTGCTCGTATTCCTTGTGGAGAATCTTGTGTATGGATTCCATGTACTATAACAGCGCTTGTTGGTTGTGCATGCCATGAGAAAGTTTGCTATAA

>54

AAACAGGAGCTGCTCGTATTCCTTGTGGAGAATCTTGTGTATGGATTCCATGTACTATAACAGCGCTTGTTGGTTGTGCATGCCATGAGAAAGTTTGCTA

**CDS to Protein Translation (only cyclotide-like transcripts):**

>5_2

VKKTGAARIPCGESCVWIPCTITALVGCACHEK

>7_3

RIPCGESCVWIPCTITALVGCACHEKEIGRASX

>8_2

KKTGAARIPCGESCVWIPCTITALVGCACHEKV

>9_2

AARIPCGESCVWIPCTITALVGCACHEKVCYTS

>10_2

KKTGAARIPCGESCVWIPCTITALVGCACHEKV

>12_3

KTGAARIPCGESCVWIPCTITALVGCACHEKVX

>13_2

IPCGESCVWIPCTITALVGCACHEKVCYKSSSI

>14_2

VKKTGAARIPCGESCVWIPCTITALVGCACHEK

>15_2

TGAARIPCGESCVWIPCTITALVGCACHEKVCY

>17_3

RIPCGESCVWIPCTITALVGCACHEKVCYKSSX

>19_3

RIPCGESCVWIPCTITALVGCACHEKVCYKSSX

>20_2

VKKTGAARIPCGESCVWIPCTITALVGCACHEK

>21_2

VKKTGAARIPCGESCVWIPCTITALVGCACHEK

>22_1

VKKTGAARIPCGESCVWIPCTITALVGCACHEKX

>24_2

GAARIPCGESCVWIPCTITALVGCACHEKVCYK

>26_1

RIPCGESCVWIPCTITALVGCACHEKVCYKSSSX

>28_3

TGAARIPCGESCVWIPCTITALVGCACHEKVCX

>29_3

KTGAARIPCGESCVWIPCTITALVGCACHEKVX

>32_2

VKKTGAARIPCGESCVWIPCTITALVGCACHEK

>33_3

KTGAARITCGESCVWIPCTITALVGCACHEKVX

>34_3

AARIPCGESCVWIPCTITALVGCACHEKVCYKS

>35_2

KTGAARIPCGESCVWIPCTITALVGCACHEKVC

>36_3

KTGAARIPCGESCVWIPCTITALVGCACHEKVX

>37_3

KTGAARIPCGESCVWIPCTITALVGCACHEKVX

>38_2

KTGAARIPCGESCVWIPCTITALVGCACHEKVC

>40_1

LLKTGAARIPCGESCVWIPCTITALVGCACHEKX

>41_3

RIPCGESCVWIPCTITALVGCACHEKVCYKSSX

>42_2

KKTGAARIPCGESCVWIPCTITALVGCACHEKV

>44_3

RIPCGESCVWIPCTITALVGCACHEKVCYKSSX

>46_3

KTGAARIPCGESCVWIPCTITALVGCACHEKVX

>49_1

KTGAARIPCGESCVWIPCTITALVGCACHEKVCX

>50_3

KTGAARIPCGESCVWIPCTITALVGCACHEKVX

>51_1

ARIPCGESCVWIPCTITALVGCACHEKVCYKSSX

>52_2

TGAARIPCGESCVWIPCTITALVGCACHEKVCY

>53_3

GAARIPCGESCVWIPCTITALVGCACHEKVCYX

>54_3

TGAARIPCGESCVWIPCTITALVGCACHEKVCX

**Multiple sequence alignment:**

7_3 -------**RIPCGESCVWIPCTITALVGCACHEK**EIGRASX- 33

40_1 LLKTGA**ARIPCGESCVWIPCTITALVGCACHEK**X------- 34

33_3 --KTGA**ARITCGESCVWIPCTITALVGCACHEK**VX------ 33

12_3 --KTGA**ARIPCGESCVWIPCTITALVGCACHEK**VX------ 33

29_3 --KTGA**ARIPCGESCVWIPCTITALVGCACHEK**VX------ 33

36_3 --KTGA**ARIPCGESCVWIPCTITALVGCACHEK**VX------ 33

37_3 --KTGA**ARIPCGESCVWIPCTITALVGCACHEK**VX------ 33

46_3 --KTGA**ARIPCGESCVWIPCTITALVGCACHEK**VX------ 33

50_3 --KTGA**ARIPCGESCVWIPCTITALVGCACHEK**VX------ 33

5_2 VKKTGA**ARIPCGESCVWIPCTITALVGCACHEK**-------- 33

14_2 VKKTGA**ARIPCGESCVWIPCTITALVGCACHEK**-------- 33

20_2 VKKTGA**ARIPCGESCVWIPCTITALVGCACHEK**-------- 33

21_2 VKKTGA**ARIPCGESCVWIPCTITALVGCACHEK**-------- 33

22_1 VKKTGA**ARIPCGESCVWIPCTITALVGCACHEK**X------- 34

32_2 VKKTGA**ARIPCGESCVWIPCTITALVGCACHEK**-------- 33

8_2 -KKTGA**ARIPCGESCVWIPCTITALVGCACHEK**V------- 33

10_2 -KKTGA**ARIPCGESCVWIPCTITALVGCACHEK**V------- 33

42_2 -KKTGA**ARIPCGESCVWIPCTITALVGCACHEK**V------- 33

17_3 -------**RIPCGESCVWIPCTITALVGCACHEK**VCYKSSX- 33

19_3 -------**RIPCGESCVWIPCTITALVGCACHEK**VCYKSSX- 33

41_3 -------**RIPCGESCVWIPCTITALVGCACHEK**VCYKSSX- 33

44_3 -------**RIPCGESCVWIPCTITALVGCACHEK**VCYKSSX- 33

51_1 ------**ARIPCGESCVWIPCTITALVGCACHEK**VCYKSSX- 34

13_2 --------**IPCGESCVWIPCTITALVGCACHEK**VCYKSSSI 33

26_1 -------**RIPCGESCVWIPCTITALVGCACHEK**VCYKSSSX 34

9_2 -----A**ARIPCGESCVWIPCTITALVGCACHEK**VCYTS--- 33

34_3 -----A**ARIPCGESCVWIPCTITALVGCACHEK**VCYKS--- 33

28_3 ---TGA**ARIPCGESCVWIPCTITALVGCACHEK**VCX----- 33

49_1 --KTGA**ARIPCGESCVWIPCTITALVGCACHEK**VCX----- 34

54_3 ---TGA**ARIPCGESCVWIPCTITALVGCACHEK**VCX----- 33

35_2 --KTGA**ARIPCGESCVWIPCTITALVGCACHEK**VC------ 33

38_2 --KTGA**ARIPCGESCVWIPCTITALVGCACHEK**VC------ 33

15_2 ---TGA**ARIPCGESCVWIPCTITALVGCACHEK**VCY----- 33

52_2 ---TGA**ARIPCGESCVWIPCTITALVGCACHEK**VCY----- 33

24_2 ----GA**ARIPCGESCVWIPCTITALVGCACHEK**VCYK---- 33

53_3 ----GA**ARIPCGESCVWIPCTITALVGCACHEK**VCYX---- 33

*************************

**Possible sequence: ARIPCGESCVWIPCTITALVGCACHEK**

**5. >Clitoria ternatea cyclotide precursor, internal, ctr****29746_c1_g3_i3, 2-256(-)**

**Query string:** TATAACAAACATTGCTTTTACAGGAACATCCGACAATTGTCGAGATCGGAAGAG

**Reads:**

>1

CGATGTTCATCCAATGATTTTGCAGTGGCAGCAATGACATGGTTACTATAACAAACATTGCTTTTACAGGAACATCCGACAATTGTCGAGATCGGAAGAG

>2

ATCCAATGATTTTGCAGTGGCAGCAATGACATGGTTACTATAACAAACATTGCTTTTACAGGAACATCCGACAATTGTCGAGATCGGAAGAGCACACGTC

>3

GCAGTGGCAGCAATGACATGGTTACTATAACAAACATTGCTTTTACAGGAACATCCGACAATTGTCGAGATCGGAAGAGCCCACGTCTGAACTCCAGTCA

>4

CGATGTTCATCCAATGATTTTGCAGTGGCAGCAATGACATGGTTACTATAACAAACATTGCTTTTACAGGAACATCCGACAATTGTCGAGATCGGAAGAG

>5

CGATGTTCATCCAATGATTTTGCAGTGGCAGCAATGACATGGTTACTATAACAAACATTGCTTTTACAGGAACATCCGACAATTGTCGAGATCGGAAGAG

>6

GTTCATCCAATGATTTTGCAGTGGCAGCAATGACATGGTTACTATAACAAACATTGCTTTTACAGGAACATCCGACAATTGTCGAGATCGGAAGAGCACA

>7

CAATGATTTTGCAGTGGCAGCAATGACATGGTTACTATAACAAACATTGCTTTTACAGGAACATCCGACAATTGTCGAGATCGGAAGAGCACCCGTCTGA

>8

TGTTCATCCAATGATTTTGCAGTGGCAGCAATGACATGGTTACTATAACAAACATTGCTTTTACAGGAACATCCGACAATTGTCGAGATCGGAAGAGCAC

>9

CAATGATTTTGCAGTGGCAGCAATGACATGGTTACTATAACAAACATTGCTTTTACAGGAACATCCGACAATTGTCGAGATCGGAAGAGCACACGTCTGA

>10

TGTTCATCCAATGATTTTGCAGTGGCAGCAATGACATGGTTACTATAACAAACATTGCTTTTACAGGAACATCCGACAATTGTCGAGATCGGAAGAGCAC

>11

CGATGTTCATCCAATGATTTTGCAGTGGCAGCAATGACATGGTTACTATAACAAACATTGCTTTTACAGGAACATCCGACAATTGTCGAGATCGGAAGAG

>12

CGATGTTCATCCAATGATTTTGCAGTGGCAGCAATGACATGGTTACTATAACAAACATTGCTTTTACAGGAACATCCGACAATTGTCGAGATCGGAAGAG

>13

AATGATTTTGCAGTGGCAGCAATGACATGGTTACTATAACAAACATTGCTTTTACAGGAACATCCGACAATTGTCGAGATCGGAAGAGCACACGTCTGAA

>14

TGATTTTGCAGTGGCAGCAATGACATGGTTACTATAACAAACATTGCTTTTACAGGAACATCCGACAATTGTCGAGATCGGAAGAGCACACGTCTGAACT

>15

GTTCATCCAATGATTTTGCAGTGGCAGCAATGACATGGTTACTATAAAAAACATTGCTTTTACAGGAACATCCGACAATTGTCGAGATCGGAAGAGCACA

>16

CGATGTTCATCCAATGATTTTGCAGTGGCAGCAATGACATGGTTACTATAACAAACATTGCTTTTACAGGAACATCCGACAATTGTCGAGATCGGAAGAG

>17

CGATGTTCATCCAATGATTTTGCAGTGGCAGCAATGACATGGTTACTATAACAAACATTGCTTTTACAGGAACATCCGACAATTGTCGAGATCGGAAGAG

>18

CAATGATTTTGCAGTGGCAGCAATGACATGGTTACTATAACAAACATTGCTTTTACAGGAACATCCGACAATTGTCGAGATCGGAAGAGCACACGTCTGA

>19

AGCAATGACATGGTTACTATAACAAACATTGCTTTTACAGGAACATCCGACAATTGTCGAGATCGGAAGAGCACACGTCTGAACTCCAGTCACTTAGGCA

>20

CGATGTTCATCCAATGATTTTGCAGTGGCAGCAATGACATGGTTACTATAACAAACATTGCTTTTACAGGAACATCCGACAATTGTCGAGATCGGAAGAG

>21

CGATGTTCATCCAATGATTTTGCAGTGGCAGCAATGACATGGTTACTATAACAAACATTGCTTTTACAGGAACATCCGACAATTGTCGAGATCGGAAGAG

>22

GTTCATCCAATGATTTTGCAGTGGCAGCAATGACATGGTTACTATAACAAACATTGCTTTTACAGGAACATCCGACAATTGTCGAGATCGGAAGAGCACA

>23

GTTCATCCAATGATTTTGCAGTGGCAGCAATGACATGGTTACTATAACAAACATTGCTTTTACAGGAACATCCGACAATTGTCGAGATCGGAAGAGCACA

>24

TGTTCATCCAATGATTTTGCAGTGGCAGCAATGACATGGTTACTATAACAAACATTGCTTTTACAGGAACATCCGACAATTGTCGAGATCGGAAGAGCAC

>25

GTTCATCCAATGATTTTGCAGTGGCAGCAATGACATGGTTACTATAACAAACATTGCTTTTACAGGAACATCCGACAATTGTCGAGATCGGAAGAGCACA

>26

GCCAGTGGCAGCAATGACATGGTTACTATAACAAACATTGCTTTTACAGGAACATCCGACAATTGTCGAGATCGGAAGAGCACACGTCTGAACTCCAGTC

>27

CGATGTTCATCCAATGATTTTGCAGTGGCAGCAATGACATGGTTACTATAACAAACATTGCTTTTACAGGAACATCCGACAATTGTCGAGATCGGAAGAG

>28

TGATTTTGCAGTGGCAGCAATGACATGGTTACTATAACAAACATTGCTTTTACAGGAACATCCGACAATTGTCGAGATCGGAAGAGCACACGTCTGAACT

>29

CAATGATTTTGCAGTGGCAGCAATGACATGGTTACTATAACAAACATTGCTTTTACAGGAACATCCGACAATTGTCGAGATCGGAAGAGCACACGTCTGA

>30

CAATGATTTTGCAGTGGCAGCAATGACATGGTTACTATAACAAACATTGCTTTTACAGGAACATCCGACAATTGTCGAGATCGGAAGAGCACACGTCTGA

>31

TGTTCATCCAATGATTTTGCAGTGGCAGCAATGACATGGTTACTATAACAAACATTGCTTTTACAGGAACATCCGACAATTGTCGAGATCGGAAGAGCAC

>32

CGATGTTCATCCAATGATTTTGCAGTGGCAGCAATGACATGGTTACTATAACAAACATTGCTTTTACAGGAACATCCGACAATTGTCGAGATCGGAAGAG

>33

CGATGTTCATCCAATGATTTTGCAGTGGCAGCAATGACATGGTTACTATAACAAACATTGCTTTTACAGGAACATCCGACAATTGTCGAGATCGGAAGAG

>34

TGTTCATCCAATGATTTTGCAGTGGCAGCAATGACATGGTTACTATAACAAACATTGCTTTTACAGGAACATCCGACAATTGTCGAGATCGGAAGAGCAC

>35

GTTCATCCAATGATTTTGCAGTGGCAGCAATGACATGGCTACTATAACAAACATTGCTTTTACAGGAACATCCGACAATTGTCGAGATCGGAAGAGCCAC

>36

CAATGATTTTGCAGTGGCAGCAATGACATGGTTACTATAACAAACATTGCTTTTACAGGAACATCCGACAATTGTCGAGATCGGAAGAGCACACGTCTGA

>37

CAATGATTTTGCAGTGGCAGCAATGACATGGTTACTATAACAAACATTGCTTTTACAGGAACATCCGACAATTGTCGAGATCGGAAGAGCACACGTCTGA

>38

CCAATGATTTTGCAGTGGCAGCAATGACATGGTTACTATAACAAACATTGCTTTTACAGGAACATCCGACAATTGTCGAGATCGGAAGAGCACACGTCTG

>39

ATGATTTTGCAGTGGCAGCAATGACATGGTTACTATAACAAACATTGCTTTTACAGGAACATCCGACAATTGTCGAGATCGGAAGAGCACACGTCTGAAC

>40

CAATGATTTTGCAGTGGCAGCAATGACATGGTTACTATAACAAACATTGCTTTTACAGGAACATCCGACAATTGTCGAGATCGGAAGAGCACACGTCTGA

>41

TGTTCATCCAATGATTTTGCAGTGGCAGCAATGACATGATTACTATAACAAACATTGCTTTTACAGGAACATCCGACAATTGTCGAGATCGGAAGAGCAC

>42

CAATGATTTTGCAGTGGCAGCAATGACATGGTTACTATAACAAACATTGCTTTTAAAGGAACATCCGACAATTGTCGAGATCGGAAGAGCACACGTCTGA

>43

CGATGTTCATCCAATGATTTTGCAGTGGCAGCAATGACATGGTTACTATAACAAACATTGCTTTTACAGGAACATCCGACAATTGTCGAGATCGGAAGAG

>44

TGTTCATCCAATGATTTTGCAGTGGCAGCAATGACATGGTTACTATAACAAACATTGCTTTTACAGGAACATCCGACAATTGTCGAGATCGGAAGAGCAC

>45

CAATGATTTTGCAGTGGCAGCAATGACATGGTTACTATAACAAACATTGCTTTTACAGGAACATCCGACAATTGTCGAGATCGGAAGAGCACACGTCTGA

>46

CAATGATTTTGCAGTGGCAGCAATGACATGGTTACTATAACAAACATTGCTTTTACAGGAACATCCGACAATTGTCGAGATCGGAAGAGCACACGTCTGA

>47

TGTTCATCCAATGATTTTGCAGTGGCAGCAATGACATGGTTACTATAACAAACATTGCTTTTACAGGAACATCCGACAATTGTCGAGATCGGAAGAGCAC

>48

CGATGTTCATCCAATGATTTTGCAGTGGCAGCAATGACATGGGTACTATAACAAACATTGCTTTTACAGGAACATCCGACAATTGTCGAGATCGGAAGAG

>49

CGATGTTCATCCAATGATTTTGCAGTGGCAGCAATGACATGGTTACTATAACAAACATTGCTTTTACAGGAACATCCGACAATTGTCGAGATCGGAAGAG

>50

CGATGTTCATCCAATGATTTTGCAGTGGCAGCAATGACATGGTTACTATAACAAACATTGCTTTTACAGGAACATCCGACAATTGTCGAGATCGGAAGAG

>51

CCAATGATTTTGCAGTGGCAGCAATGACATGGTTACTATAACAAACATTGCTTTTACAGGAACATCCGACAATTGTCGAGATCGGAAGAGCACACGTCTG

>52

CGATGTTCATCCAATGATTTTGCAGTGGCAGCAATGACATGGTTACTATAACAAACATTGCTTTTACAGGAACATCCGACAATTGTCGAGATCGGAAGAG

>53

CCAATGATTTTGCAGTGGCAGCAATGACATGGTTACTATAACAAACATTGCTTTTACAGGAACATCCGACAATTGTCGAGATCGGAAGAGCACACGTCTG

>54

CGATGTTCATCCAATGATTTTGCAGTGGCAGCAATGACATGGTTACTATAACAAACATTGCTTTTACAGGAACATCCGACAATTGTCGAGATCGGAAGAG

>55

GTTCATCCAATGATTTTGCAGTGGCAGCAATGACATGGTTACTATAACAAACATTGCTTTTACAGGAACATCCGACAATTGTCGAGATCGGAAGAGCACA

>56

CAATGATTTTGCAGTGGCAGCAATGACATGGTTACTATAACAAACATTGCTTTTACAGGAACATCCGACAATTGTCGAGATCGGAAGAGCACACGTCTGA

>57

CGATGTTCATCCAATGATTTTGCAGTGGCAGCAATGACATGGTTACTATAACAAACATTGCTTTTACAGGAACATCCGACAATTGTCGAGATCGGAAGAG

>58

AGCAGTGGCAGCAATGACATGGTTACTATAACAAACATTGCTTTTACAGGAACATCCGACAATTGTCGAGATCGGAAGAGCACACGTCTGAACTCCAGTC

>59

CAATGATTTTGCAGTGGCAGCAATGACATGGTTACTATAACAAACATTGCTTTACAGGAACATCCGACAATTGTCGAGATCGGAAGAGCACACGTCTGAA

>60

TTTTGCAGTGGCAGCAATGACATGGTTACTATAACAAACATTGCTTTTACAGGAACATCCGACAATTGTCGAGATCGGAAGAGCACACGTCTGAACTCCA

>61

TCCAATGATTTTGCAGTGGCAGCAATGACATGGTTACTATAACAAACATTGCTTTTACAGGAACATCCGACAATTGTCGAGATCGGAAGAGCACACGTCT

>62

GGCAGCAATGACATGGTTACTATAACAAACATTGCTTTTACAGGAACATCCGACAATTGTCGAGATCGGAAGAGCACACGTCTAAACTCCAGTCACTTAG

>63

CAATGATTTTGCAGTGGCAGCAATGACATGGTTACTATAACAAACATTGCTTTTACAGGAACATCCGACAATTGTCGAGATCGGAAGAGCACACGTCTGA

>64

TGTTCATCCAATGATTTTGCAGTGGCAGCAATGACATGGTTACTATAACAAACATTGCTTTTACAGGAACATCCGACAATTGTCGAGATCGGAAGAGCAC

>65

GTTCATCCAATGATTTTGCAGTGGCAGCAATGACATGGTTACTATAACAAACATTGCTTTTACAGGAACATCCGACAATTGTCGAGATCGGAAGAGCACA

>66

GTTCATCCAATGATTTTGCAGTGGCAGCAATGACATGGTTACTATAACAAACATTGCTTTTACAGGAACATCCGACAATTGTCGAGATCGGAAGAGCACA

>67

CGATGTTCATCCAATGATTTTGTAGTGGCAGCAATGACATGGTTACTATAACAAACATTGCTTTTACAGGAACATCCGACAATTGTCGAGATCGGAAGAG

>68

CCAATGATTTTGCAGTGGCAGCAATGACATGGTTACTATAACAAACATTGCTTTTACAGGAACATCCGACAATTGTCGAGATCGGAAGAGCACACGTCTG

>69

TGTTCATCCAATGATTTTGCAGTGGCAGCAATGACATGGTTACTATAACAAACATTGCTTTTACAGGAACATCCGACAATTGTCGAGATCGGAAGAGCAC

>70

CAATGATTTTGCAGTGGCAGCAATGACATGGTTACTATAACAAACATTGCTTTTACAGGAACATCCGACAATTGTCGAGATCGGAAGAGCACACGTCTGA

>71

GTTCATCCAATGATTTTGCAGTGGCAGCAATGACATGGTTACTATAACAAACATTGCTTTTACAGGAACATCCGACAATTGTCGAGATCGGAAGAGCACA

>72

TGTTCATCCAATGATTTTGCAGTGGCAGCAATGACATGGTTACTATAACAAACATTGCTTTTACAGGAACATCCGACAATTGTCGAGATCGGAAGAGCAC

>73

GATGTTCATCCAATGATTTTGCAGTGGCAGCAATGACATGGTTACTATAACAAACATTGCTTTTACAGGAACATCCGACAATTGTCGAGATCGGAAGAGC

>74

CGATGTTCATCCAATGATTTTGCAGTGGCAGCAATGACATGGTTACTATAACAAACATTGCTTTTACAGGAACATCCGACAATTGTCGAGATCGGAAGAG

>75

CGATGTTCATCCAATGATTTTGCAGTGGCAGCAATAACATGGTTACTATAACAAACATTGCTTTTACAGGAACATCCGACAATTGTCGAGATCGGAAGAG

>76

CGATGTTCATCCAATGATTTTGCAGTGGCAGCAATGACATGGTTACTATAACAAACATTGCTTTTACAGGAACATCCGACAATTGTCGAGATCGGAAGAG

>77

CGATGTTCATCCAATGATTTTGCAGTGGCAGCAATGACATGGTTACTATAACAAACATTGCTTTTACAGGAACATCCGACAATTGTCGAGATCGGAAGAG

>78

TGTTCATCCAATGATTTTGCAGTGGCAGCAATGACATGGTTACTATAACAAACATTGCTTTTACAGGAACATCCGACAATTGTCGAGATCGGAAGAGCAC

>79

CGATGTTCATCCAATGATTTTGCAGTGGCAGCAATGACATGGTTACTATAACAAACATTGCTTTTACAGGAACATCCGACAATTGTCGAGATCGGAAGAG

>80

CGATGTTCATCCAATGATTTTGCAGTGGCAGCAATGACATGGTTACTATAACAAACATTGCTTTTACAGGAACATCCGACAATTGTCGAGATCGGAAGAG

>81

CGATGTTCATCCAATGATTTTGCAGTGGCAGCAATGACATGGTTACTATAACAAACATTGCTTTTACAGGAACATCCGACAATTGTCGAGATCGGAAGAG

>82

GTTCATCCAATGATTTTGCAGTGGCAGCAATGACATGGTTACTATAACAAACATTGCTTTTACAGGAACATCCGACAATTGTCGAGATCGGAAGAGCACA

>83

TGCAGTGGCAGCAATGACATGGTTACTATAACAAACATTGCTTTTACAGGAACATCCGACAATTGTCGAGATCGGAAGAGCACACGTCTGAACTCCAGTC

>84

TGTTCATCCAATGATTTTGCAGTGGCAGCAATGACATGGTTACTATAACAAACATTGCTTTTACAGGAACATCCGACAATTGTCGAGATCGGAAGAGCAC

>85

GGCAGTGGCAGCAATGACATGGTTACTATAACAAACATTGCTTTTACAGGAACATCCGACAATTGTCGAGATCGGAAGAGCACACGTCTGAACTCCAGTC

>86

CGATGTTCATCCAATGATTTTGCAGTGGCAGCAATGACATGGTTACTATAACAAACATTGCTTTTACAGGAACATCCGACAATTGTCGAGATCGGAAGAG

>87

TGTTCATCCAATGATTTTGCAGTGGCAGCAATGACATGGTTACTATAACAAACATTGCTTTTACAGGAACATCCGACAATTGTCGAGATCGGAAGAGCAC

>88

TGTTCATCCAATGATTTTGCAGTGGCAGCAATGACATGGTTACTATAACAAACATTGCTTTTACAGGAACATCCGACAATTGTCGAGATCGGAAGAGCAC

>89

CAATGATTTTGCAGTGGCAGCAATGACATGGTTACTATAACAAACATTGCTTTTACAGGAACATCCGACAATTGTCGAGATCGGAAGAGCACACGTCTGA

>90

CAATGATTTTGCAGTGGCAGCAATGACATGGTTACTATAACAAACATTGCTTTTACAGGAACATCCGACAATTGTCGAGATCGGAAGAGCACACGTCTGA

>91

CAATGATTTTGCAGTGGCAGCAATGACATGGTTACTATAACAAACATTGCTTTTACAGGAACATCCGACAATTGTCGAGATCGGAAGAGCACACGTCTGA

>92

CGATGTTCATCCAATGATTTTGCAGTGGCAGCAATGACATGGTTACTATAACAAACATTGCTTTTACAGGAACATCCGACAATTGTCGAGATCGGAAGAG

>93

CGATGTTCATCCAATGATTTTGCAGTGGCAGCAATGACATGGTTACTATAACAAACATTGCTTTTACAGGAACATCCGACAATTGTCGAGATCGGAAGAG

>94

CCAATGATTTTGCAGTGGCAGCAATGACATGGTTACTATAACAAACATTGCTTTTACAGGAACATCCGACAATTGTCGAGATCGGAAGAGCACACGTCTG

>95

CAATGATTTTGCAGTGGCAGCAATGACATGGTTACTATAACAAACATTGCTTTTACAGGAACATCCGACAATTGTCGAGATCGGAAGAGCACACGTCTGA

>96

GCAGTGGCAGCAATGACATGGTTACTATAACAAACATTGCTTTTACAGGAACATCCGACAATTGTCGAGATCGGAAGAGCACACGTCTGAACTCCAGTCA

>97

TGTTCATCCAATGATTTTGCAGTGGCAGCAATGACATGGTTACTATAACAAACATTGCTTTTACAGGAACATCCGACAATTGTCGAGATCGGAAGAGCAC

>98

CAATGATTTTGCAGTGGCAGCAATGACATGGTTACTATAACAAACATTGCTTTTACAGGAACATCCGACAATTGTCGAGATCGGAAGAGCACACGTCTGA

>99

ATCCAATGATTTTGCAGTGGCAGCAATGACATGGTTACTATAACAAACATTGCTTTTACAGGAACATCCGACAATTGTCGAGATCGGAAGAGCACACGTC

>100

GCAGTGGCAGCAATGACATGGTTACTATAACAAACATTGCTTTTACAGGAACATCCGACAATTGTCGAGATCGGAAGAGCACACGTCTGAACTCCAGTCA

>101

TGTTCATCCAATGATTTTGCAGTGGCAGCAATGACATGGTTACTATAACAAACATTGCTTTTACAGGAACATCCGACAATTGTCGAGATCGGAAGAGCAC

>102

TGTTCATCCAATGATTTTGCAGTGGCAGCAATGACATGGTTACTATAACAAACATTGCTTTTACAGGAACATCCGACAATTGTCGAGATCGGAAGAGCAC

>103

TGTTCATCCAATGATTTTGCAGTGGCAGCAATGACATGGTTACTATAACAAACATTGCTTTTACAGGAACATCCGACAATTGTCGAGATCGGAAGAGCAC

>104

ATTTTGCAGTGGCAGCAATGACATGGTTACTATAACAAACATTGCTTTTACAGGAACATCCGACAATTGTCGAGATCGGAAGAGCACACGTCTGAACTCC

>105

ATGTTCATCCAATGATTTTGCAGTGGCAGCAATGACATGGTTACTATAACAAACATTGCTTTTACAGGAACATCCGACAATTGTCGAGATCGGAAGAGCA

>106

TGTTCATCCAATGATTTTGCAGTGGCAGCAATGACATGGTTACTATAACAAACATTGCTTTTACAGGAACATCCGACAATTGTCGAGATCGGAAGAGCAC

>107

CAATGATTTTGCAGTGGCAGCAATGACATGGTTACTATAACAAACATTGCTTTTACAGGAACATCCGACAATTGTCGAGATCGGAAGAGCACACGTCTGA

**CDS to Protein Translation (only cyclotide-like transcripts):**

>2_4

TCALPISTIVGCSCKSNVCYSNHVIAATAKSLD

>3_6

TGVQTWALPISTIVGCSCKSNVCYSNHVIAATA

>6_5

CALPISTIVGCSCKSNVCYSNHVIAATAKSLDEX

>7_4

QTGALPISTIVGCSCKSNVCYSNHVIAATAKSL

>8_6

ALPISTIVGCSCKSNVCYSNHVIAATAKSLDEX

>9_4

QTCALPISTIVGCSCKSNVCYSNHVIAATAKSL

>10_6

ALPISTIVGCSCKSNVCYSNHVIAATAKSLDEX

>13_6

QTCALPISTIVGCSCKSNVCYSNHVIAATAKSX

>14_4

VQTCALPISTIVGCSCKSNVCYSNHVIAATAKS

>15_5

CALPISTIVGCSCKSNVFYSNHVIAATAKSLDEX

>18_4

QTCALPISTIVGCSCKSNVCYSNHVIAATAKSL

>19_4

A*VTGVQTCALPISTIVGCSCKSNVCYSNHVIA

>22_5

CALPISTIVGCSCKSNVCYSNHVIAATAKSLDEX

>23_5

CALPISTIVGCSCKSNVCYSNHVIAATAKSLDEX

>24_6

ALPISTIVGCSCKSNVCYSNHVIAATAKSLDEX

>25_5

CALPISTIVGCSCKSNVCYSNHVIAATAKSLDEX

>26_4

TGVQTCALPISTIVGCSCKSNVCYSNHVIAATG

>28_4

VQTCALPISTIVGCSCKSNVCYSNHVIAATAKS

>29_4

QTCALPISTIVGCSCKSNVCYSNHVIAATAKSL

>30_4

QTCALPISTIVGCSCKSNVCYSNHVIAATAKSL

>31_6

ALPISTIVGCSCKSNVCYSNHVIAATAKSLDEX

>34_6

ALPISTIVGCSCKSNVCYSNHVIAATAKSLDEX

>35_5

VALPISTIVGCSCKSNVCYSSHVIAATAKSLDEX

>36_4

QTCALPISTIVGCSCKSNVCYSNHVIAATAKSL

>37_4

QTCALPISTIVGCSCKSNVCYSNHVIAATAKSL

>38_5

QTCALPISTIVGCSCKSNVCYSNHVIAATAKSLX

>39_5

VQTCALPISTIVGCSCKSNVCYSNHVIAATAKSX

>40_4

QTCALPISTIVGCSCKSNVCYSNHVIAATAKSL

>41_6

ALPISTIVGCSCKSNVCYSNHVIAATAKSLDEX

>42_4

QTCALPISTIVGCSFKSNVCYSNHVIAATAKSL

>44_6

ALPISTIVGCSCKSNVCYSNHVIAATAKSLDEX

>45_4

QTCALPISTIVGCSCKSNVCYSNHVIAATAKSL

>46_4

QTCALPISTIVGCSCKSNVCYSNHVIAATAKSL

>47_6

ALPISTIVGCSCKSNVCYSNHVIAATAKSLDEX

>51_5

QTCALPISTIVGCSCKSNVCYSNHVIAATAKSLX

>53_5

QTCALPISTIVGCSCKSNVCYSNHVIAATAKSLX

>55_5

CALPISTIVGCSCKSNVCYSNHVIAATAKSLDEX

>56_4

QTCALPISTIVGCSCKSNVCYSNHVIAATAKSL

>58_4

TGVQTCALPISTIVGCSCKSNVCYSNHVIAATA

>59_6

QTCALPISTIVGCSCKAMFVIVTMSLLPLQNHX

>60_4

GVQTCALPISTIVGCSCKSNVCYSNHVIAATAK

>61_6

TCALPISTIVGCSCKSNVCYSNHVIAATAKSLX

>62_4

*VTGV*TCALPISTIVGCSCKSNVCYSNHVIAA

>63_4

QTCALPISTIVGCSCKSNVCYSNHVIAATAKSL

>64_6

ALPISTIVGCSCKSNVCYSNHVIAATAKSLDEX

>65_5

CALPISTIVGCSCKSNVCYSNHVIAATAKSLDEX

>66_5

CALPISTIVGCSCKSNVCYSNHVIAATAKSLDEX

>68_5

QTCALPISTIVGCSCKSNVCYSNHVIAATAKSLX

>69_6

ALPISTIVGCSCKSNVCYSNHVIAATAKSLDEX

>70_4

QTCALPISTIVGCSCKSNVCYSNHVIAATAKSL

>71_5

CALPISTIVGCSCKSNVCYSNHVIAATAKSLDEX

>72_6

ALPISTIVGCSCKSNVCYSNHVIAATAKSLDEX

>73_5

ALPISTIVGCSCKSNVCYSNHVIAATAKSLDEHX

>78_6

ALPISTIVGCSCKSNVCYSNHVIAATAKSLDEX

>82_5

CALPISTIVGCSCKSNVCYSNHVIAATAKSLDEX

>83_4

TGVQTCALPISTIVGCSCKSNVCYSNHVIAATA

>84_6

ALPISTIVGCSCKSNVCYSNHVIAATAKSLDEX

>85_4

TGVQTCALPISTIVGCSCKSNVCYSNHVIAATA

>87_6

ALPISTIVGCSCKSNVCYSNHVIAATAKSLDEX

>88_6

ALPISTIVGCSCKSNVCYSNHVIAATAKSLDEX

>89_4

QTCALPISTIVGCSCKSNVCYSNHVIAATAKSL

>90_4

QTCALPISTIVGCSCKSNVCYSNHVIAATAKSL

>91_4

QTCALPISTIVGCSCKSNVCYSNHVIAATAKSL

>94_5

QTCALPISTIVGCSCKSNVCYSNHVIAATAKSLX

>95_4

QTCALPISTIVGCSCKSNVCYSNHVIAATAKSL

>96_6

TGVQTCALPISTIVGCSCKSNVCYSNHVIAATA

>97_6

ALPISTIVGCSCKSNVCYSNHVIAATAKSLDEX

>98_4

QTCALPISTIVGCSCKSNVCYSNHVIAATAKSL

>99_4

TCALPISTIVGCSCKSNVCYSNHVIAATAKSLD

>100_6

TGVQTCALPISTIVGCSCKSNVCYSNHVIAATA

>101_6

ALPISTIVGCSCKSNVCYSNHVIAATAKSLDEX

>102_6

ALPISTIVGCSCKSNVCYSNHVIAATAKSLDEX

>103_6

ALPISTIVGCSCKSNVCYSNHVIAATAKSLDEX

>104_5

GVQTCALPISTIVGCSCKSNVCYSNHVIAATAKX

>105_4

ALPISTIVGCSCKSNVCYSNHVIAATAKSLDEH

>106_6

ALPISTIVGCSCKSNVCYSNHVIAATAKSLDEX

>107_4

QTCALPISTIVGCSCKSNVCYSNHVIAATAKSL

**Multiple sequence alignment:**

19_4 A*VTGVQTC**ALPISTIVGCSCKSNVCYSN**HVIA---------- 32

62_4 -*VTGV*TC**ALPISTIVGCSCKSNVCYSN**HVIAA--------- 31

73_5 ---------**ALPISTIVGCSCKSNVCYSN**HVIAATAKSLDEHX 34

105_4 ---------**ALPISTIVGCSCKSNVCYSN**HVIAATAKSLDEH- 33

2_4 -------TC**ALPISTIVGCSCKSNVCYSN**HVIAATAKSLD--- 33

99_4 -------TC**ALPISTIVGCSCKSNVCYSN**HVIAATAKSLD--- 33

6_5 --------C**ALPISTIVGCSCKSNVCYSN**HVIAATAKSLDEX- 34

8_6 ---------**ALPISTIVGCSCKSNVCYSN**HVIAATAKSLDEX- 33

10_6 ---------**ALPISTIVGCSCKSNVCYSN**HVIAATAKSLDEX- 33

22_5 --------C**ALPISTIVGCSCKSNVCYSN**HVIAATAKSLDEX- 34

23_5 --------C**ALPISTIVGCSCKSNVCYSN**HVIAATAKSLDEX- 34

24_6 ---------**ALPISTIVGCSCKSNVCYSN**HVIAATAKSLDEX- 33

25_5 --------C**ALPISTIVGCSCKSNVCYSN**HVIAATAKSLDEX- 34

31_6 ---------**ALPISTIVGCSCKSNVCYSN**HVIAATAKSLDEX- 33

34_6 ---------**ALPISTIVGCSCKSNVCYSN**HVIAATAKSLDEX- 33

41_6 ---------**ALPISTIVGCSCKSNVCYSN**HVIAATAKSLDEX- 33

44_6 ---------**ALPISTIVGCSCKSNVCYSN**HVIAATAKSLDEX- 33

47_6 ---------**ALPISTIVGCSCKSNVCYSN**HVIAATAKSLDEX- 33

55_5 --------C**ALPISTIVGCSCKSNVCYSN**HVIAATAKSLDEX- 34

64_6 ---------**ALPISTIVGCSCKSNVCYSN**HVIAATAKSLDEX- 33

65_5 --------C**ALPISTIVGCSCKSNVCYSN**HVIAATAKSLDEX- 34

66_5 --------C**ALPISTIVGCSCKSNVCYSN**HVIAATAKSLDEX- 34

69_6 ---------**ALPISTIVGCSCKSNVCYSN**HVIAATAKSLDEX- 33

71_5 --------C**ALPISTIVGCSCKSNVCYSN**HVIAATAKSLDEX- 34

72_6 ---------**ALPISTIVGCSCKSNVCYSN**HVIAATAKSLDEX- 33

78_6 ---------**ALPISTIVGCSCKSNVCYSN**HVIAATAKSLDEX- 33

82_5 --------C**ALPISTIVGCSCKSNVCYSN**HVIAATAKSLDEX- 34

84_6 ---------**ALPISTIVGCSCKSNVCYSN**HVIAATAKSLDEX- 33

87_6 ---------**ALPISTIVGCSCKSNVCYSN**HVIAATAKSLDEX- 33

88_6 ---------**ALPISTIVGCSCKSNVCYSN**HVIAATAKSLDEX- 33

97_6 ---------**ALPISTIVGCSCKSNVCYSN**HVIAATAKSLDEX- 33

101_6 ---------**ALPISTIVGCSCKSNVCYSN**HVIAATAKSLDEX- 33

102_6 ---------**ALPISTIVGCSCKSNVCYSN**HVIAATAKSLDEX- 33

103_6 ---------**ALPISTIVGCSCKSNVCYSN**HVIAATAKSLDEX- 33

106_6 ---------**ALPISTIVGCSCKSNVCYSN**HVIAATAKSLDEX- 33

7_4 ------QTG**ALPISTIVGCSCKSNVCYSN**HVIAATAKSL---- 33

61_6 -------TC**ALPISTIVGCSCKSNVCYSN**HVIAATAKSLX--- 33

9_4 ------QTC**ALPISTIVGCSCKSNVCYSN**HVIAATAKSL---- 33

18_4 ------QTC**ALPISTIVGCSCKSNVCYSN**HVIAATAKSL---- 33

29_4 ------QTC**ALPISTIVGCSCKSNVCYSN**HVIAATAKSL---- 33

30_4 ------QTC**ALPISTIVGCSCKSNVCYSN**HVIAATAKSL---- 33

36_4 ------QTC**ALPISTIVGCSCKSNVCYSN**HVIAATAKSL---- 33

37_4 ------QTC**ALPISTIVGCSCKSNVCYSN**HVIAATAKSL---- 33

38_5 ------QTC**ALPISTIVGCSCKSNVCYSN**HVIAATAKSLX--- 34

40_4 ------QTC**ALPISTIVGCSCKSNVCYSN**HVIAATAKSL---- 33

45_4 ------QTC**ALPISTIVGCSCKSNVCYSN**HVIAATAKSL---- 33

46_4 ------QTC**ALPISTIVGCSCKSNVCYSN**HVIAATAKSL---- 33

51_5 ------QTC**ALPISTIVGCSCKSNVCYSN**HVIAATAKSLX--- 34

53_5 ------QTC**ALPISTIVGCSCKSNVCYSN**HVIAATAKSLX--- 34

56_4 ------QTC**ALPISTIVGCSCKSNVCYSN**HVIAATAKSL---- 33

63_4 ------QTC**ALPISTIVGCSCKSNVCYSN**HVIAATAKSL---- 33

68_5 ------QTC**ALPISTIVGCSCKSNVCYSN**HVIAATAKSLX--- 34

70_4 ------QTC**ALPISTIVGCSCKSNVCYSN**HVIAATAKSL---- 33

89_4 ------QTC**ALPISTIVGCSCKSNVCYSN**HVIAATAKSL---- 33

90_4 ------QTC**ALPISTIVGCSCKSNVCYSN**HVIAATAKSL---- 33

91_4 ------QTC**ALPISTIVGCSCKSNVCYSN**HVIAATAKSL---- 33

94_5 ------QTC**ALPISTIVGCSCKSNVCYSN**HVIAATAKSLX--- 34

95_4 ------QTC**ALPISTIVGCSCKSNVCYSN**HVIAATAKSL---- 33

98_4 ------QTC**ALPISTIVGCSCKSNVCYSN**HVIAATAKSL---- 33

107_4 ------QTC**ALPISTIVGCSCKSNVCYSN**HVIAATAKSL---- 33

26_4 ---TGVQTC**ALPISTIVGCSCKSNVCYSN**HVIAATG------- 33

3_6 ---TGVQTW**ALPISTIVGCSCKSNVCYSN**HVIAATA------- 33

58_4 ---TGVQTC**ALPISTIVGCSCKSNVCYSN**HVIAATA------- 33

83_4 ---TGVQTC**ALPISTIVGCSCKSNVCYSN**HVIAATA------- 33

85_4 ---TGVQTC**ALPISTIVGCSCKSNVCYSN**HVIAATA------- 33

96_6 ---TGVQTC**ALPISTIVGCSCKSNVCYSN**HVIAATA------- 33

100_6 ---TGVQTC**ALPISTIVGCSCKSNVCYSN**HVIAATA------- 33

60_4 ----GVQTC**ALPISTIVGCSCKSNVCYSN**HVIAATAK------ 33

104_5 ----GVQTC**ALPISTIVGCSCKSNVCYSN**HVIAATAKX----- 34

13_6 ------QTC**ALPISTIVGCSCKSNVCYSN**HVIAATAKSX---- 33

39_5 -----VQTC**ALPISTIVGCSCKSNVCYSN**HVIAATAKSX---- 34

14_4 -----VQTC**ALPISTIVGCSCKSNVCYSN**HVIAATAKS----- 33

28_4 -----VQTC**ALPISTIVGCSCKSNVCYSN**HVIAATAKS----- 33

************************

**Possible sequence: QTCALPISTIVGCSCKSNVCYSN**
